# Fauna Europaea – all European animal species on the web

**DOI:** 10.3897/BDJ.2.e4034

**Published:** 2014-09-17

**Authors:** Yde de Jong, Melina Verbeek, Verner Michelsen, Per de Place Bjørn, Wouter Los, Fedor Steeman, Nicolas Bailly, Claire Basire, Przemek Chylarecki, Eduard Stloukal, Gregor Hagedorn, Florian Tobias Wetzel, Falko Glöckler, Alexander Kroupa, Günther Korb, Anke Hoffmann, Christoph Häuser, Andreas Kohlbecker, Andreas Müller, Anton Güntsch, Pavel Stoev, Lyubomir Penev

**Affiliations:** † University of Eastern Finland, Joensuu, Finland; ‡ University of Amsterdam - Faculty of Science, Amsterdam, Netherlands; § Unaffiliated, Basel, Switzerland; | Natural History Museum of Denmark, Copenhagen, Denmark; ¶ Danish Agency for Digitisation, Copenhagen, Denmark; # University of Amsterdam, Amsterdam, Netherlands; †† Unaffilated, Copenhagen, Denmark; ‡‡ WorldFish Center, Los Baños, Philippines; §§ ISUPNAT, Viroflay, France; || Institute of Zoology, Warsaw, Poland; ¶¶ Department of Zoology, Comenius University, Bratislava, Slovakia; ## Museum für Naturkunde Berlin, Leibniz Institute for Evolution and Biodiversity Science, Berlin, Germany; ††† FU Berlin, Berlin, Germany; ‡‡‡ National Museum of Natural History and Pensoft Publishers, Sofia, Bulgaria; §§§ Institute of Biodiversity & Ecosystem Research, Bulgarian Academy of Sciences and Pensoft Publishers, Sofia, Bulgaria; ||| Pensoft Publishers, Sofia, Bulgaria

**Keywords:** Biodiversity Informatics, Animals, nomenclature, taxonomy, Fauna Europaea, Taxonomic indexing, Taxonomic standard, INSPIRE, Taxonomic reference, Taxonomic checklist

## Abstract

Fauna Europaea is Europe's main zoological taxonomic index, making the scientific names and distributions of all living, currently known, multicellular, European land and freshwater animals species integrally available in one authoritative database. Fauna Europaea covers about 260,000 taxon names, including 145,000 accepted (sub)species, assembled by a large network of (>400) leading specialists, using advanced electronic tools for data collations with data quality assured through sophisticated validation routines. Fauna Europaea started in 2000 as an EC funded FP5 project and provides a unique taxonomic reference for many user-groups such as scientists, governments, industries, nature conservation communities and educational programs. Fauna Europaea was formally accepted as an INSPIRE standard for Europe, as part of the European Taxonomic Backbone established in PESI.

Fauna Europaea provides a public web portal at faunaeur.org with links to other key biodiversity services, is installed as a taxonomic backbone in wide range of biodiversity services and actively contributes to biodiversity informatics innovations in various initiatives and EC programs.

## Project description

*Fauna Europaea* kicked-off as a European Commission (EC) funded project, starting March 2000. It provides a web-based information infrastructure with an index of scientific names (including important synonyms) of all living European land and freshwater animals, their geographical distribution at country level (up to the Ural mountains, excluding The Caucasus region), and some additional, optional information.

### Why Fauna Europaea?

The European Commission has published the Community Biodiversity Strategy to provide a framework for the development of Community policies and instruments in order to comply with the Convention on Biological Diversity. With regard to biodiversity in Europe, both science and policies depend on a knowledge of its components. The assessment of biodiversity, monitoring of changes, sustainable exploitation of biodiversity, and much legislative work depend upon a validated overview of taxonomic biodiversity. The Strategy recognises the incomplete state of knowledge at all levels concerning biodiversity, which is a constraint on the successful implementation of the Convention.

Fauna Europaea contributes to the European Community Biodiversity Strategy by supporting one of the main themes of the Strategy: to identify and catalogue the components of European biodiversity into a single database to serve as a basic tool for science and conservation policies. Prior to Fauna Europaea such a taxonomic index did not exist. Partial overviews were scattered around Europe in different scientific institutes, while only some countries were working on national information systems. Fauna Europaea integrates all efforts to establish a taxonomic standard for Europe on terrestrial and freshwater animal taxonomy, meaning Fauna Europaea includes a widely shared scientific consensus, contains highly scrutinised data (in terms of correctness of spelling, etc.) and is commonly used as a reference file in zoo-taxonomy.

### Fauna Europaea background

Fauna Europaea started in 2000 as an European Commission (EC) Fifth Framework (FP5) four-years project, delivering its first release in September 2004. Project coordination and management were in hands of the University of Amsterdam (then: Zoological Museum Amsterdam), two other principal partners included the Zoological Museum, Natural History Museum of Denmark (then: Zoological Museum of Copenhagen) and the Museum National d’Histoire Naturelle in Paris.

During the initial four years, the Fauna Europaea project successfully accomplished the extensive and innovative work-plan (Suppl. material 14), delivering the projected knowledge networks, information infrastructures, and dissemination services. Fauna Europaea was actively involved in a wide range of associated initiatives in biodiversity informatics and has strong institutional outreach, contributing to a range of networking activities in biodiversity science, as a knowledge hub on high-level taxonomic expertise.

After fifteen years of steady operation, the hosting of Fauna Europaea was migrated to the Museum für Naturkunde (MfN) in Berlin who play a key-role in on-going biodiversity networking programs. This includes the development of a new generation of virtual tools and workbenches and supporting data interoperability and biodiversity science as a community effort.

 In order to improve the dissemination of Fauna Europaea and acknowledge the Fauna Europaea contributors, this Special Issue has been compiled using novel e-Publishing tools to prepare data-papers of all major Fauna Europaea taxonomic groups. This article provides the general background of the Fauna Europaea project to date and described the release of the initial series of data-papers.

## Material and methods

To ensure the collation of high quality data a network of specialists, including Group Coordinators coordinating the efforts in particular taxonomic groups, are contracted. Advanced on-line and off-line tools for data import and data management were developed. Sophisticated procedures for data verification were applied, including a review procedure on the inclusiveness and quality of the data sets. Further validation was taken care by a network of national Focal Points and other thematic partners, fully supported by the virtual infrastructure. The sections below will highlight some aspects of the established Fauna Europaea social and technical infrastructures.

### Guidelines & Standards

The Fauna Europaea project provided 'Guidelines for Group Coordinators and Taxonomic Specialists' (Suppl. material 1), including the standards, protocols, scope, and limits that constituted the instructions for about around 500 specialists contributing to the project. An initial version of the standards document was established in the first year of the project, which has been refined over subsequent years. The following sections provide a summary of the essential points from the Fauna Europaea taxonomic guidelines:

- The focus is on species and subspecies where appropriate of European multicellular animals of terrestrial and freshwater environments. Recommendations are given for inclusion or exclusion of domesticated animals and exotic intruders, animals of brackish waters, extinct species etc.
- Classification of animal species deals with the placement of taxa in a hierarchical system of ranks. It is recommended to keep the hierarchy of taxon ranks as simple as possible, especially at higher levels, because the focus is on the species level.
- Nomenclature of animal species deals with the formation and treatment of scientific names. All formalities of nomenclature must follow the rules and provisions of the latest (= 4^th^) edition of  ‘International Code of Zoological Nomenclature’. Synonymy, the most troublesome aspect of nomenclature, is explained in some detail. It is a well-known phenomenon that a species or other taxon is referred to under multiple names.
- Species names are binomial (a combination of a generic and a specific name). In zoology (after the first description) combinations are not formalised by the code of nomenclature. Also synonymy is not primary linked to the combination, but to the respected species-group, genus-group or higher rank names. Therefore also in Fauna Europaea taxonomic records are name based, meaning split into generic and specific names, and into accepted names and synonymous names (i.e. different names used for the same taxon). This is also reflected in the database model (see below).

### Taxonomic scope

Taxonomic scope includes issues like, (1) the definition of taxonomic and biological criteria to assure species in Fauna Europaea only includes natural, stable populations (not artefacts or incidental and unnatural occurrences), (2) the taxonomic hierarchy, i.e. the building of a classification scheme and (3) nomenclature, i.e. the structuring of detailed information about accepted and synonymous genus- and species-group names.

A general synopsis on how to deal with aspects of taxonomy and nomenclature in the Fauna Europaea project is available as part of the mentioned 'Guidelines'. An excerpt is given below.

**Taxonomic criteria**

The taxonomic scope has proved to be by far the most controversial part of this project. What satisfy the entering of a species name into Fauna Europaea database? Obviously, an “accepted species” should be living, multi-cellular, non-marine animal species with a documented occurrence in Europe. However, there is more to it. An “accepted species” in a Fauna Europaea context should also be scientifically named and described according to the regulations of the International Code of Zoological Nomenclature. Further, if there exist two or more synonymous names for the same species, it is the oldest valid name that should be used. Even by these additional criteria it is unavoidable that specialists have to face difficult decisions, which are bat est, made on a mixture of common sense and tradition. The following are species categories that should normally be excluded, but potentially contain species that need to be included:

1. species occupying the marine/freshwater or marine/terrestrial transition zones
2. rare, irregular immigrants (some birds, butterflies etc.)
3. accidental or deliberate releases of exotic (pet)species 
4. foreign species imported and released for bio-control
5. foreign species largely confined to hothouses

Even more problematic is the *nomina dubia* category, that is names of “phantom species” that cannot be safely associated with any living species. Such names occur most frequently in species-rich, poorly investigated groups like parasitic wasps and spiders. *Nomina dubia* tend to blur the picture of known species diversity in a given geographical area. This is why the Fauna Europaea project has made substantial efforts to encourage specialists not to include any such “phantom species”. Unfortunately, the specialists often feel disinclined to categorize a nominal species as a *nomen dubium*, even if the name has remained obscure for many decades. One major reason could be that the FaEu database cannot presently accommodate this “third” category of names that neither belongs to the accepted names or synonymous names categories. One aspect of the future updating of the Fauna Europaea database will be the removal of any “surviving” *nomina dubia* from the lists of accepted species.

**Taxonomic hierarchy**

The higher taxonomic hierarchy ranging from phylum to family for the European fauna was the first major accomplishment of Fauna Europaea project. The hierarchy includes the universally accepted Linnean hierarchy categories:

Kingdom – Subkingdom – Phylum – Subphylum – Infraphylum – Class – Subclass – Superorder – Order – Suborder – Infraorder – Superfamily – Family – Subfamily – Tribe – Subtribe – Genus – Subgenus – Species – Subspecies

All species covered by Fauna Europaea belong to Kingdom Animalia.

It is important to underline that the Fauna Europaea hierarchy does not purport to be the phylogenetically “most correct” one. There is considerable disagreement between taxonomists about what is the best hierarchy, and anything like a consensus is not within view. For purely managerial purposes, however, Fauna Europaea needed to settle on one common hierarchy.

Some categories (e.g. suborder, subgenus and subspecies) are not applicable to all taxa. This may be because a category may not be generally applied in the current taxonomy of the group, or because the group coordinator has chosen not to provide this information (see below under the format of names).

The higher hierarchy (categories above superfamily) used for the project are shown in the Fauna Europaea Guidelines document. The full hierarchy can be browsed on the Fauna Europaea, the original version can be downloaded from the website, the latest version can be found here: Suppl. material 11.

**The format of names**

Group coordinators and taxonomic specialists had to deliver the names according to very specific standards. The names provided by FaEu are *scientific names* (also known as “Latin names” although they are of Greek origin in very many cases). Many European animal species also have names in national languages, but these are not yet part of the FaEu service.

The scientific name of an animal species basically consists of two parts:

*(1) Genus group names*  (Generic name)

For each generic name, the following information was required:

- Containing family
- Containing subfamily, tribe and/or subtribe (optional)
- Author
- Year of publication

For a subgeneric name (located between the generic and the specific name), information was in addition required about the containing genus.

*(2) Species group names*  (Specific name)

For each specific name, the following information was required

- Containing genus
- Containing subgenus (optional)
- Author
- Year of publication
- Original genus, i.e., the genus in which the species was originally described
- Whether “Author, Year” information should be presented in parentheses or not (Y/N). Parentheses around the author citation indicate that this was not the original taxonomic placement.

For a subspecific name (appended after the specific name), information was in addition required about the containing species.

*Synonyms*

Synonymy arises when different names refer to the same taxon and is a very important issue in biosystematics. One and the same taxon (genus or species) may have been described under different names by different authors (or by the same author at different times), or a name may have been wrongly applied to a taxon due to misidentification (actually a misapplied usage of a name). In many cases, different names have traditionally been used in different European countries for the same species. In other cases, some authorities regard two taxa as different species, whereas some regard them as subspecies of the same species (so conflicting taxonomic concepts). For practising taxonomists, synonymy is a major issue resulting from subjective assertions made by authors. For Fauna Europaea however, the provision of synonymies as such is not a main goal. However, Fauna Europaea groups coordinators and taxonomic specialists were asked to provide synonyms, at least such synonyms which would be likely to cause confusion.

The relevance of including synonymy is not similar between all groups. Synonymy is much more important information in less well-understood groups than in the ones where the species are non-controversial. In Fauna Europaea group coordinators and taxonomic specialists have provided synonyms to very different degrees of completeness, ranging from no synonyms at all (Examples: Aves, Reptilia, Amphibia) to virtually complete inventories of synonyms (Examples: Hemiptera: Cicadomorpha & Fulgoromorpha).

Synonyms are of two principally different kinds:

- ”True” synonyms are those that can be formally cited with authorship & date (see the FaEu Guidelines for examples).
- Variant spellings, unavailable names or misapplied names.

Synonyms of the latter category are marked in the FaEu database with “auct.”, instead of “Author, Year” information. “auct.” is short for “auctorum”, meaning “of authors” in Latin.

In order to handle synonymy FaEu chose to deal with generic (including subgeneric) names and specific (including subspecific) names separately.

*Spelling of species names in Lepidoptera*

It has become practice among Lepidopterologists to use the original spellings of the species names and not to follow Article 31.2 and 34.2 of the International Code of Zoological Nomenclature (Sommerer 2002), which requires adjectival species names to be in agreement with the gender of the genus name. For reasons of stability and interoperability with major on-line databases (Such as the Species 2000 Global Lepidoptera name database) we have chosen to show in Fauna Europaea primarily the original spelling of the specific names of the Lepidoptera. For several groups we also add(ed) as an alternative the spelling in agreement with the gender of the genus, but only for ‘real’ Latin or Greek adjectives. This means that for names with ‘invented endings’  -ella, -ana, -ata, -dactyla, we always only follow original spelling. In this version, we haven’t been able to do this for all groups, and mistakes are also likely to occur. This practice follows the resolution and recommendation by the Societas Europaea Lepidopterologica (SEL).

*Spider nomenclature*

Although generally the International Code of Zoological Nomenclature doesn't except species descriptions before 1758 as 'valid names' and the publication date for both the Systema Naturae (10th edition) and Clerck's 'Svenska Spindlar' (Clerck 1757) are set by the ICZN on 1 January 1958, for spiders we followed the Zoological Code Direction (104) allowing Clerck 1757, also used in Platnick's World Spider Catalog.

### Taxonomic framework

During the project phase of Fauna Europaea, the Zoological Museum of Copenhagen collated the individual data sets for Fauna Europaea, which were sent to the Zoological Museum Amsterdam for merging into the integrated database. Member institutes of the project are involved by providing taxonomic expertise and information and expert networks taking care about data collation. A selected small network of (67) group coordinators keeps contact with the large number of around 500 experts throughout Europe to capture data files on parts of the various taxonomic groups. Every taxonomic group is covered by at least one group coordinator. Group coordinators are responsible for the supervision and integrated input of both taxonomic and distributional data. The formal responsibility of collating and delivering the data of relevant families has resided with the Taxonomic Specialists.

After the project formal lifetime (2004–2014) the Zoological Museum Amsterdam took over all expert networking and data management tasks.

As a unique feature, Fauna Europaea funds were set aside for paying/compensating for the work of taxonomic specialists and group coordinators. Five euro per accepted species record was offered, although in some cases, where full data on a species could not be provided (typically when only taxonomic information, but no faunistic information, was provided), only partial payment was given. Group coordinators made their own arrangements with “their” taxonomic specialists about how the money was to be distributed between the two levels of collaborators.

### Geographic scope

**Figure 1.**
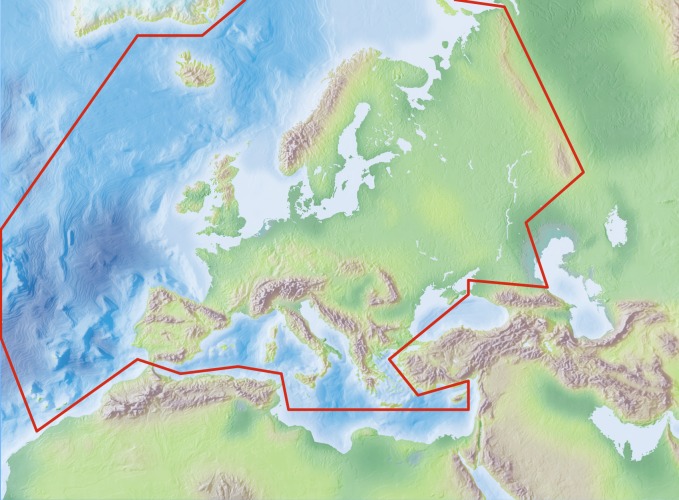
Fauna Europaea geographic coverage ('minimal Europe').

Following the Fauna Europaea contract, species and subspecies should be registered at least at the level of political countries, meaning political countries. The Fauna Europaea geographical system follows basically the ISO 3166 and TDWG 2.0 country code standards with minor modifications (see Suppl. material 2). The covered area will be the same as European mainland (Western Palearctic), plus the Macaronesian islands (excluding Cape Verde Islands), Cyprus, Franz Josef Land and Novaya Zemlya, the Western Kazakhstan excluded (Fig. 1).

The geographic boundaries includes: East: Ural  (E 60°), West: Atlantic Ocean (Mid-Atlantic Ridge) (W 30°), South: Mediterranean (N 35°), North: Arctic Islands  (N 82°).

For details see the FaEu Guidelines chapter 3: Geographic information (Suppl. material 1).

### Validation Framework & Quality Control

**Figure 2.**
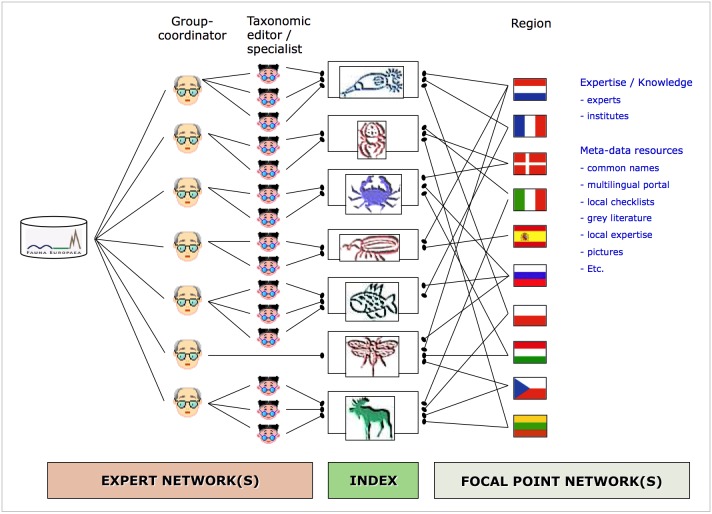
Fauna Europaea Expert network(s) versus Focal Points network(s).

**Figure 3.**
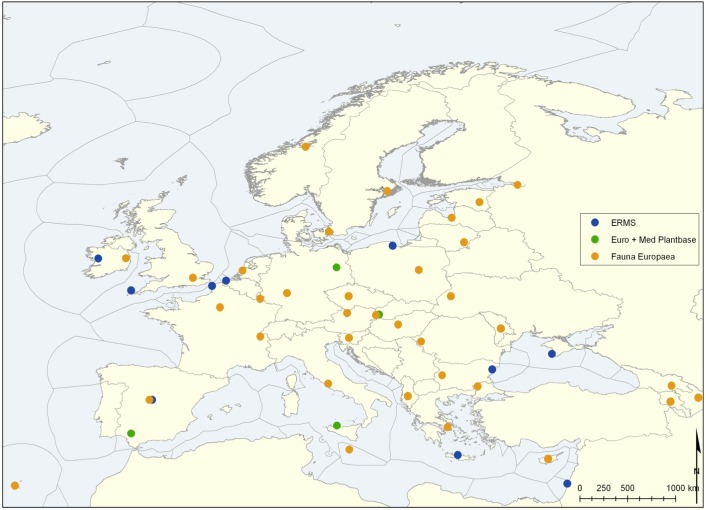
PESI Focal Point network.

Fauna Europaea data are unique in a sense that they are fully expert based, meaning that the database is build from scratch by leading experts, critically reviewing and evaluating all available information (collections, observations, research, etc.), not by simply merging data from available resources like faunas, monographs, checklists or collection data. For distributional details local checklists are only used selectively (when scrutinised). Furthermore, all Fauna Europaea data sets are intensively reviewed and scrutinised. Five basic procedures to check and improve the quality of the data have been effected:

(1) During the project phase of Fauna Europaea, the National Museum of Natural History in Paris was in charge for validating the delivered data sets by means of cross-checking and reviewing with existing national, regional and local resources (checklists and literature), following the criteria on data standards, data semantics, data accuracy, data reliability, and inclusiveness of data as developed for Fauna Europaea. For this purpose a semi-automatic review routine was installed reporting inconsistencies between different features of the data set (Hierarchy, Taxonomy, Faunistic and Reference) and external resources. The outcomes were discussed with the Fauna Europaea editors.

(2) In addition, for both off-line and on-line data entry systems checks on the technical and logical correctness for all data entered by the taxonomic editors have been implemented as ‘Taxonomic Integrity Rules’. This validation tool proved to be of huge value for both the experts and project management, and significantly contributed to the preparation of a remarkably clean and consistent data set. The original set of ‘Taxonomic Integrity Rules’ was extended in follow-up projects like EDIT and PESI.

(3) In order to have a better estimate of the present distribution of terrestrial species in Europe, to spread the Fauna Europaea information and share taxonomic knowledge with local specialists, regional validation workshops have been held in areas relevant for their importance in terms of species richness or endemicity. As a result of this collaboration a network of associated specialists, representing experts from different regions, was established, taking care of the long-term reviewing of the Fauna Europaea data, providing feedback to the editors.

(4) In parallel, a program was initiated on validating Fauna Europaea with help of National Focal Points (Fig. 2), involving local taxonomic experts, cross-checking Fauna Europaea data with national species lists or other local resources (see 'NAS extension' below). Later this program was continued and extended as part of the PESI project, installing the so-called PESI Focal Point network (Fig. 3), using the PESI portal validation services for cross-validation.

(5) Finally, feedback was provided by users via the Fauna Europaea web form, which are forwarded to the relevant experts.

Since the initial project set-up, Fauna Europaea contributed significantly to programs and initiatives analysing gaps in knowledge, data and expertise, to provide relevant recommendations for the European Commission for implementation in policy and research, including ENBI, EDIT, SMEBD, EPBRS and – most recently – EU BON.

### IT services and data management

**Figure 4.**
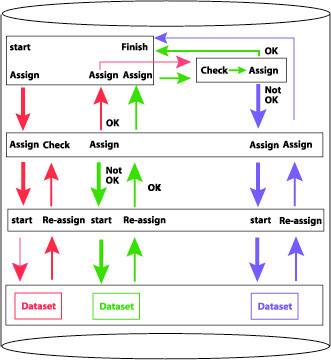
Definition of user-roles and data-flow within Fauna Europaea.

In Fauna Europaea all data editing is done on a central system by the experts themselves. By evaluating team structure and life-cycle procedures (data-entry, validation, updating, etc.), clear definitions of roles of users and user-groups, according to the taxonomic framework were established, including ownership, and read and write privileges, and their status changes during the project data-flow (Fig. 4). In addition, guidelines on common data exchange formats and codes have been issued.

**Figure 5.**
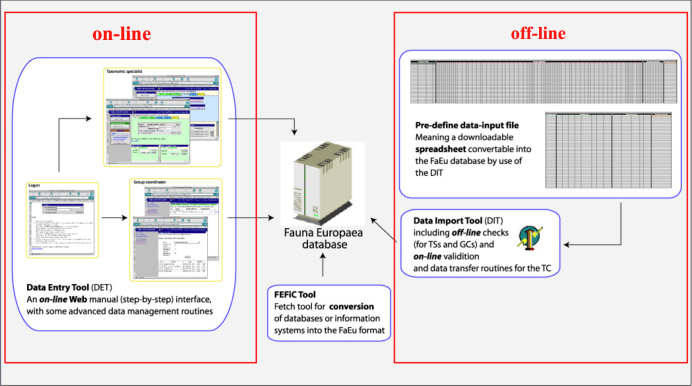
Fauna Europaea on-line (web-interface) and off-line (spreadsheet) data import routines.

To facilitate data transfer and data import within the Fauna Europaea database on-line and off-line data-entry routines have been build (Fig. 5), including:

(1)  An on-line Web interface data-entry tool (step-by-step through web-interfaces).

(2)  An off-line data import tool by use of a pre-defined spreadsheet file; including validation checks, and inventive data import and export routines.

For details on the data-entry tool functional interfaces, please consider the respective manuals.

**Figure 6.**
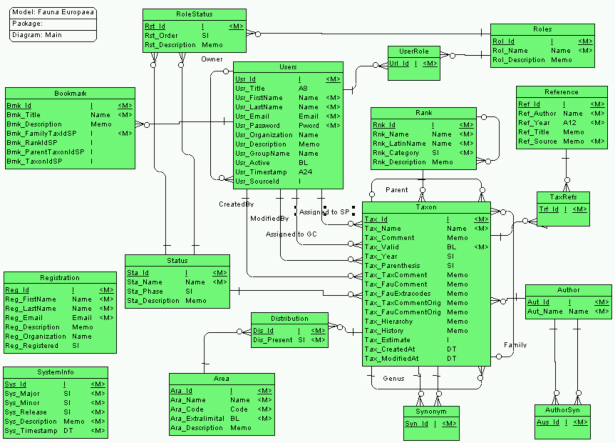
Fauna Europaea data model.

Based on the functional and technical requirements a database model has been developed (Fig. 6), including components to manage taxonomic data, distributional details, references and user information. Fauna Europaea follows a typical name-based data model proceeding from a 'Taxon table' including names as single entities (uninomials/monomials), defining the classification, name combinations and synonymy as recursive relationships between names. This model is able to handle the complex issues of zoological nomenclature and synonymy.

The final Fauna Europaea server-infrastructure included an Oracle RDBMS transaction-server interconnected to a set of tools for data entry and data transfer, establishing an advanced virtual environment for data import and management, which well performed for more then 10 years and an Oracle RDBMS production-server linked to the Fauna Europaea web portal.

In 2009 the Fauna Europaea servers moved to a Virtual Machine. In 2013 the Fauna Europaea web portal hosting was taken over by the Museum für Naturkunde (MfN) in Berlin. The migration of the Fauna Europaea data management environment towards the EDIT Platform for Cybertaxonomy is currently in progress, which also includes a new web-portal.

### Focal points and NAS extension

**Figure 7.**
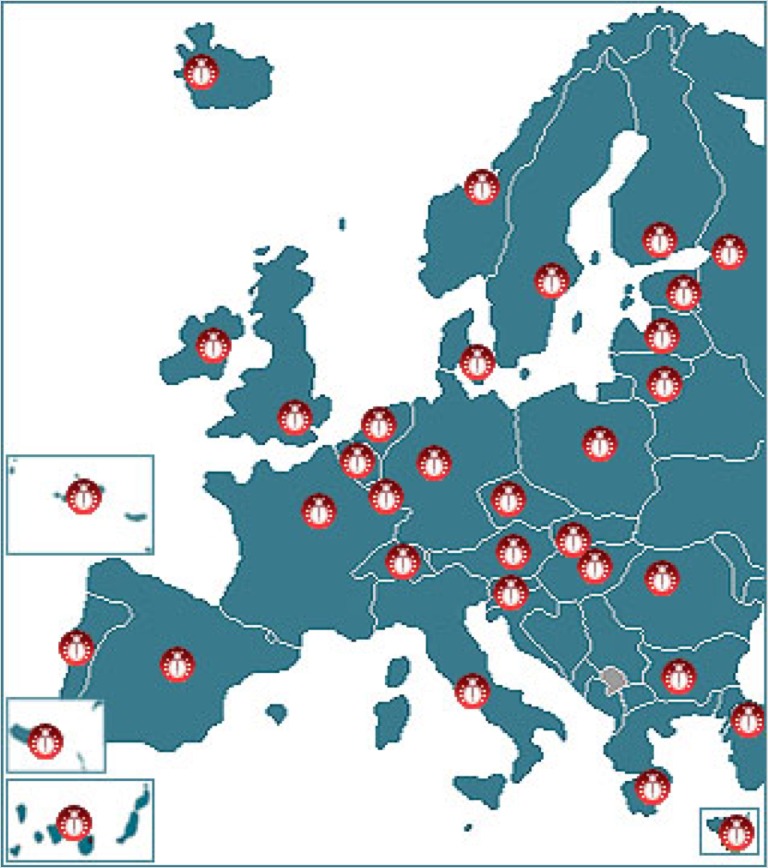
Fauna Europaea initial Focal Points network in NAS extension. See also: http://www.faunaeur.org/focal_point.php.

In the initial part of the Fauna Europaea project it was concluded that a stronger involvement of institutes and experts, to support ‘regional/national validation’ and to harmonise on-going activities, was desirable. Therefore a network of national partner institutes was established, acting as 'Focal Points' for regional or national liaison and supporting the taxonomic framework. In most cases these institutes maintain or host national checklists and provide access to information about experts and literature resources in a country (Fig. 7).

The importance on developing contacts with national partner institutes was particularly relevant for Eastern Europe, because in the initial phase the institutional outreach of Fauna Europaea was rather oriented on Western Europe. In the fall of 2001, a call from the European Commission was published, which asked for proposals to extend themselves to the so-called NAS countries (Newly Associated States). A Fauna Europaea proposal for such an extension was approved by the EC early 2002. As a results an extended network of Focal Points was established and a detailed work program launched on the sharing of meta-data on taxonomic resources and the validation of Fauna Europaea by cross-checking with help of local checklists and expertise.

Later, as part of the pan-European Species-directory Infrastructure (PESI) project, the Fauna Europaea Focal Points have been merged with the networks of the European Register of Marine Species (ERMS), Euro+Med PlantBase (E+M) and Index Fungorum (IF) (see above).

### Intellectual property rights and community management

An important aspect of the Fauna Europaea expert agreement is that experts keep the ownership on their data delivered, meaning that the rights of Fauna Europaea to disseminate the data are non-exclusive. This agreement was formalised by signing contracts Suppl. material 3 with all group coordinators and specialists after the project.

In addition, it was decided to search for a relevant mechanism to keep the involvement and secure the joint intellectual property rights of all contributors, which resulted in the joint membership of the "Society for the Management of Electronic Biodiversity Data" (SMEBD). SMEBD is a society, which on behalf of its members, attempts to provide a legal basis for the protection of their data as well as promotes the interest of experts on issues related to data governance and dissemination.

For (the substantial number of) Fauna Europaea expert employed at European taxonomic institutes, support for their activities was received from the "Consortium of European Taxonomic Facilities" (CETAF), not only underlining the relevance of a European taxonomic standard, but also understanding Fauna Europaea as an important collaborative knowledge network on taxonomic expertise.

## Data resources

**Object name:** Fauna Europaea

**Resource link**: http://www.faunaeur.org

**Alternative identifier**: http://data.gbif.org/datasets/resource/13560

**Version:** 2.6.2

**Publication date of data:** 2013-08-29

**Language:** English & Latin

**Format name:** HTML / PHP

**Character encoding:** UTF-8

## Results

Fauna Europaea results in a unique overview of the state of art with respect to our understanding of the taxonomy and occurrence of European species and serves as a clearing house to identify taxonomic knowledge and expertise, including potential gaps. As a taxonomic data standard resource, Fauna Europaea provides a gateway serving the integration and sharing of European biodiversity data, supporting major biodiversity informatics initiatives, such as LifeWatch, EU BON, and GBIF, as well as serving many other stakeholders, such as national biodiversity portals, nature conservation agencies and biodiversity managers.

### Fauna Europaea main outcomes

The first release of the Fauna Europaea index was formally presented at the Fauna Europaea final meeting at the 27^th^ of September 2004 in Paris. In the following years new releases have been published at around a yearly sequence. Since the initial release, updates includes individual data sets (smaller or larger taxonomic sectors). The most recent release (version 2.6.2) was launched at 29 August 2013. An overview of Fauna Europaea main releases can be found here: http://www.faunaeur.org/about_fauna_versions.php.

Currently Fauna Europaea covers 221,701 accepted taxon names, including 146,288 accepted species and subspecies (Table 1), allocated to 58 taxonomic groups (Table 2). Homonym percentage in Fauna Europaea are trivial (less then 0.01%) compared to the total number of taxa. The few homonyms are well classifiable and annotatable as senior or junior homonyms comparing the authorships (Suppl. material 6).

The Fauna Europaea expert network includes 67 Group Coordinators and up to 500 specialists. Since 2004, several expert replacements took place, especially substituting retired or deceased specialists (see Table 2 on Group Coordinator changes). In general the expert network remained rather stable for the last 15 years, although the actual participation and policy to contribute to new updates varied significantly within and between the different taxonomic groups and data sectors. Some group coordinators remain very active and delivered new data sets for each release, others were more passive, but allowed taxonomic specialists to contribute individually, and for some groups neither the group coordinators were active nor the taxonomic specialists could contribute. During the last updating period around one third of the Fauna Europaea experts could be considered as 'active'.

**Figure 8.**
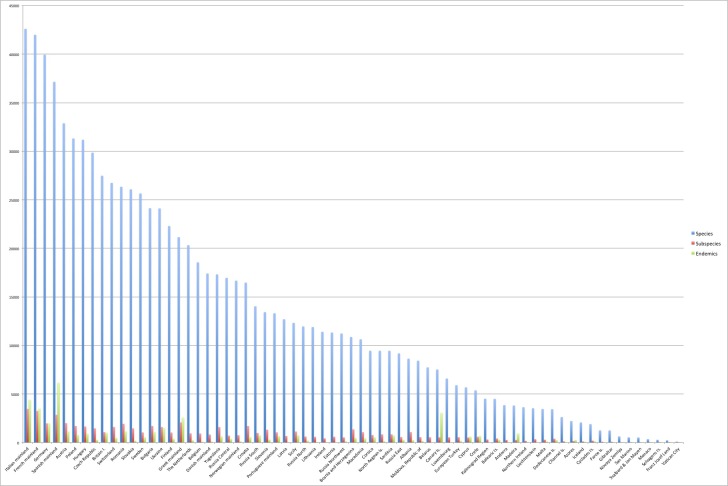
Fauna Europaea country/region statistics. Source data can be found at Suppl. materials 7, 8.

The geographic details of the Fauna Europaea data have been subject of various analyses within Europe (e.g. Essl et al. 2013, Fontaine et al. 2012) or with adjacent areas (Baron 2008). In general species richness shows a positive correlation with country size and lower altitude, endemicity being substantially higher in countries with island archipelagos (Fig. 8).

### Dissemination and cross-linking

Fauna Europaea data are firstly disseminated via a public web site at **faunaeur.org**. Fauna Europaea appears a popular site on taxonomic information considering the high web-usage statistics, including 637,535 unique visitors for 2013 (Suppl. material 9). A series of search interfaces (displays) have been developed focusing on different search options and showing relevant details (distribution, synonyms, classification) of the resulting taxa (Fig. 12). In the result page a number of direct links to other biodiversity resources have been implemented, including occurrence data (GBIF), libraries (Zoological Record, BHL, AnimalBase), multimedia data (Europeana), genetic data (GenBank/NCBI), vernacular names (PESI) and other checklists (CoL, ITIS).

In addition, Fauna Europaea is included as the terrestrial-zoological component of the *pan-European Species-directories Infrastructure* (PESI), representing the taxonomic backbone for Europe (see Fig. 15). As part of PESI, Fauna Europaea is selected as an INSPIRE directive (= formal taxonomic standard) for Europe. The second version of PESI was released at 4 March 2013. Fauna Europaea data are also accessed via copies hosted at other platforms. For this purpose around 80 formal licenses for downloads has been approved and disseminated since the Fauna Europaea initial release. Some licenses are regularly renewed, like the Fauna Europaea copy for the GBIF Checklist Bank and for PESI Focal Points.

Fauna Europaea is frequently used as a taxonomic reference in scientific papers (e.g. Hausmann et al. 2013, Mutanen et al. 2012, Padrón et al. 2009, Ramilo et al. 2012, Vervoort et al. 2012, Weigand et al. 2012). It is also referenced in nearly 5,000 wiki pages, and used as a resource for scientific analysis (e.g. Brewer et al. 2012, Entling et al. 2012, Essl et al. 2013) often using customised downloads.

Around 2005, the relevance of applying unique and stable species identifiers, to secure long-term internal consistency and optimise external interoperability was recognised in the biodiversity informatics community (Hussey et al. 2008). Following the zoological context of the Fauna Europaea database, species identifiers are released at a specific level, maintaining species-group names as rigid identifiers, not species names (combinations). A positive result of this feature is that species names always resolve towards their nominal taxa (the only fixed point in zoological nomenclature), a disadvantage is that new combinations, such as changed genus assignments of the same nominal species, have similar taxon IDs between different versions of Fauna Europaea (see also Discussion). To make the Fauna Europaea identifiers sustainable, the use of the (much appreciated) data import spreadsheet was stopped in 2009, because loading the spreadsheets required the removal of existing data set, including the taxonomic record IDs, which are the basis for the identifiers.

Lately so-called Uniform Resource Identifiers (URIs) have been established for Fauna Europaea, which dereference to the taxon respective pages (for instance: http://www.faunaeur.org/t/337992). URI's improve stable and unambiguous cross-referencing among biodiversity services and are more easy to be used by stakeholders. This was recently also recommended as part of the Bouchout Declaration.

A practical guideline on cross-linking Fauna Europaea can be found here: Suppl. material 10.

### Gaps in data and knowledge

After the first release of Fauna Europaea in 2004, the completeness of the received taxonomic data was calculated to include 99.3% of the known European fauna (actual number of databased species 128,692; estimated number of described species 129,647). The faunistic coverage is less complete, but nevertheless including 90-95% of the total fauna. Recognised gaps for major groups are given in Table 2 (figures from 'unfinished work' provided by the experts after the first release). The analysis of existing gaps is quite essential for evaluating the quality of the contained information and to provide evidence for the need to constantly curate and update the database. Particularly as there is ongoing work being done to close the gaps, a thorough gap analysis helps to outline taxonomic groups where increased efforts are needed, not only for the involved coordinators and involved specialists but also for future European biodiversity projects that are focusing on biodiversity information.

**Figure 9.**
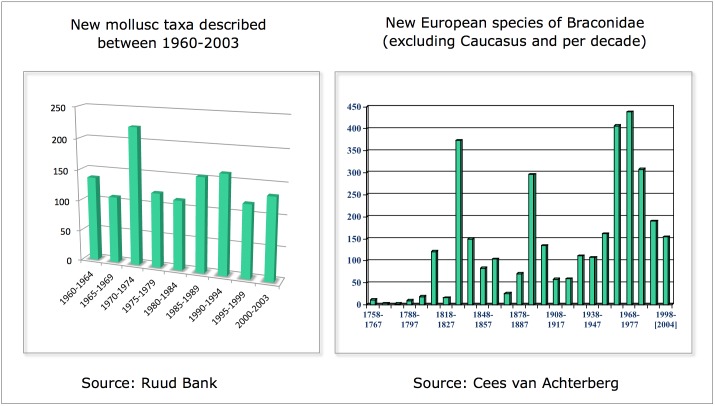
Statistics of newly described species per period in Molluscs (source: Ruud Bank) and Braconidae (source Cees van Achterberg).

The data-entry figures show a delay of around four years for entering newly described species into the Fauna Europaea database. Considering an average yearly increment of newly described animal species for Europe of around 670 species (figures from the Fauna Europaea gap analysis Suppl. material 4), the current taxonomic completeness is predicted to be 97.5% (actual number of databased species 132,100; estimated number of described species around 135,400). According to figures of the Zoological Record, however, the average yearly increment of newly described animal species in Europe could be less, namely around 220 species, although the description rate of new species significantly differs within and between taxonomic groups (Fig. 9).

The delay factor indicates that experts prefer to focus on adding older names (not yet included) or improving existing records (for instance refining distributional details), instead of including new species. This could be caused by the often unclear taxonomic 'robustness' of newly described species (new names are quickly synonymised) plus the yet unavailability of sufficient occurrence details (newly described species have a poor distributional record). It could also reflect the rather slow uptake of new taxonomic information, which could be improved when experts are served with instant information (e.g. via RSS feeds) on publications of relevant new species.

Gaps in taxonomic knowledge (species unknown to science) have been surveyed using indirect methods, with help of additional data from the European faunal inventories as recorded in the Biosis database (Suppl. material 4, Fontaine et al. 2012). The analyses show an almost saturation for well known groups, like the vertebrates or Lepidoptera (only few new species remaining to discover), whereas for many other groups, like the Acari and Collembola, the analysis reveal gaps in knowledge or a decline in description rates, even for insects with an important economic impact, like the aphids. In terms of gaps in geographic coverage Portugal and the Balkan states are the most prominent areas.

Fauna Europaea contributed to various gap assessments in ensuing projects, like PESI (Suppl. material 12). In the PESI project also special emphasis was given to the quality and completeness of data on target species, meaning species featured on lists for species conservation, quarantine control or pest management, which resulted in a separate search routine in the PESI portal (Fig. 13). In the recently started EU BON project (Hoffmann et al. 2014) further steps will be made by using Fauna Europaea as a basic tool for biodiversity assessment and for taxonomic expertise evaluation and management in Europe. In Projects like EU BON, Fauna Europaea is used as a standard reference for taxonomic Information. The gap analysis of the taxonomic information and occurrence data will be further conducted and used for improving the Fauna Europaea database in terms of quality of the information and species presence.

### Vernacular names

Common names are the most important search terms for non-professional users to retrieve biodiversity information. By means of the extended network of national Focal Points (more then 50 Focal Points in around 40 countries), the PESI project was used to harvest additional information on European species (images, literature, conservation status, etc.). Thus the PESI portal became a major meta-data repository for local biodiversity information and resources, including a resource for non-scientific names of species with 183,622 common names in 105 languages, also accessible via the Fauna Europaea portal. This information is also shared with Europeana as part of the OpenUp! project.

### Collaboration and interoperability

As a large thematic network and centre of taxonomic excellence, Fauna Europaea contributed to different projects dealing with biodiversity topics, including the development of a virtual biodiversity research community, such as the *European Network for Biodiversity Information* (ENBI), the *European Distributed Institute of Taxonomy* (EDIT), the *pan-European Species-directory Infrastructure* (PESI), the *Virtual Biodiversity Research and Access Network for Taxonomy* (ViBRANT) and lastly in *Building the European Biodiversity Observation Network* (EU BON); Europe's contribution to GEO BON, where Fauna Europaea contributes its data to the taxonomic backbone. For managing the large knowledge networks, Fauna Europaea got instrumental support from overarching bodies, like the *Consortium of European Taxonomic Facilities* (CETAF) and the *Society for the Management of Electronic Biodiversity Data* (SMEBD). Furthermore, Fauna Europaea contributes to initiatives like *LifeWatch* and *BiodiversityKnowledge* on establishing a European Community Clearing-House on biodiversity expertise, serving EC H2020 ambitions on creating an open and transparent, high-grade science-society interface for biodiversity science.

In the EuroCAT (Species2000 Europe) project Fauna Europaea contributed to the establishment of the so-called Euro-Hub of the *Catalogue of Life* (CoL), a commitment further continued in PESI (Fig. 14). Later CoL plans deviated from this model, but Fauna Europaea still supports the set up of 'proto-GSDs' to complete gaps in CoL, providing customised downloads on selected groups.

The PESI project opened opportunities for Fauna Europaea experts to apply automated tools for validation themselves (e.g. Helsdingen 2012). These quite advanced earlier validation efforts, including the NAS validation, resulting in manually assembled spreadsheets, which integrated the input of local specialists collated by National Focal Points (see: Suppl. material 13). As part of the new MfN hosting environment, new advancements are foreseen. New data annotation and validation services will be developed using the input from different Fauna Europaea validation frameworks and feed-back mechanisms (see earlier). These will also allow an easy uptake by the Fauna Europaea editors in their virtual workbenches.

In the ViBRANT project the interoperability of several core taxonomic platforms has been enhanced, guaranteeing cross-platform compatibility and shared access to important publishing infrastructures and services (Berendsohn et al. 2011). Fauna Europaea took advantage of the hosting capacity of the EDIT Platform for Cybertaxonomy to start a migration from the expiring Amsterdam services. This process was envisioned in the EDIT project, initiated in the PESI project and will be completed in the EU BON project. Fauna Europaea can profit from the virtual common access point provided by the Cybertaxonomy Platform, optimising data exchange for taxonomists as a virtual community, enabling access to other taxonomic data resources, to on-line publishing tools, to other external ontologies and supporting the generalisation of descriptive data (Fig. 11). More specifically, this could allow Fauna Europaea experts to exchange data with Scratchpads, to facilitate the preparation of data papers (see Discussion), to cross-validate their distributional details with GBIF occurrence data, to optimise literature linking, to support the addition of identification keys to their species and – in general – advance the further integration into the e-Taxonomy and e-Science domains.

**Figure 10.**
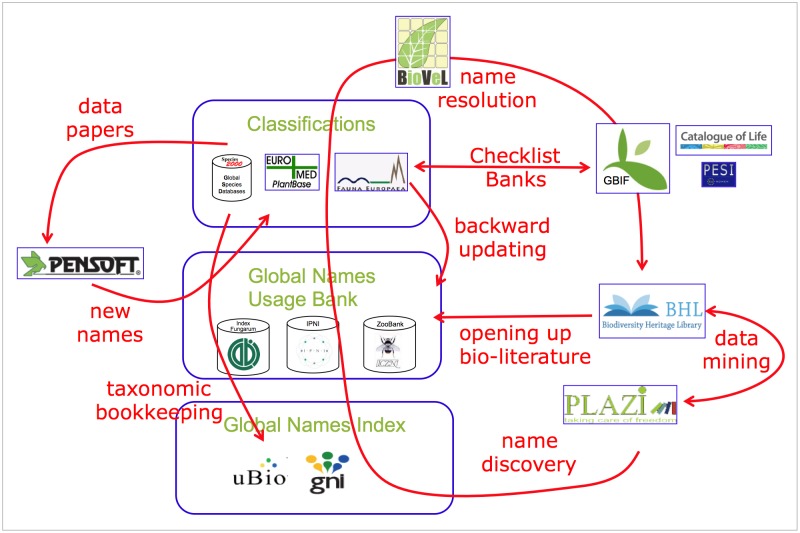
Potential work- and dataflows in a next generation linked open data names architecture.

**Figure 11.**
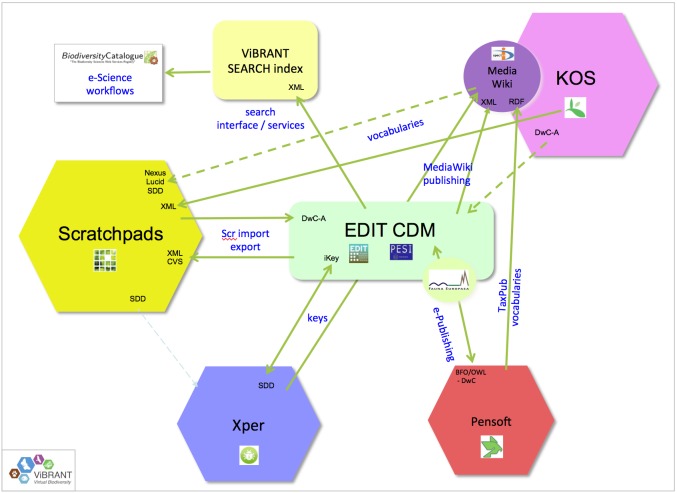
Some aspects of platform interoperability as established in the ViBRANT project. Resource: http://vbrant.eu/sites/vbrant.eu/files/ViBRANT_D4.3—Design_of_robust_services_v3.pdf

**Figure 12.**
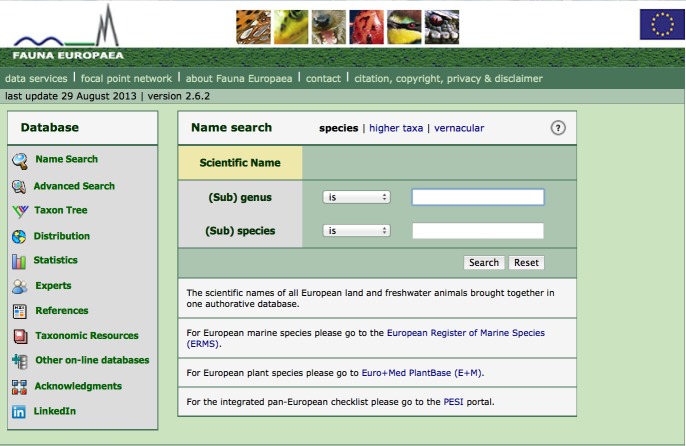
Fauna Europaea web-portal interface (faunaeur.org).

**Figure 13.**
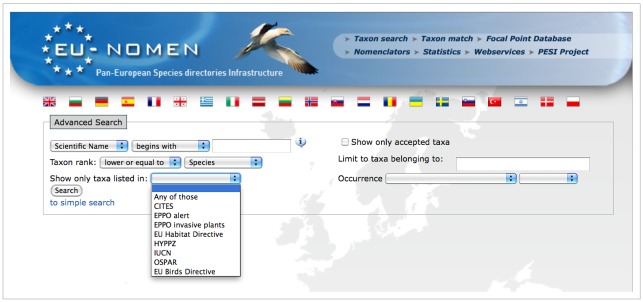
PESI target species lists search interface.

**Figure 14.**
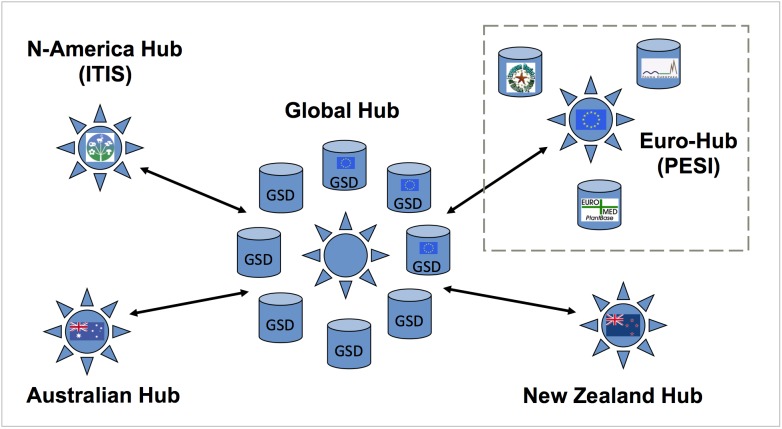
Position of PESI as Euro-Hub in the Catalogue of Life initial architecture, proceeding from the EuroCat project.

**Figure 15.**
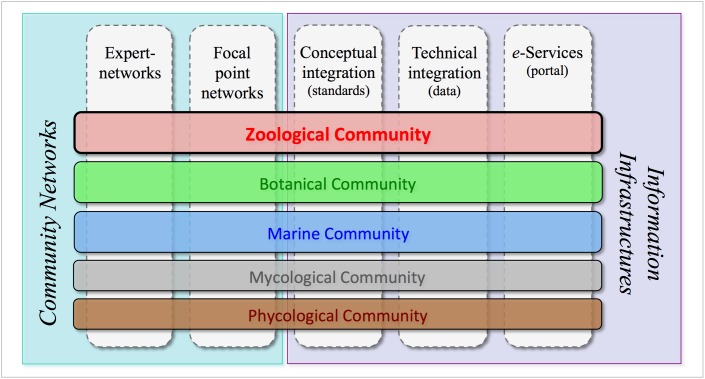
PESI infrastructural components, Fauna Europaea representing the zoological community.

At the global level Fauna Europaea contributes to the development of a next generation linked open data names architecture, also called the 'Global Names Architecture', supporting biodiversity research as a name-based science. The Fauna Europaea community especially focus on the zoological components, like the implementation of ZooBank (Pyle and Michel 2008) as a common nomenclatural reference and utilising the advanced interoperability to literature resources (for data discovery) and e-publishing tools (Fig. 10). On a longer term this new name architecture should transform Fauna Europaea (and similar projects) from a taxonomic indexing project, focused on the collation and classification of names, to a rather scientific project, dedicated to delivering high-quality taxonomic concepts and annotations; the names being managed by a common infrastructure.

### Governance and property rights

To handle the (co-)ownership of experts over their data, all Fauna Europaea specialists became members of the *Society for the Management of Electronic Biodiversity Data* (SMEBD). In addition, a Fauna Europaea executive committee was installed (to deal with Fauna Europaea related agreements) and Fauna Europaea members (experts and management staff) were elected for SMEBD council functions at a regular base. The role and relevance of SMEBD as a professional society of authors of biodiversity databases, defending the interests of its members on issues like copyright and intellectual property rights, attempting to provide a legal basis for their protection and keeping a common agenda on the sustainability of taxonomic databases, is currently under evaluation (e.g. Costello et al. 2014). In the next phase of Fauna Europaea stronger governance from internal Fauna Europaea committees is expected in place of cooperative attempts (like SMEBD). However, on defending property rights to external users, the relevance of the SMEBD organisation, experts or database custodians not protecting property rights as individuals, but lined-up as a consortium, is still of great (symbolic) value.

Recently it was decided to further license the Fauna Europaea data under CC BY SA version 4.0. We realise that most (if not all) parts of Fauna Europaea are not truly copyrightable (see Egloff et al. 2014). Also licensing doesn't prevent any (intentional) misuse or violation of copyright or property rights, but having a good license policy in place at least facilitates a proper application by the willing users and could ease the maintenance of the rights, especially on proper attribution and acknowledgement.

The future governance of the Fauna Europaea infrastructures is integrated in major programs such as LifeWatch and EU BON (e.g. as 'PESI Plus'), and embedded in collaborative approaches on profiling biodiversity informatics in EC H2020, steered by initiatives like BIH2013 (Hardisty 2013).

## Discussion

After fifteen years of delivering a steady service to the biodiversity community, Fauna Europaea is entering a next life stage, which will require a re-evaluation of the existing starting-points, including its role and functioning in the European structure of biodiversity services.

### Scope and infrastructure

The self-limitation of Fauna Europaea on delivering the best data for native species having a stable population in Europe, using strict guidelines (streamlining the various traditions in zoological taxonomy) worked out very well. Fauna Europaea didn't aim to install a universal model for zoological databasing neither to be complete. We have realised that including all exotics, incidental observations, occasional visitors, wrong identifications or obsolete names would have resulted in an undesirable situation ("everything occurring everywhere"). Besides, the effort on scrutinising such data would not only exceed the manageable capacity of the involved experts, but would also deliver a disfunctional product as a name standard and reference file.

This situation was later on balanced by the validation networks and services developed in FaEu-NAS and PESI, which also showed that most national/regional lists are over-complete. A significant amount of supposed Fauna Europaea 'gaps' actually pertain from local synonyms, infrequent visitors, incorrect identifications, invalid names, alternative taxonomies or 'fussy data' (records uncritically taken-over from literature) often relevant to be maintained at a regional level (because locally 'in use'), but inappropriate for inclusion in Fauna Europaea.

The restricted scope of Fauna Europaea will be re-evaluated as part of the EU BON project. However, establishing a comprehensive European 'super-index' still seems to be a wrong approach. A names architecture allowing national focal points to map their individual lists to a regulated (central) authoritative index, including a checklist-bank (to map other lists and classifications), a shared name-bank (presumably the Global Names Index), advanced annotation routines (allowing a selective uptake), and a cross-reference to a nomenclatural standard (presumably ZooBank), seems to be a better concept (see also Fig. 10).

Other extensions in the scope of Fauna Europaea, requested by the experts, would include a further detailing of the 'present' status for occurrences (for instance also dealing with 'Native', 'Rare', and 'Introduced') and customised taxon lists (other than alphabetically) in the classification. Further the regions should be adapted to the current political situation (e.g. the breakup of Yugoslavia) and source annotations should be insertable to distributional details.

### Pitfalls of virtual zoology

For application in the digital domain, zoological nomenclature has different advantages over botany, including a straightforward treatment of species-group names (epithet and authorship) as 'natural' nomenclatural identifiers, easing name management and resolution, independent from its generic assignment. In addition, the issue of 'potential taxon concept' (linking specimen to taxonomic concepts) is much more prominent in botany compared to zoology and complications regarding authorship changes (and derivate spellings) when introducing a new taxonomic concept, as required in botany, don’t affect zoology. However, a disadvantage of this situation is a lack of formal accounting of (past) objective synonymies in zoology. This contrast the situation in botany where the International Code of Nomenclature for algae, fungi, and plants regulates the proposal of new combinations and consequently ensures the maintenance of taxonomic history.

This feature also affects Fauna Europaea that for the search function past combinations can only be estimated as 'potential combinations' that taxonomic cross-referencing with other resources only can be really efficient at the nominal level and that in-between Fauna Europaea versions different species names sharing the same species-group name (species names which are functionally objective synonyms) have the same unique identifier.

Several solutions to this situation are intended. Firstly in the new data management environment bookkeeping of combinations is proposed, including the allocation of 'species-IDs' (in addition to the existing name-IDs). For the 'species-IDs' the PESI model on constructing a GUID could be followed (in PESI a LSID is a combination of the namespace and the Fauna Europaea Id for example: “urn:lsid:faunaeur.org:taxname:93540”). Secondly, as part of a global name infrastructure a collaborative solution could be found on organising zoological nomenclature in so-called 'nomenclators', a situation corresponding to botany.

### Publication of Fauna Europaea in a novel, 'data papers' format

It is rather demanding for specialists to continue improving data sets and for group coordinators to keep the networks together and functioning without budget or other incentives. Nevertheless, Fauna Europaea survived through the years on a low budget, and with a minimal staff, thanks to a very effective data management system, a wonderful community commitment (both from experts and institutes) and generous, in-kind support from hosting organisations.

To ensure that Fauna Europaea's contributing experts are fully acknowledged and to expand the citability of the Fauna Europaea data, it was decided to implement certain advancements in the virtual workbench development into the Fauna Europaea work routine. This was achieved during the EC-FP7 project ViBRANT, including the publication of 'data papers', meaning scholarly publications describing the respective data sectors of Fauna Europaea, using the publishing tools of the recently established Biodiversity Data Journal (Smith et al. 2013). With the support of the EC-FP7 project EUBON, this project was further developed to extend the formal descriptions of data with gap analysis for the treated taxonomic groups.

Publishing data papers in biodiversity was proposed recently as a mechanism to incentivize efforts towards discovery and publishing of biodiversity data resources (Chavan and Penev 2011). In the last two years this type of publication increased significantly in popularity among biodiversity scholars (see Erwin et al. 2011). Data papers have been published for various organismic groups, for example vascular plans of Canada (Desmet and Brouillet 2013), ants of Belgium (Brosens et al. 2013), dragonflies of the Iberian Peninsula (Torralba-Burrial and Ocharan 2013), biological traits of marine polychaetes (Faulwetter et al. 2014) to name a few.

‘Contributions on Fauna Europaea’ is the second series launched by Biodiversity Data Journal after the Checklist of British and Irish Hymenoptera (Broad 2014, Broad and Livermore 2014a, Broad and Livermore 2014b, Liston et al. 2014) and the first one that embraces thematic data papers structured in a common pattern extracted from a large data base. This novel publication model will assemble in a single issue 57 data papers on different taxonomic groups covered by Fauna Europaea project in the period 2000-2014. This is the first collection of data papers of this scale. It will formalise and effectively publish the scientific contributions of more than 400 experts building the largest European animal database. The new publication model provides a reliable mechanism for citation and bibliographic indexing of large and uniformly structured databases. We hope that this pilot by Fauna Europaea will soon be followed by other similar large-scale databases, such as the European Register of Marine Species and Euro+Med PlantBase.

In the future publishing data papers is foreseen as a standard practice after updating a Fauna Europaea data set. Apart from the value of producing scientific output and creditability for the Fauna Europaea experts, this will also allow for a more dynamic way of (additional) expert involvement, because all relevant contributions can be more easily acknowledged than what is currently the case, which will strengthen the ownership feeling (by producing 'legacy reports' for each version) and the sense of communality.

In addition, the data papers satisfy the need for the provision of more background information on Fauna Europaea data sets, including for instance the used resources and expertise and on the taxonomic decisions taken. In this context they are valuable as 'micro-publications', acting as a landing-place for small bits and pieces of information on European species. This mostly concerns unpublished new distributional records, which are often direct or indirect spin-offs from the working on Fauna Europaea.

Last but not least the Fauna Europaea data papers could also facilitate the publication of analyses based on the project, including small treatises (like master theses) or assessments carried-out by important stakeholders.

## Supplementary Material

Supplementary material 1 The Fauna Europaea (FaEu) database project – Guidelines for Group Coordinators and Taxonomic Specialists Data type: pdf
Brief description: The 'Guidelines for Group Coordinators and Taxonomic Specialists' include the standards, protocols, scope, and limits that provide the instructions for all 400 specialists contributing to the project. A first version was established in year 1 of the project. These were published in the “Guidelines for Group Coordinators and Taxonomic Specialists” as a attachment to the first annual report and improved during the project life-time.
File: oo_8432.pdf Verner Michelsen, Nicolas Billy, Yde de Jong

Supplementary material 2 Fauna Europaea geographic codes and mappings to other standards (TDWG, ISO) and projects (Euro+Med PlantBase). Data type: xls
File: oo_8516.xls Nicolas Bailly & Miguel Alonso Zarazaga

Supplementary material 3 Fauna Europaea standard agreement Data type: pdf
File: oo_8522.pdf Verner Michelsen, Per de Place Bjørn, Nicolas Bailly, Yde de Jong

Supplementary material 4 Fauna Europaea Gap Analysis - final report (including slides) Data type: pdf
File: oo_8549.pdf Benoît Fontaine

Supplementary material 5 Fauna Europaea database statistics (source file) Data type: xlsx
File: oo_8553.xlsx Yde de Jong

Supplementary material 6 Fauna Europaea homonyms Data type: xlslx
File: oo_8552.xls Yde de Jong

Supplementary material 7 Fauna Europaea species statistics for areas Data type: xlsx
File: oo_8554.xlsx Yde de Jong

Supplementary material 8 Fauna Europaea Endemics Data type: xlsx
Brief description: Figures of species and subspecies in Fauna Europaea only occuring in one area/country.
File: oo_8555.xlsx Yde de Jong

Supplementary material 9 Fauna Europaea web statistics 2013 Data type: pdf
File: oo_8638.pdf Yde de Jong

Supplementary material 10 Fauna Europaea guidelines for cross-linking Data type: pdf
File: oo_8639.pdf Yde de Jong & Günther Korb

Supplementary material 11 Fauna Europaea higher hierarchy - version 2.6.2 Data type: xlsx
File: oo_8640.xlsx Yde de Jong, Verner Michelsen, Nicolas Bailly

Supplementary material 12 PESI Gap Analysis Data type: pdf
File: oo_8642.pdf Roisin Nash (editor)

Supplementary material 13 Fauna Europaea Guidelines for NAS validation Data type: pdf
File: oo_9470.pdf Yde de Jong & Melina Verbeek

Supplementary material 14 Fauna Europaea Description of Work Data type: pdf
File: oo_9517.pdf Wouter Los

## Figures and Tables

**Table 1.** Fauna Europaea general statistics, showing taxon numbers for different taxonomic levels. Source data can be found here Suppl. material 5.

Taxa (level)	Accepted (No.)	Synonyms (No.)
All taxon names	221 701	52 882
Higher taxon names	5 838	233
(Sub)genus names	28 589	6 950
Species names	132 100	40 354
Subspecies names	14 188	5 564

**Table 2.** Fauna Europaea taxonomic groups, listing the responsible Group Coordinators, species numbers, family numbers and expert numbers for version 2.6.2. An indication of known gaps is given according to the gap analysis done after the Fauna Europaea first release (version 1.3). Source data can be found here Suppl. material 5.

Taxonomic group	Group Coordinator version 1	Group Coordinator version 2	No. of species	No. of families	No. of experts	Known gaps
Acari: Acariformes	Wojciech Magowski	Wojciech Magowski	6642	299	14	505 species
Acari: Ixodida	Jean-Louis Camicas	Jean-Louis Camicas	77	3	22	
Acari: Mesostigmata	Lars Lundqvist	Lars Lundqvist	1479	51	1	
Amblypygi & Uropygi	Henrik Enghoff	Henrik Enghoff	2	2	1	
Amphibians & Reptiles	Alain Dubois	Alain Dubois	230	30	6	
Annelida: Hirudinea	Alessandro Minelli	Alessandro Minelli	98	9	2	
Annelida: Oligochaeta (limnic)	Tarmo Timm	Tarmo Timm	268	8	1	
Annelida: Oligochaeta (terrestrial)	Emilia Rota	Emilia Rota	735	18	1	
Apterygote Insecta	Luis F. Mendes	Luis F. Mendes	273	4	2	
Araneae	Peter van Helsdingen	Peter van Helsdingen	4517	63	6	
Aves	Cees Roselaar	Cees Roselaar	809	87	1	
Bryozoa (Ectoprocta)	Jos & Gaby Massard-Geimer	Emmy Woss	25	7	1	
Cnidaria: Hydroida	Wim Vervoort	Wim Vervoort	54	27	1	
Coleoptera 1	Miguel A. Alonso-Zarazaga	Miguel A. Alonso-Zarazaga	15552	57	65	500 species
Coleoptera 2	Paolo Audisio	Paolo Audisio	12425	80	45	
Collembola	Louis Deharveng	Louis Deharveng	1941	23	10	
Crustacea	Geoffrey Boxshall	Geoffrey Boxshall	3493	127	9	
Diplura	Jean-Marc Thibaud	Jean-Marc Thibaud	278	5	4	
Diptera: Brachycera	Thomas Pape	Thomas Pape & Paul Beuk	11751	96	55	
Diptera: Nematocera	Herman de Jong	Paul Beuk & Thomas Pape	7526	30	14	700 species
Entoprocta	Claus Nielsen	Claus Nielsen	1	1	1	
Ephemeroptera	Carlo Belfiore & Alain Thomas	Carlo Belfiore & Alain Thomas	339	18	14	
Gastrotricha	Maria Balsamo	Maria Balsamo	214	5	5	
Helminths (Animal parasitics)	David Gibson	David Gibson	3986	214	19	
Hemiptera: Aphidoidea	Juan M. Nieto Nafría	Juan M. Nieto Nafría	1415	3	7	
Hemiptera: Cicadomorpha etc.	Hannelore Hoch	Hannelore Hoch	2053	23	3	
Hemiptera: Coccoidea etc.	Daniel Burckhardt	Daniel Burckhardt	1289	20	1	
Hemiptera: Heteroptera	Berend Aukema	Berend Aukema	2709	48	1	
Hymenoptera: Apocrita (excl. Ichneumonoidea)	John Noyes	Mircea-Dan Mitroiu	13211	52	19	
Hymenoptera: Symphyta + Ichneumonoidea	Kees van Achterberg	Kees van Achterberg	10717	14	7	
Lepidoptera: Moths + Butterflies	Ole Karsholt & Erik van Nieukerken & Willy De Prins	Ole Karsholt & Erik van Nieukerken	9865	86	60	
Mammalia	Wieslaw Bogdanowicz	Wieslaw Bogdanowicz	254	31	2	
Mecoptera	Rainer Willmann	Rainer Willmann	23	3	1	
Mollusca: Bivalvia	Rafael Araujo	Rafael Araujo	55	5	1	
Mollusca: Gastropoda	Ruud A. Bank	Ruud A. Bank	3337	70	1	
Myriapoda	Henrik Enghoff	Henrik Enghoff	2225	73	9	
Nematoda	Tom Bongers	Vlada Peneva	2618	96	36	
Nematomorpha	Andreas Schmidt-Rhaesa	Andreas Schmidt-Rhaesa	68	2	1	
Nemertea	Ray Gibson	Ray Gibson	12	3	1	
Neuropteroid orders	Horst & Ulrike Aspöck	Horst & Ulrike Aspöck & Agostine Letardi	397	15	3	
Odonata	Jan van Tol	Jan van Tol	131	11	8	
Opiliones	Jochen Martens	Jochen Martens	330	11	1	
Orthopteroid orders	Klaus-Gerhard Heller	Klaus-Gerhard Heller	1371	35	4	
Palpigradi, Pseudoscorpiones & Solifugae	Mark S. Harvey	Mark S. Harvey	831	19	1	
Phthiraptera	Eberhard Mey	Eberhard Mey	719	19	1	
Pisces	Nicolas Bailly	Jorg Freyhof	507	23	3	350 species
Platyhelminthes: Turbellaria	Anno Faubel	Carolina Noreña Janssen	738	34	2	
Plecoptera	Romolo Fochetti	Romolo Fochetti	426	7	2	
Porifera: Spongillidae	Renata Manconi	Ole Tendal	18	3	1	
Protura	Andrzej Szeptycki	Julia Shrubovych	177	4	1	
Psocoptera	Verner Michelsen	Verner Michelsen	234	25	3	
Rotifera	Hendrik Segers	Hendrik Segers	1288	30	7	
Scorpiones	Pierangelo Crucitti	Pierangelo Crucitti	23	4	2	
Siphonaptera	Maria Soledad Gomez Lopez	Maria Soledad Gomez Lopez	266	7	1	
Strepsiptera	Hans Pohl	Hans Pohl	30	7	1	
Tardigrada	Sandra J. McInnes	Sandra J. McInnes	428	14	5	
Thysanoptera	Richard zur Strassen	Bert Vierbergent	571	6	2	
Trichoptera	Peter Barnard	Hans Malicky	1049	24	1	
