# Supplementary material for: Fauna Europaea – all European animal species on the web
Source: Biodivers Data J. 2014 Sep 17;(2):e4034. doi: 10.3897/BDJ.2.e4034 (PMC4206781; doi:10.3897/BDJ.2.e4034)
Supplement: Supplementary material 3 — Fauna Europaea standard agreement [file biodiversity_data_journal-2-e4034-s003.pdf]

Fauna Europaea

Contracts

Standard Agreements for

Group Coordinators

and

Taxonomic Specialists

Verner Michelsen, Per de Place Bjørn, Nicolas Bailly, Yde de Jong

Version — 15 May 2001

Standard Group Co-ordinator Agreement - Fauna Europaea Project

Between

The Fauna Europaea Project Bureau – Copenhagen Office, hereinafter called 'FaEu-CO' acting on behalf of the Fauna Europaea Project

And

Name: #name#
Affiliation: #Address#

hereinafter called 'the Group Coordinator or GC'

Preamble

Fauna Europaea is an EU funded project (ref. EVR1-CT-1999-2001) with the objective to produce a public, WWW-accessible taxonomic and geographic distribution checklist (hereinafter called 'the Work') of European terrestrial and freshwater animals. The Fauna Europaea checklist will allow the scientific community and society to benefit from up-to-date high quality taxonomic and faunistic information.

This contract arranges for the coordination of the compilation of taxonomic and faunistic data (hereinafter called 'the Data') on the following group(s) of animals (estimated number of species in parentheses)

#Group# (#spestim#)

by the GC through a network of specialist Contributors, and the transfer of this Data to the FaEu-CO. It provides the legal basis for payments of EU funds administered by the FaEu-CO to the GC for coordinating the Data, and the subsequent non-exclusive transfer of the Data from the GC to the FaEu-CO, and the rights to subsequent dissemination of the Data in the Work.

1. Standards for delivery of data

1.1 The FaEu-CO delegates the organisation of the Data acquisition for specified taxonomic groups to the GC.

1.2 The GC acts on behalf of the FaEu-CO and informs any Contributor(s) about the:

- specification of the Data (a checklist with important synonymy of the species of the specified taxonomic group, accompanied by readily available distribution data for the defined geographical area).
- format options for delivery of Data or expertise
- timeframe and deadlines for Data delivery
- procedures for assessing quality standards through peer review by the GC and/or external experts.

1.3 Complete Data from a Contributor checked and accepted by the GC is subjected to final validation by the FaEU-Paris Office, i.e. the Data is subjected to a general, in part automatized, check for consistency of contents and spellings.

1.4 The GC will provide the FaEu-CO with a currently up-to-date list of names and addresses of the Contributors, and eventually a copy of the letters of acceptance of the Contributor's Data.

2. Obligations of the GC

2.1 The GC agrees to provide the Data to the standards specified in clause 1.2 above in agreement with the Contributor.

2.2 The GC agrees to inform the Contributors within 4 weeks of any requirement to change the Data they have submitted as requested upon peer review, and to agree a schedule for the submission of the revised Data not to exceed 4 weeks. The GC has editorial control of the Data in matters of disagreement with the Contributors.

2.3 The GC agrees to submit finally corrected Data within 4 weeks of any requirement to change the Data they have submitted as requested upon validation by the FaEu-Paris Office (refer to clause 1.3).

2.4 The GC will if payment to Contributors has been agreed on, arrange for the payment to Contributors within 4 weeks of acceptance of the entire validated and accepted Data.

3. Obligations of the GC as a Contributor

3.1 The Contributor agrees to provide the Data to the standards specified in clause 1.2 above.

3.2 The Contributor agrees to take due account of peer review feedback after submission and to improve or correct his/her Data if requested to do so. The time span between delivery of the Data and feedback will not exceed a period

of 4 weeks. Any correction to the Data should be implemented within a time period to be agreed and not to exceed 4 weeks.

3.3 The Contributor authorises FaEu-CO to store, compile, extract and disseminate the Data provided in the Work in the form specified in the Preamble.

3.4 The Contributor authorises the Data to be transferred from the Fauna Europaea Project Bureau to the designated Society who will take the Project forward beyond 2004 after the Work is published, along with any rights in that Data granted here.

4. Payment

4.1 The GC will receive for his/her work on managing the Contributors in his/her group and compilation of the Data of that group, a fixed amount of € 5.- per species on which the Contributor has delivered data as agreed with the FaEu-CO (from the Fauna Europaea grant given to the University of Copenhagen for this purpose), upon final delivery of the group Data. Final delivery is defined as delivery of the completed group Data and final written acceptance by the FaEu-CO.

4.2 Full payment for the Data will take place within 4 weeks after written acceptance; payment will be made to the GC by the FaEu-CO.

4.3 The GC may apply for an advance payment of up to 40% of the estimate of the total sum agreed upon at the date of commencement of the work, the remainder to be paid on final delivery of the group Data.

5. Copyright

5.1 The GC grants as Contributor a non-exclusive licence in the Data to Fauna Europaea for use in the Work only. Any further uses of the Data beyond the Work will be the subject of a separate agreement.

5.2 The GC retains as Contributor the right to use the data compiled by him/herself for any purpose, including publication and dissemination in any form.

6. Obligations of Fauna Europaea

The FaEu-CO and thereby the Fauna Europaea Project will acknowledge the Contributor and/or his/her affiliation as indicated by the Contributor on the completed Data, and to include that Data in the Work. The Fauna Europaea

Project will also ensure that the designated Society referred to in Clause 3.4 will commit to these obligations.

7. Termination

7.1 This agreement shall remain in force until either party notifies the other in writing that it wishes to discontinue it.

7.2 In the event of termination of this agreement FaEu-CO will continue to have the non-exclusive right to disseminate the Data in the Work, but the GC and the Contributor may have his or her acknowledgement and/or that of his/her affiliation removed upon request.

8. Law

8.1 This agreement and any dispute arising thereof shall be governed by the general principles of Danish law.

8.2 Any dispute between Contributor and FaEu-CO arising out of the interpretation or execution of this agreement shall be settled by mutual agreement. If the Contributor and FaEu-CO are unable to reach agreement on any question in dispute or on a mode of settlement other than arbitration, either party shall have the right to request arbitration by the court of law in Copenhagen.

8.3 The Contributor and FaEu-CO agree to be bound by any arbitration award rendered in accordance with this article as the final judgement of any such dispute.

This agreement shall come into force upon signature by both parties.

On behalf of Fauna Europaea, the FaEu-CO represented by Dr. Henrik Enghoff, Copenhagen

Henrik Enghoff ________________ (signature)

Henrik Enghoff (print name)

May 15. 2001 (date)

The Group Coordinator

________________ (signature)

________________ (print name)

________________ (date)

Standard Contributor Agreement
Fauna Europaea Project

Between

The Fauna Europaea Project Bureau – Copenhagen Office, hereinafter called 'FaEu-CO' acting on behalf of the Fauna Europaea Project

And

Name: ____________________________
Affiliation: ____________________________
____

hereinafter called 'the Contributor'

Preamble

Fauna Europaea is an EU funded project (ref. EVR1-CT-1999-2001) with the objective to produce a public, WWW-accessible taxonomic and geographic distribution checklist (hereinafter called 'the Work') of European terrestrial and freshwater animals. The Fauna Europaea checklist will allow the scientific community and society to benefit from up-to-date high quality taxonomic and faunistic information.

This contract arranges for the compilation of taxonomic and faunistic data (hereinafter called 'the Data') on the following group of animals (estimated number of species in parentheses)

--(--------------)

by the Contributor and the transfer of this Data to the FaEu-CO. It provides the legal basis for payments of EU funds administered by the FaEu-CO to the Contributor, and the subsequent non-exclusive transfer of the Data to the FaEu-CO, and the rights to subsequent dissemination of the Data in the Work.

1. Standards for delivery of data

1.1 The FaEu-CO delegates the organisation of the Data acquisition for specified taxonomic groups to 'Group Coordinators' (GC).

1.2 The GC acts on behalf of the FaEu-CO and informs any Contributor(s) about the:

- specification of the Data (a checklist with important synonymy of the species of the specified taxonomic group, accompanied by readily available distribution data for the defined geographical area).
- format options for delivery of Data or expertise

- time frame and deadlines for Data delivery
- procedures for assessing quality standards through peer-review by the GC and/or external experts.

1.3 Completed Data from a Contributor checked and accepted by the GC is subjected to final validation by the FaEU-Paris Office, i.e. the Data is subjected to a general, in part automatized, check for consistency of contents and spellings.

2. Obligations of the Contributor

2.1 The Contributor agrees to provide the Data to the standards specified in clause 1.2 above in agreement with the GC.

2.2 The Contributor agrees to take due account of peer review feedback after submission and to improve or correct his/her Data if requested to do so. The time span between delivery of the Data and feedback via the GC will not exceed a period of 4 weeks. Any correction to the Data should be implemented within a time period to be agreed with the GC and not to exceed 4 weeks.

2.3 After final delivery, the Contributor accepts that the GC may edit the Data provided to resolve or annotate any taxonomic conflicts and will inform the Contributor of this. The Contributor agrees that the GC has final editorial control.

2.4 The Contributor authorises FaEu-CO to store, compile, extract and disseminate the Data provided in the Work in the form specified in the Preamble.

2.5 The Contributor authorises the Data to be transferred from the Fauna Europaea Project Bureau to the designated Society who will take the Project forward beyond 2004 after the Work is published, along with any rights in that Data granted here.

3. Payment

3.1 The Contributor will receive for his/her work on compiling the Data as specified in clause 1 above, the fixed sum of -------- per species as agreed with the GC upon final delivery of the Data. Final delivery is defined as delivery of the completed Data, followed by peer review, acceptable completion of any requested revision, and final written acceptance by the GC.

3.2 Full payment for the Data will take place within 4 weeks after written acceptance from the GC; payment to be made to the Contributor by the GC or by FaEu-CO as agreed.

3.3 The Contributor may apply for an advance payment of up to 40% of the estimate of the total sum agreed upon at the date of commencement of the work, the remainder to be paid on final delivery of the Data. The advance payment is returnable if the Data is not delivered or if it is not in a form acceptable to the GC. If the Data is delivered in part then the GC and Contributor may agree a revised fee for this work, and the advance will be treated as part of this revised payment, and some part of it may be returnable.

4. Copyright

4.1 The Contributor grants a non-exclusive licence in the Data to Fauna Europaea for use in the Work only. Any further uses of the Data beyond the Work will be the subject of a separate agreement.

4.2 The Contributor retains the right to use the Data compiled by him/herself for any purpose, including publication and dissemination in any form.

5. Obligations of Fauna Europaea

The FaEu-CO and thereby the Fauna Europaea Project will acknowledge the Contributor and/or his/her affiliation as indicated by the Contributor on the completed Data, and to include that Data in the Work. The Fauna Europaea Project will also ensure that the designated Society referred to in Clause 2.5 will commit to these obligations.

6. Termination

6.1 This agreement shall remain in force until either party notifies the other in writing that it wishes to discontinue it.

6.2 In the event of termination of this agreement FaEu-CO will continue to have the non-exclusive right to disseminate the Data in the Work, but the Contributor may have his or her acknowledgement and/or that of his/her affiliation removed upon request.

7. Law

7.1 This agreement and any dispute arising thereof shall be governed by the general principles of Danish law.

7.2 Any dispute between Contributor and FaEu-CO arising out of the interpretation or execution of this agreement shall be settled by mutual agreement. If the Contributor and FaEu-CO are unable to reach agreement on any question in dispute or on a mode of settlement other than arbitration, either party shall have the right to request arbitration by the court of law in Copenhagen.

7.3 The Contributor and FaEu-CO agree to be bound by any arbitration award rendered in accordance with this article as the final judgement of any such dispute.

This agreement shall come into force upon signature by both parties.

On behalf of Fauna Europaea, the FaEu-CO represented by Dr. Henrik Enghoff, Copenhagen

The Contributor:

________________ (signature) ________________ (signature)

________________ (print name) ________________ (print name)

[illegible] [illegible]
