# Supplementary material for: Fauna Europaea – all European animal species on the web
Source: Biodivers Data J. 2014 Sep 17;(2):e4034. doi: 10.3897/BDJ.2.e4034 (PMC4206781; doi:10.3897/BDJ.2.e4034)
Supplement: Supplementary material 4 — Fauna Europaea Gap Analysis - final report (including slides) [file biodiversity_data_journal-2-e4034-s004.pdf]

Fauna Europaea Gap Analysis

Final Report

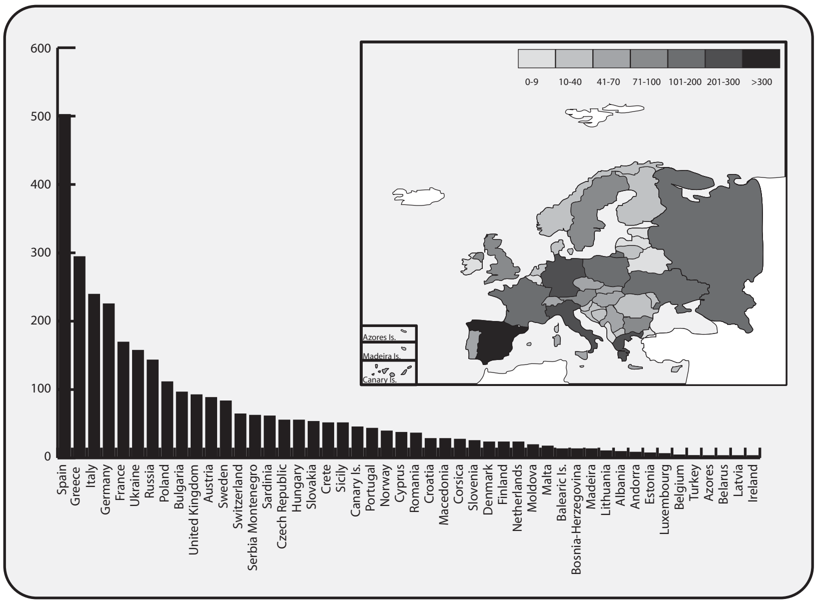

Benoît FONTAINE

January 2005

WP12: Workpackage on Gap Analysis - Part of Deliverable 14

FAUNA EUROPAEA

CONTENT

Content 1
Objectives 2
Methods 2
Results 6
Discussion 11
Conclusion 13
Perspectives 13
Appendix I: figures, 1758-2003 14
Appendix II: figures, 1998-2002 39
Appendix III: raw data, 1998-2002 59
Appendix IV: paris meeting presentation 65

OBJECTIVES

In the framework of the Fauna Europaea Project, we analyzed the growth of the taxonomic inventory of the non-marine European fauna. Our objective was to answer the following questions:

- How completely do we know the fauna of Europe?
- Where are the gaps?
- Who fills the gaps?

This report presents the results of the Gap Analysis. Analyses of the Biosis database were completed in 2003, as well as the analyse of the data available at this date in the Fauna Europaea database (27,000 species). These first results were presented in the preliminary report (FONTAINE, B. 2003. *Fauna Europaea Gap Analysis - Preliminary report*. October 2003. 11 + 28 pp.). The analyses on the 10% random subsample, the top 10 Fauna Europaea species and an updating of the Fauna Europaea database analysis were presented at the Fauna Europaea meeting in Paris (25-27 September 2004). At that point, some data were still missing in the the Fauna Europaea database (117,311 species in the database). This reports summarizes all the analyses previously presented, as well as an update of the analysis on the Fauna Europaea database as of December 2004.

METHODS

TOTAL FAUNA

In order to measure the global growth of the taxonomic inventory since 1758, we have used species lists provided by Fauna Europaea Group Coordinators. These lists include only valid species, i.e. all synonyms have been excluded. To date (January 2005), the available dataset comprises 125,854 species (Table 5, Appendix I).

For selected taxa, as well as for the whole dataset, the cumulative number of known species was plotted vs. the year of description.

RECENT DESCRIPTIONS

The analysis of the structure of the current growth (years 1998-2002) is based on a dataset extracted from the Zoological Record and purchased from Biosis. Several analysis were performed, with respect to the geographical origin of the new species and the country address of the first descriptor of new species.

The dataset includes all non-marine animal species described between 1998 and 2002 included (five years), cited in the Zoological Record, for the countries and islands covered by Fauna Europaea (see the area covered in the Fauna Europaea Guidelines at www.faunaeur.org/PUBLIC/DOCUMENTS/DOCUMENTS1.html). For each species, the following information were provided: name, taxonomical hierarchy, author, year of description, country of type locality, author's country of affiliation and full title of the description paper. Fossil species and Protozoa were excluded. It has to be stressed that the datasets initially provided by Biosis were "polluted" by unwanted references (e.g. fossil

species, duplications, confusion between country of occurence and country of type locality), and it was not before two months of e-mail exchanges with Biosis that an acceptable dataset was made available.

Table 1: Summary of databases characteristics for the Gap Analysis project.

Database	Period	Database source	No species	Figures
Total fauna	1758-2003	Fauna Europaea Group Coordinators	125,854	Appendix I
Recent descriptions	1998-2002	Zoological Record	3,149	Appendix II

Treatment of new species from Russia and Turkey

Biosis did not make a distinction between the European and Asiatic parts of Russia and Turkey. Species described from Turkey and Russia had then to be screened, for large extent of these countries are outside the Fauna Europaea coverage (Asian Turkey, Russia east of Ural and Caucasus region): the species described from Asian parts had to be removed from the dataset. In most cases, the title of the paper describing the species, the locality given in the Zoological Record database or the paper abstract, if available, allowed to decide whether to include or exclude the species. In the remaining cases, the species name could sometimes be used to decide: species with names such as *magadanensis* (after the city of Magadan in the Russian Far-East), *tauricus* (after the classical province of Taurid in Asia Minor), *anatolensis*, *anatolicus*, *antakyaensis*, *burdurensis*, *sertavulensis*, *surucicus*, *ussuriensis*, *vanensis* or *yakutiensis* were excluded. Once these species are excluded, the dataset provided by Biosis comprises 3,149 new species.

In the analysis, no distinction was made between “mainland” Russia and the Kaliningrad enclave.

Number of descriptors:

Descriptors where characterized by their family name, first (given) name and country of affiliation: if a name and first name of descriptor appeared twice with two different affiliation country, it was counted twice (once with each country).

When species were described by more than one descriptor, only the first one was used in the analysis of the geographical origin of descriptors, because the Biosis dataset provided only one affiliation address per paper. For example, no distinction was made between a description by Assing, V., another by Assing, V. & Maruyama, M. and a third by Assing, V. & Monguzzi, R.: these were all treated as if described by Assing, V., and the descriptor country did not take into account the countries of the second descriptors. It should be noted that 1,198 species (out of 3,149, i.e. 38%) were described by more than one author, so the results given below are an under-estimate of the true number of co-authors, and some descriptor countries might be under-represented.

Surface, population and GDP

For each country, surface, population and Gross Domestic Product (GDP) data were taken from the CIA world factbook (www.cia.gov/cia/publications/factbook/index.html). Islands surfaces were taken from the Times Atlas. For Russia and Turkey, figures of population and GDP for the whole country were used. However, we did not use the total surface of Russia or Turkey, but the surface of European Turkey and Russia west of Ural.

For all European countries, following data were plotted:

- Number of descriptions per geographical unit (country or island)
- Number of descriptions per country and island divided by country or island surface
- Number of descriptions per country of descriptor
- Number of descriptors per country
- Number of descriptors per country divided by country population
- Number of descriptors per country divided by GDP
- Percentage of new species described by taxonomists working in the country of type locality
- For each affiliation country, percentage of new species described from countries different from affiliation country

Databases accuracy

The lists provided by Group Coordinators are supposed to be the most accurate available species lists for Europe, reflecting the most up to date state of the art. In order to control the accuracy of both databases (Fauna Europaea and Zoological Record), the numbers of species were compared between both databases for some large taxa. Results are summarized in Table 2. There are differences between databases, either due to the fact that some species have not been included yet in the Zoological Record, because some species included in Zoological Record were not known by Group Coordinators, or because some species have been synonymized and removed from the Fauna Europaea list. However, these differences were considered negligeable for our purpose because of their low percentage.

Table 2: Numbers of species described between 1998 and 2002, for selected taxa, from both databases.

Taxon	Fauna Europaea Database	Zoological Record Database
Turbellaria	5	7
Pseudoscorpiones	28	23
Aphididae	17	16
Ichneumonidae	348	331
Diptera	427	424
Trichoptera	40	43

Top 10 Fauna Europaea species

Group coordinators where requested to highlight some species recently described that had something "significant", to send the message that there are still unexpected and significant discoveries to be made in Europe. "Significance" may be in terms of economical, zoological or ecological importance. We selected ten species out of those suggested by Group Coordinators, to get a “qualitative” picture of what is currently discovered in Europe. These are briefly presented in the powerpoint presentation, together with the reason why they were selected (Appendix IV, slides 17-26).

RANDOM SAMPLE OF RECENT DESCRIPTIONS

A subsample of 10% of the recently described species was randomly extracted from the Biosis dataset: each species was allocated a number, and we had a computer choosing numbers randomly. The primary litterature containing the original descriptions of all 338 selected species were searched for, but 310 only were found: 4 were found on the web, 282 were found in the various libraries of the Muséum national d'Histoire naturelle (Paris), 2 were provided by researchers, out of their own libraries. Fifty descriptions could not be found by these ways, so we requested them from the authors via e-mail, and got 22. We could not access 28 descriptions.

These original descriptions allowed us to check various parameters:

- Exact type localities were plotted on a map;
- Whether descriptions were based on morphology only, or as well on more recent technologies such as sonograms, karyotypes or molecular techniques. When a description mentionned new techniques, it was allocated to the category "new techniques", even if it was only peripheral: in one description, it was mentionned that the karyotype lead to the discovery of a new species, but the description itself was only morphological. Still, this description was put in the "new techniques" category;
- Time elapsed between collection of new species and formal naming;
- Weight of the amateur community: we asked the Group Coordinators to assign the first authors of these papers a category, i.e. Professional taxonomist (being paid to do taxonomy), Amateur (unpaid to do taxonomy: a professional ecologist describing species occasionnally was considered as amateur), Retired professional or Student.

RESULTS

TOTAL FAUNA

Slide 8 (Appendix IV) presents the number of known species in European countries as of September 2004 (117,311 species. It is now 125,854 species, but that doesn't change the global results). The large western countries are the richest. However, this richness is not only related to the size of the country, as shown by slide 9 (number of species divided par log(surface)): it reflects the biogeographical heterogeneity of the country (France, Italy) and/or the level of taxonomic knowledge and activity in the country (Spain, Germany).

Graphs plotting the number of known species against the year of description are presented in Appendix I. For the whole dataset (125,854 species), the curve shows three sections of even growth, from Linnaeus to 1830, from 1830 to 1950, and then since 1950. Each section is even, but steeper than the previous one (Figure 3): as many species are described each year now than thirty years ago, but more than one century ago, and even more than two centuries ago. There is a slight apparent levelling since 2002, due to the fact that all data from 2002 to 2004 are not available yet (Figure 1). A t-test was performed to compare the number of descriptions in the decade 1993-2002 and in the decade 1983-1992. From this test, it appears that there is no significant difference in the number of yearly descriptions between those decades. At a larger scale, there is no indication that the curve is reaching an asymptote, indicating that the number of species descriptions per year in Europe does not decrease.

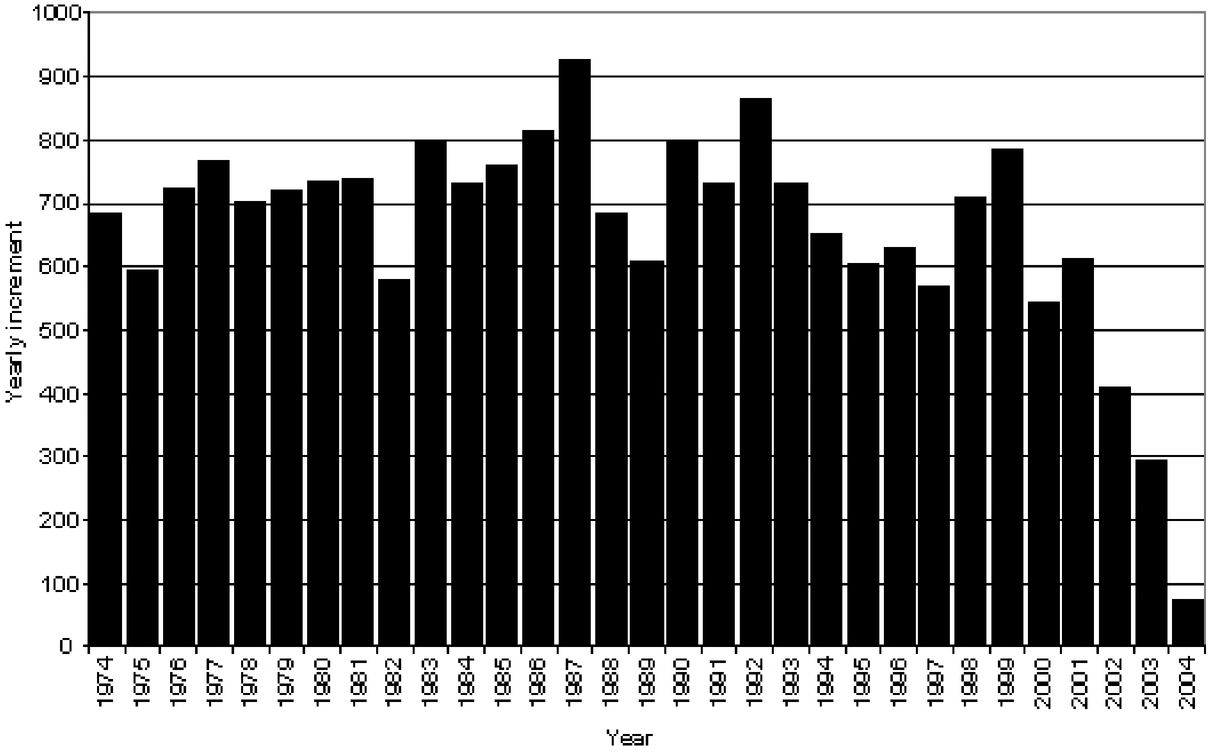

Figure 1: Yearly increment of the number of species in the period 1974-2004, as given by the available dataset.

The evenly growing curve seen on Figure 3 is the sum of various situations, which could be summarized as follow:

- Taxa for which the number of described species does not increase anymore, the asymptote having been reached many decades ago (e.g. Odonata [Figure 21], Aves [Figure 36], all terrestrial vertebrates [Figure 41]);
- Taxa where we are approaching an asymptote (however, this might be only an artefact - e.g. Plathyhelminthes Turbellaria [Figure 9], Neuropteroid orders [Figure 27]);
- Taxa for which the number of described species has been evenly increasing for more than 100 years (e.g. Pisces [Figure 35], Insecta as a whole [Figure 38], Orthopteroid orders [Figure 23]);
- Taxa which remained totally unknown or poorly known for decades, but have been experimenting a rapid increase of the number of species since then (e.g. Annelida [Figure 11], Acari [Figure 15], Apterygote Insecta [Figure 19], Plecoptera [Figure 22]).

When the countries where the species are present are taken into account, it appears clearly that the inventory is close to completion in some countries, whereas the curve is evenly increasing in some others (Slide 33, Appendix IV). This is especially obvious for selected taxa: for instance, Coleoptera and Gastropoda are more or less completely inventoried in Netherlands, Sweden, United Kingdom and Germany, but not in France, Spain, Italy or Greece (Slides 31-32, Appendix IV). The impact of large-scale government funded programs such as Fauna Iberica is visible on these graphs. The case of Greece is remarkable: it is a country with a high potential (Mediterranean biome with many islands, and mountaneous areas with Balkanic affinities), but under explored; after a slow start, the inventory in Greece is steadily increasing and will outnumber the inventory of larger country which have been well explored in the past such as Germany.

Effect of wars on species descriptions

Worth being mentionned, the rate of species descriptions has been slowed down during World Wars I and II. Considering that there is a lap between the actual description work and its publication, we have considered that species described during WWI and II have been published in 1915-1919 and 1940-1945 (Figure 2). From this result, it appears that beside many more serious consequences, war has an impact on taxonomic activity.

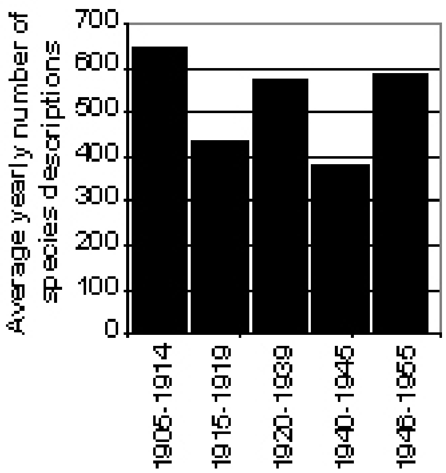

Figure 2: Average number of species descriptions per year before, during and after WWI and II, as given by the available dataset.

Recent descriptions

Once the species from Asian Turkey and Russia east of Ural are excluded, the dataset provided by Biosis comprises 3,149 new species. Figures are presented in Appendix II.

The taxonomic composition of new species is described Figure 42. Out of 3,149 new species, 2,358 (74.9%) are Hexapoda, and 766 (24.3%) are Hymenoptera. Diptera, Lepidoptera, Hymenoptera, Coleoptera and Hemiptera taken together make up 64.7% of all the descriptions. After insects, arachnids are the second most important taxon in term of descriptions in Europe. Between 1998 and 2001 (year 2002 was excluded, assuming that some of the data for that year were still missing in Biosis database), the average yearly increment has been 673.5 new species.

Not surprisingly, mainland Spain had the highest number of new species descriptions (502 new species with type locality in Spain, i.e. 16% of new species in Europe for that period), followed by two Mediterranean countries, Greece (without Crete) and Italy (without Sicily and Sardinia). More unexpectedly, Germany is the fourth country. Then Ukraine, with Crimea, and Russia, the largest country, follow (Figure 43). Iceland is the only European country where no new species was described in 1998-2002. To account for the size of the country, the same data divided by country or island area were plotted (Figure 44). Malta, with 17 n. sp. (53.8 x 10^{-3} n.sp./km²), ranks first, followed by Andorra (8 n. sp., 17.1 x 10^{-3} n.sp./km²) and by seven Macaronesian and Mediterranean islands. Greece is the first continental geographic unit (with 294 n. sp.), but all Greek islands except Crete were treated together with mainland Greece.

Out of the 3,149 new species, 3,082 (97.8%) were described by taxonomists working in Europe, USA being the non-European country most contributing to the description of European species, with 24 descriptions. Taxonomists working in Germany described the highest number of European new species in 1998-2002 (698 n. sp.), followed by taxonomists working in Italy (410 n. sp.), Spain (251 n. sp.) and Russia (214 n. sp.) (Figure 45). Taxonomists from these four countries make up 50.0% of the total number of descriptions in the study period.

Altogether, 855 first authors were involved in the descriptions of 3,149 new species in 1998-2002 (Figure 46). Of these, 55% were working in five countries only (Germany, Italy, Spain, Russia and France). No scientist working in Albania, Andorra, Bosnia-Herzegovina, Cyprus, Iceland, Luxembourg and Moldova described new species in the study period, at least as first author. When the country population (Figure 47) or GDP (Figure 48) is taken into account, Central/Eastern Europe has the highest contribution to species description: Czech Republic, Slovenia, Austria and Bulgaria are the most productive countries in term of number of descriptors per inhabitant, and Serbia/Montenegro, Bulgaria, Czech Republic and Slovenia are the most productive countries in term of number of descriptors/country GDP.

Figure 49 and Figure 50 show the inbreeding/outbreeding of national expertise i.e. wether taxonomists describe species from their affiliation country or from abroad. “Inbreeding” is globally more important in northern Europe (Scandinavia excepted), and less in south-eastern Europe (Figure 49). Among the most species-rich countries, Greece is the country were most species are described by foreign taxonomists (5.4% of Greek species are described by Greek taxonomists). At the other end, most new species described from Germany are described by German taxonomists (79.7% - see Table 9). It should be noted that depending on the country, Mediterranean and Macaronesian islands are treated differently: species from Sardinia and Sicily are mostly described by Italians, whereas species from the Canary Islands are mostly described by non Spanish taxonomists, and no species from Madeira and the Azores was

described by a Portuguese taxonomist (Table 3). When one considers the percentage of descriptions from countries different from the affiliation country (Figure 50 and Table 10), it appears clearly that outbreeding is most important in northern and northwestern Europe, i.e. most species described by taxonomists working in northern and northwestern Europe were not discovered in affiliation country.

Table 3: Percentage of species from Mediterranean and Macaronesian islands described by taxonomists working in the mainland country, 1998-2002. Data are given for mainland countries for comparison. Data extracted from Biosis dataset.

Island or Country	% of new species described by nationals
Sardinia	86.9
Sicily	64.7
Mainland Italy	56.5
Corsica	48.1
Mainland France	40.8
Balearic Islands	46.2
Canary Islands	15.9
Mainland Spain	44.3
Crete	13.7
Greece excl. Crete	5.4
Azores	0
Madeira	0
Mainland Portugal	27.9

When the exact type localities of 310 recently described species are plotted on a map (Slide 30, Appendix IV), it appears clearly that the Alpine and Balkanic chains are important sources of new species, as well as the whole of Spain and Germany. Mediterranean islands also harbour many new species. Some “hotspots” are highlighted, but they reflect centers of taxonomic activity rather than biologically rich areas: Oxford and Madrid areas, or Borok area, in western Russia, from where a single researcher named 22 nematode species between 1998 and 2003. When taxa are taken into account, it appears that some areas are less diverse than others in terms of new species descriptions: in Netherlands or Scandinavia, most new species are Hymenoptera, whereas in Poland, Slovakia or Moldavia, they mostly belong to Acari. On the other hand, new species from southern Europe belong to all taxa.

Amateurs vs. professional

As is shown on slide 37 (Appendix IV), only 55% of the descriptors of recent species are professional, but amateurs describe proportionnaly more than professionals (54% of new species): more than half of the species are described by people who are not paid for that.

Time between collection and descriptions

Most descriptions are from material recently collected (Slide 39, Appendix IV): 25% of descriptions are made within three years after collecting, 50% after six years, and 75% after 13 years. Only nine species (out of 310) were described more than 50 years after collecting. When this analysis takes into account professionnal status of descriptors, the graphs are similar for amateur and professionnal, i.e. professionnals don’t wait longer to describe species.

New technologies

Only 2% of the 310 randomly selected descriptions had a "new technologies" component, i.e. 98% were based only on morphology (Slide 40, Appendix IV). However, this probably underestimates the weight of new technologies in modern taxonomy, as these technologies can lead to the discovery of new species that are later formally described with morphology only (in that case, these technologies may not be mentionned in the description). New technologies (mainly genetics) play also a role in the lumping of species, an issue which is not addressed in this study.

Access to primary litterature

A side result of this study was the difficulty to access to primary litterature (Slide 41, Appendix IV). Only 84% of the descriptions of recently described European species where found in the various libraries of the Muséum national d'Histoire naturelle in Paris, one of the largest in Europe, and only 1% on the web. That means that 15% of the descriptions are not easily accessible in this Museum. However, a quick internet search revealed that The Natural History Museum (London) seems to have most of the journals which are missing in Paris.

DISCUSSION

One of the main results of this analysis is that globally, more species are being described in Europe today than one century ago, which means that there are still many discoveries to be done in Europe, i.e. 600 or 700 per year on average. However, this curve is the result of a balance between two facts:

- it is more and more difficult to discover new species, as more and more are being known;
- collecting and/or discriminating techniques are getting more efficient.

It is surprising that these facts are balanced, i.e. the curve slope is constant: it could be that the slope either decreases or increases, depending on which factor is heaviest. In fact, all available species taken together, the new prospecting/discriminating techniques exactly counterbalanced the increasing difficulty to find new species since 1830. When taxa are considered separately, this balance works differently: in some taxa, it is more and more difficult to find new ones even with new discriminating techniques (Lepidoptera Rhopalocera, [Slide 14, Appendix IV] for instance), whereas in some others new techniques of discrimination or collecting (Rotifera [Figure 5], Annelida [Figure 11] for instance) have accelerated the number of descriptions.

However, it should be stressed that another important factor impacts these curves, namely the availability of taxonomic expertise for a given group at a given time, i.e. presence or absence of taxonomists studying it. Some curves show either temporary levelling(s) of the increase of described species (e.g. Mollusca [Figure 10], Neuropteroid orders [Figure 27]), Pseudoscorpiones [Figure 13]), or leaps on particular years (e.g. Tardigrada [Figure 12]; Turbellaria [Figure 9]; Pseudoscorpiones [Figure 13] - Table 4). These temporary levellings and leaps must be linked to the availability of taxonomic expertise and interest at a given time, together with the availability of new discriminating and/or prospecting methods.

The results are impacted by the works of single authors. For the 1998-2002 period, the importance of Germany in term of number of described species and number of species described by German authors, as well as the representation of Hymenoptera in the total set of new species is partly due to a single monograph (Schwenke, W. 1999. Revision der europaischen Mesochorinae (Hymenoptera, Ichneumonoidea, Ichneumonidae). *Spixiana Supplement* 26: 1-124). This single work adds 221 new Hymenoptera species to the total (7% of all new species), including 77 from Germany. It accounts for 34% of new species from Germany, and for 31% of the species described by German authors. If this single work is removed, Germany goes from the fourth to the sixth rank (between Ukraine and Russia) for the number of described species per country, but still ranks first for the number of species described per country of descriptor.

However, there is no other work of this importance in the study period (1998-2002): the next largest monographs added only 37 and 26 new species (Hymenoptera) to the total. Out of 3,149 descriptions, 1,120 (35.5%) were from papers describing only one species.

Table 4: Selected leaps in the descriptions of new species in Europe, taken from the available dataset.

Taxon	Year	Number of descriptions	Number of descriptors	% described by one descriptor
Rotifera	1947	42	2	97.6
Turbellaria	1924	43	8	60.5
Pseudoscorpiones	1938-39	102	3	93.1
Aphididae	1950	89	8	83.1
Ichneumonidae	1829	633	2	99.8
	1999	230	7	89.1
Asilidae	1820	70	2	78.6

Considering that there are still thousands of species to be described in Europe, the main limiting factor preventing the species description rate to speed up is the global availability of taxonomists. If taxonomists were more numerous in the groups with high potential for new discoveries, no doubt that there would be more species described each year.

Not unexpectedly, Mediterranean countries and large countries had the highest numbers of descriptions in the study period (Figure 43). Main exceptions, Albania and Bosnia-Herzegovina (respectively 9 and 13 n. sp.), and to a lesser extent, Slovenia, Macedonia and Croatia (respectively 25, 28 and 28 n. sp.) had a very low number of species descriptions, especially compared to neighbouring Greece (294 n. sp.). When the number of new species per square kilometer is considered, Albania, Bosnia-Herzegovina and Croatia are still very low compared to their potential richness, but Slovenia and Macedonia are above Austria, Spain or Italy (Figure 44). This situation is most probably due to the political unrest in the Balkans in the last years, where field work by non-nationals has been almost impossible and taxonomical research by nationals has not been a priority.

The fact that Spain is the first country in terms of new species descriptions highlights the impact of large-scale government funded programs (Fauna Iberica) on taxonomic activity. Even with the important weight of amateur community, there is no doubt that large scale funding boosts the activity at a country level.

Another noticeable geographical gap is Portugal, way below Spain in term of number of raw descriptions (43 vs. 502), but also for descriptions.km^{-2}. At the other end of Europe, Belarus had only three new species in 1998-2003, much less than its neighbours, Poland (111 n. sp.), Russia (131 n. sp.) and Ukraine (157 n. sp.), but not far from Lithuania (10 n. sp.), Latvia (3 n. sp.) and Estonia (7 n. sp.). When number of new species is divided by country area, Belarus ranks last in Europe, still far from Poland or Ukraine, but close to Russia (second to last country) and Latvia. Lithuania and Estonia are between Belarus/Russia/Latvia and Poland/Ukraine. This reflects ecological heterogeneity and the size of the reservoir of new species.

CONCLUSION

According to this dataset, the inventory of very few European taxa is close to completion. Birds, of course, can be considered as completely inventoried in Europe, even when new technologies are used, and there will be few additional freshwater Bivalvia to the European fauna. The rate of descriptions is slowing down for Turbellaria, Psocoptera, Thysanoptera, Aphididae and Neuroptera, suggesting that there will be fewer and fewer additional species. However, it is possible that this slowing down is due to a lack of taxonomic expertise, and as it has often been the case, it is possible that new prospecting/discriminating techniques, or availability of taxonomic expertise allow the description of many new species in these taxa. For all the other taxa, our data show no indication of slowing down of the description rate, suggesting that many new species are still to be described.

As it has been mentioned above, the main and most obvious geographical gaps are the Balkans and Portugal.

Taxonomists working in Germany, Italy, Spain, Russia and France contribute to 55% of descriptions, and 55% of descriptors work in these countries. However, when population or GDP are taken into account (as a measure of human and economic resources that a country may contribute to taxonomy), Czech Republic, Slovenia and Austria (population) and Serbia/Montenegro, Bulgaria and Czech Republic (GDP) rank first.

PERSPECTIVES

This work will lead to the publication of scientific papers that will aim to raise the awareness on the situation of taxonomy in Europe. As was proposed during the Paris meeting, we suggest that a paper will be authored by the Paris team (Benoît Fontaine, Philippe Bouchet, Daniel Goujet and Nicolas Bailly), the Fauna Europaea bureau and all the group coordinators.

APPENDIX I: FIGURES, 1758-2003

Taxon	Number of species
Porifera-Spongillidae	18
Cnidaria	53
Rotifera Monogononta	878
Rotifera-Bdelloidea	301
Animal Paras Helminths	3782
Gastrotricha	205
Nematoda	2780
Nematomorpha	56
Platyhelminthes-Turbel	633
Nemertea	12
Entoprocta-Bryozoa	20
Mollusca	3125
Annelida	1101
Tardigrada	426
Arachnida Opiliones	330
Arachnida Scorpiones	23
Arachnida Palpigradi-Pseudoscorpiones-Solifugae	761
Arachnida Araneae	4045
Arachnida Acari	7133
Crustacea	3424
Myriapoda	2190
Collembola	1940
Diplura	277
Protura	173
Insecta - Apterygote	273
Insecta - Ephemeroptera	339
Insecta - Odonata	131
Insecta - Phthiraptera	718
Insecta - Plecoptera	426
Insecta - Orthopteroid orders	1300
Insecta - Psocoptera	234
Insecta - Thysanoptera	571
Insecta - Hemiptera	5355
Insecta - Neuropteroid Orders	378
Insecta - Coleoptera	27331
Insecta - Hymenoptera	23659
Insecta - Trichoptera	1049
Insecta - Lepidoptera	9511
Insecta - Mecoptera	23
Insecta - Siphonaptera	266
Insecta - Diptera	19049
Insecta - Strepsiptera	30
Amphibia-Reptilia	230
Pisces	507
Aves	534
Mammalia	254

Table 5: Number of species (without subspecies) for each taxon as of January 2005.

Total: 125,854 species.

The following figures represent the number of described European species for selected taxa. The sources of these data are lists provided by Fauna Europaea Group Coordinators, a list of which is given above (Table 5). For each graph, the X-axis represents the year of description, the Y-axis represents the cumulative number of valid species (synonyms are not included).

We have represented most available taxa above order level, and some below order level, when the taxa is remarkably numerous (more than 1000 species) or particularly demonstrative.

Figure 3: All species (as of January 2005)

Figure 4: Cnidaria

Figure 5: Rotifera

Figure 6: Gastrotricha

Figure 7: Nematoda

Figure 8: Nematomorpha

Figure 9: Platyhelminthes Turbellaria

Figure 10: Mollusca

Figure 11: Annelida

Figure 12: Tardigrada

Figure 13: Palpigradi, Solifugae, Pseudoscorpiones

Figure 14: Aranea

Figure 15: Acari

Figure 16: Crustacea

Figure 17: Myriapoda

Figure 18: Collembola

Figure 19: Apterygote Insecta

Figure 20: Ephemeroptera

Figure 21: Odonata

Figure 22: Plecoptera

Figure 23: Orthopteroid orders

Figure 24: Psocoptera

Figure 25: Thysanoptera

Figure 26: Hemiptera

Figure 27: Neuropteroid orders

Figure 28: Coleoptera

Figure 29: Hymenoptera

Figure 30: Trichoptera

Figure 31: Lepidoptera

Figure 32: Siphonaptera

Figure 33: Diptera

Figure 34: Amphibia-Reptilia

Figure 35: Pisces

Figure 36: Aves

Figure 37: Mammalia

Figure 38: All Insecta

Figure 39: Arthropoda excl. Insecta

Figure 40: Other invertebrates

Figure 41: Terrestrial Vertebrates

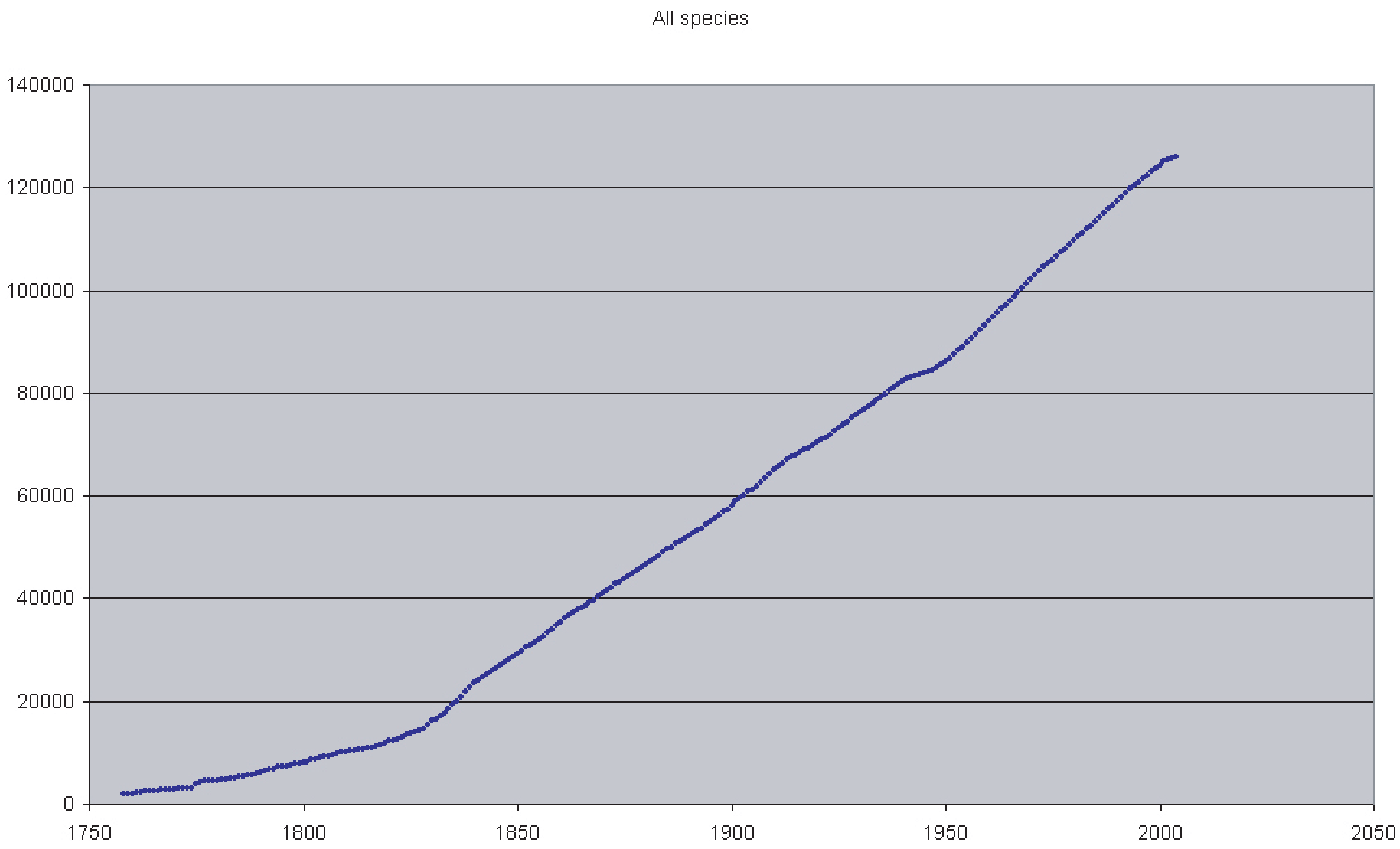
All species
140000
120000
100000
80000
60000
40000
20000
0
1750
1800
1850
1900
1950
2000
2050

Figure 3

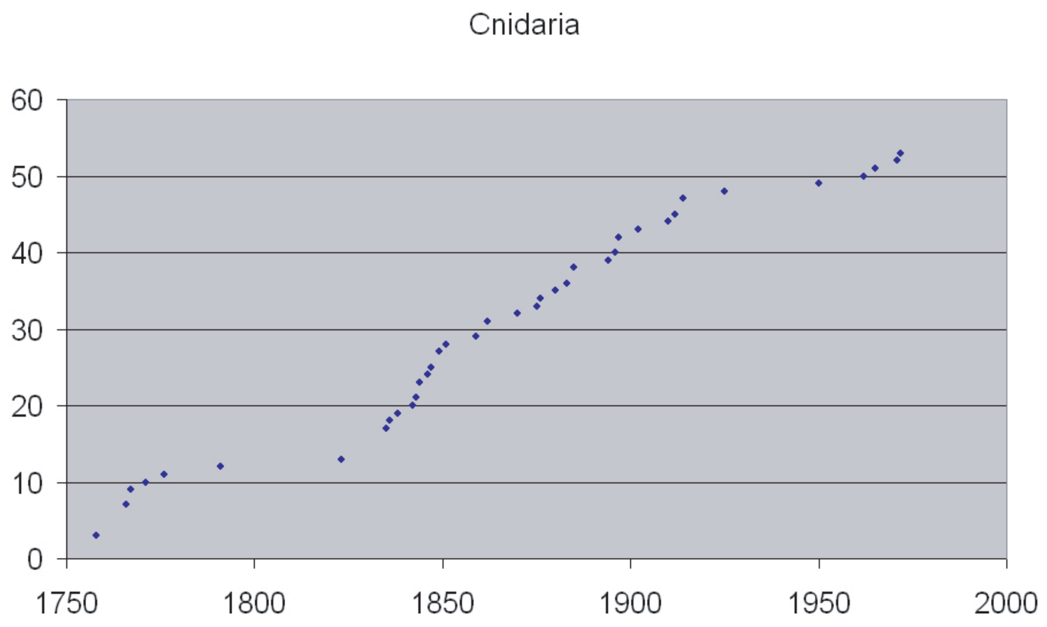

Figure 4

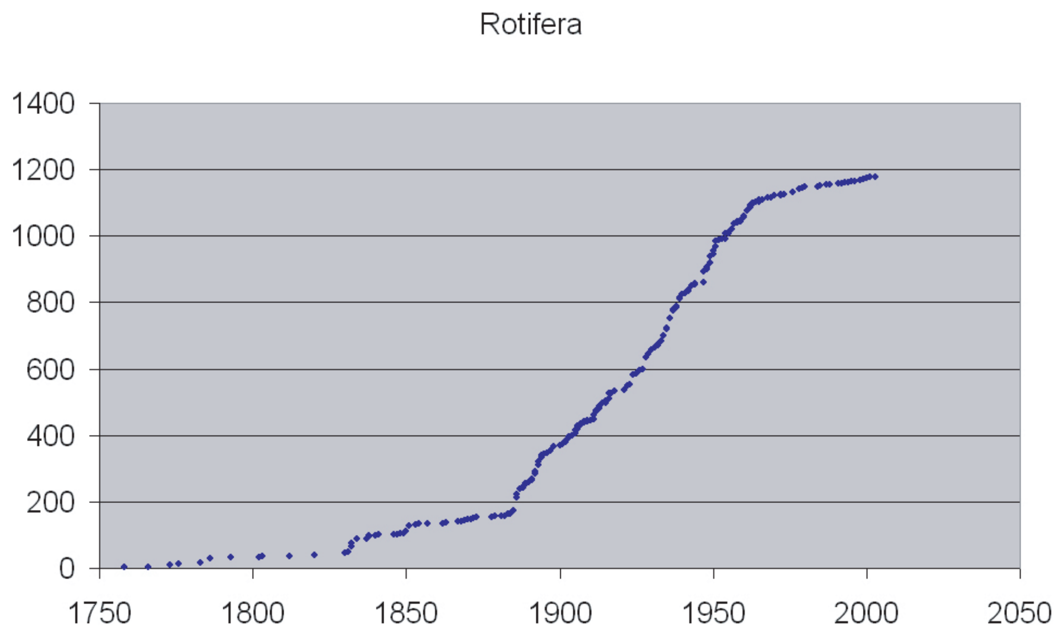

Figure 5

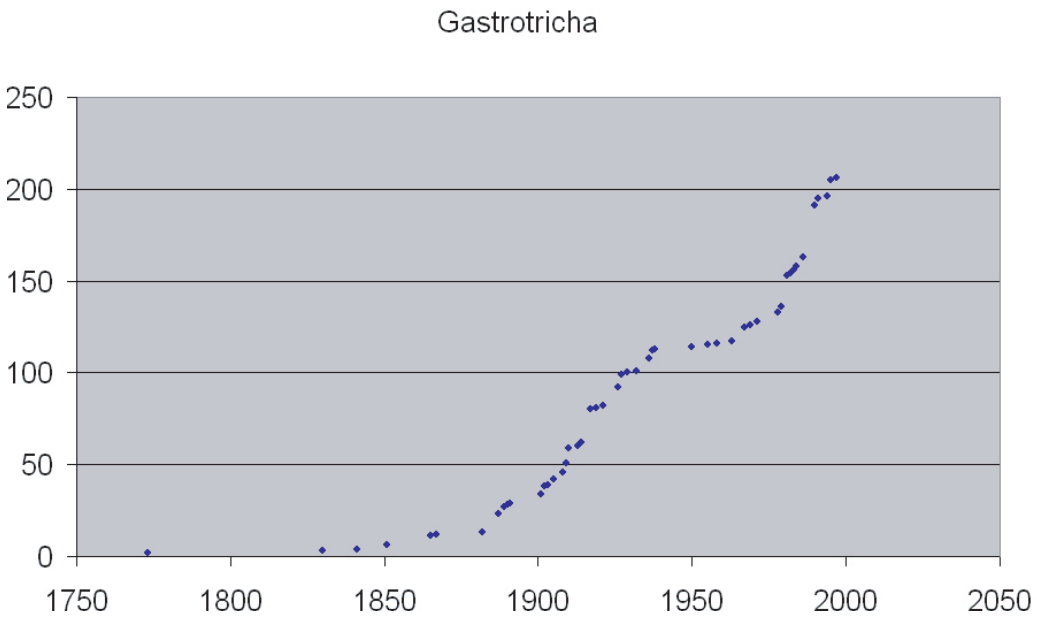

Figure 6

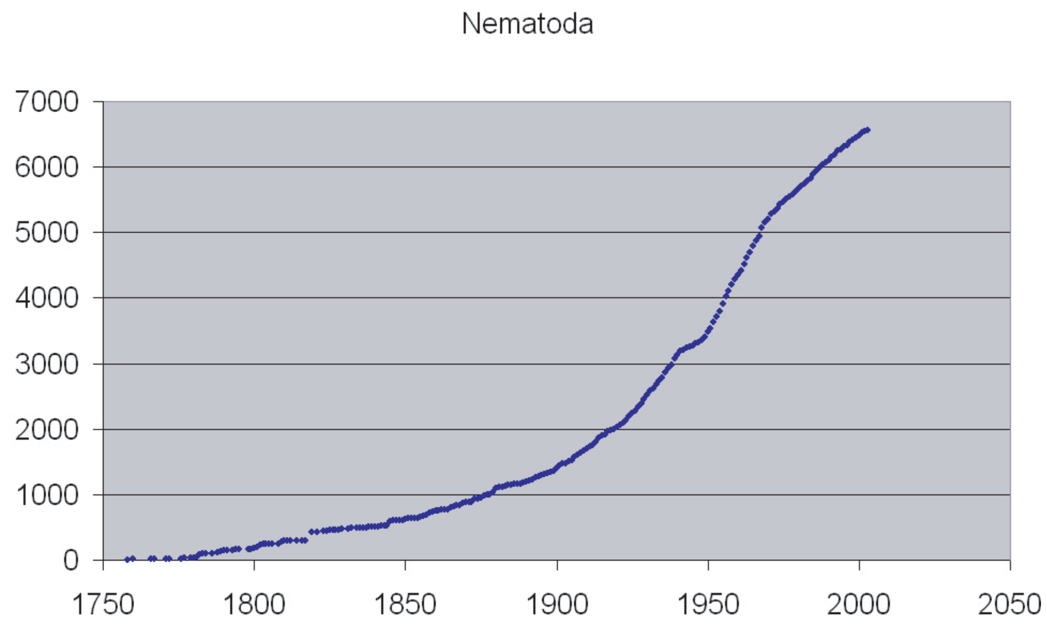

Figure 7

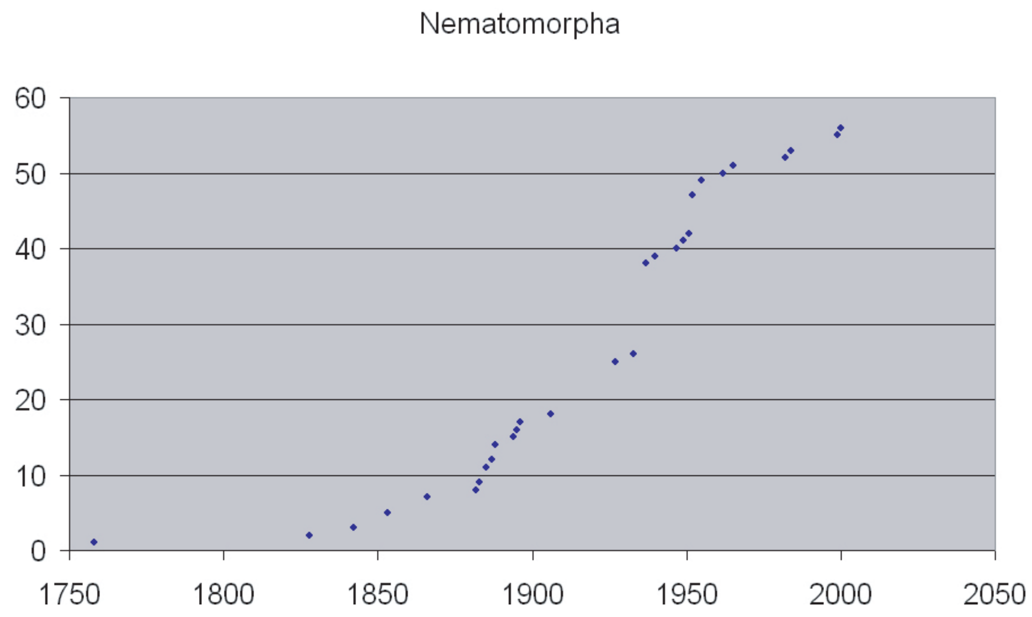

Figure 8

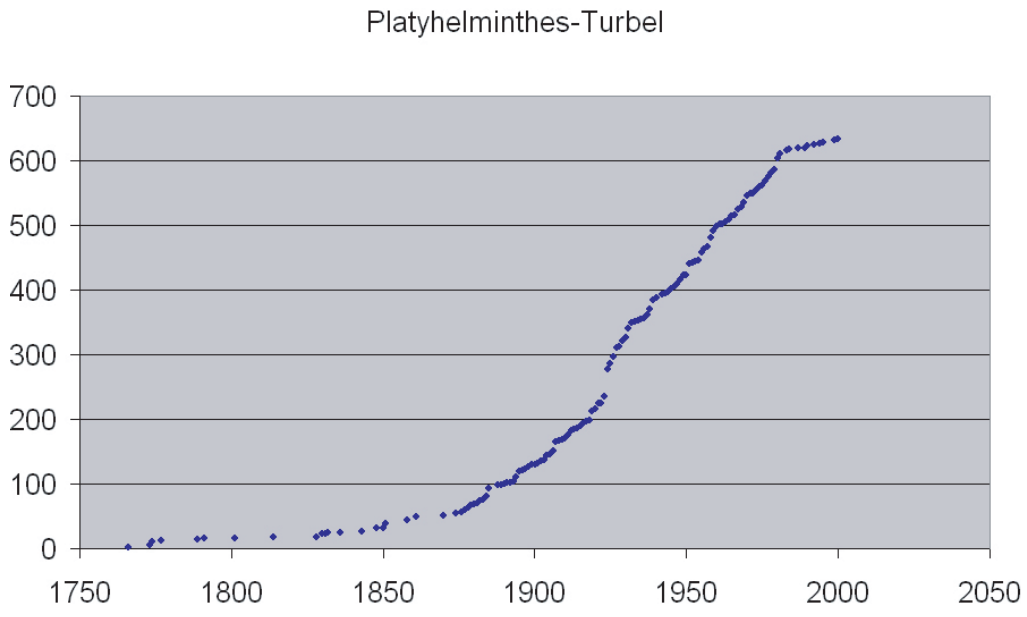

Figure 9

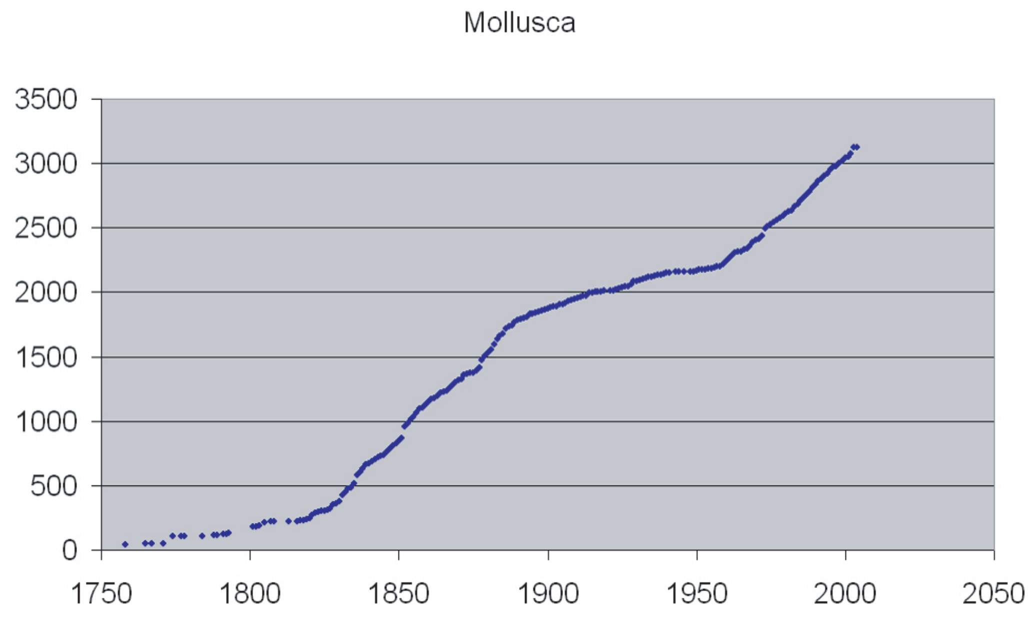

Figure 10

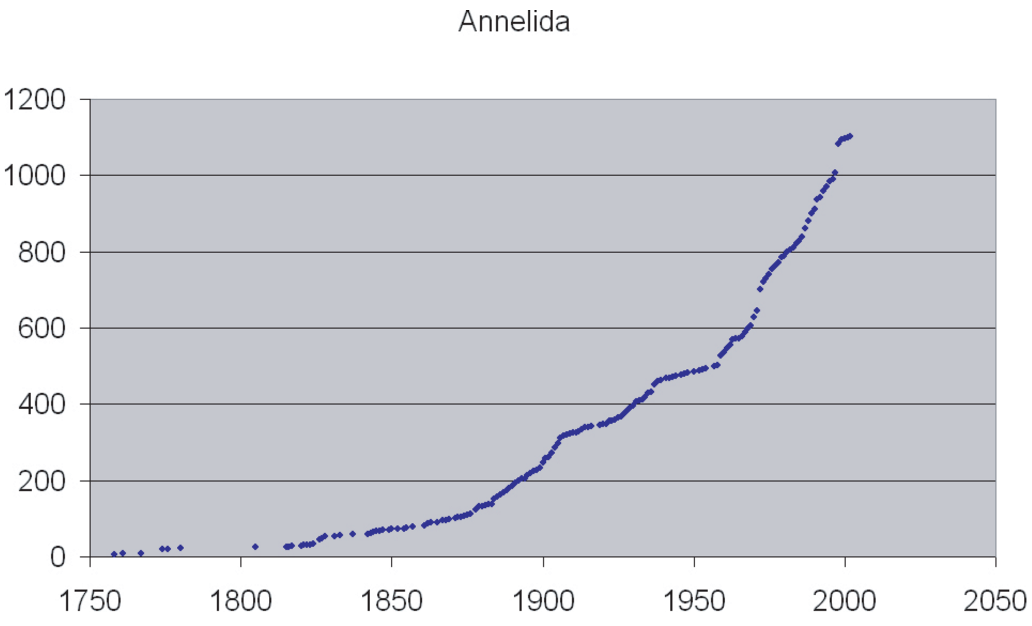

Figure 11

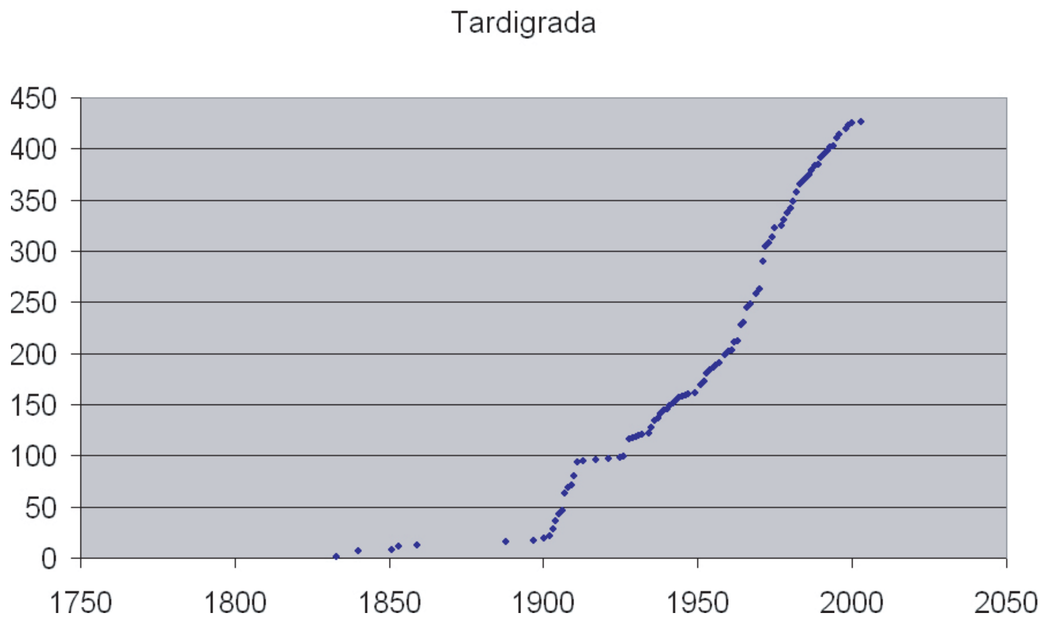

Figure 12

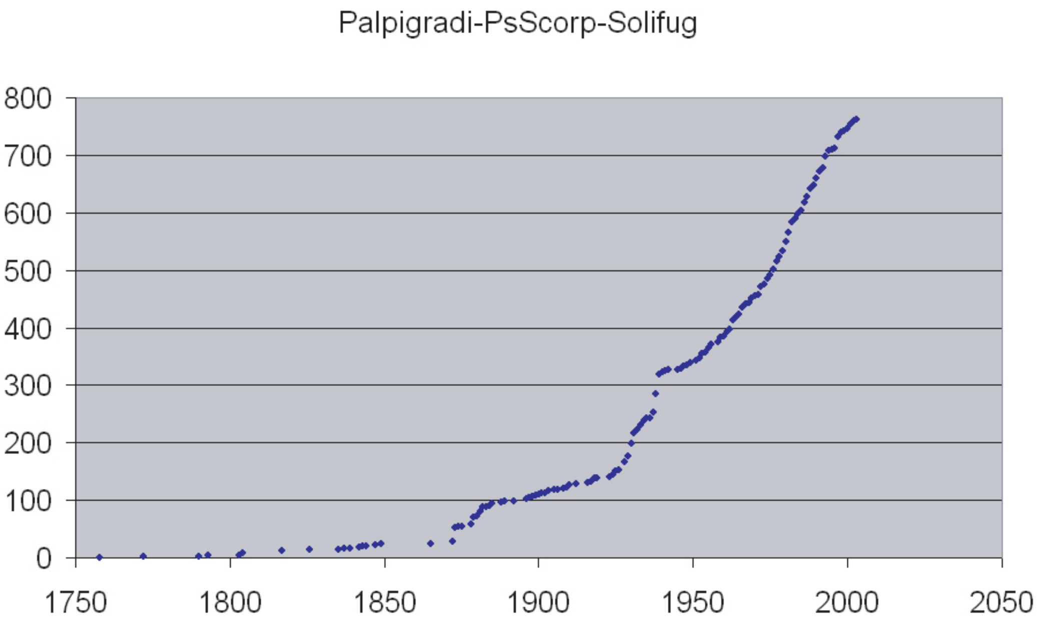

Figure 13

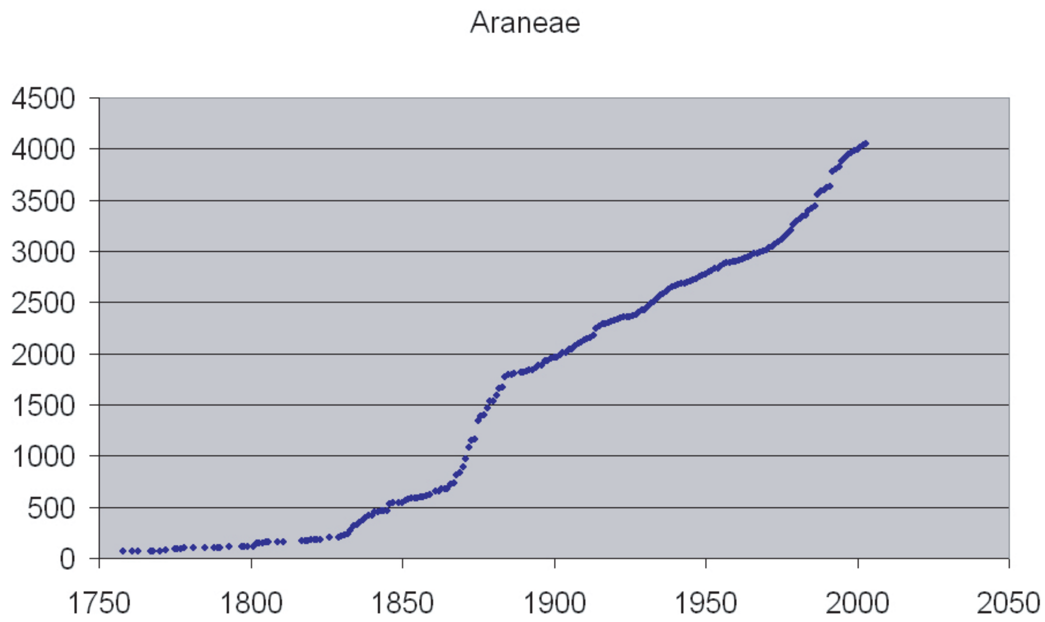

Figure 14

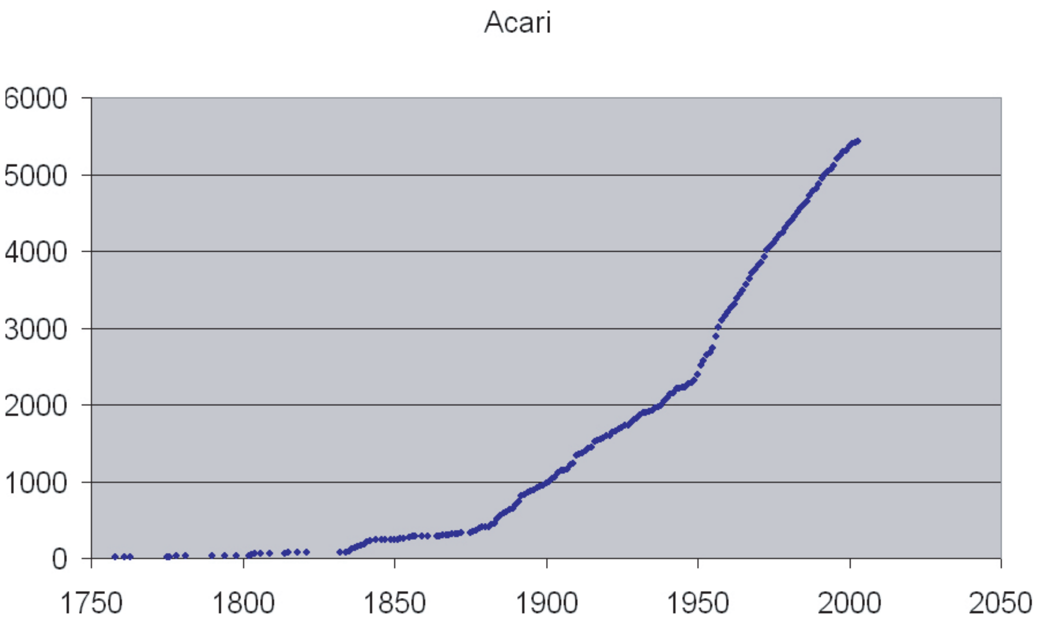

Figure 15

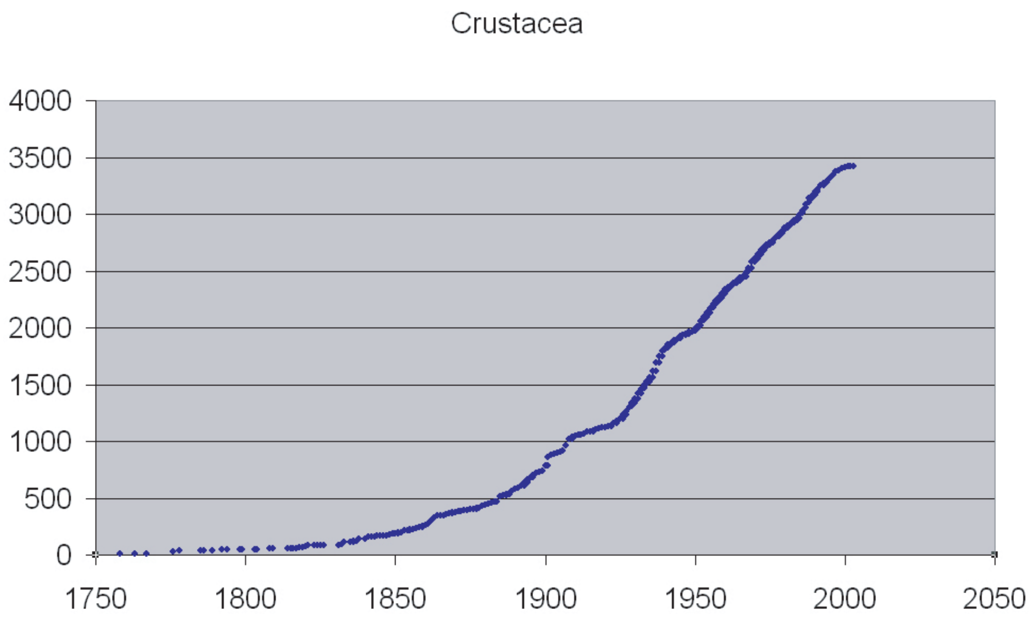

Figure 16

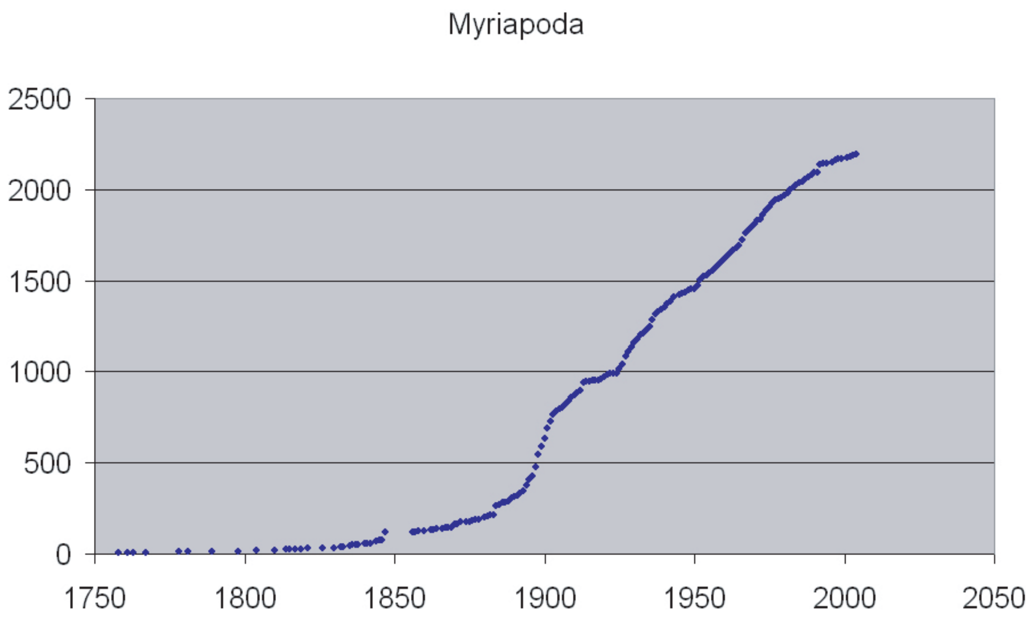

Figure 17

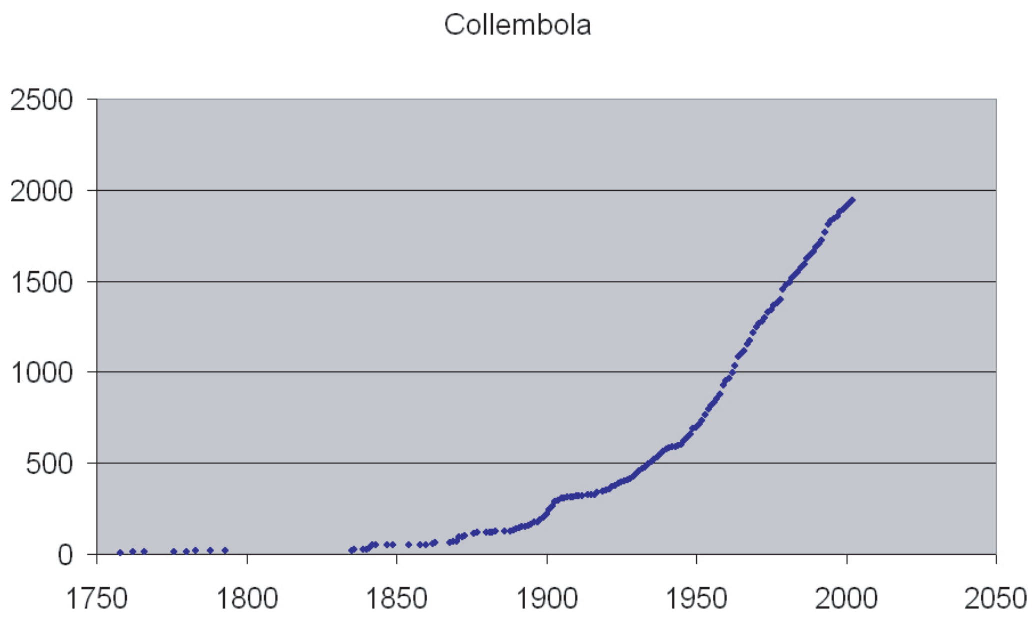

Figure 18

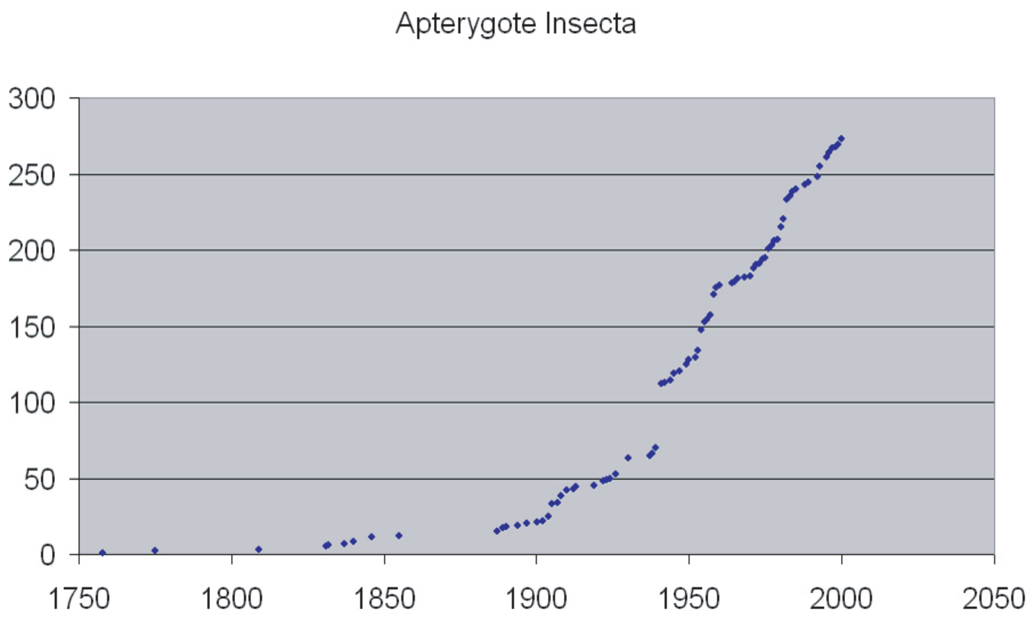

Figure 19

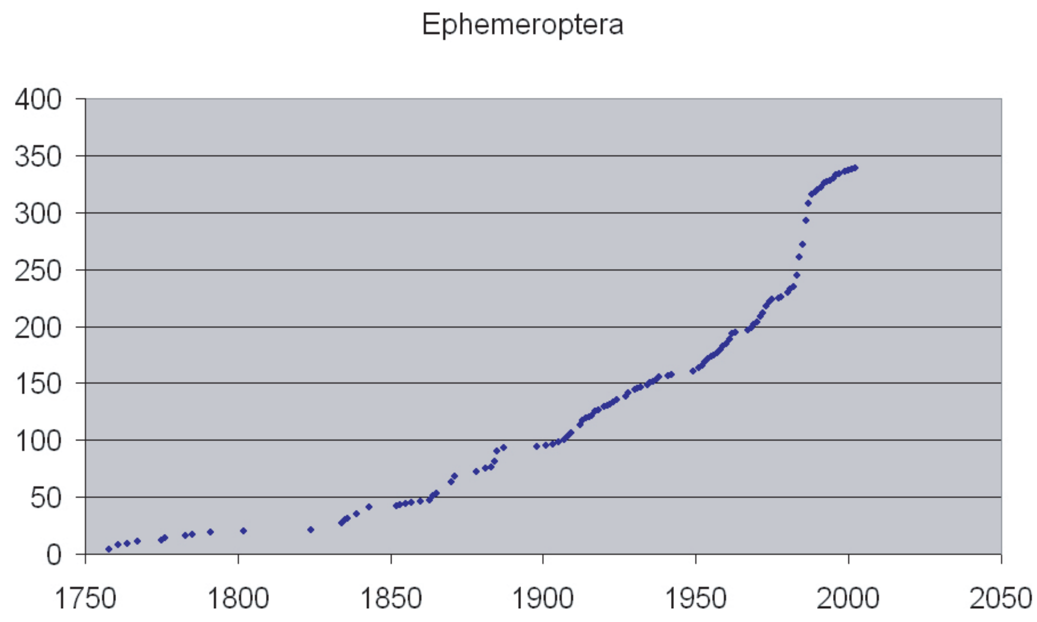

Figure 20

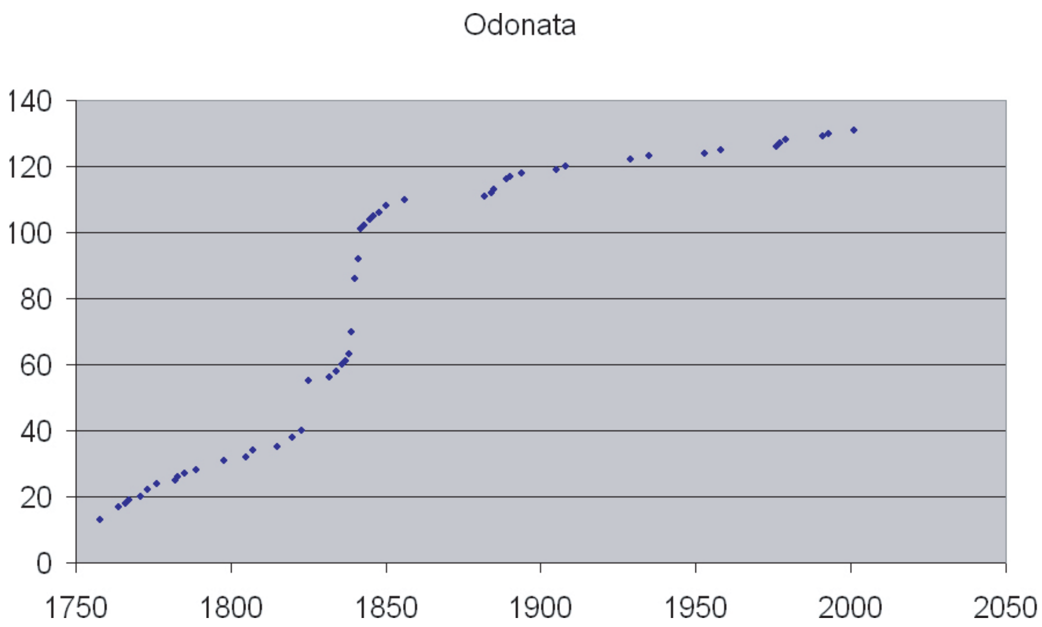

Figure 21

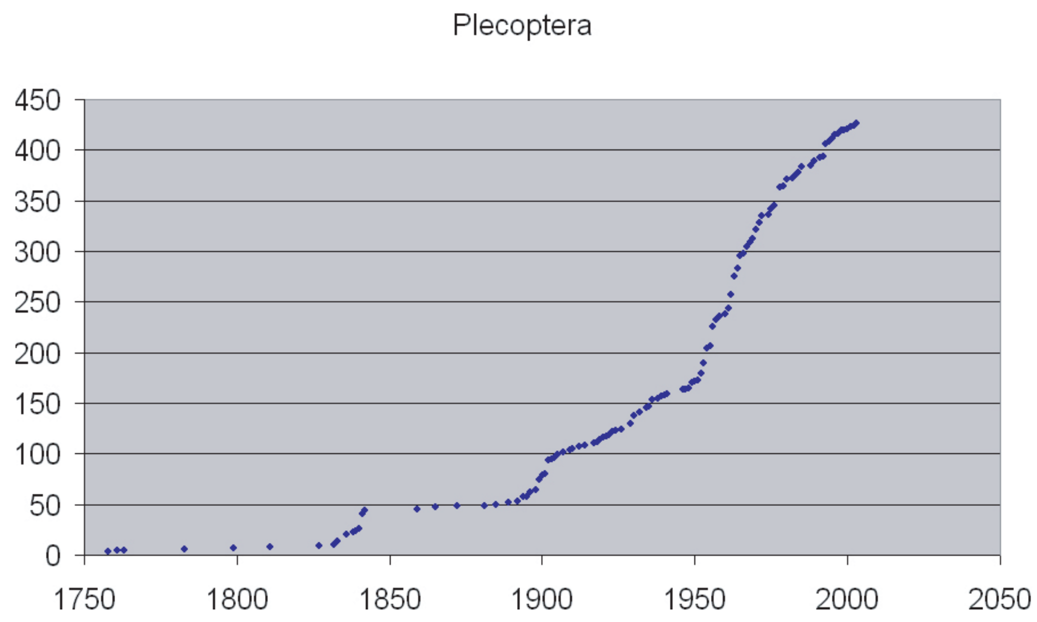

Figure 22

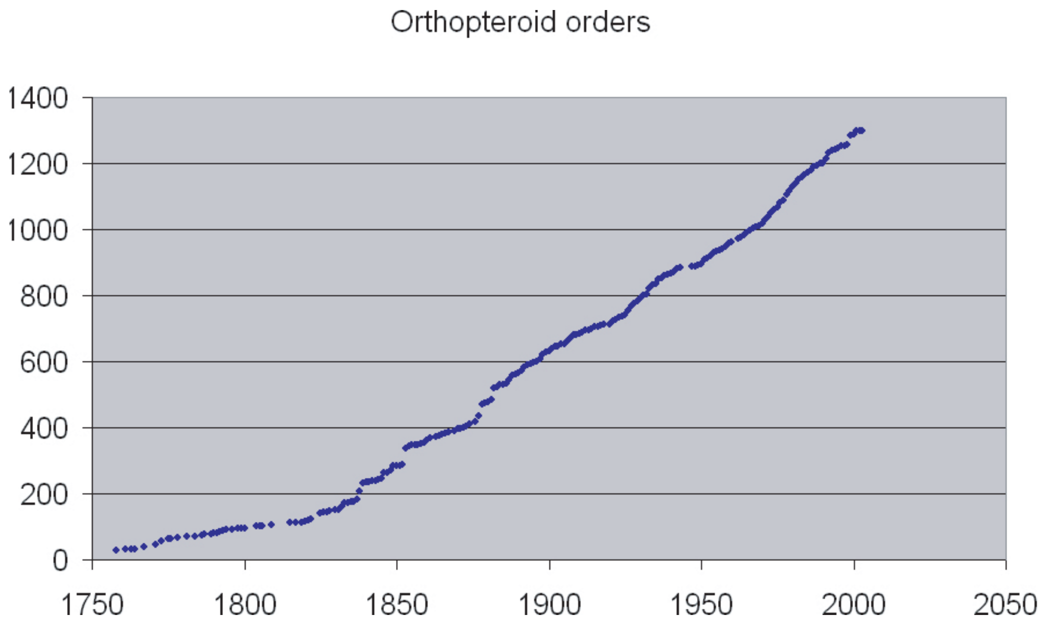

Figure 23

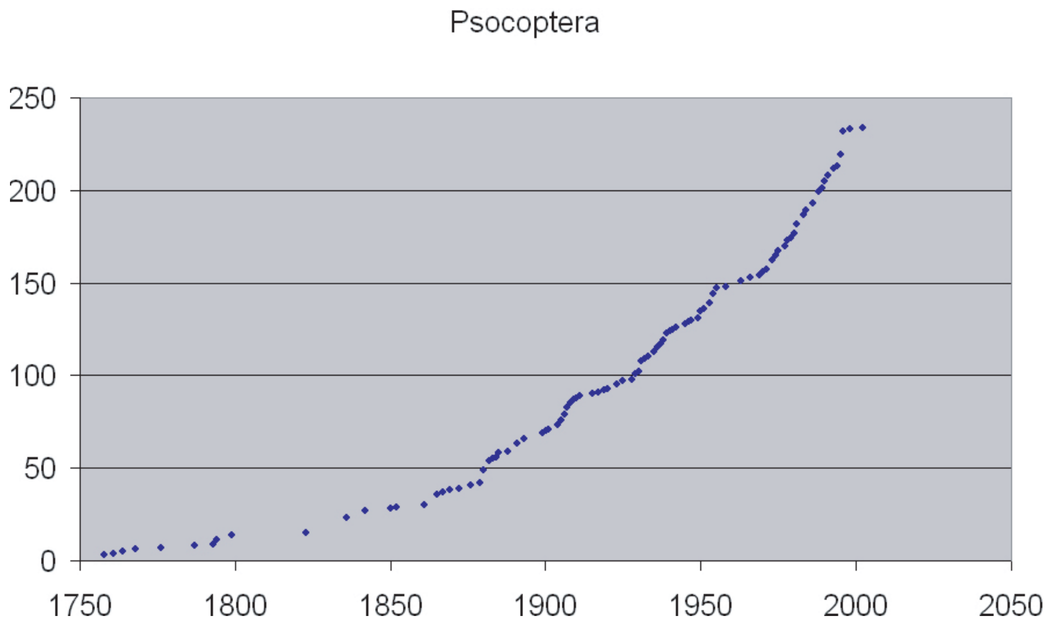

Figure 24

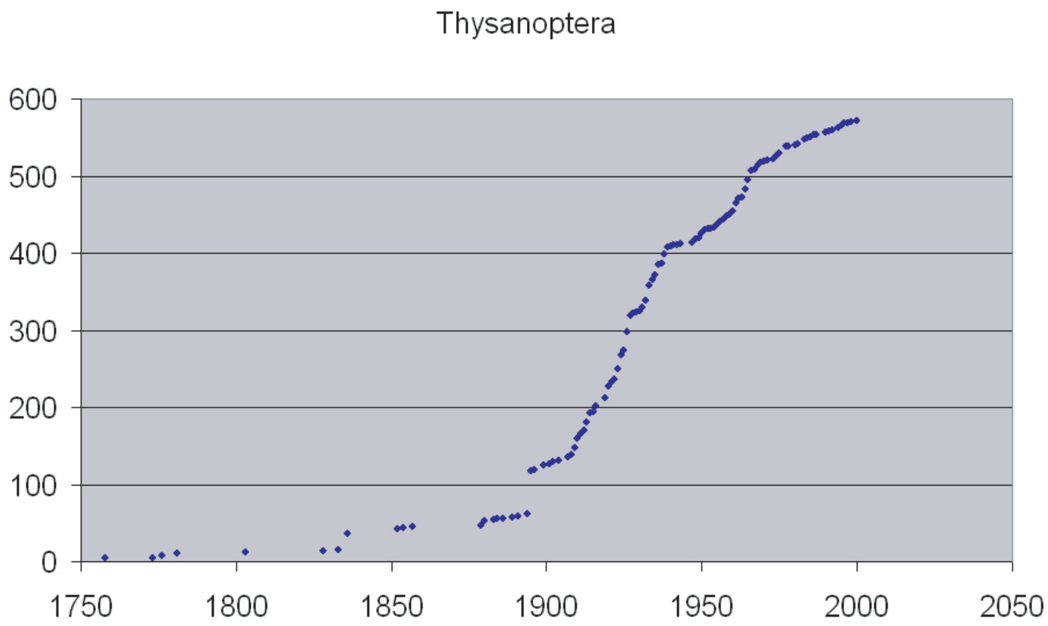

Figure 25

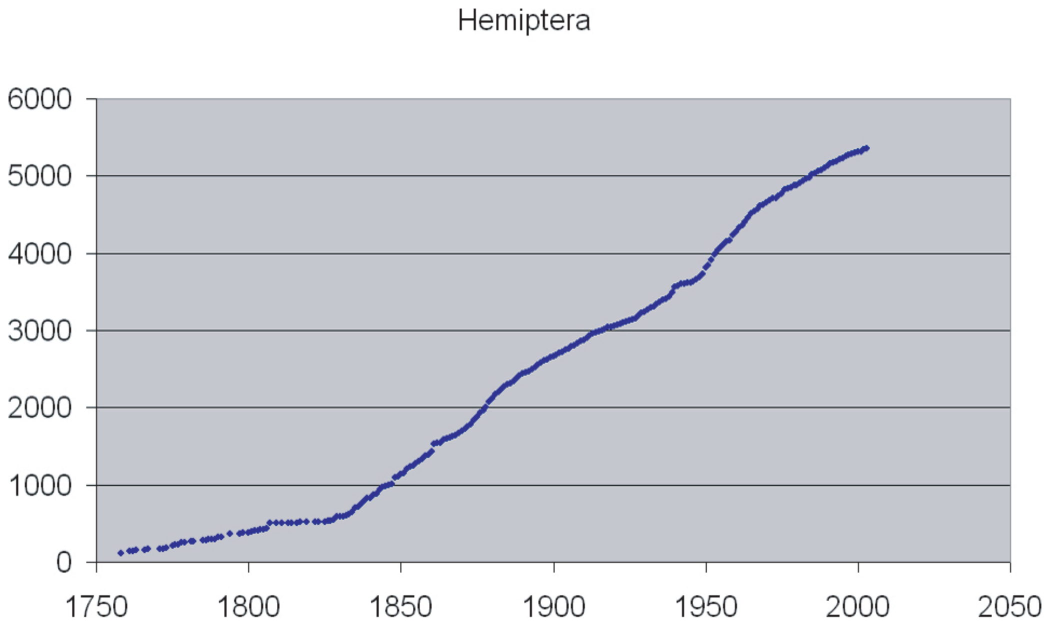

Figure 26

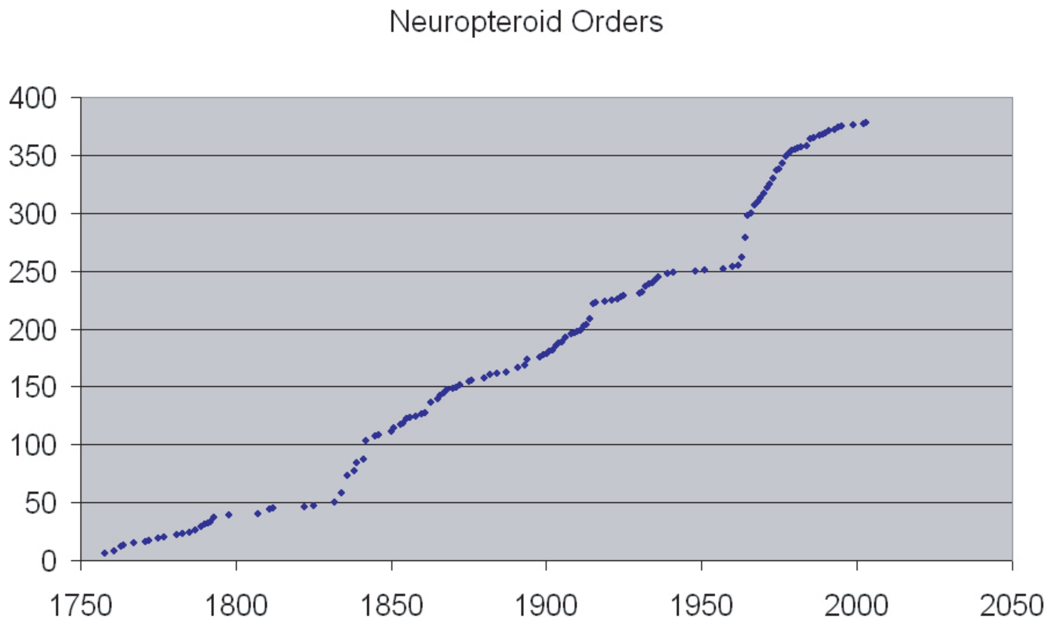

Figure 27

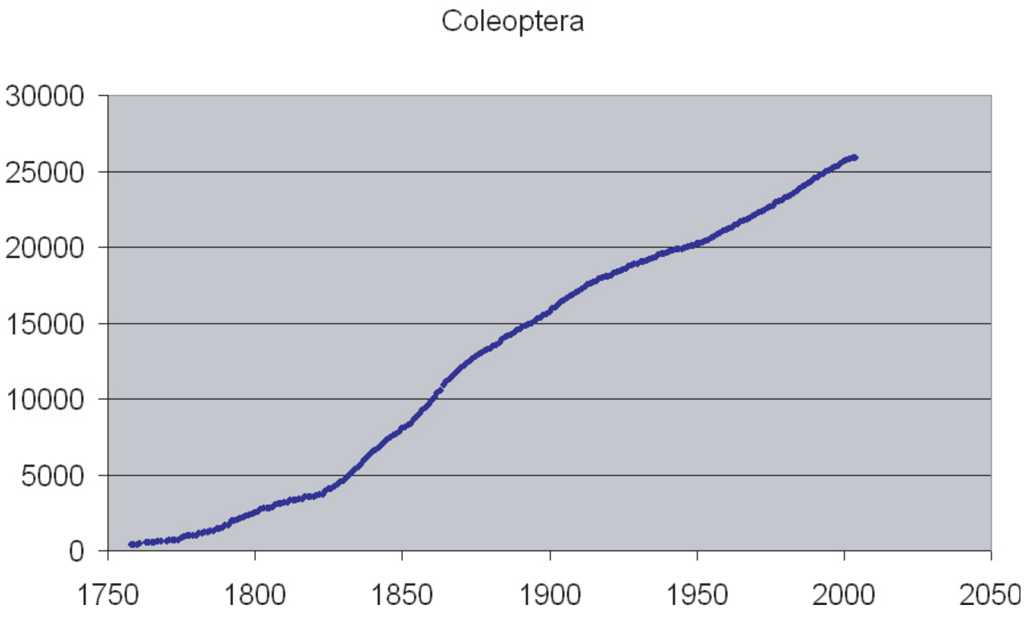

Figure 28

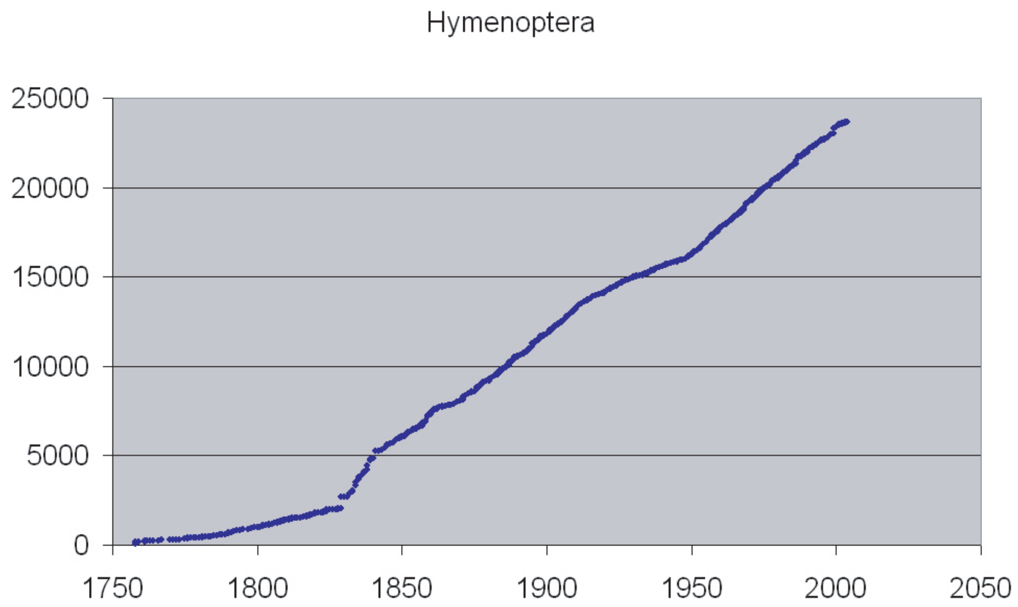

Figure 29

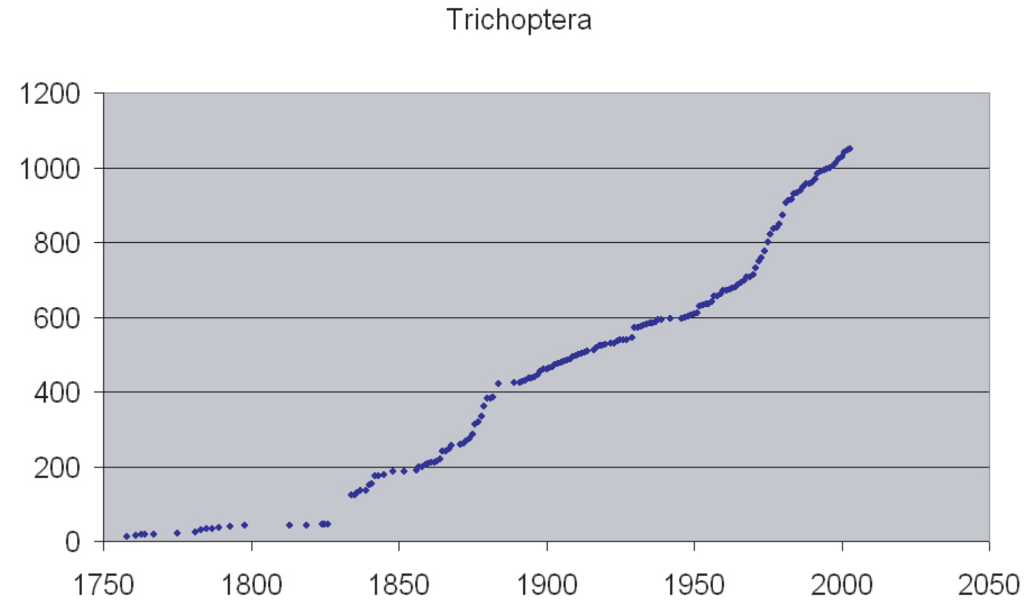

Figure 30

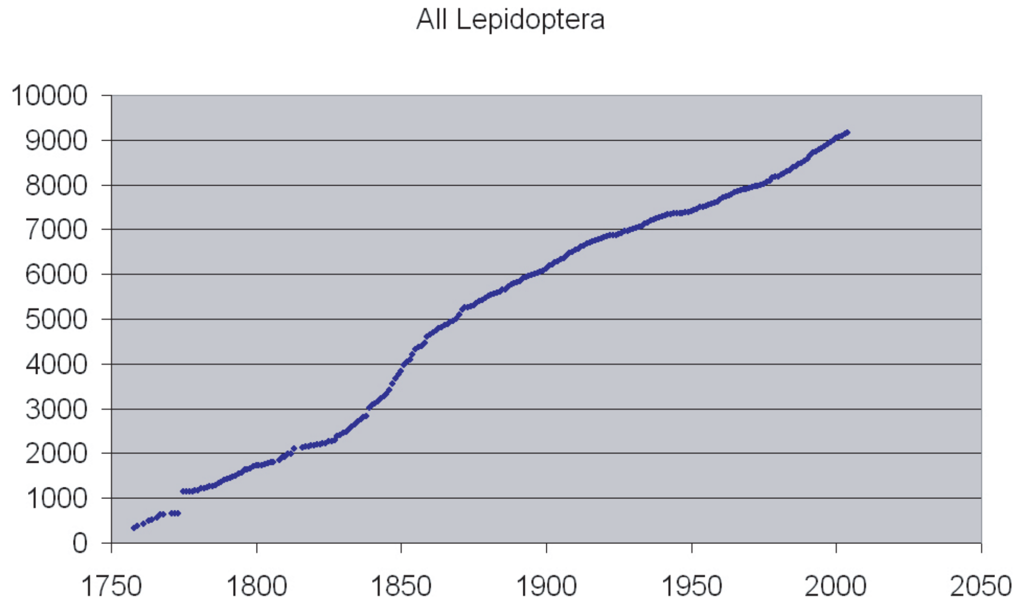

Figure 31

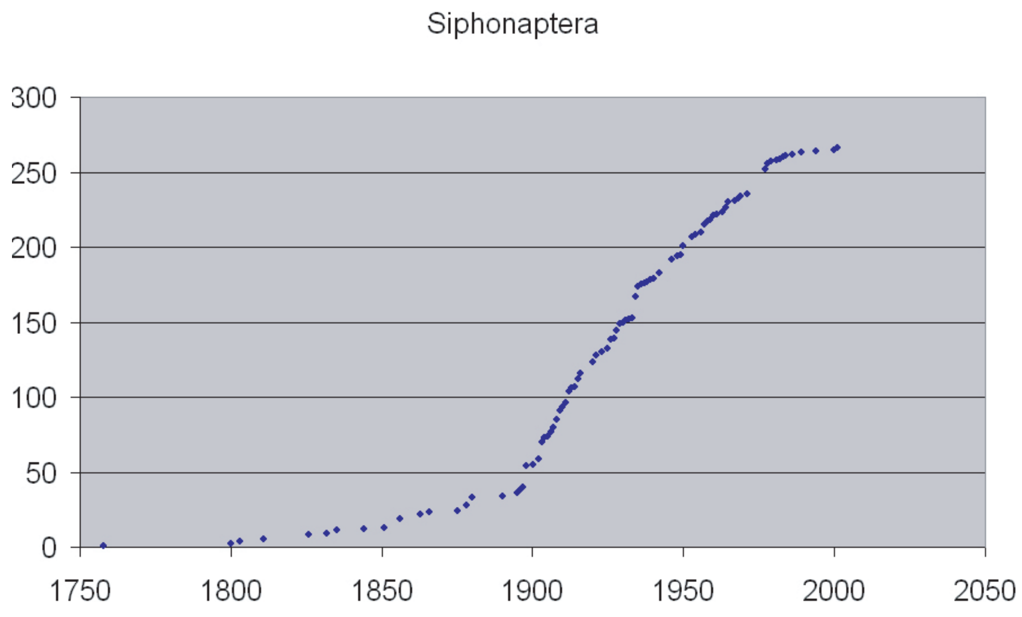

Figure 32

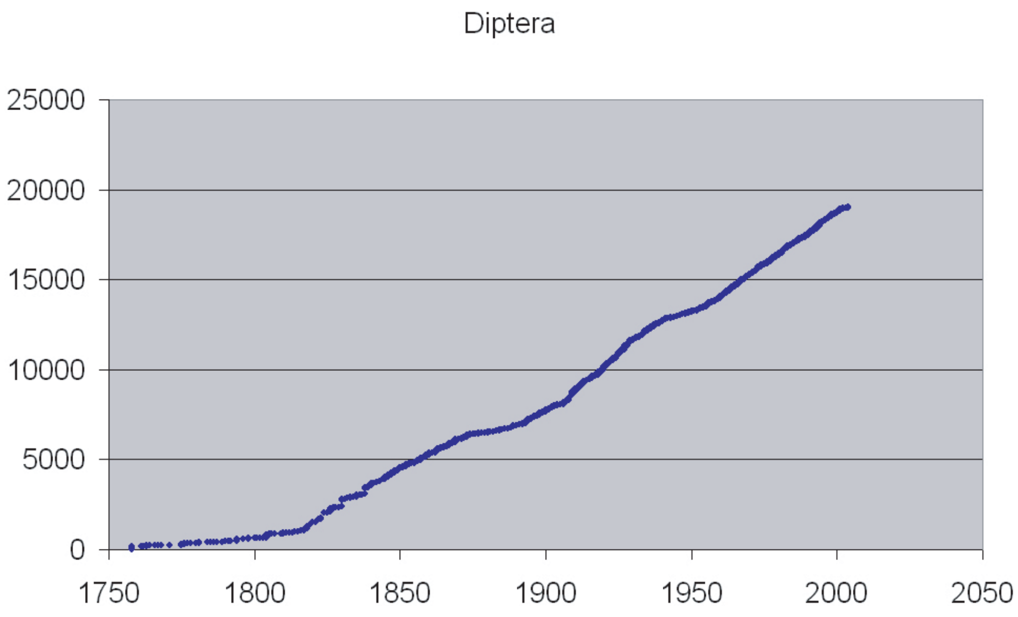

Figure 33

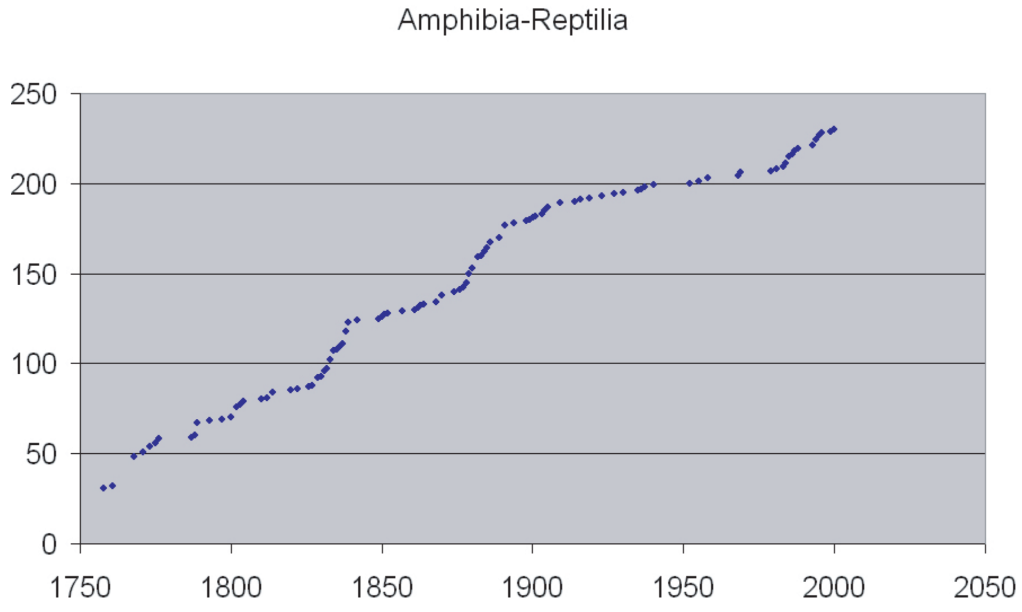

Figure 34

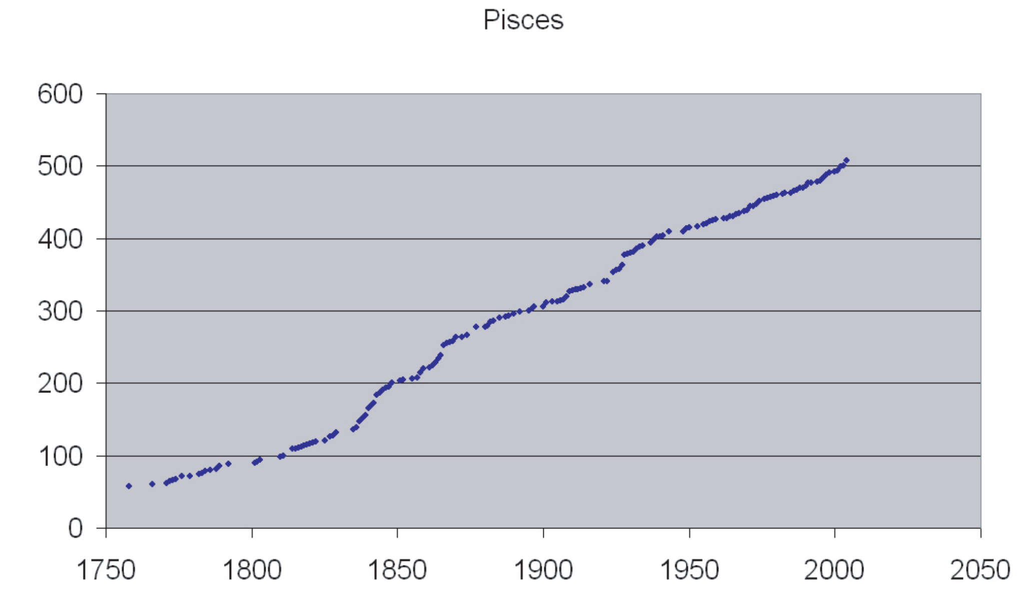

Figure 35

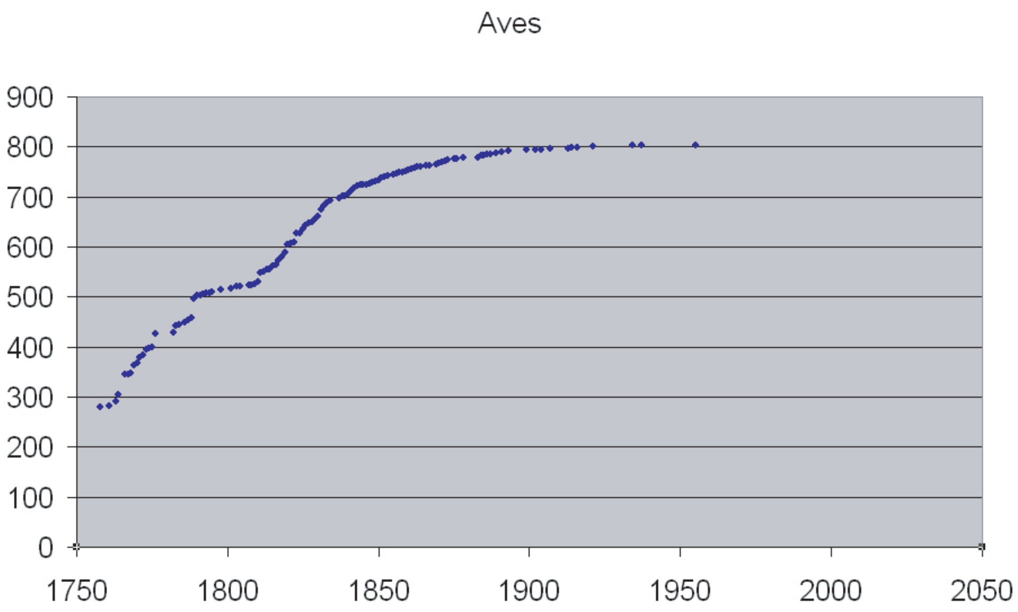

Figure 36

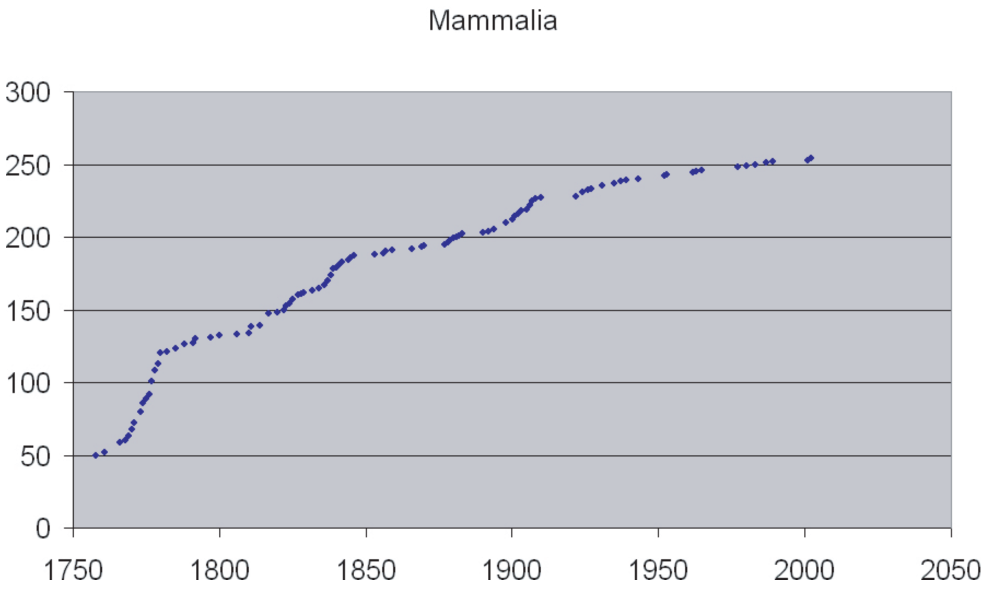

Figure 37

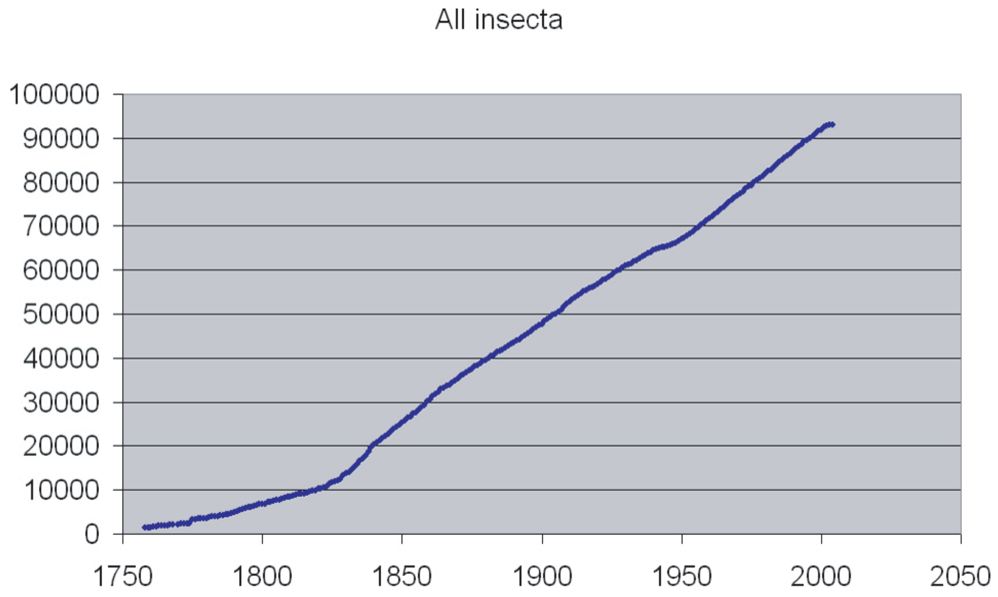

Figure 38

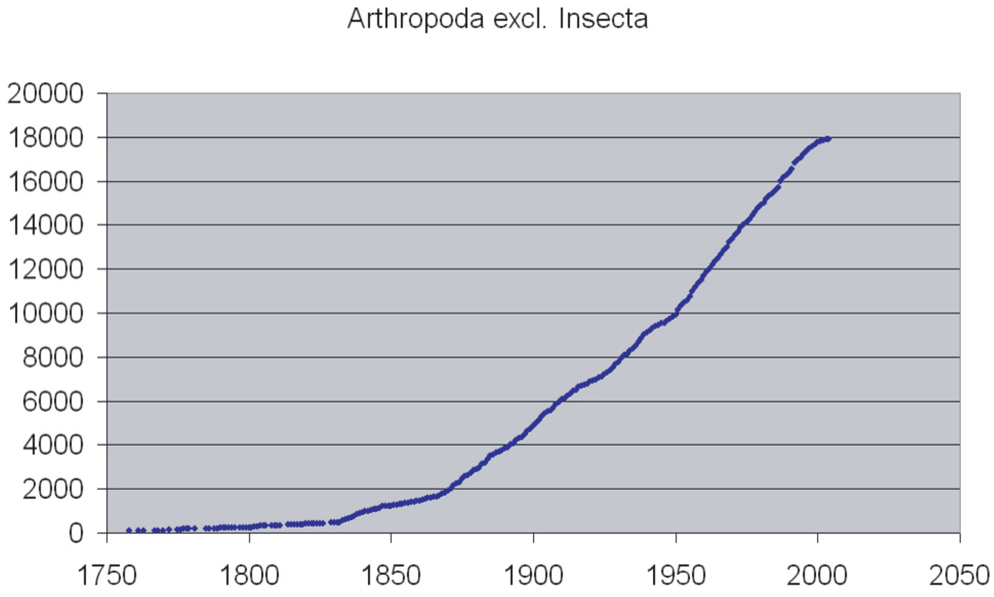

Figure 39

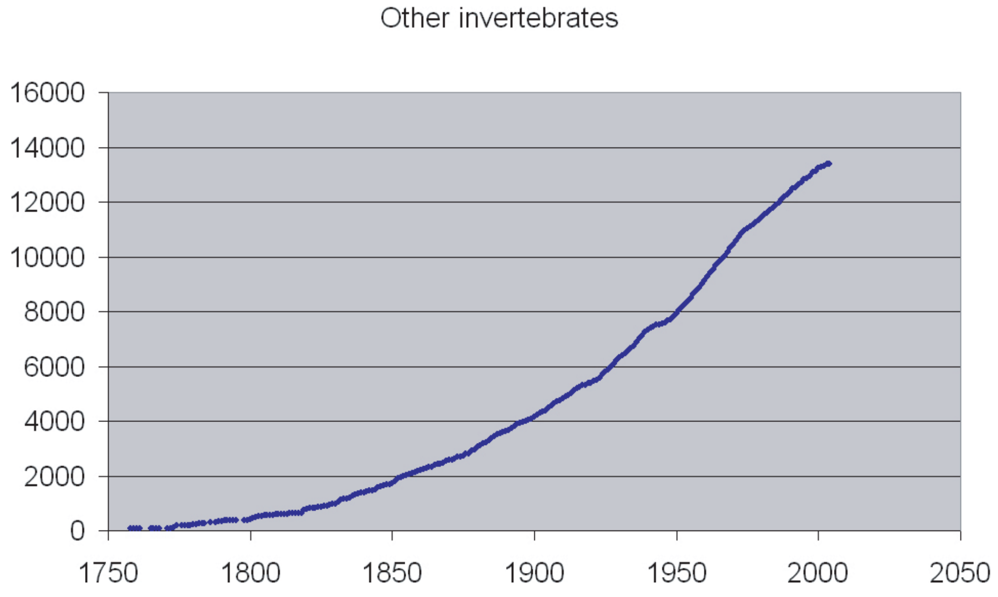

Figure 40

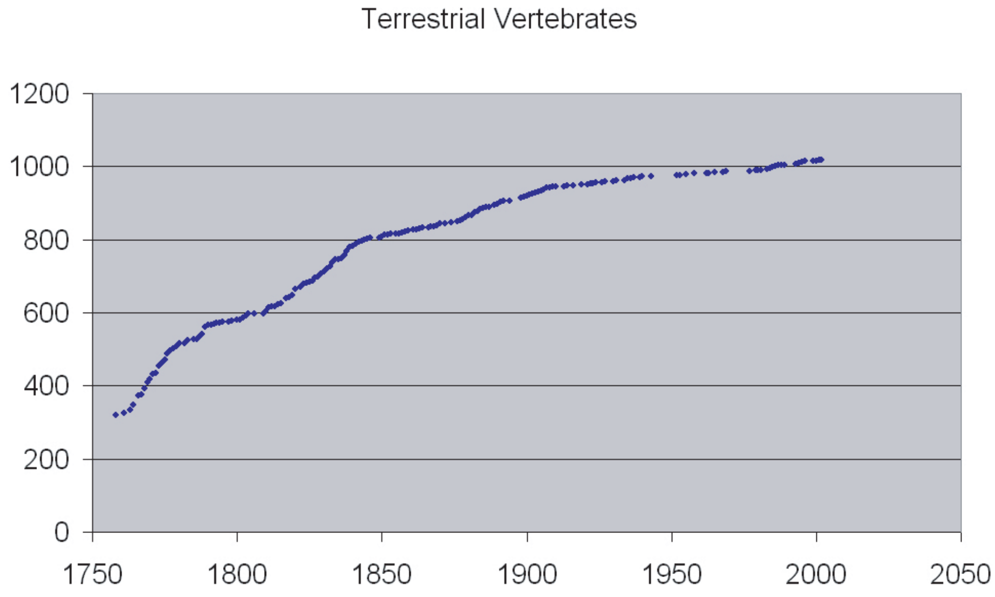

Figure 41

APPENDIX II: FIGURES, 1998-2002

Figures and graphs representing data extracted from the Biosis dataset (Zoological Record), years 1998-2002.

Figure 42: repartition of the new species descriptions in Europe, 1998-2002, with numbers of new species.

Figure 43: Numbers of new species descriptions in Europe, 1998-2002. Total: 3161 n. sp.

Figure 44: Descriptions of new species in Europe, 1998-2002: numbers of new species per country/island divided by country/island area x 10^5. For Russia and Turkey, the surface used is the surface of European Turkey and of Russia west of Ural and north of Caucasus. Total: 3161 n. sp.

Figure 45: Numbers of new species descriptions in Europe per country of descriptor, 1998-2002. Total: 3149 descriptions.

Figure 46: Country of descriptors, 1998-2002: numbers of descriptors per country. Total: 855 descriptors (only first descriptors are taken into account. See text).

Figure 47: Country of descriptors, 1998-2002: numbers of descriptors per country divided by country population x 10^7.

Figure 48: Country of descriptors, 1998-2002: numbers of descriptors per country divided by GDP x 100

Figure 49: % of new species described by taxonomists working in the species country, 1998-2002.

Figure 50: For each affiliation country, % of new species described from countries different from affiliation country, 1998-2002.

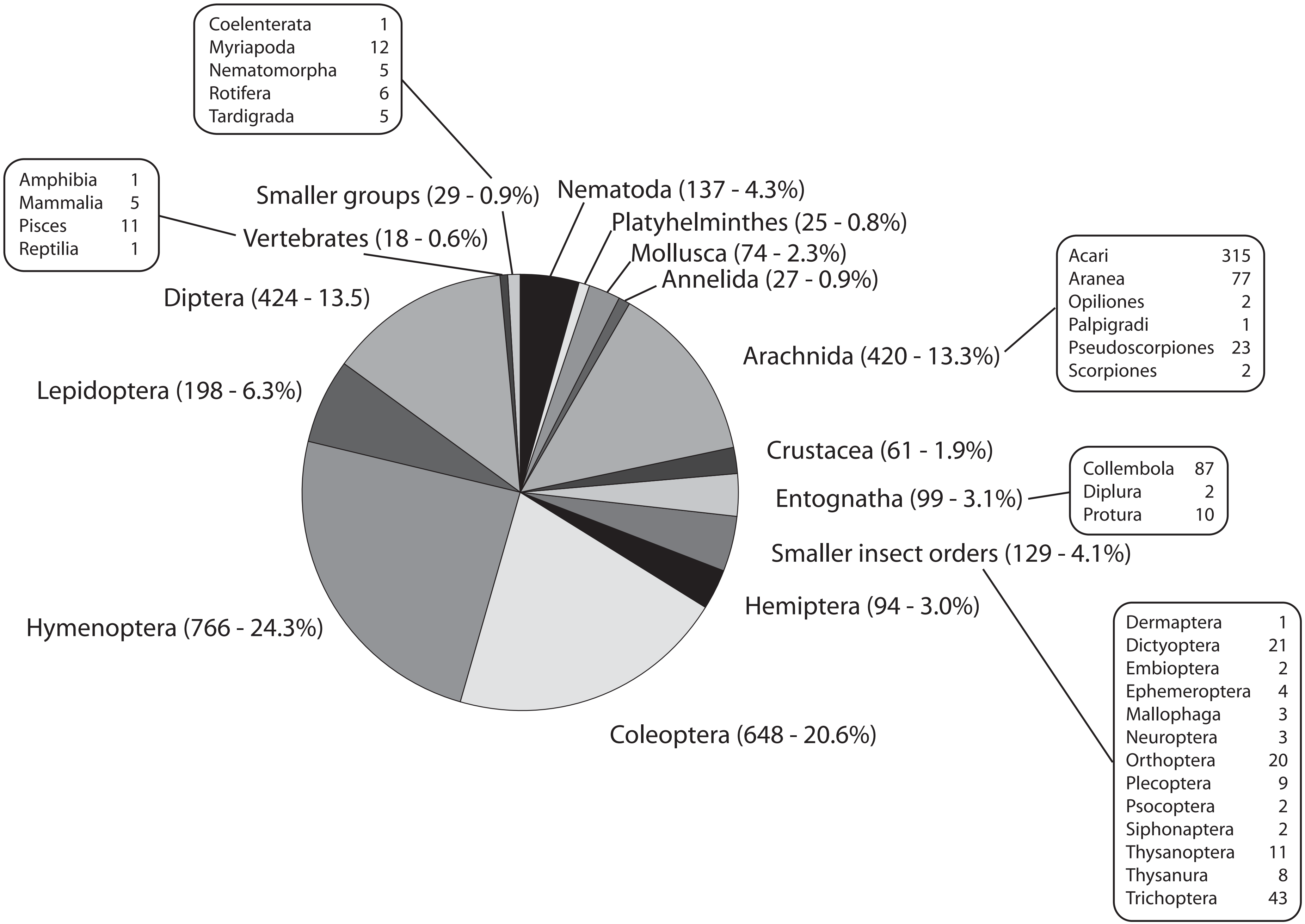

Figure 42: Taxonomic repartition of the new species descriptions in Europe, 1998-2002, with numbers of new species and percentage of total number.

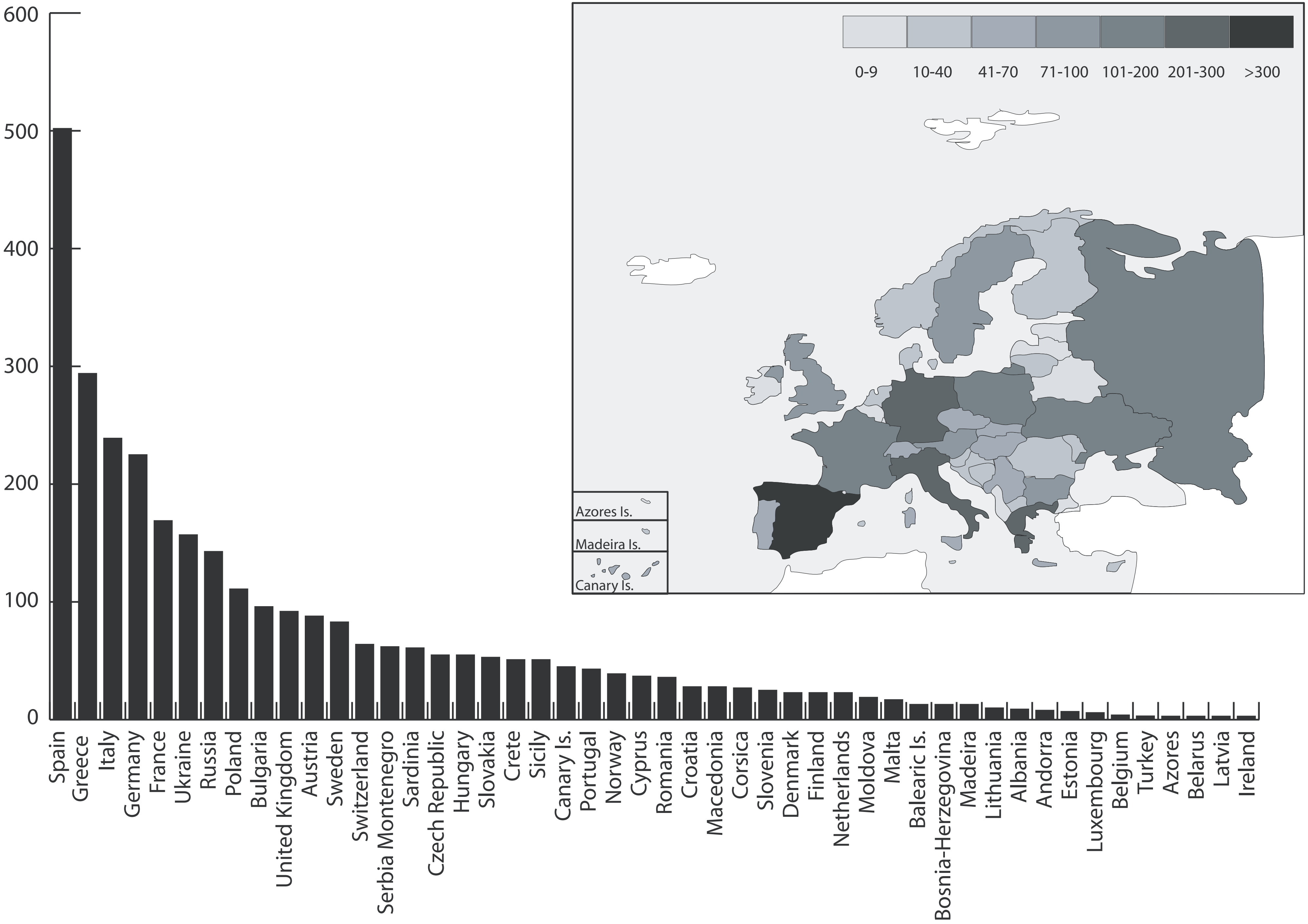

Figure 43: Numbers of new species descriptions in Europe, 1998-2002. Total: 3161 n. sp.

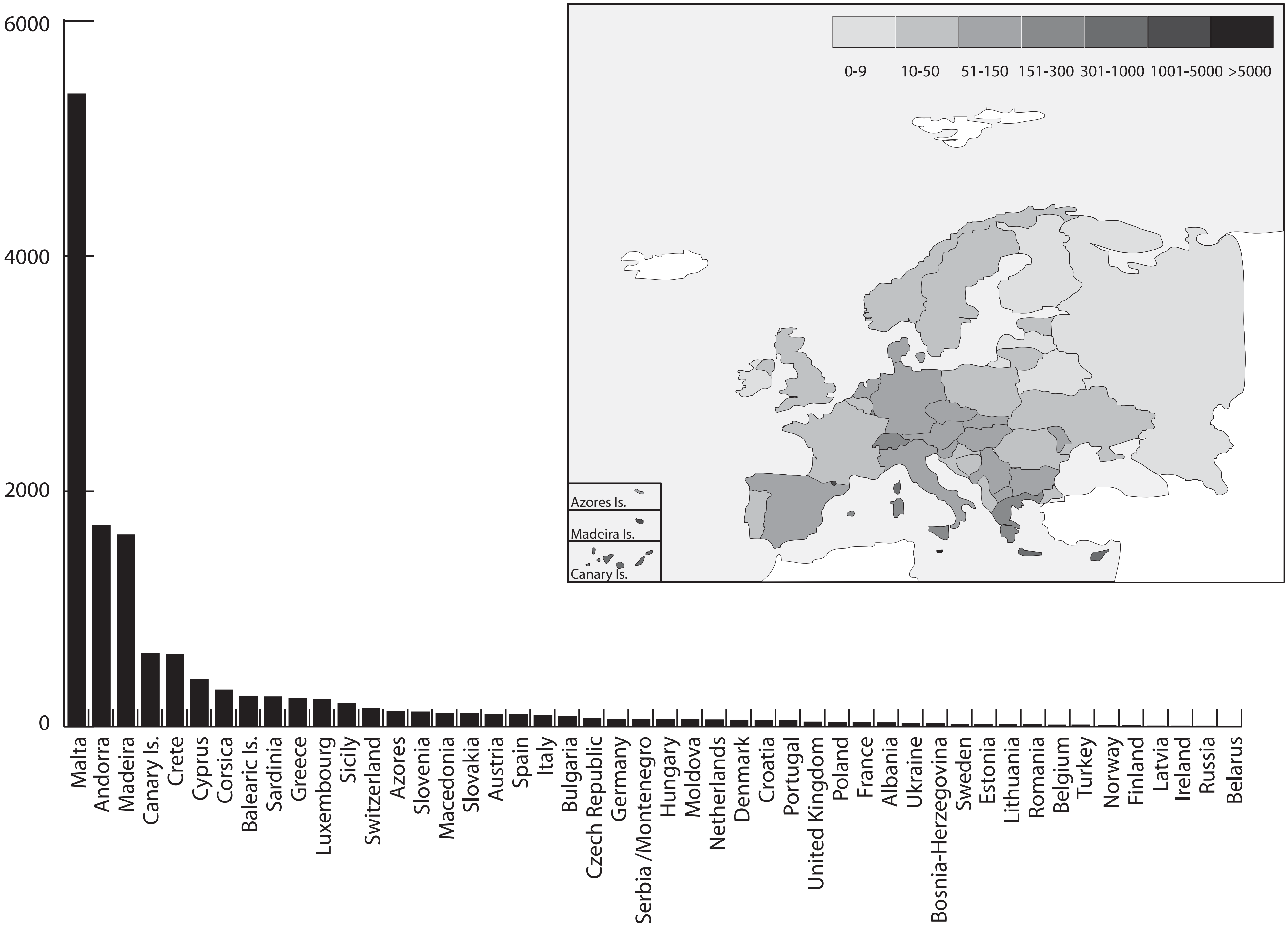

Figure 44: Descriptions of new species in Europe, 1998-2002: numbers of new species per country or island divided by country or island area x 10^5. For Russia and Turkey, the surface used in the surface of European Turkey and Russia west of Ural and north of Caucasus. Total: 3161 n. sp.

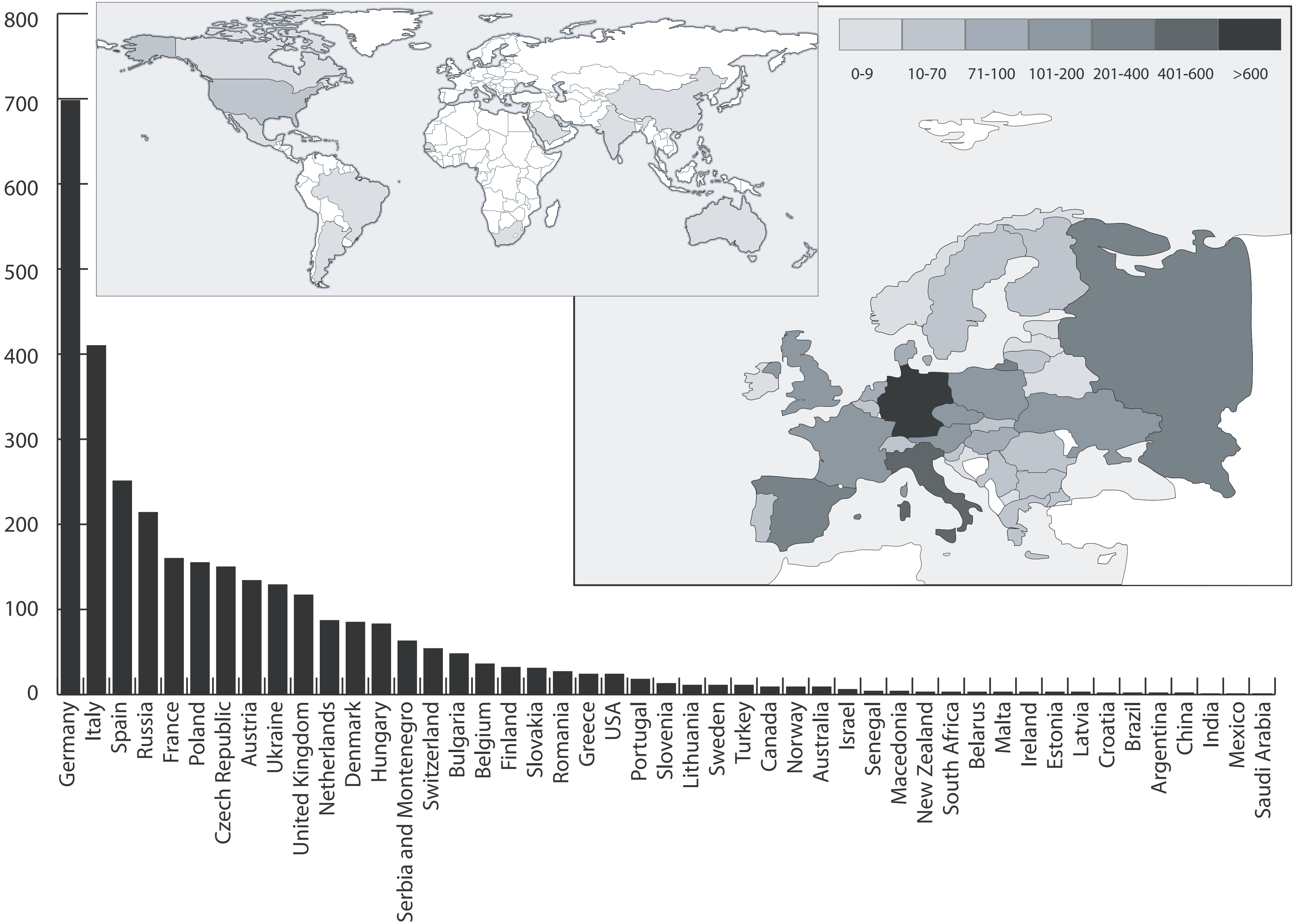

Figure 45: Numbers of new species descriptions in Europe per country of descriptor, 1998-2002. Total: 3149 descriptions.

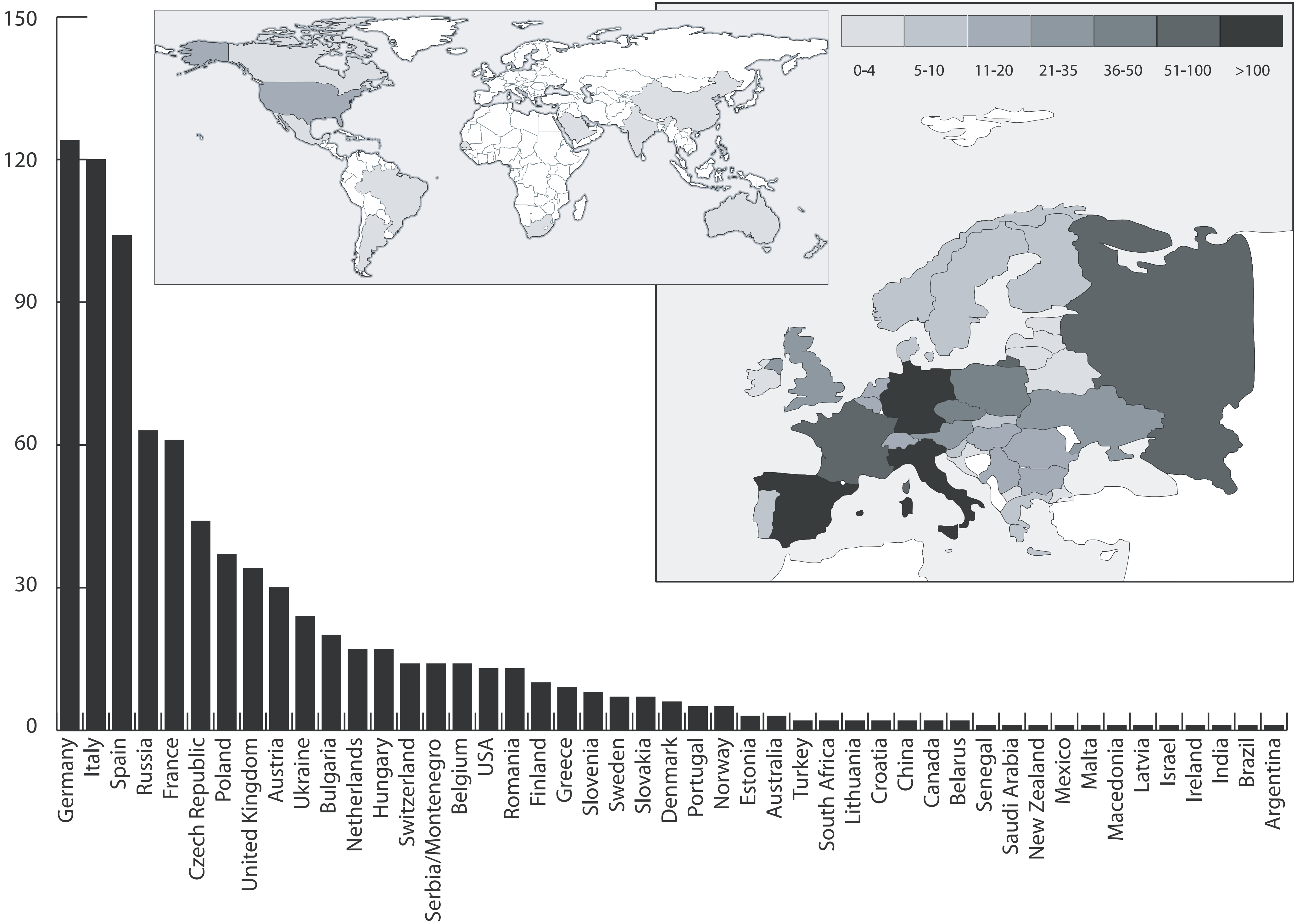

Figure 46: Country of descriptors, 1998-2002: numbers of descriptors per country. Total: 855 descriptors (only first descriptors are taken into account. See text).

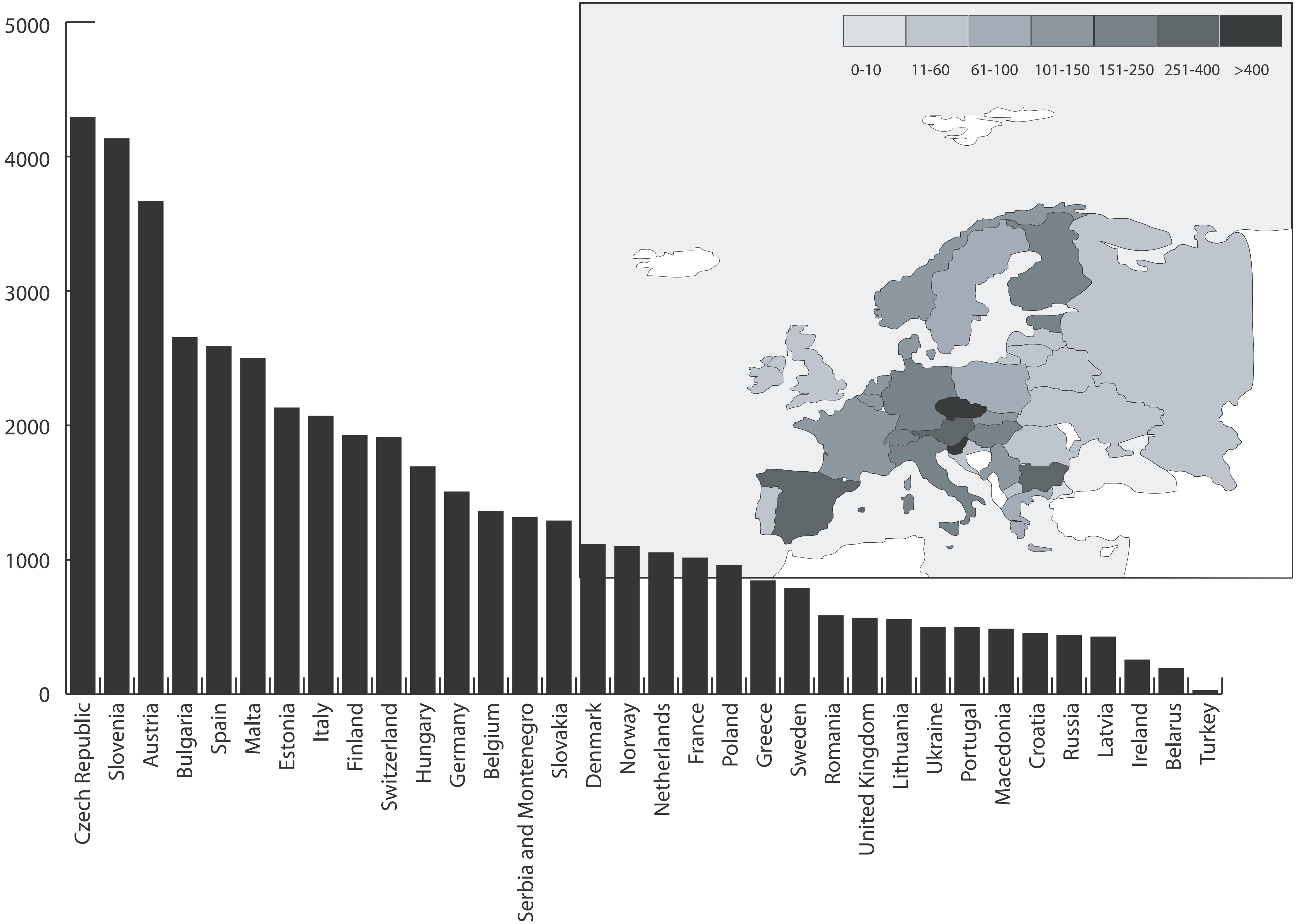

Figure 47: Country of descriptors, 1998-2002: numbers of descriptors per country divided by country population x 10^7.

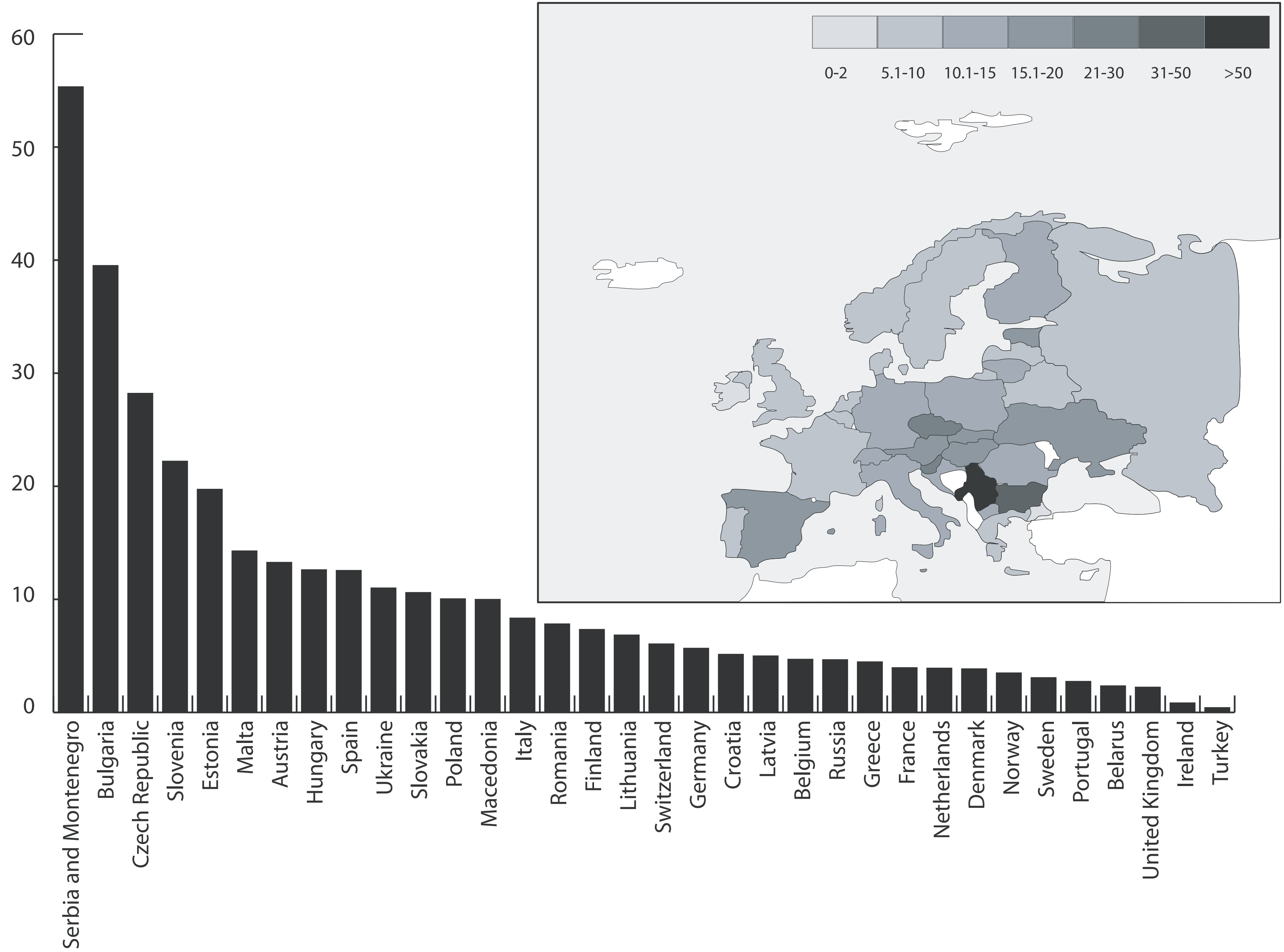

Figure 48: Country of descriptors, 1998-2002: numbers of descriptors per country divided by GDP x 100

Figure 49: % of new species described by taxonomists working in the species country, 1998-2002.
Note: countries where 10 or less species were described have been excluded (see Table 10).

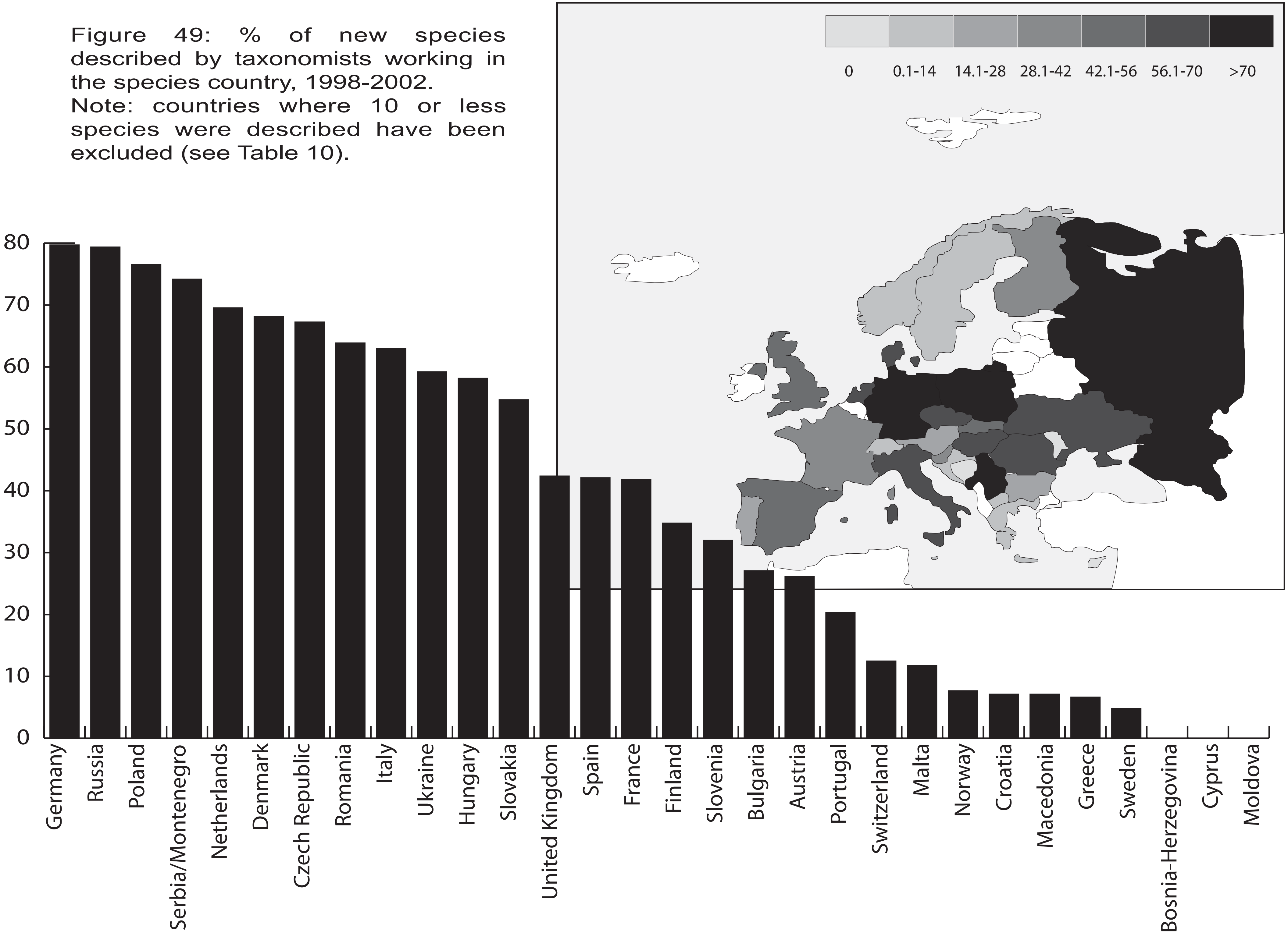

Figure 50: For each affiliation country, % of new species described from countries different from affiliation country, 1998-2002.
Note : affiliation countries where taxonomists described less than 20 species have been excluded (see Table 11).

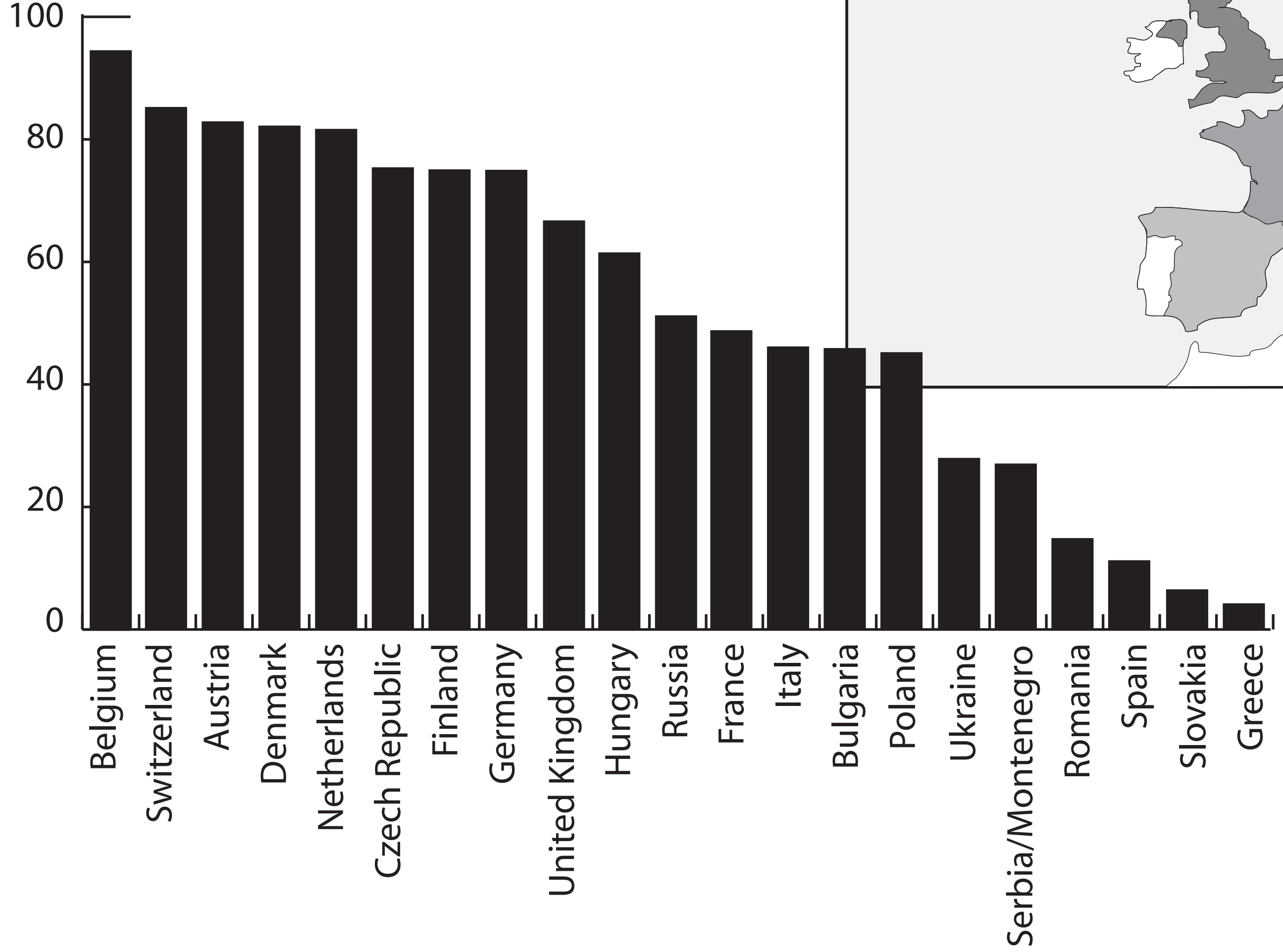

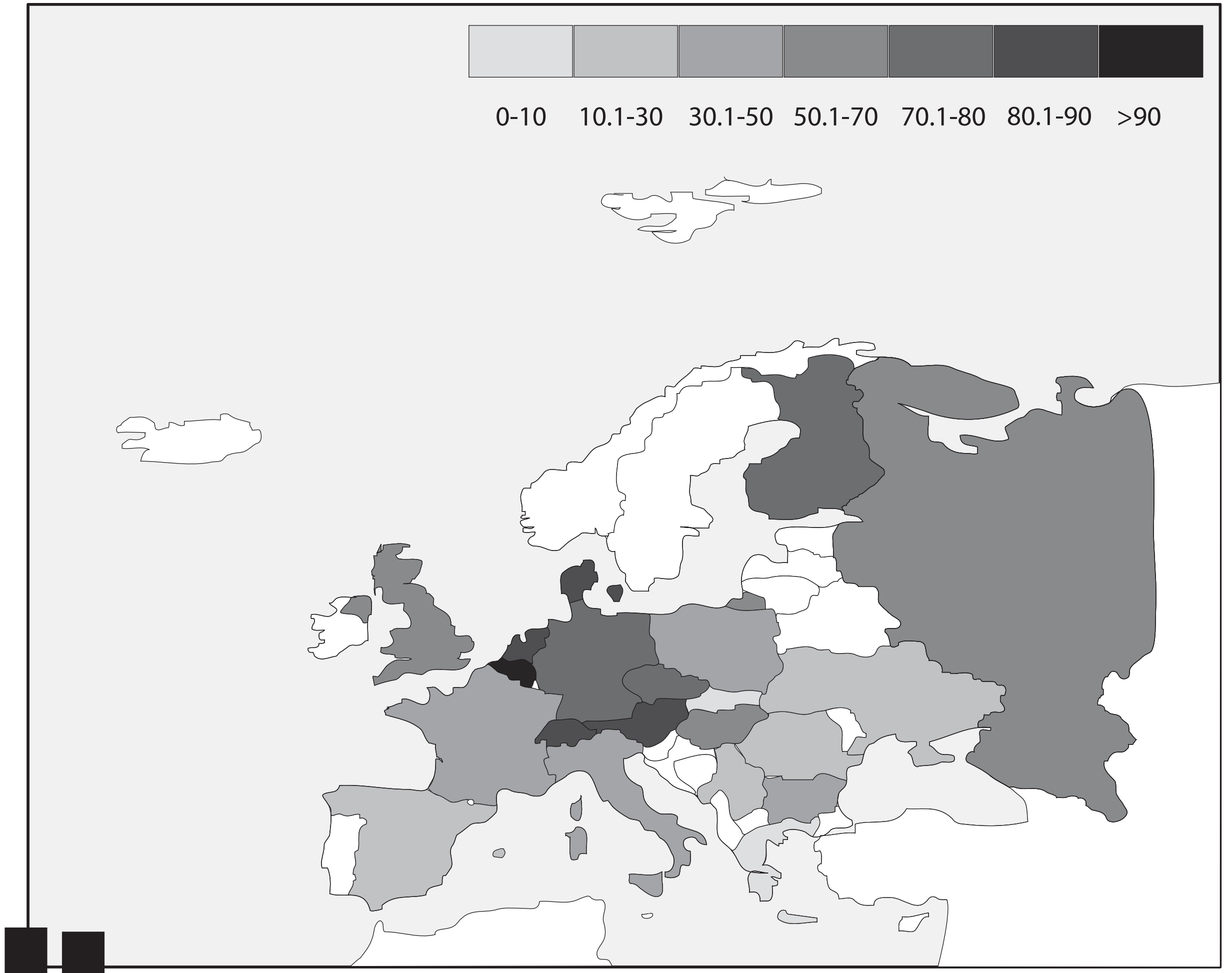

APPENDIX III: RAW DATA, 1998-2002

Table 6: Number of new species descriptions per country, 1998-2002. Data extracted from the Biosis dataset.

Albania	9
Andorra	8
Austria	88
Azores	3
Balearic Is.	13
Belarus	3
Belgium	4
Bosnia-Herzegovina	13
Bulgaria	96
Canary Is.	45
Corsica	27
Crete	51
Croatia	28
Cyprus	37
Czech Republic	55
Denmark	23
Estonia	7
Finland	23
France (without Corsica)	169
Germany	225
Greece (without Crete)	294
Hungary	55
Iceland	0
Ireland	3
Italy (without Sicily and Sardinia)	239
Latvia	3
Liechtenstein	0
Lithuania	10
Luxembourg	6
Macedonia	28
Madeira	13
Malta	17
Moldova	19
Netherlands	23
Norway	39
Poland	111
Portugal (without Madeira and the Azores)	43
Romania	36
Russia	142
San Marino	0
Sardinia	61
Serbia and Montenegro	62
Sicily	51
Slovakia	53
Slovenia	25
Spain (without Bakeares and Canary Is.)	502
Sweden	83
Switzerland	64
Turkey	3
Ukraine	157
United Kingdom	92
Vatican City	0

Table 7: Number of new species descriptions per country of descriptor, 1998-2002. Data extracted from the Biosis dataset.

Argentina	2	Malta	3
Australia	9	Mexico	1
Austria	134	Netherlands	87
Belarus	3	New Zealand	3
Belgium	36	Norway	9
Brazil	2	Poland	155
Bulgaria	48	Portugal	18
Canada	9	Republic of Ireland	3
China	2	Romania	27
Croatia	2	Russia	214
Czech Republic	150	Saudi Arabia	1
Denmark	85	Senegal	4
Estonia	3	Serbia and Montenegro	63
Finland	32	Slovakia	31
France	160	Slovenia	13
Germany	698	South Africa	3
Greece	24	Spain	251
Hungary	83	Sweden	11
India	1	Switzerland	54
Israel	6	Turkey	11
Italy	410	Ukraine	129
Latvia	3	United Kingdom	117
Lithuania	11	USA	24
Macedonia	4		

Table 8: Number of descriptors (first authors only) per country, 1998-2002. Data extracted from Biosis dataset.

Argentina	1	Macedonia	1
Australia	3	Malta	1
Austria	30	Mexico	1
Belarus	2	Netherlands	17
Belgium	14	New Zealand	1
Brazil	1	Norway	5
Bulgaria	20	Poland	37
Canada	2	Portugal	5
China	2	Romania	13
Croatia	2	Russia	63
Czech Republic	44	Saudi Arabia	1
Denmark	6	Senegal	1
Estonia	3	Serbia and Montenegro	14
Finland	10	Slovakia	7
France	61	Slovenia	8
Germany	124	South Africa	2
Greece	9	Spain	104
Hungary	17	Sweden	7
India	1	Switzerland	14
Ireland	1	Turkey	2
Israel	1	Ukraine	24
Italy	120	United Kingdom	34
Latvia	1	USA	13
Lithuania	2		

Table 9: % of new species described by taxonomists working in the species country, 1998-2002. Data extracted from the Biosis dataset.

Note that in Figure 49, countries where 10 or less species were described have been excluded (for instance, 9 species only were described from Albania: Albania was excluded from the graph). These countries are in italics in this table.

Albania	*0.0*	*Luxembourg*	*0.0*
Andorra	*0.0*	Macedonia	7.1
Austria	26.1	Malta	11.8
Belarus	*66.7*	Moldova	0.0
Belgium	*50.0*	Netherlands	69.6
Bosnia-Herzegovina	0.0	Norway	7.7
Bulgaria	27.1	Poland	76.6
Croatia	7.1	Portugal	20.3
Cyprus	0.0	*Republic of Ireland*	*33.3*
Czech Republic	67.3	Romania	63.9
Denmark	68.2	Russia	79.4
Estonia	*28.6*	Serbia and Montenegro	74.2
Finland	34.8	Slovakia	54.7
France	41.8	Slovenia	32.0
Germany	79.7	Spain	42.1
Greece	6.7	Sweden	4.8
Hungary	58.2	Switzerland	12.5
Italy	63.0	*Turkey*	*50.0*
Latvia	*33.3*	Ukraine	59.2
Lithuania	*100.0*	United Kingdom	42.4

Table 10: For each affiliation country, % of new species described from countries different from affiliation country, 1998-2002. Data extracted from the Biosis dataset.

Note that in Figure 50, affiliation countries where taxonomists described less than 20 species have been excluded (for instance, 13 species only were described by taxonomists working in Slovenia: Slovenia was excluded from the graph). These countries are in italics in this table.

Austria	82.8	*Malta*	*33.3*
Belarus	*33.3*	Netherlands	81.6
Belgium	94.4	*Norway*	*66.7*
Bulgaria	45.8	Poland	45.2
Croatia	*0.0*	*Portugal*	*33.3*
Czech Republic	75.3	*Republic of Ireland*	*66.7*
Denmark	82.1	Romania	14.8
Estonia	*33.3*	Russia	51.2
Finland	75.0	Serbia and Montenegro	27.0
France	48.8	Slovakia	6.5
Germany	74.9	*Slovenia*	*38.5*
Greece	4.2	Spain	11.2
Hungary	61.4	*Sweden*	*63.6*
Italy	46.1	Switzerland	85.2
Latvia	*66.7*	*Turkey*	*90.9*
Lithuania	*9.1*	Ukraine	27.9
Macedonia	*50.0*	United Kingdom	66.7

APPENDIX IV: PARIS MEETING PRESENTATION

This appendix shows the presentation that was given at the Fauna Europaea Paris meeting, 25-27 September 2004.

Fauna Europaea
What remains to be discovered, where and by whom?

Gap analysis (WP 12)

Philippe Bouchet
Benoît Fontaine

Muséum national d'Histoire naturelle, Paris

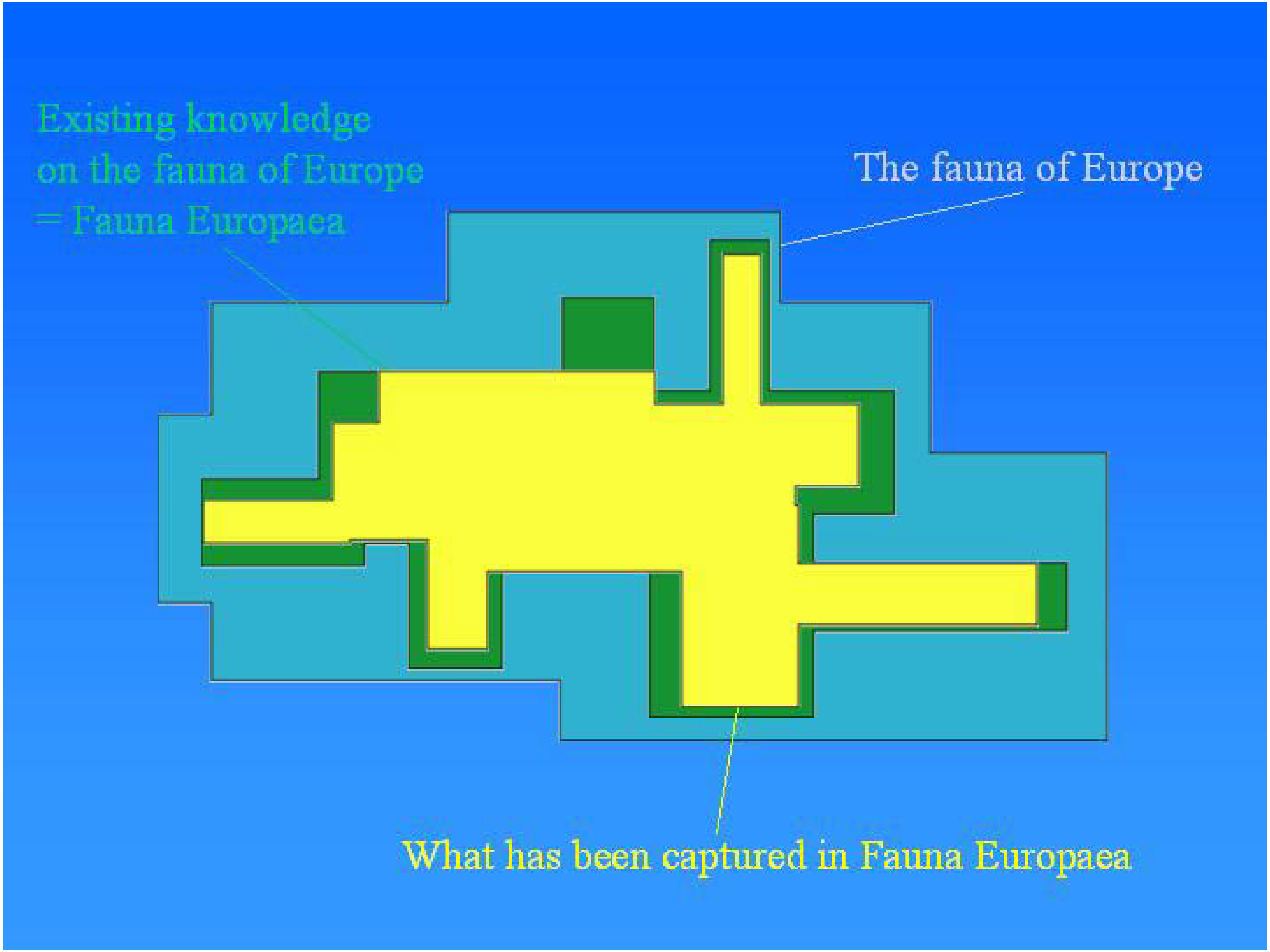
Existing knowledge
on the fauna of Europe
= Fauna Europaea
The fauna of Europe
What has been captured in Fauna Europaea

Material:

- Fauna Europaea data (as of 10 September 2004 - 117311 sp)

- Database provided by Biosis: species described in 1998-2003 (3161 sp)

- Random selection of ca. 10% of the above (338 sp)

Analysis performed

Fauna Europaea data

Increase rate of the taxonomic inventory in Europe

- per major taxa
- per country

Analysis performed

Species described in 1998-2003

- Taxonomic distribution of new species
- Geographical origin of new species
- Country of descriptors
- Inbreeding/outbreeding of national expertise
- « Top 10 » Fauna Europaea species

Analysis performed

Random selection of 10%

- Exact location of type localities
- Impact of new technologies
- Professionnal status of descriptors
- Access to primary litterature

The state of the art

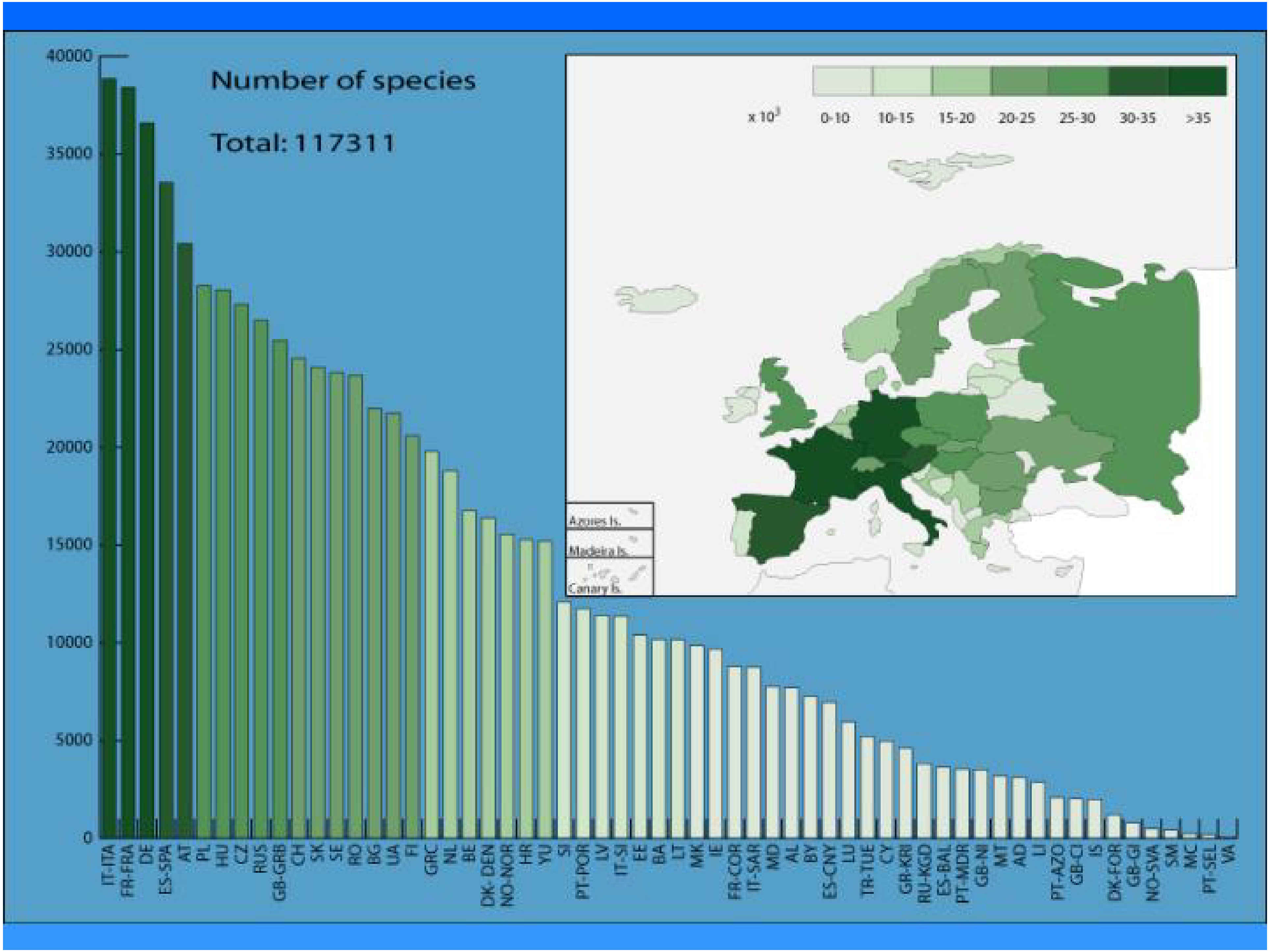
Number of species
Total: 117311
40000
35000
30000
25000
20000
15000
10000
5000
0
IT-ITA
FR-FRA
DE
ES-SPA
AT
PL
HU
CZ
RUS
GB-GRB
CH
SK
SE
RO
BG
UA
FI
GRC
NL
BE
DK-DEN
NO-NOR
HR
YU
SI
PT-POR
LV
IT-SI
EE
BA
LT
MK
IE
FR-COR
IT-SAR
MD
AL
BY
ES-CNY
LU
TR-TUE
CY
GR-KRI
RU-KGD
ES-BAL
PT-MDR
GB-NI
MT
AD
LI
PT-AZO
GB-CI
IS
DK-FOR
GB-GI
NO-SVA
SM
MC
PT-SEL
VA
x 10³
0-10
10-15
15-20
20-25
25-30
30-35
>35
Azores Is.
Madeira Is.
Canary Is.

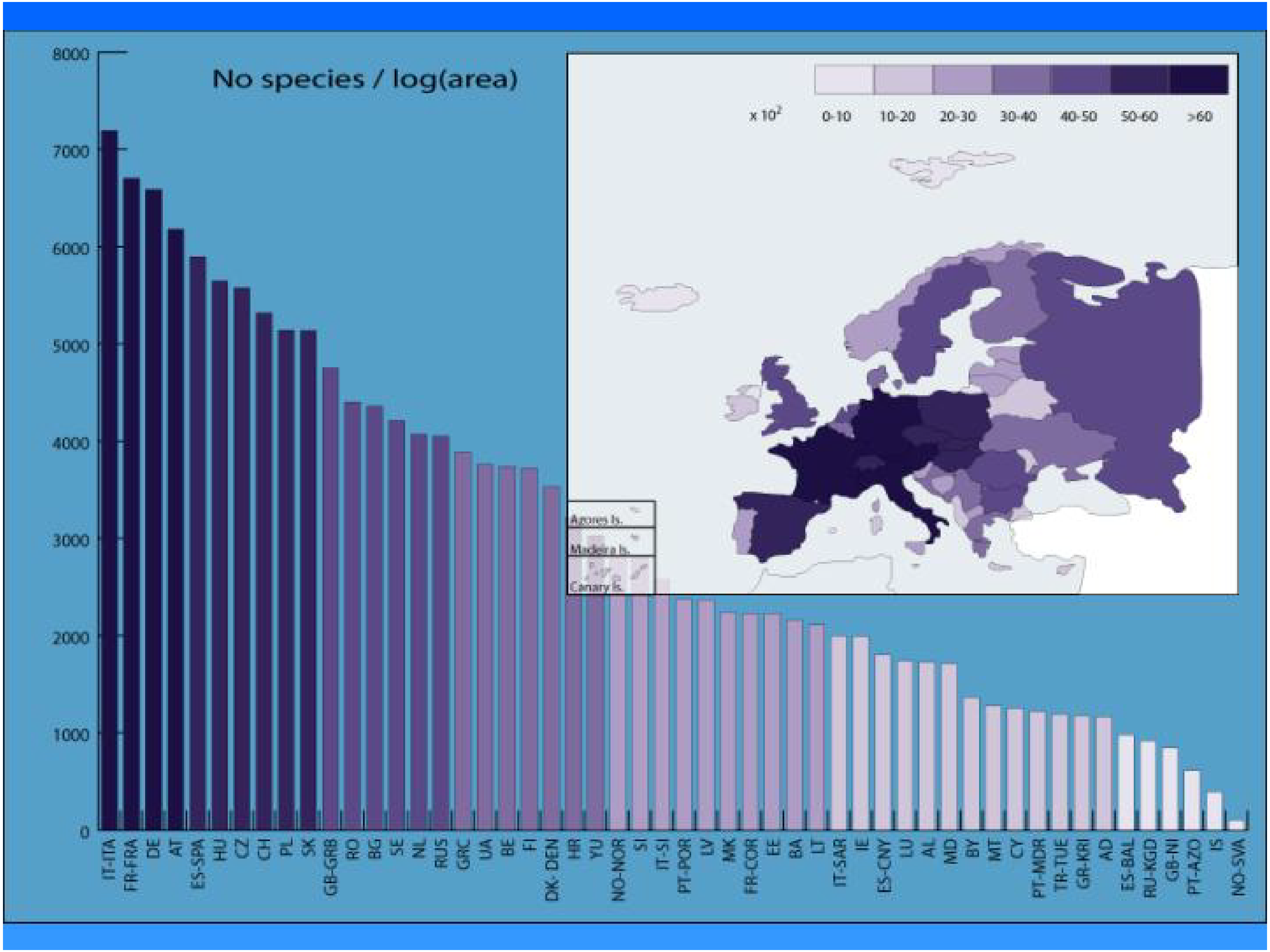

No species / log(area)
8000
7000
6000
5000
4000
3000
2000
1000
0
x 10²
0-10
10-20
20-30
30-40
40-50
50-60
>60
Azores Is.
Madeira Is.
Canary Is.
IT-ITA
FR-FRA
DE
AT
ES-SPA
HU
CZ
CH
PL
SK
GB-GRB
RO
BG
SE
NL
RUS
GRC
UA
BE
FI
DK- DEN
HR
YU
NO-NOR
SI
IT-SI
PT-POR
LV
MK
FR-COR
EE
BA
LT
IT-SAR
IE
ES-CNY
LU
AL
MD
BY
MT
CY
PT-MDR
TR-TUE
GR-KRI
AD
ES-BAL
RU-KGD
GB-NI
PT-AZO
IS
NO-SVA

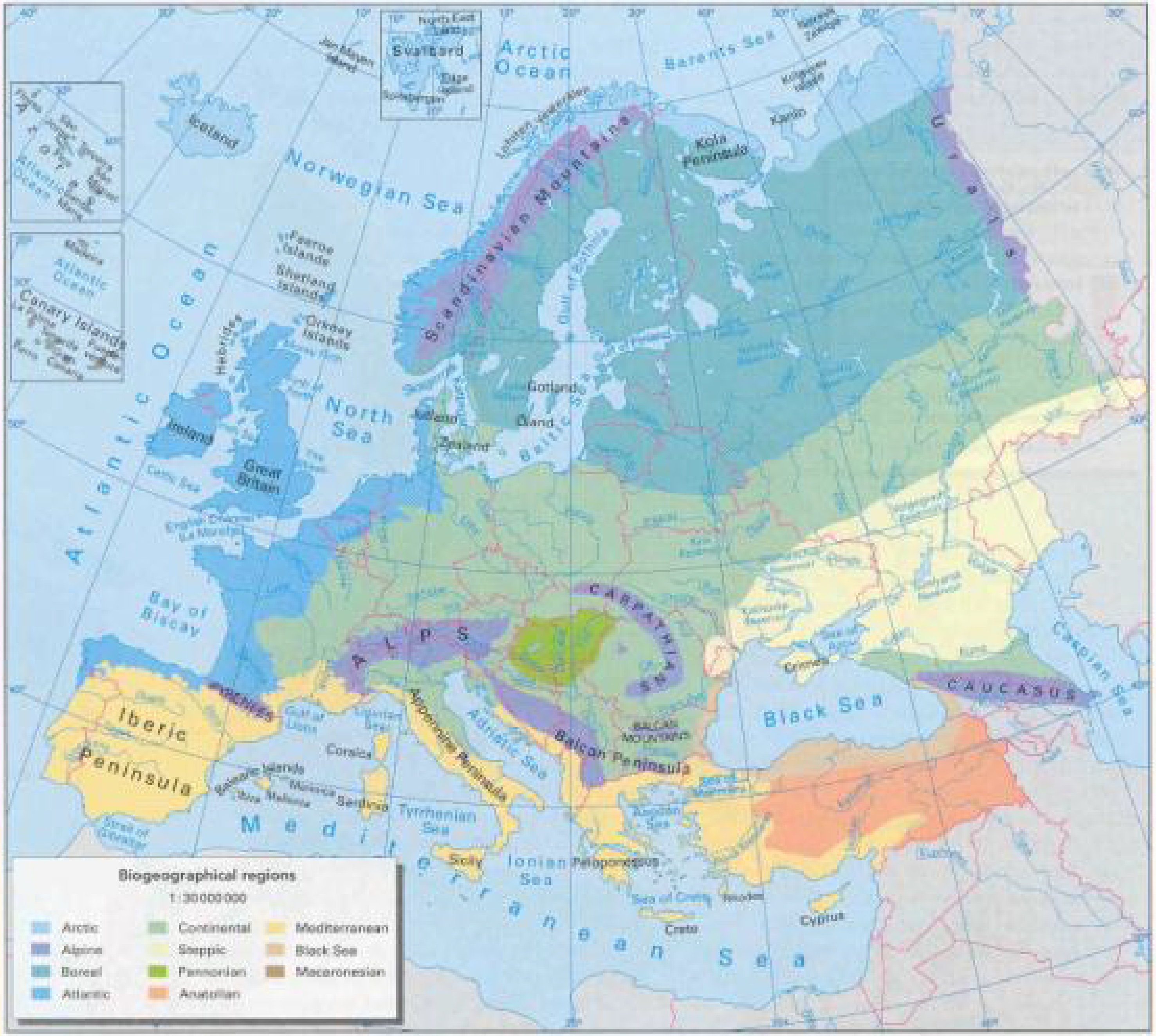

Source : CEC DG XI, Council of Europe, 1997

What remains to be discovered?

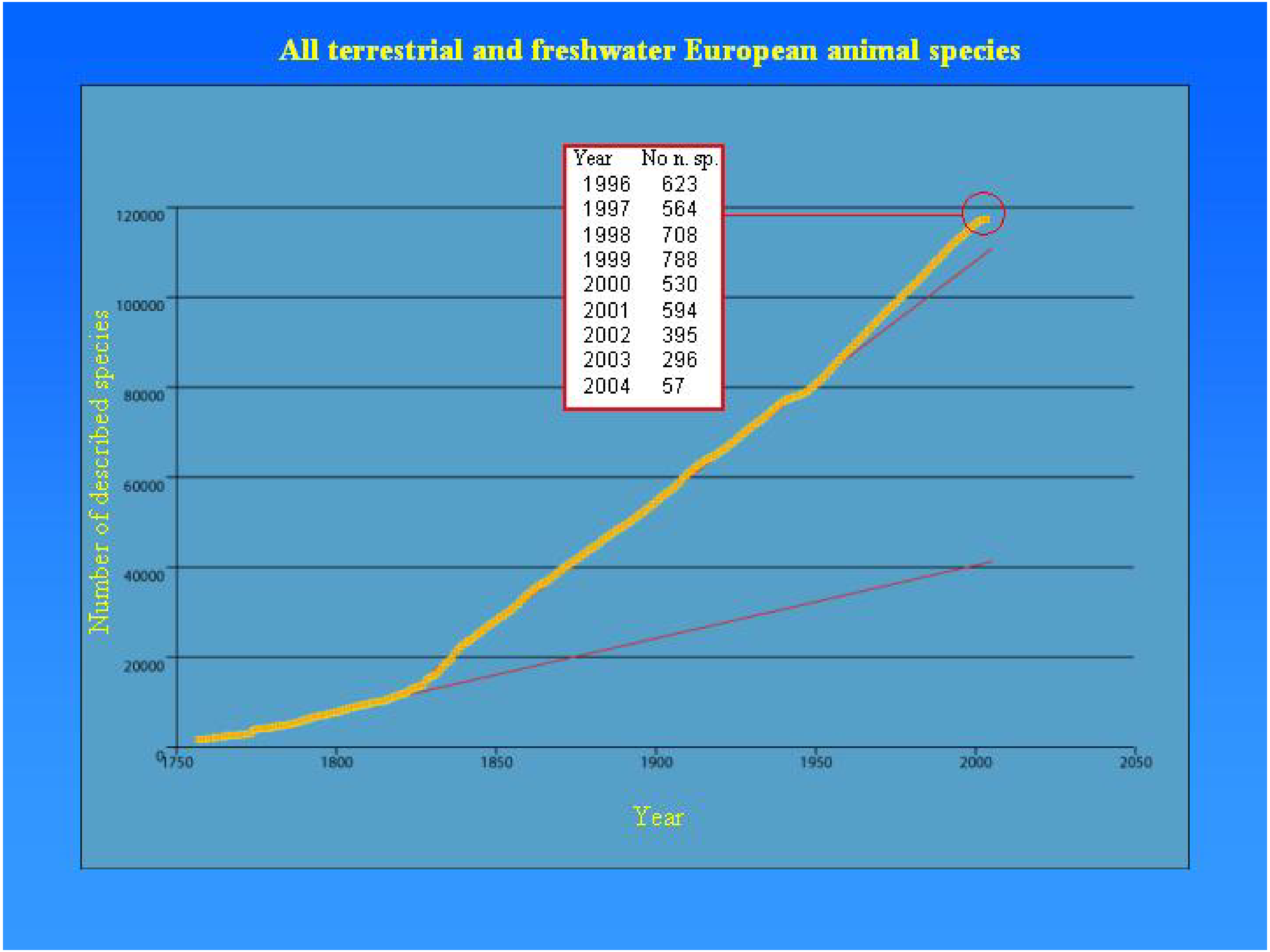
All terrestrial and freshwater European animal species
Number of described species
120000
100000
80000
60000
40000
20000
0
1750
1800
1850
1900
1950
2000
2050
Year
Year No n. sp.
1996 623
1997 564
1998 708
1999 788
2000 530
2001 594
2002 395
2003 296
2004 57

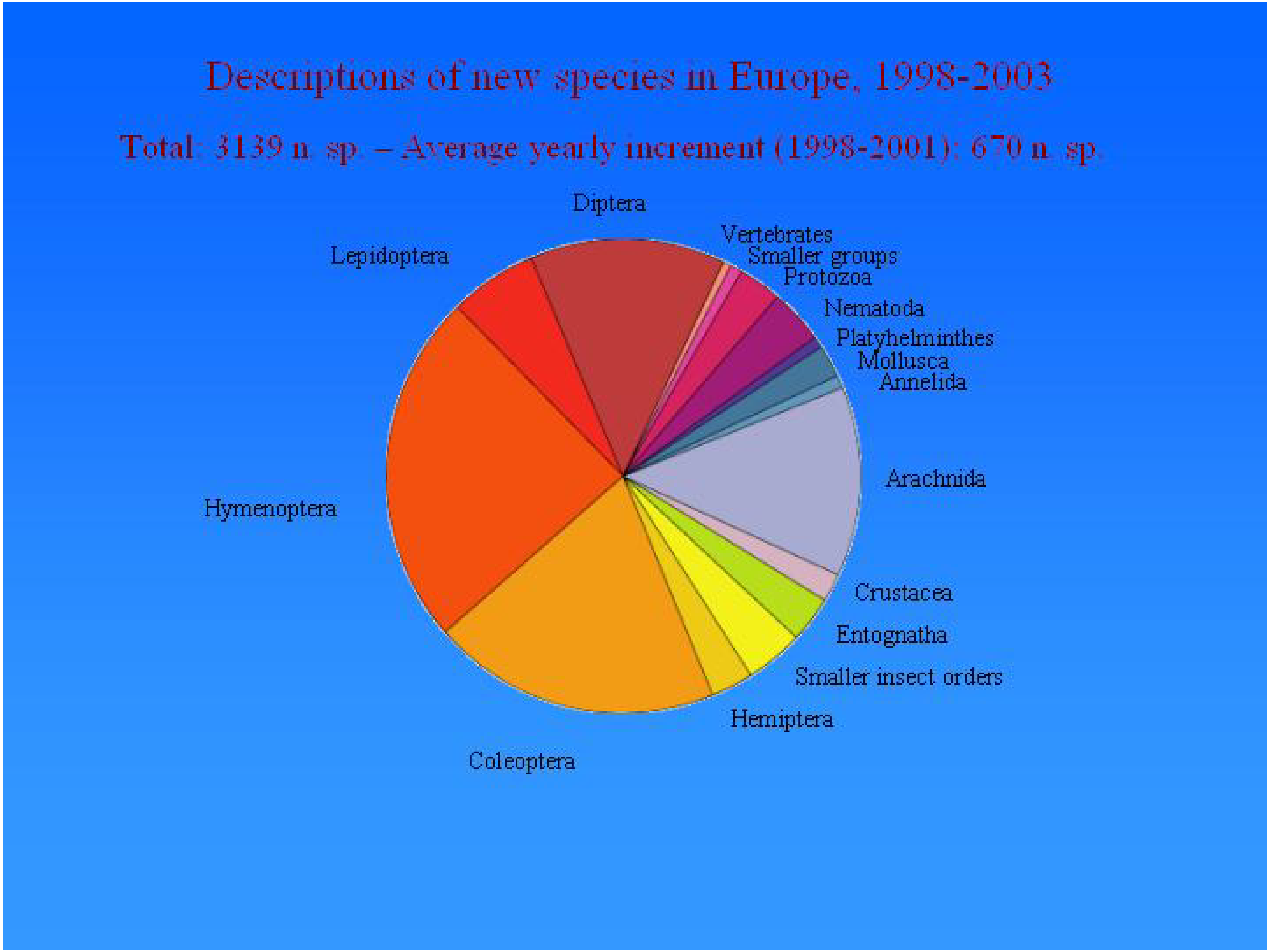
Descriptions of new species in Europe, 1998-2003
Total: 3139 n. sp. – Average yearly increment (1998-2001): 670 n. sp.
Diptera
Vertebrates
Smaller groups
Protozoa
Nematoda
Platyhelminthes
Mollusca
Annelida
Arachnida
Crustacea
Entognatha
Smaller insect orders
Hemiptera
Coleoptera
Hymenoptera
Lepidoptera

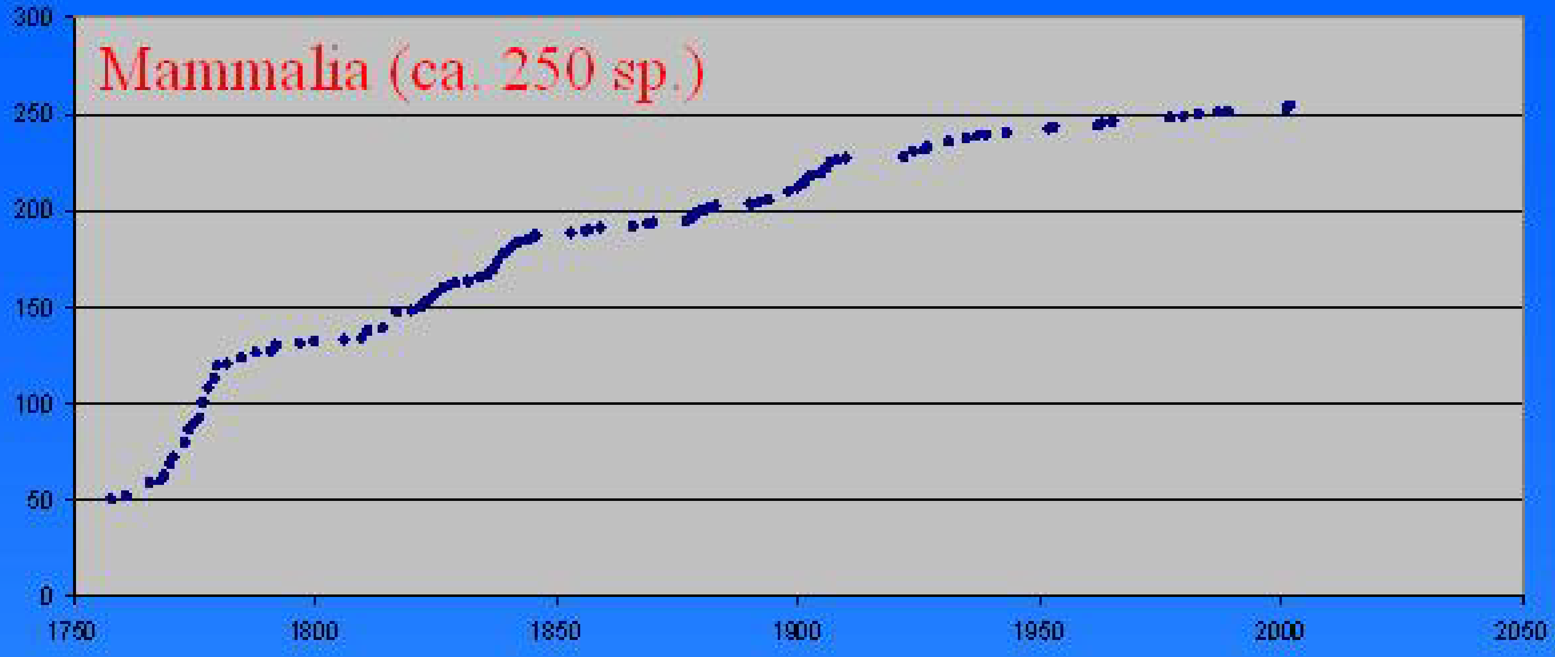
Mammalia (ca. 250 sp.)
300
250
200
150
100
50
0
1750
1800
1850
1900
1950
2000
2050

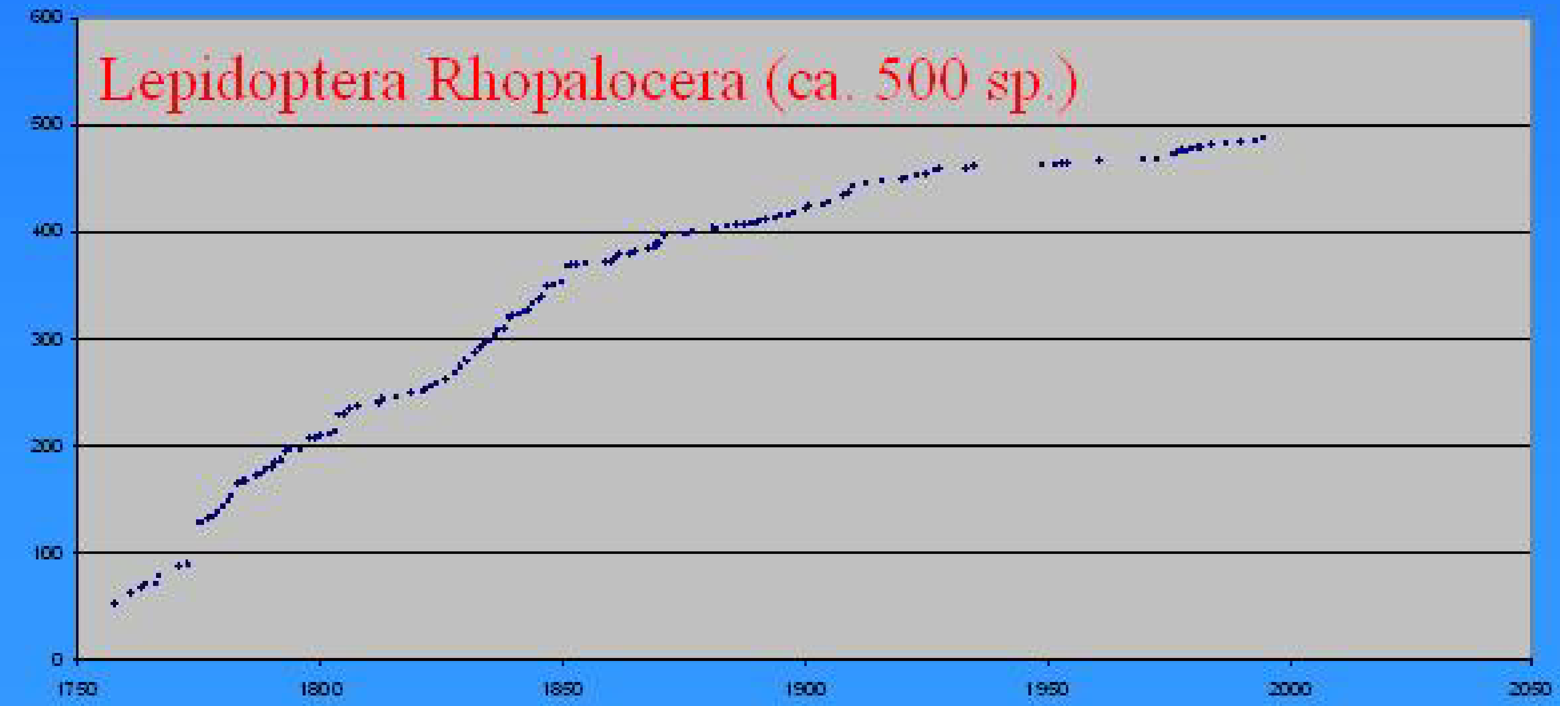
Lepidoptera Rhopalocera (ca. 500 sp.)
600
500
400
300
200
100
0
1750
1800
1850
1900
1950
2000
2050

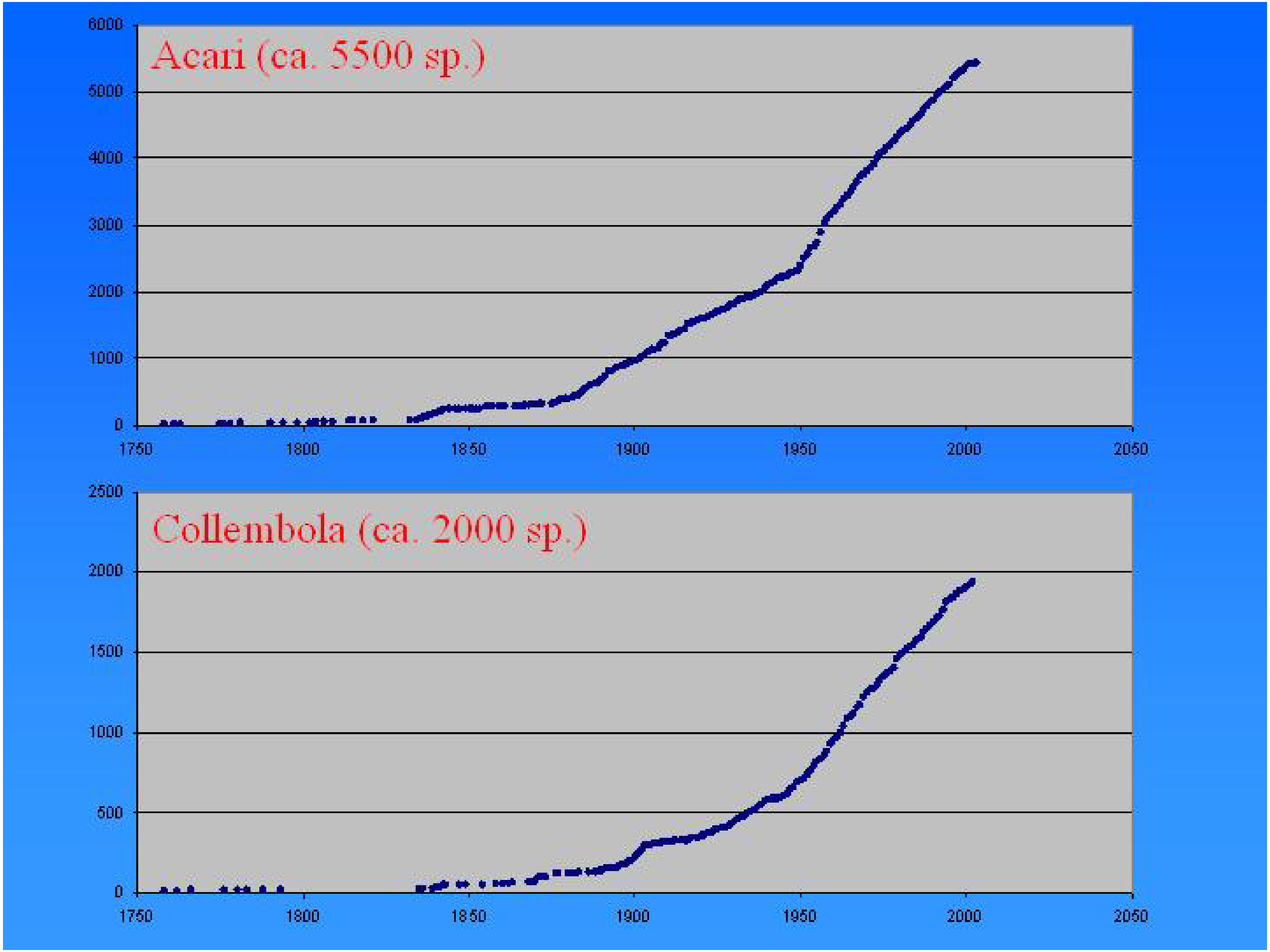
Acari (ca. 5500 sp.)
6000
5000
4000
3000
2000
1000
0
1750
1800
1850
1900
1950
2000
2050
Collembola (ca. 2000 sp.)
2500
2000
1500
1000
500
0
1750
1800
1850
1900
1950
2000
2050

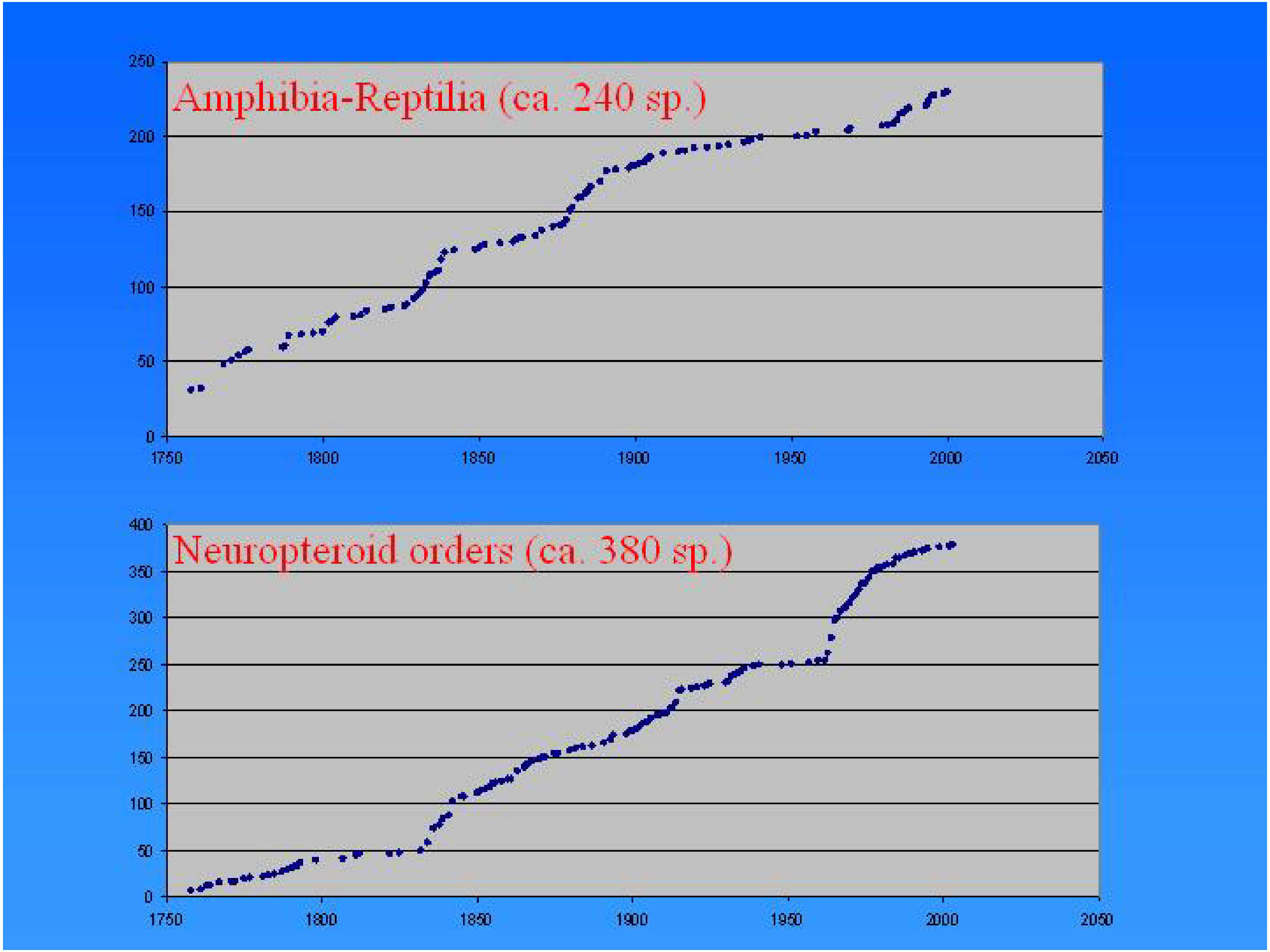
Amphibia-Reptilia (ca. 240 sp.)
250
200
150
100
50
0
1750
1800
1850
1900
1950
2000
2050
Neuropteroid orders (ca. 380 sp.)
400
350
300
250
200
150
100
50
0
1750
1800
1850
1900
1950
2000
2050

Top 10 Fauna Europaea species

Myotis alcathoe von Helversen & Heller, 2000

- Bat, Greece
- Cryptic species, description based on voice, morphology, karyotype and DNA
- Recorded in Switzerland, Hungary and Greece
- Habitat threatened

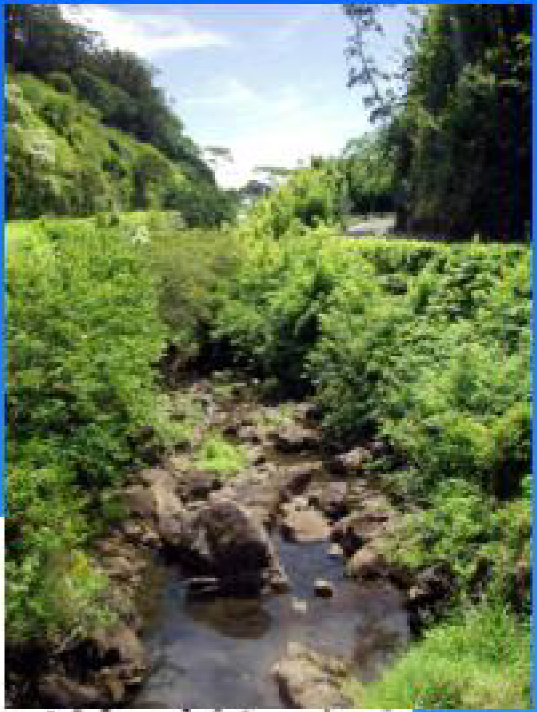

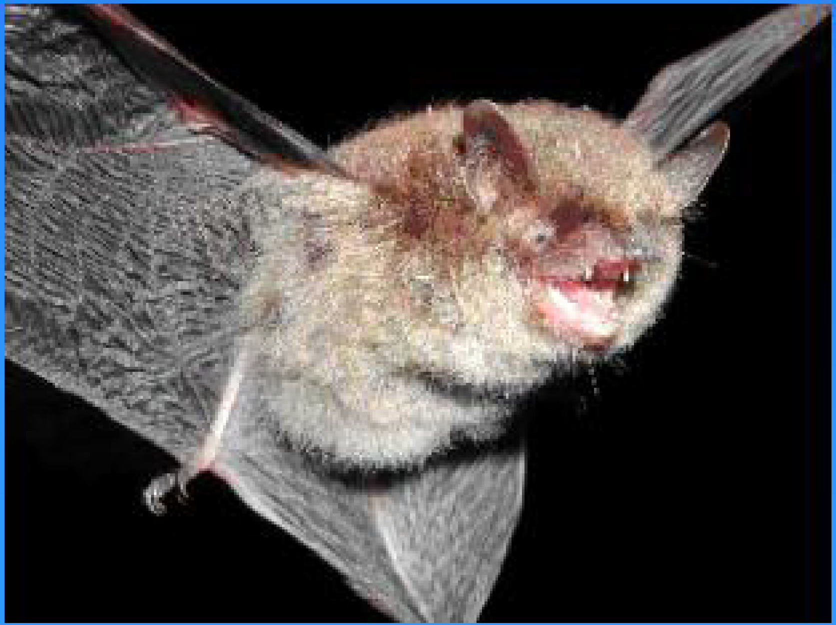

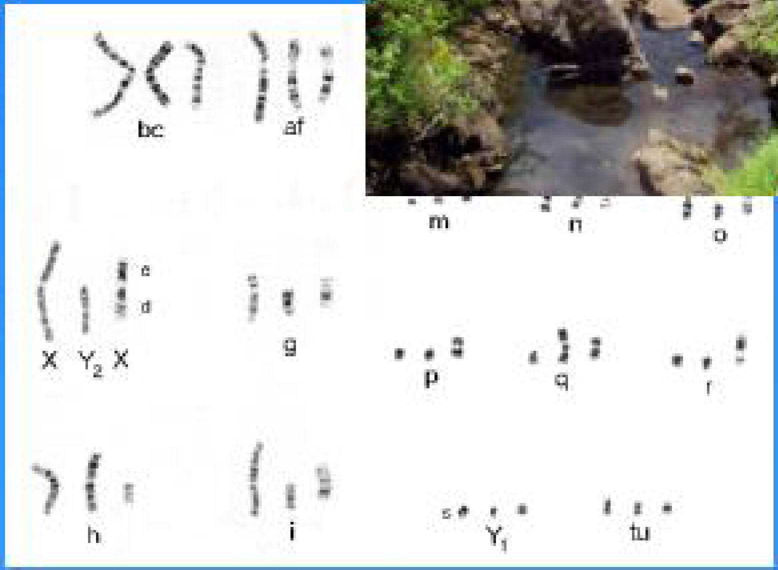

Top 10 Fauna Europaea species

Chorthippus jutlandicus Nielsen, 2003

- Grasshopper, Denmark
- "Well-known" group in a "well-explored" country
- Description based on song and morphology

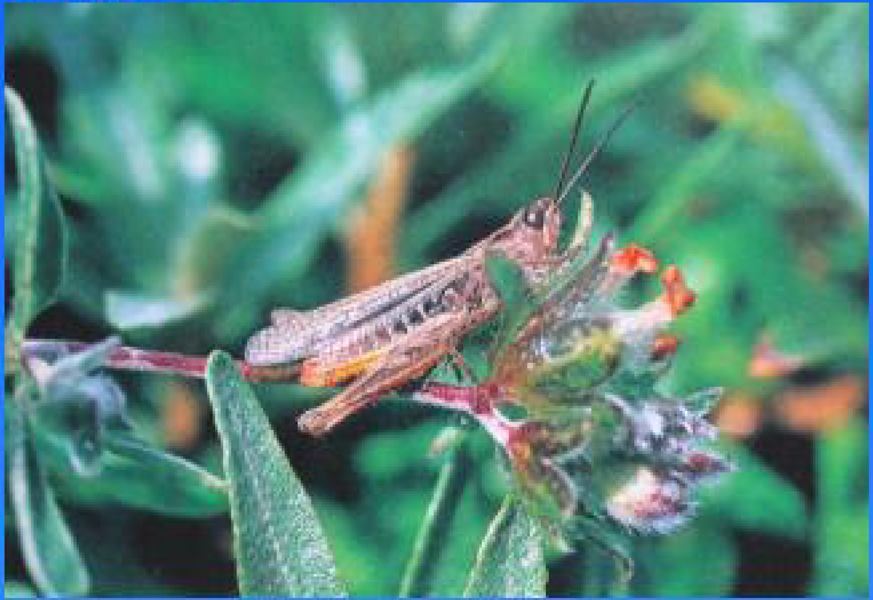

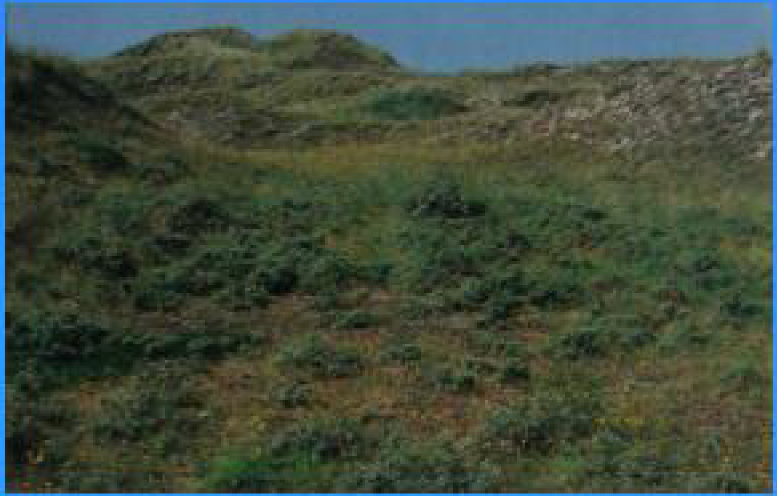

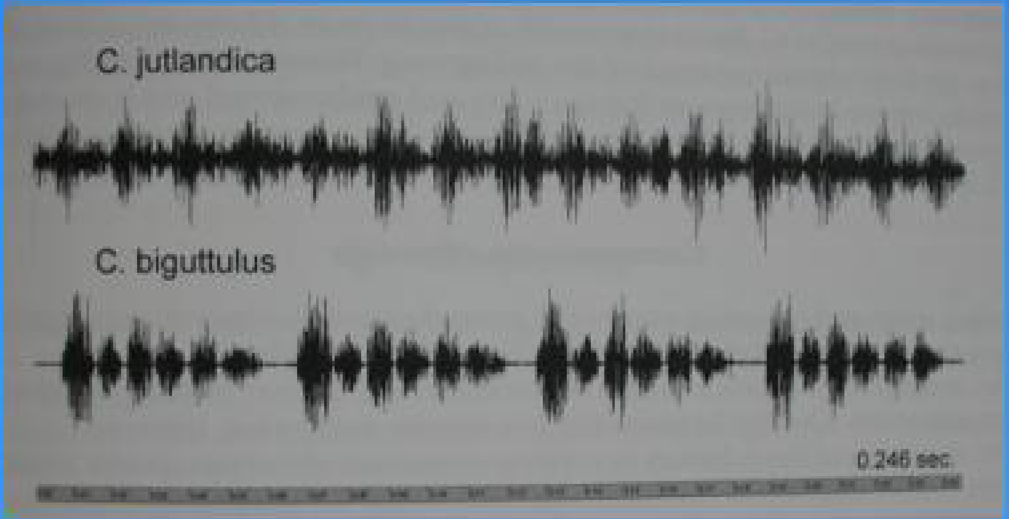

Top 10 Fauna Europaea species

Gyrodactylus teuchis Lautraite, Blanc, Thiery, Daniel & Vigneulle, 1999

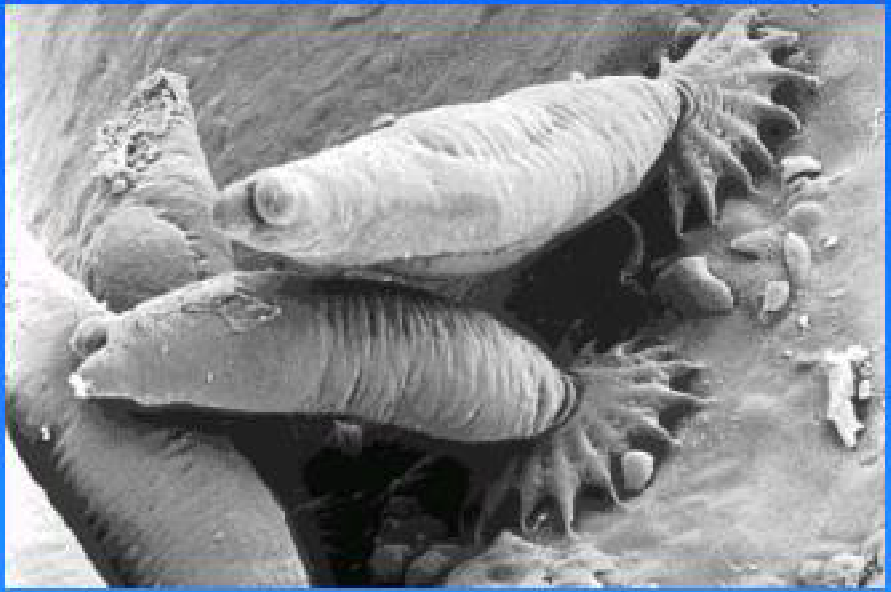

- Monogene parasite from salmon, France
- Description based on non-morphological characters
- Economic importance

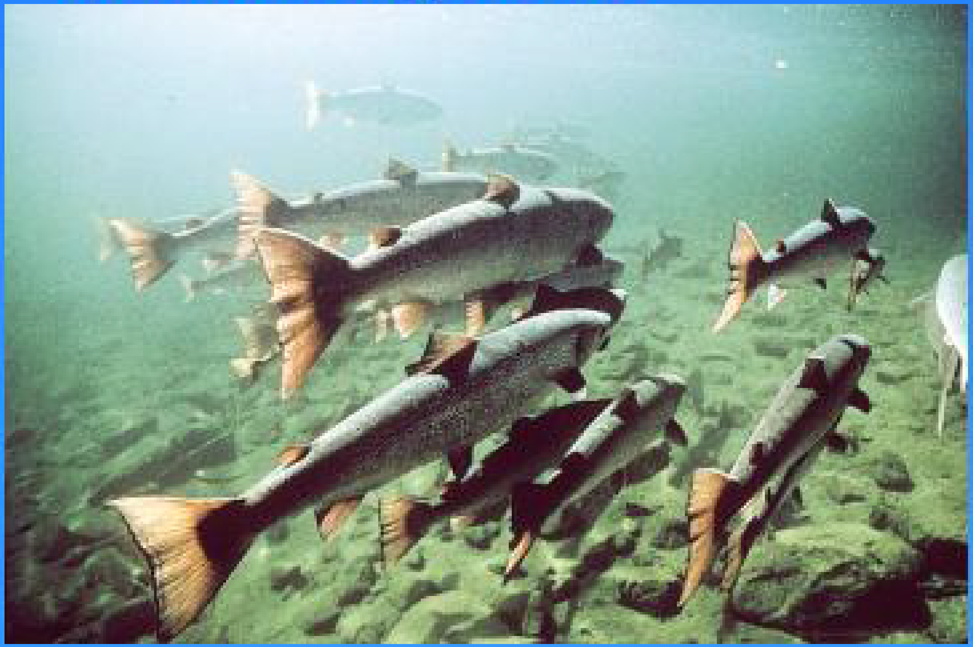

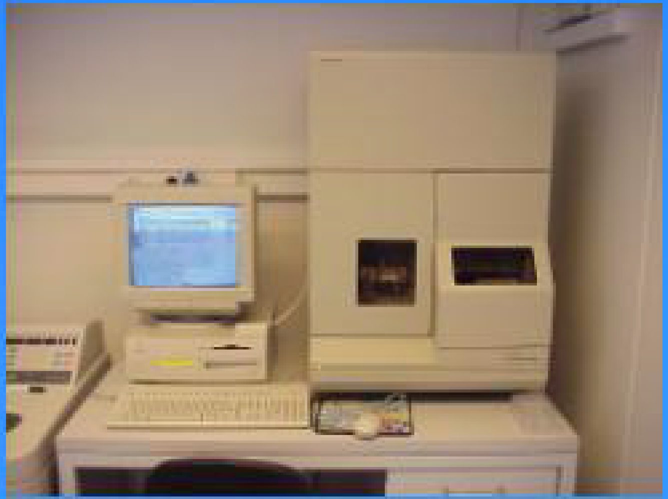

Top 10 Fauna Europaea species

Metaphycus hageni Daane & Caltagrione, 1999

- Hymenoptera, Spain
- Economic importance, used in biocontrol
- Introduced to California

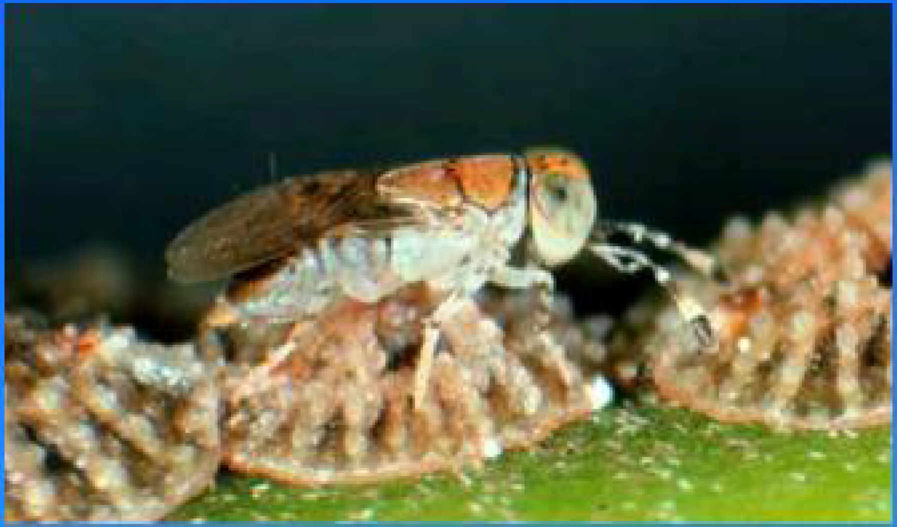

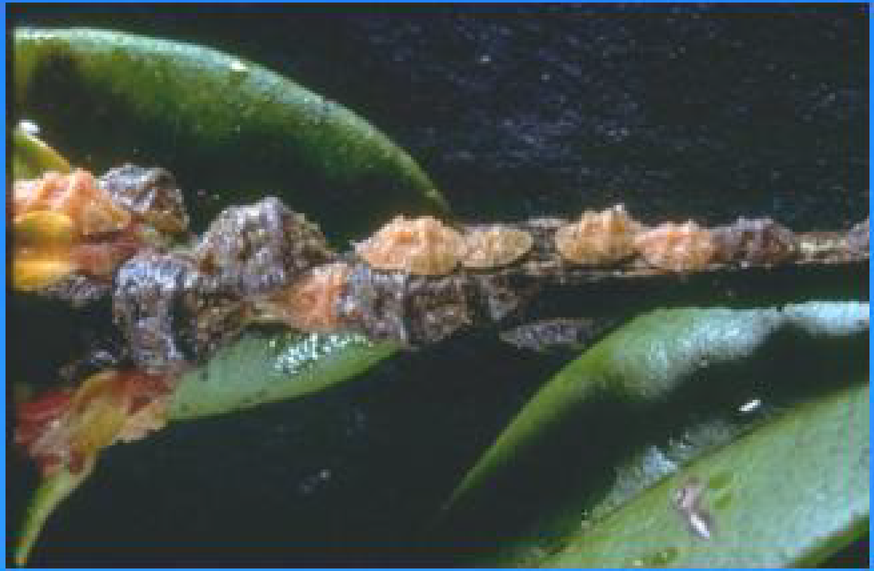

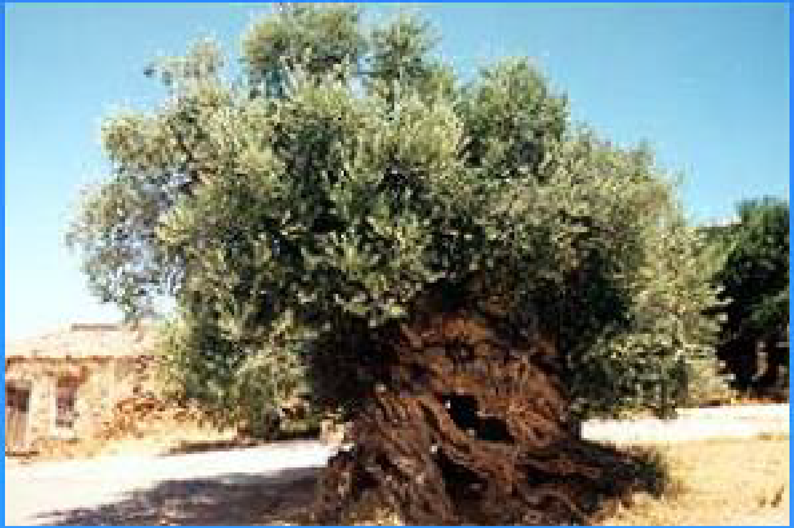

Top 10 Fauna Europaea species

Croatobranchus mestrovi Kerovec, Kucini & Jalzic, 1999

- Cave leech, Croatia
- New frontier for biodiversity exploration. > 1000 m deep cave
- Description in Croatian

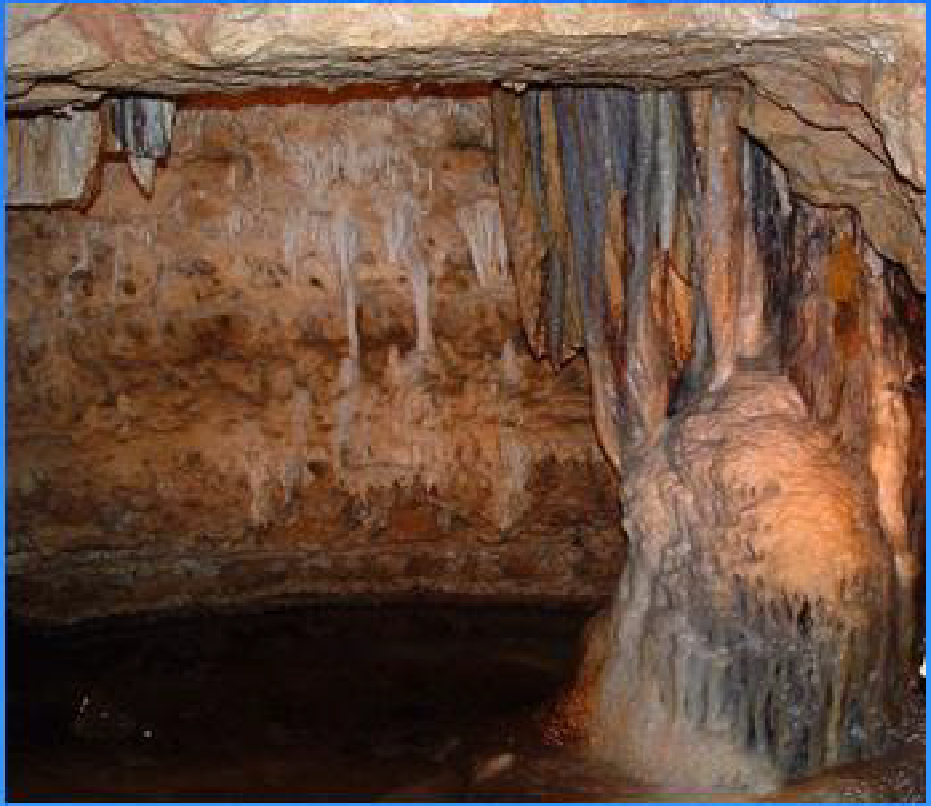

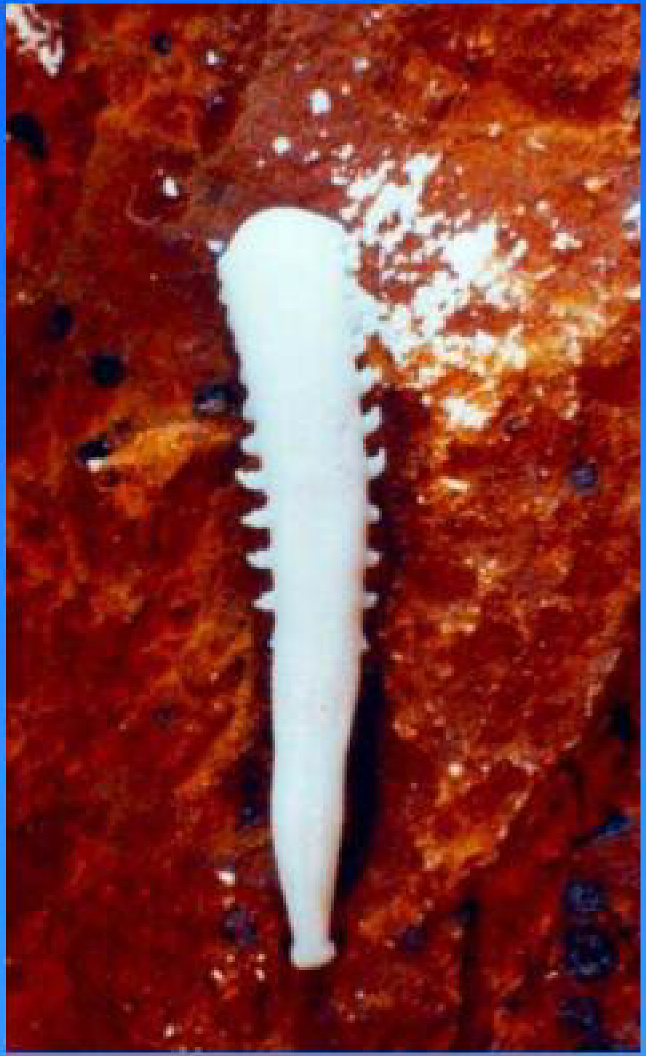

Top 10 Fauna Europaea species

Arthurdendyus albidus Jones & Gerard, 1999

•Turbellaria, Scotland, domestic garden

•Stygofauna of humic terrestrial biotopes more or less unknown in Europe

•Could be native to NZ

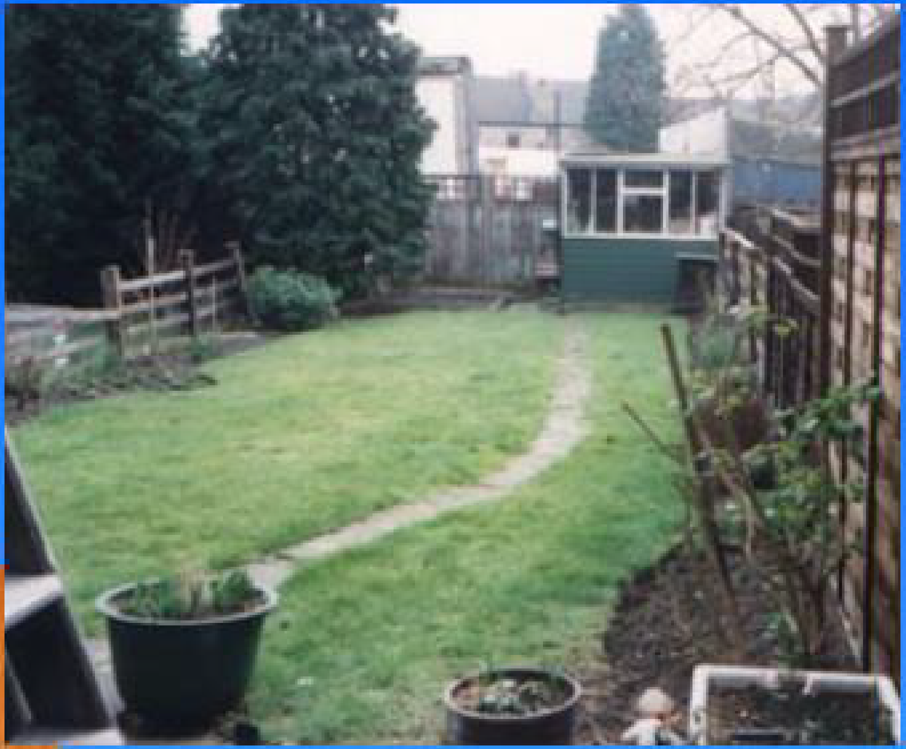

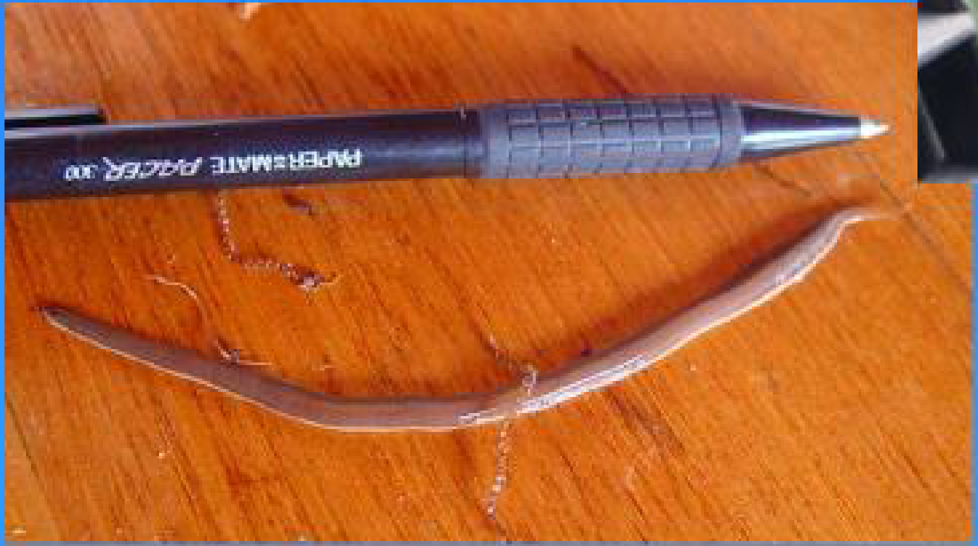

Top 10 Fauna Europaea species

Chvalaea sopianae Papp & Földvari, 2001

- Diptera, Hungary
- Discovery based on organized funded research (Project "Large Blank Spots in the Diptera Fauna of Hungary"), >1,000,000 adult flies captured)
- New genus – Second *Chvalaea* species discovered in Taiwan.

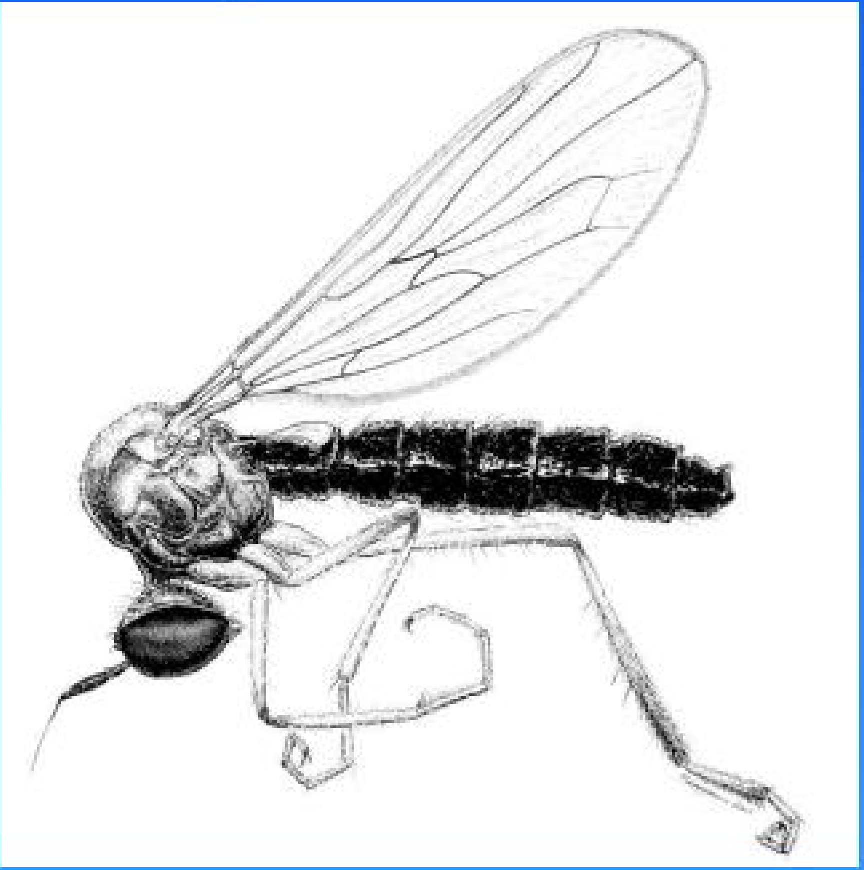

Top 10 Fauna Europaea species

Ethmia mariannae Karsholt & Kun, 2003

- Lepidoptera, Rhodes
- Islands fauna

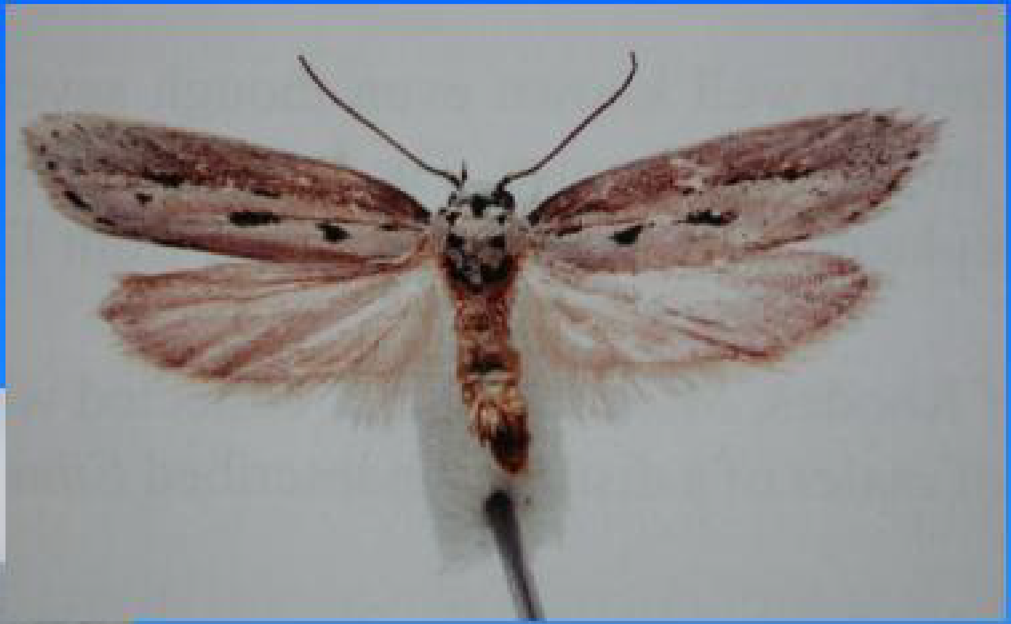

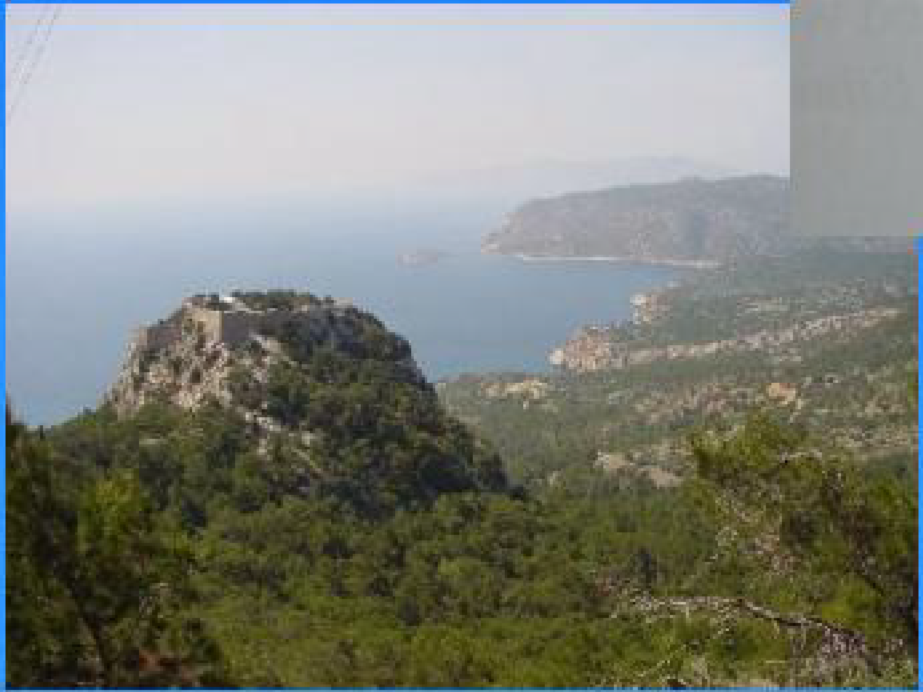

Top 10 Fauna Europaea species

Chiloplacus maginensis Abolafia & Pena-Santiago, 2003

- Nematoda, Spain
- bacterial-feeding
- Long-term government-funded research program: Fauna Iberica

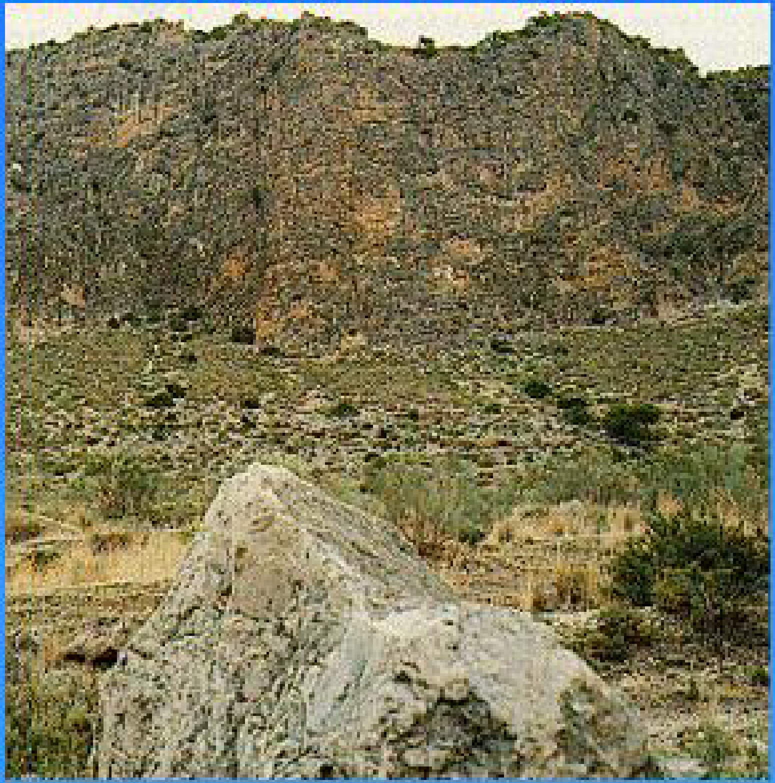

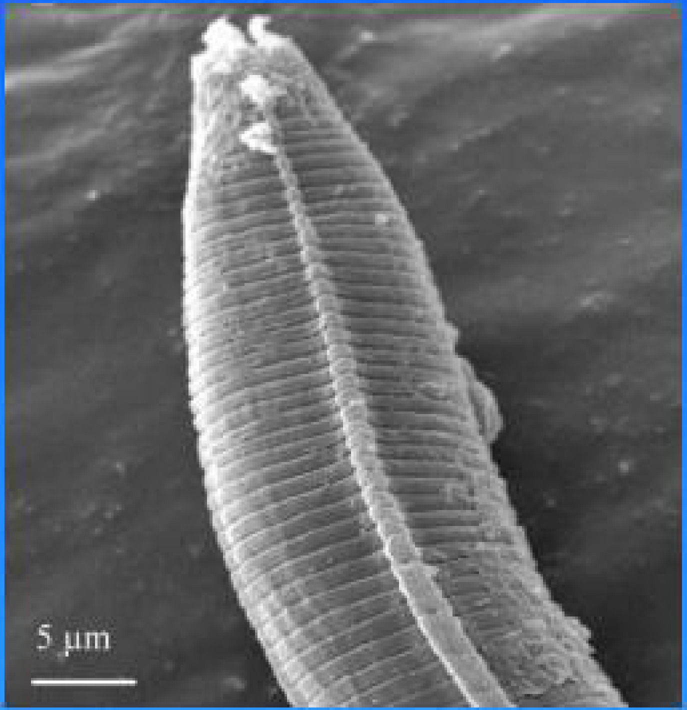

Top 10 Fauna Europaea species

Meligethes salvan Audisio, de Biase & Antonini, 2003

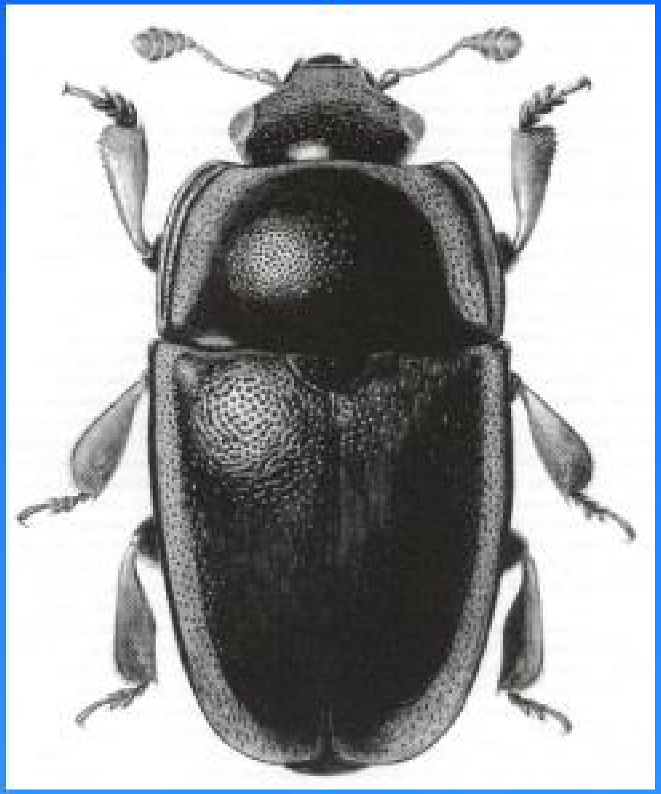

- Coleoptera, Italy
- Collected in 1912 – Never found again
- Area now destroyed - Importance of museum collections

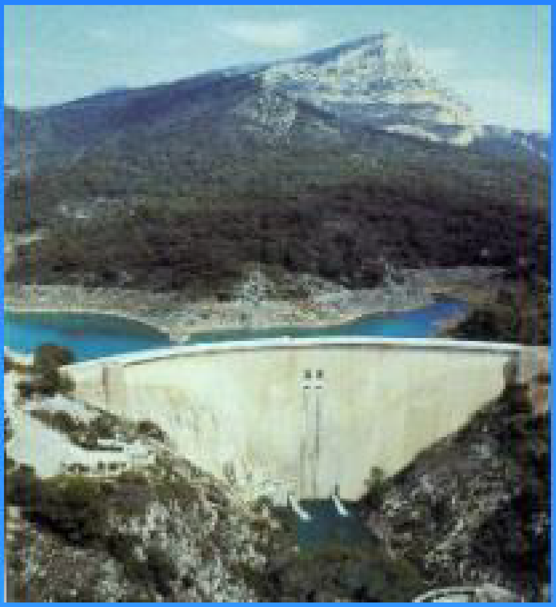

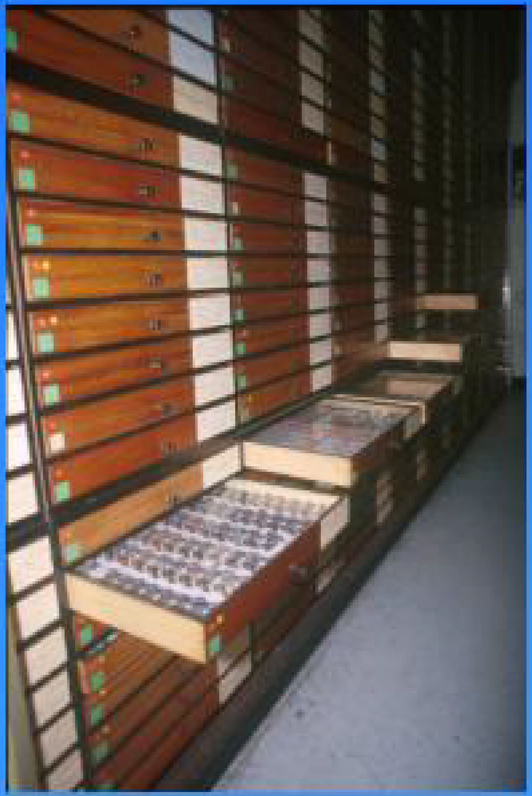

Where?

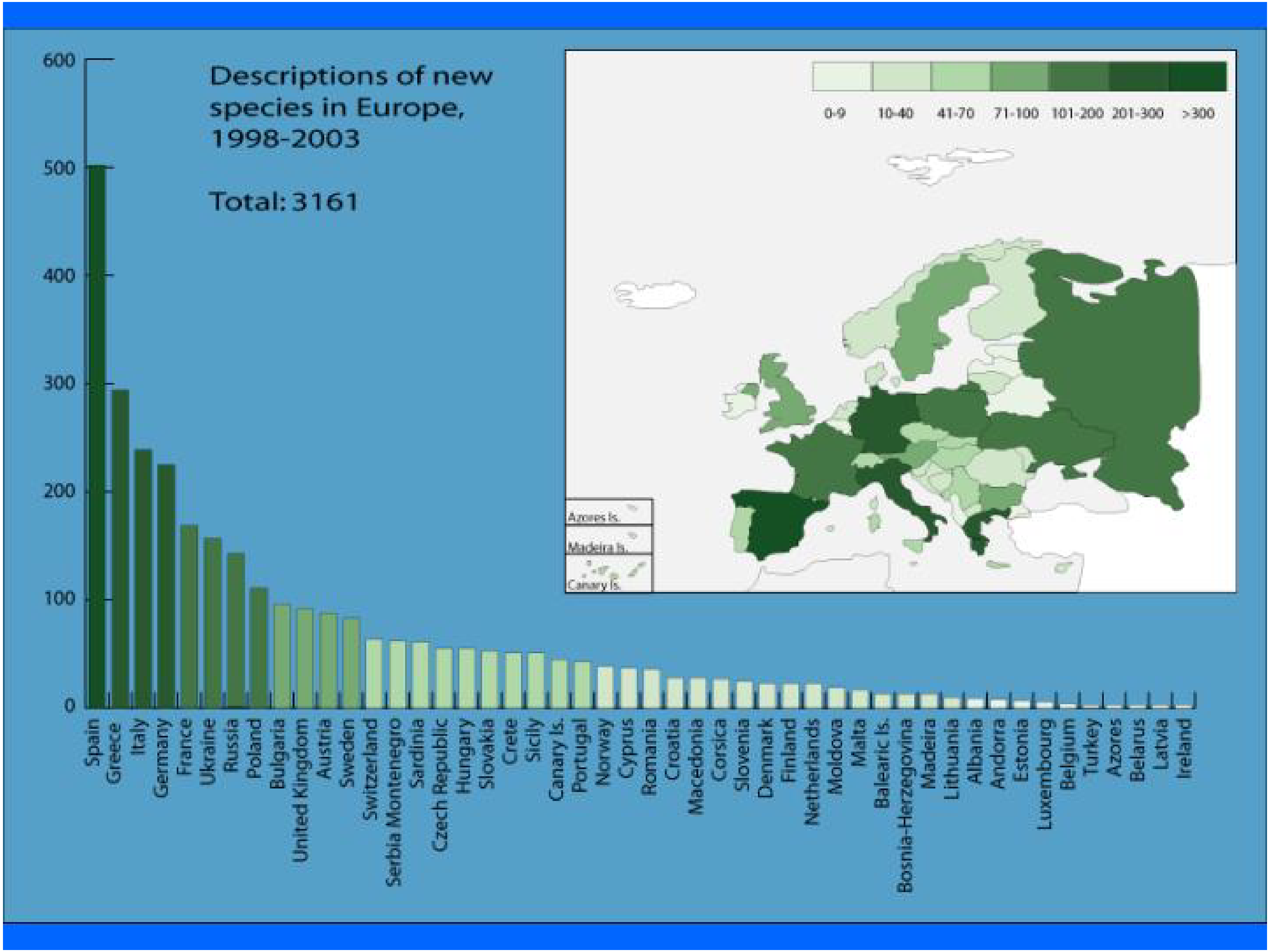
Descriptions of new species in Europe, 1998-2003
Total: 3161
600
500
400
300
200
100
0
Spain
Greece
Italy
Germany
France
Ukraine
Russia
Poland
Bulgaria
United Kingdom
Austria
Sweden
Switzerland
Serbia Montenegro
Sardinia
Czech Republic
Hungary
Slovakia
Crete
Sicily
Canary Is.
Portugal
Norway
Cyprus
Romania
Croatia
Macedonia
Corsica
Slovenia
Denmark
Finland
Netherlands
Moldova
Malta
Balearic Is.
Bosnia-Herzegovina
Madeira
Lithuania
Albania
Andorra
Estonia
Luxembourg
Belgium
Turkey
Azores
Belarus
Latvia
Ireland
0-9
10-40
41-70
71-100
101-200
201-300
>300
Azores Is.
Madeira Is.
Canary Is.

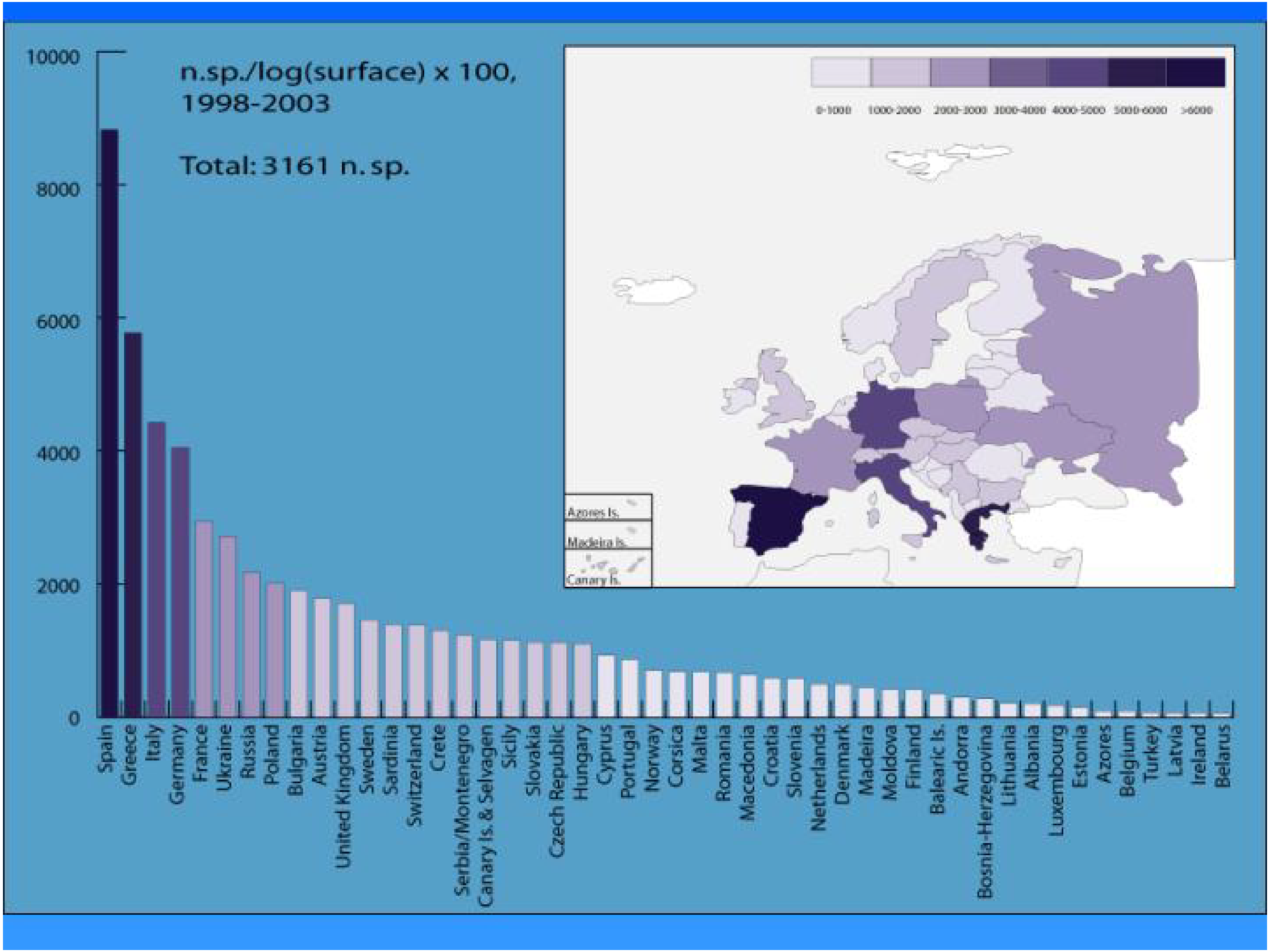
n.sp./log(surface) x 100, 1998-2003
Total: 3161 n.sp.
10000
8000
6000
4000
2000
0
Spain
Greece
Italy
Germany
France
Ukraine
Russia
Poland
Bulgaria
Austria
United Kingdom
Sweden
Sardinia
Switzerland
Crete
Serbia/Montenegro
Canary Is. & Selvagen
Sicily
Slovakia
Czech Republic
Hungary
Cyprus
Portugal
Norway
Corsica
Malta
Romania
Macedonia
Croatia
Slovenia
Netherlands
Denmark
Madeira
Moldova
Finland
Balearic Is.
Andorra
Bosnia-Herzegovina
Lithuania
Albania
Luxembourg
Estonia
Azores
Belgium
Turkey
Latvia
Ireland
Belarus
Azores Is.
Madeira Is.
Canary Is.

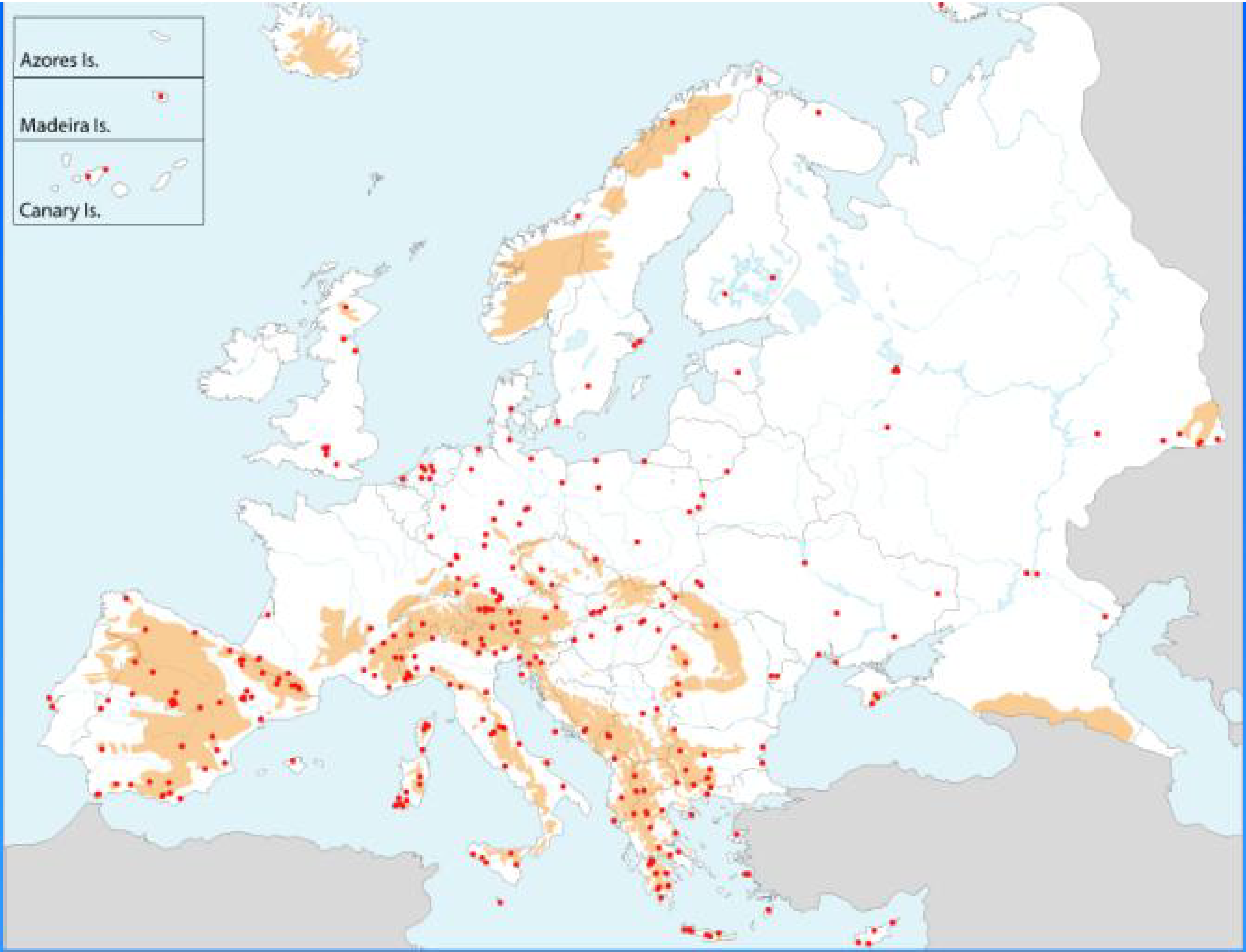

Type localities of 310 randomly selected species described between 1998 and 2003

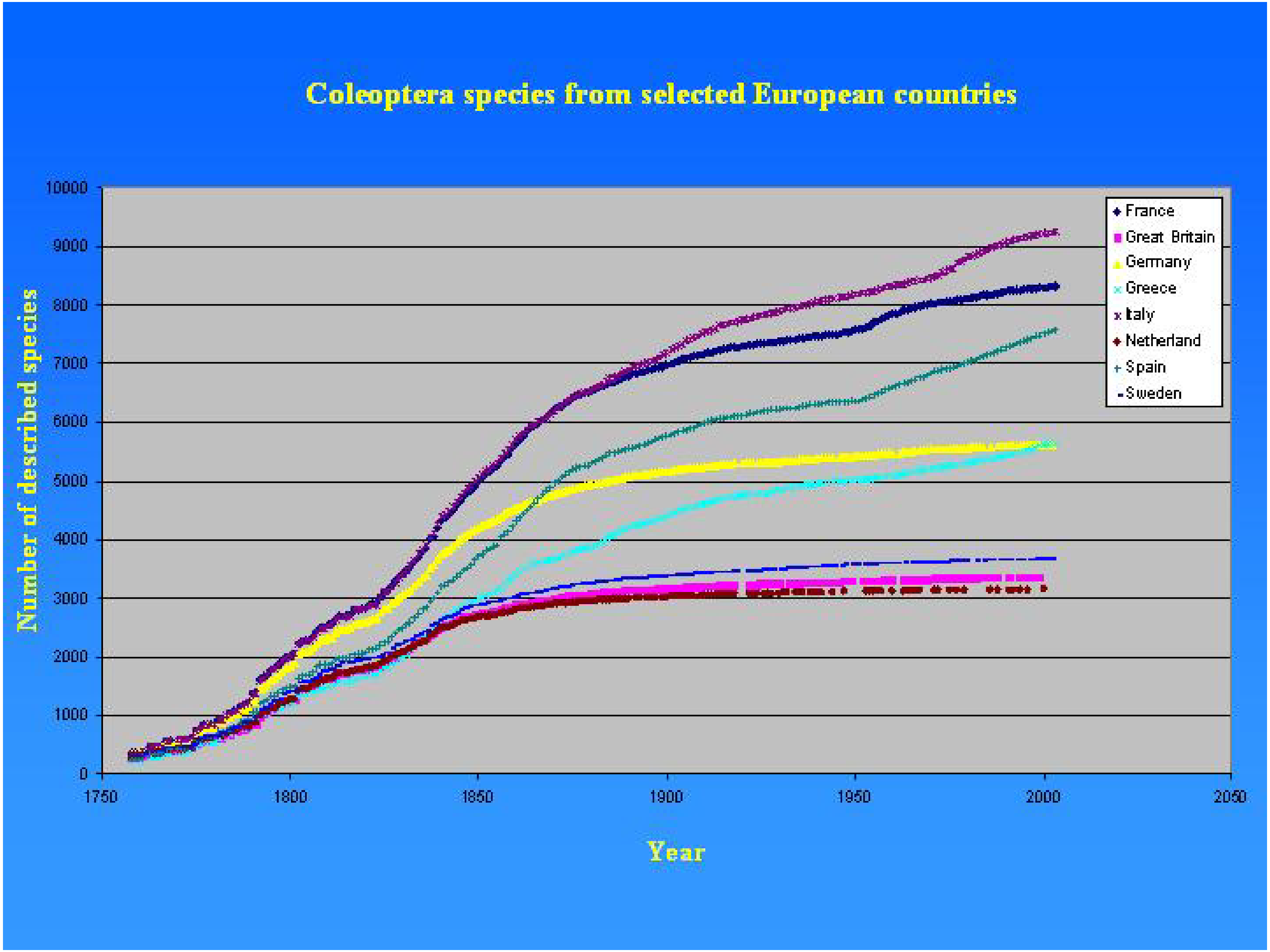
Coleoptera species from selected European countries
Number of described species
10000
9000
8000
7000
6000
5000
4000
3000
2000
1000
0
1750
1800
1850
1900
1950
2000
2050
Year
France
Great Britain
Germany
Greece
Italy
Netherland
Spain
Sweden

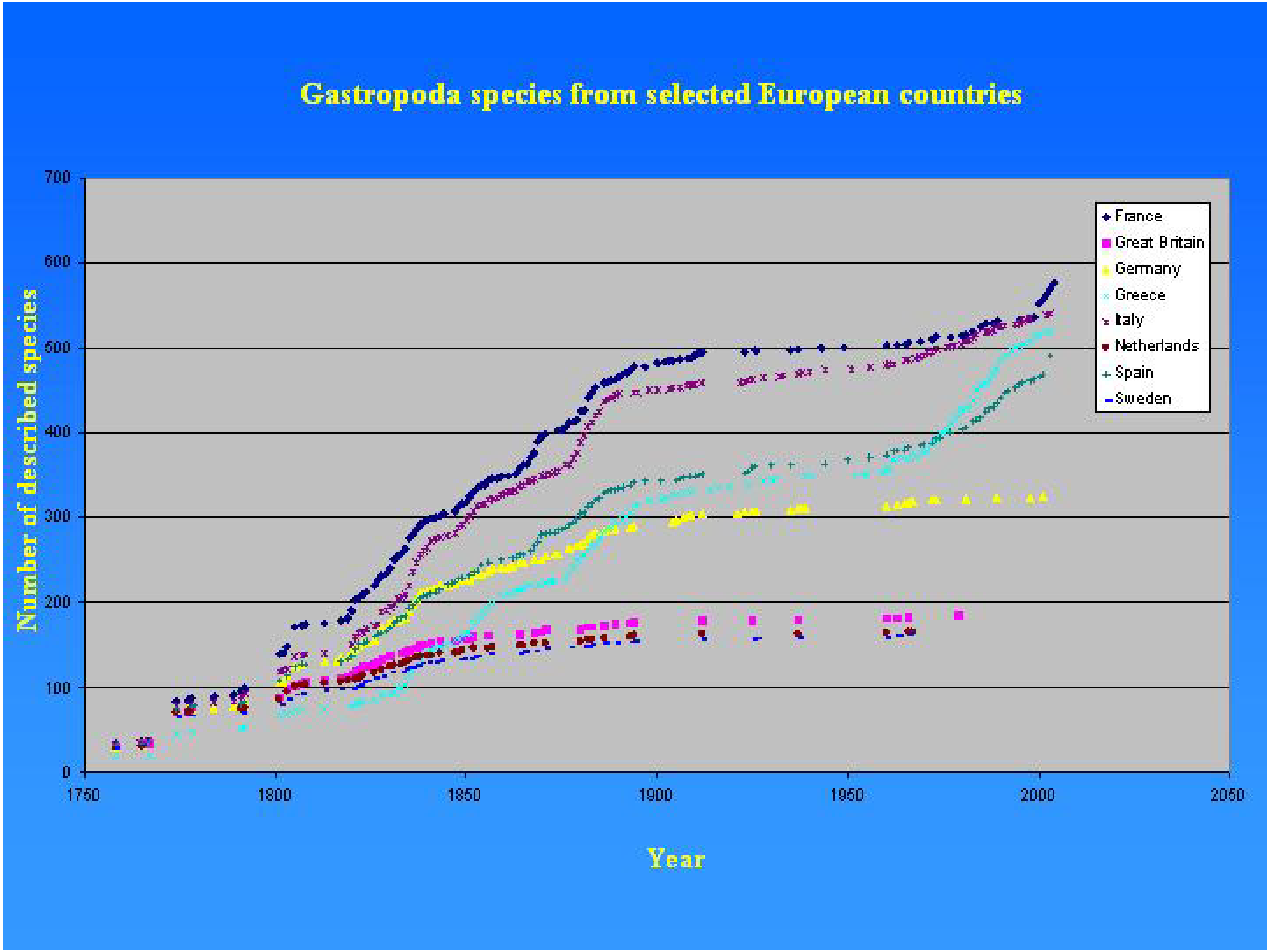

Gastropoda species from selected European countries
Number of described species
Year
700
600
500
400
300
200
100
0
1750
1800
1850
1900
1950
2000
2050
France
Great Britain
Germany
Greece
Italy
Netherlands
Spain
Sweden

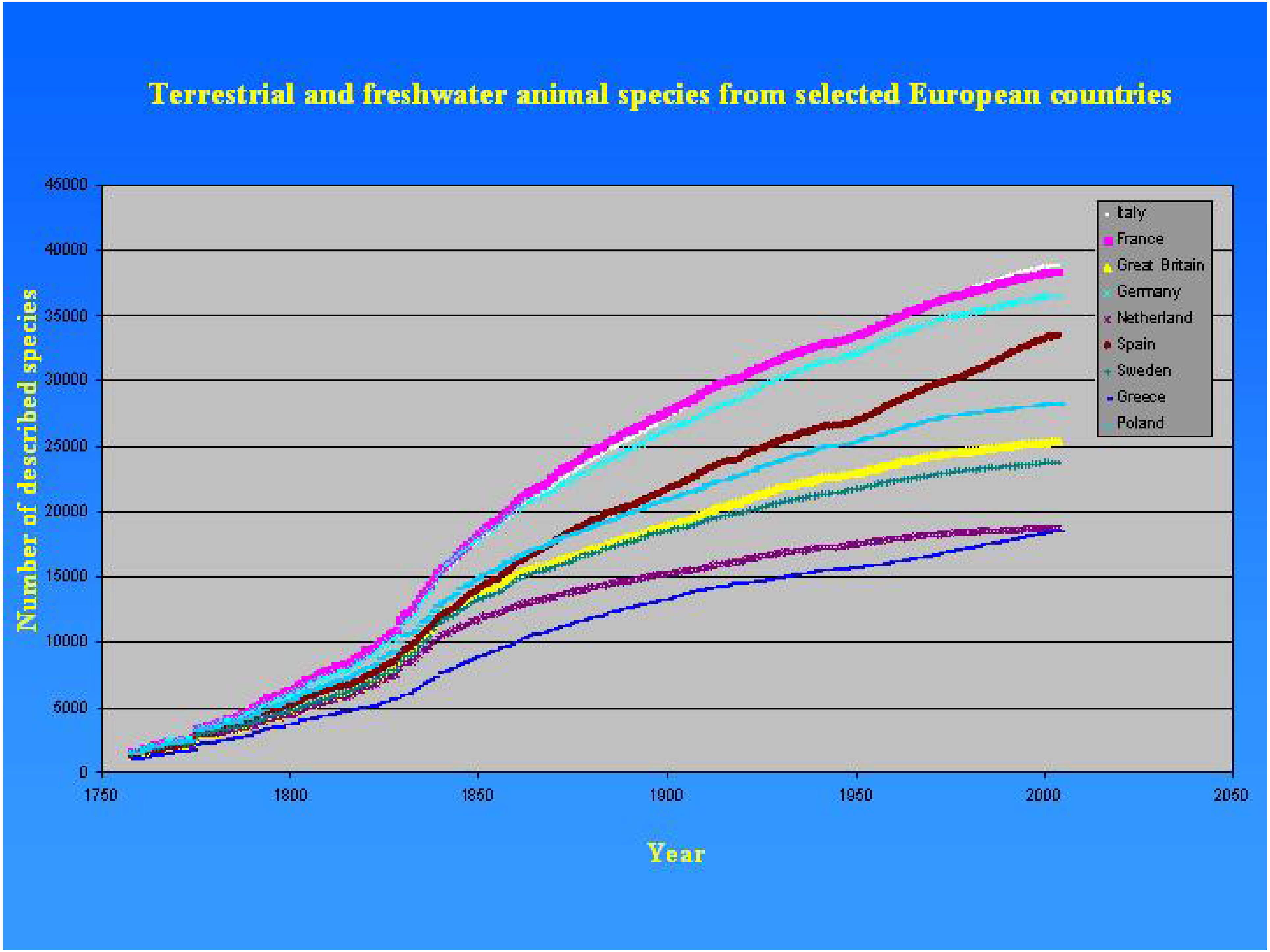
Terrestrial and freshwater animal species from selected European countries
Number of described species
45000
40000
35000
30000
25000
20000
15000
10000
5000
0
1750
1800
1850
1900
1950
2000
2050
Year
Italy
France
Great Britain
Germany
Netherland
Spain
Sweden
Greece
Poland

By whom?

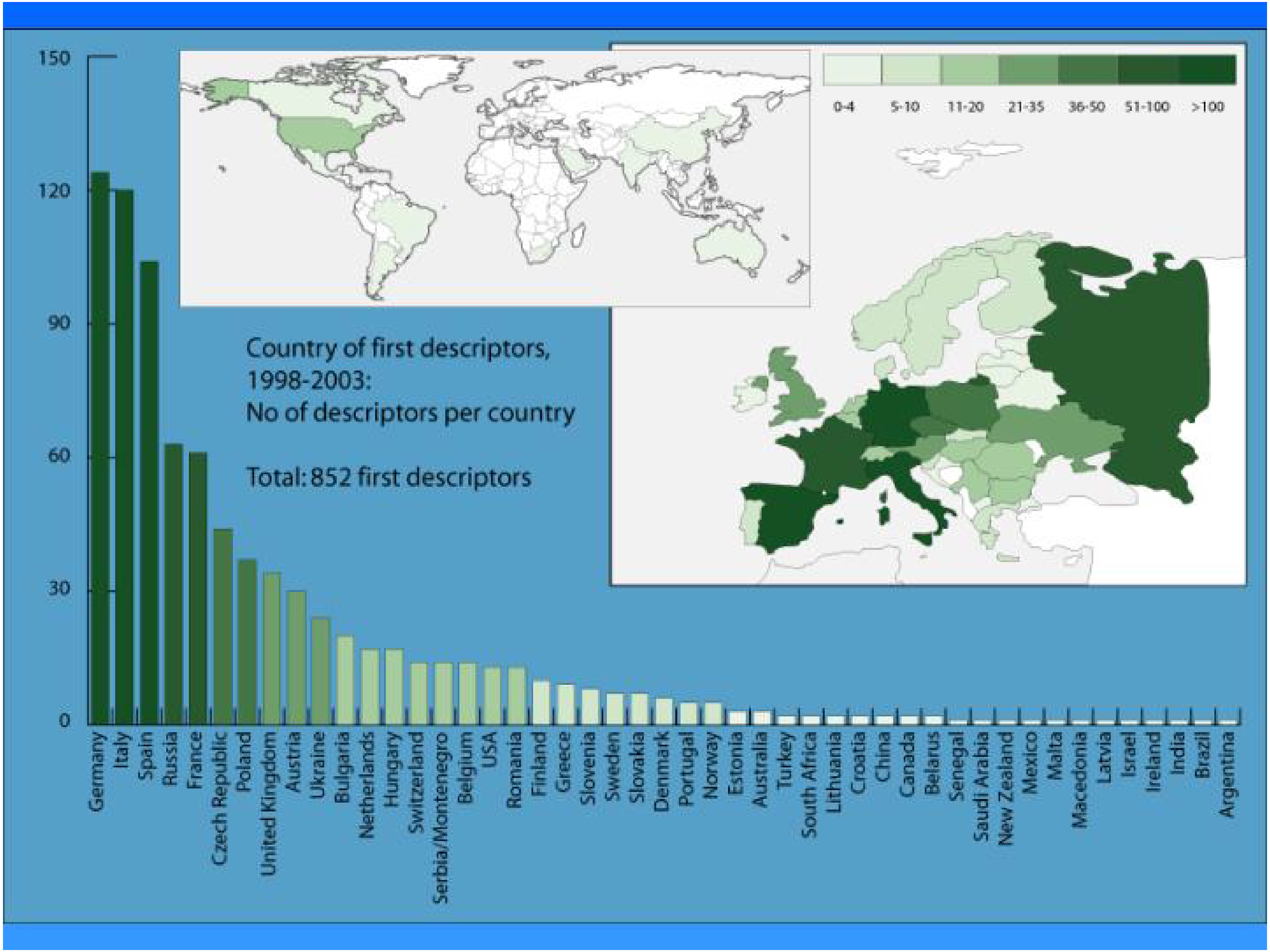

Country of first descriptors,
1998-2003:
No of descriptors per country
Total: 852 first descriptors
0-4
5-10
11-20
21-35
36-50
51-100
>100
150
120
90
60
30
0
Germany
Italy
Spain
Russia
France
Czech Republic
Poland
United Kingdom
Austria
Ukraine
Bulgaria
Netherlands
Hungary
Switzerland
Serbia/Montenegro
Belgium
USA
Romania
Finland
Greece
Slovenia
Sweden
Slovakia
Denmark
Portugal
Norway
Estonia
Australia
Turkey
South Africa
Lithuania
Croatia
China
Canada
Belarus
Senegal
Saudi Arabia
New Zealand
Mexico
Malta
Macedonia
Latvia
Israel
Ireland
India
Brazil
Argentina

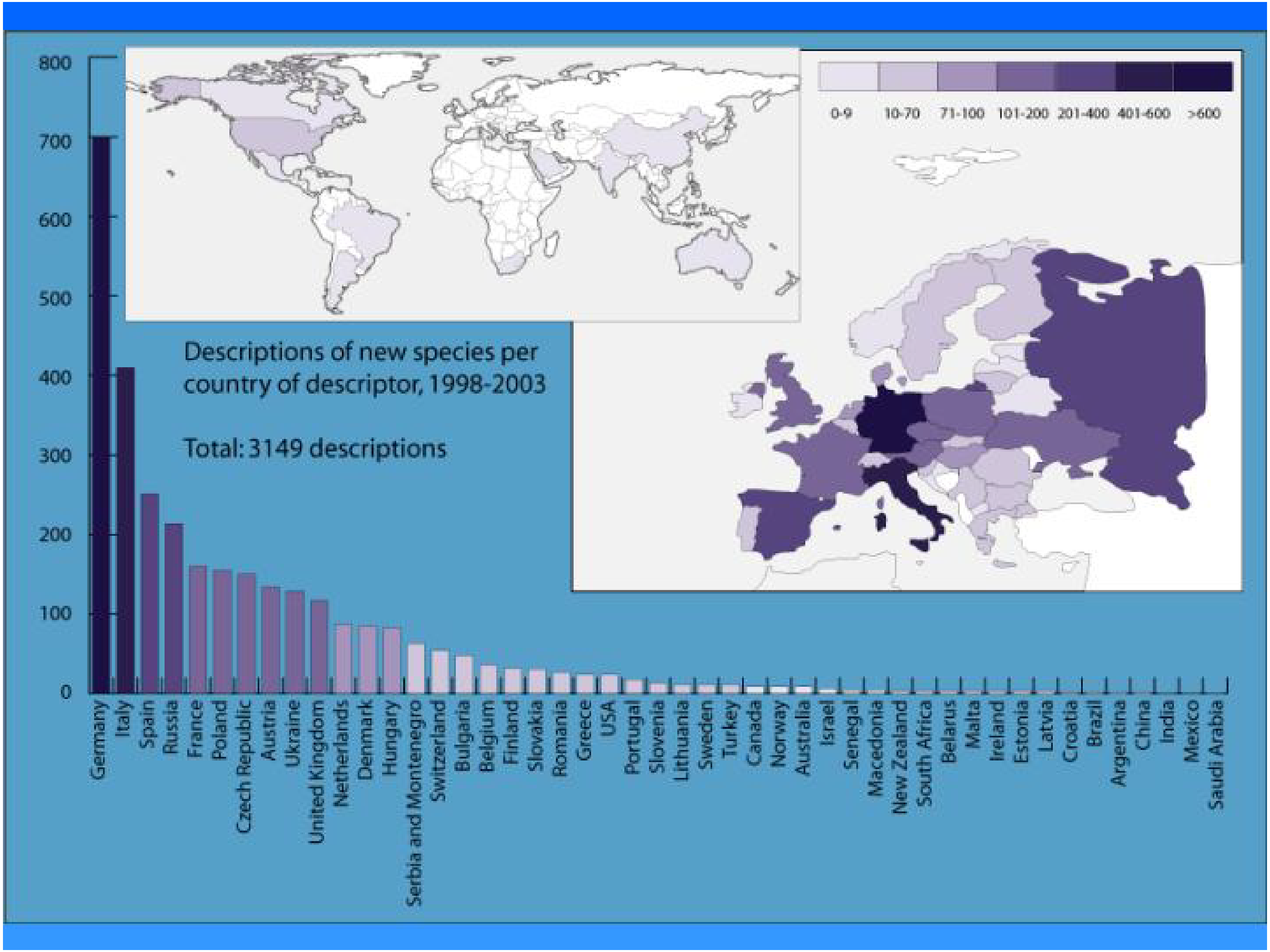
Descriptions of new species per country of descriptor, 1998-2003
Total: 3149 descriptions
0-9
10-70
71-100
101-200
201-400
401-600
>600
800
700
600
500
400
300
200
100
0
Germany
Italy
Spain
Russia
France
Poland
Czech Republic
Austria
Ukraine
United Kingdom
Netherlands
Denmark
Hungary
Serbia and Montenegro
Switzerland
Bulgaria
Belgium
Finland
Slovakia
Romania
Greece
USA
Portugal
Slovenia
Lithuania
Sweden
Turkey
Canada
Norway
Australia
Israel
Senegal
Macedonia
New Zealand
South Africa
Belarus
Malta
Ireland
Estonia
Latvia
Croatia
Brazil
Argentina
China
India
Mexico
Saudi Arabia

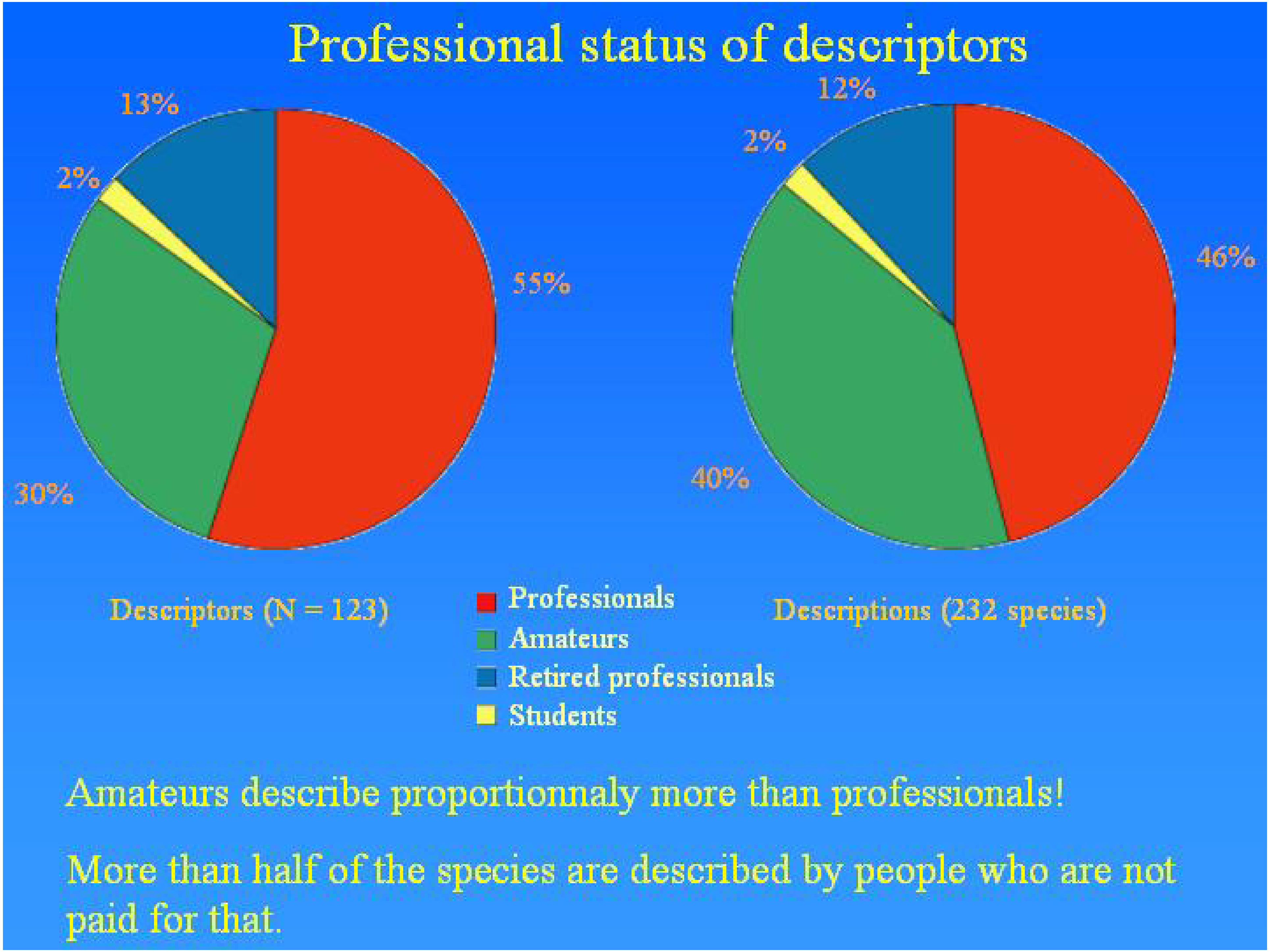
Professional status of descriptors
13%
2%
55%
30%
12%
2%
46%
40%
Descriptors (N = 123)
Professionals
Amateurs
Retired professionals
Students
Descriptions (232 species)
Amateurs describe proportionnaly more than professionals!
More than half of the species are described by people who are not paid for that.

How?

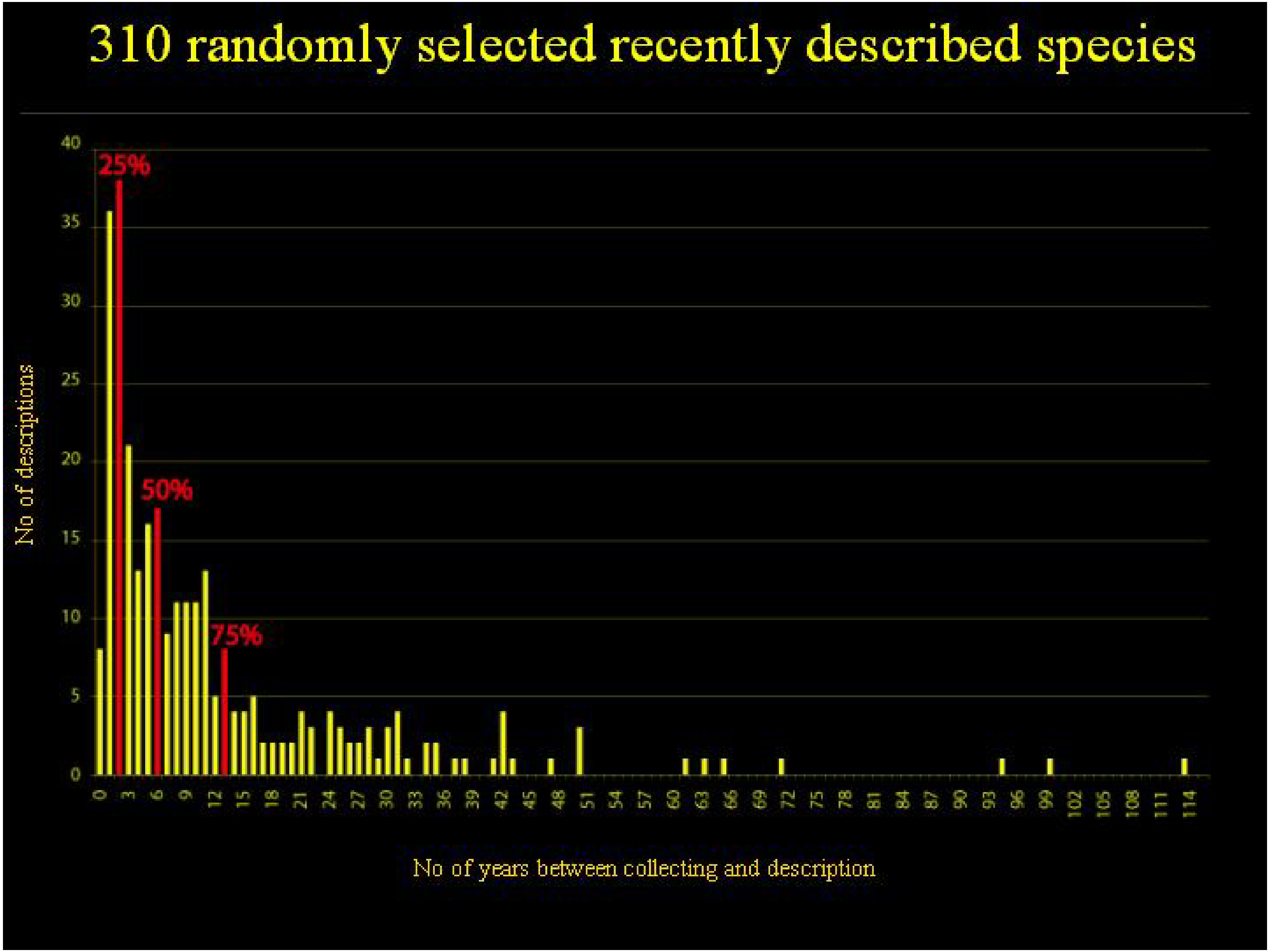

310 randomly selected recently described species
No of descriptions
No of years between collecting and description
25%
50%
75%
0
5
10
15
20
25
30
35
40
0
3
6
9
12
15
18
21
24
27
30
33
36
39
42
45
48
51
54
57
60
63
66
69
72
75
78
81
84
87
90
93
96
99
102
105
108
111
114

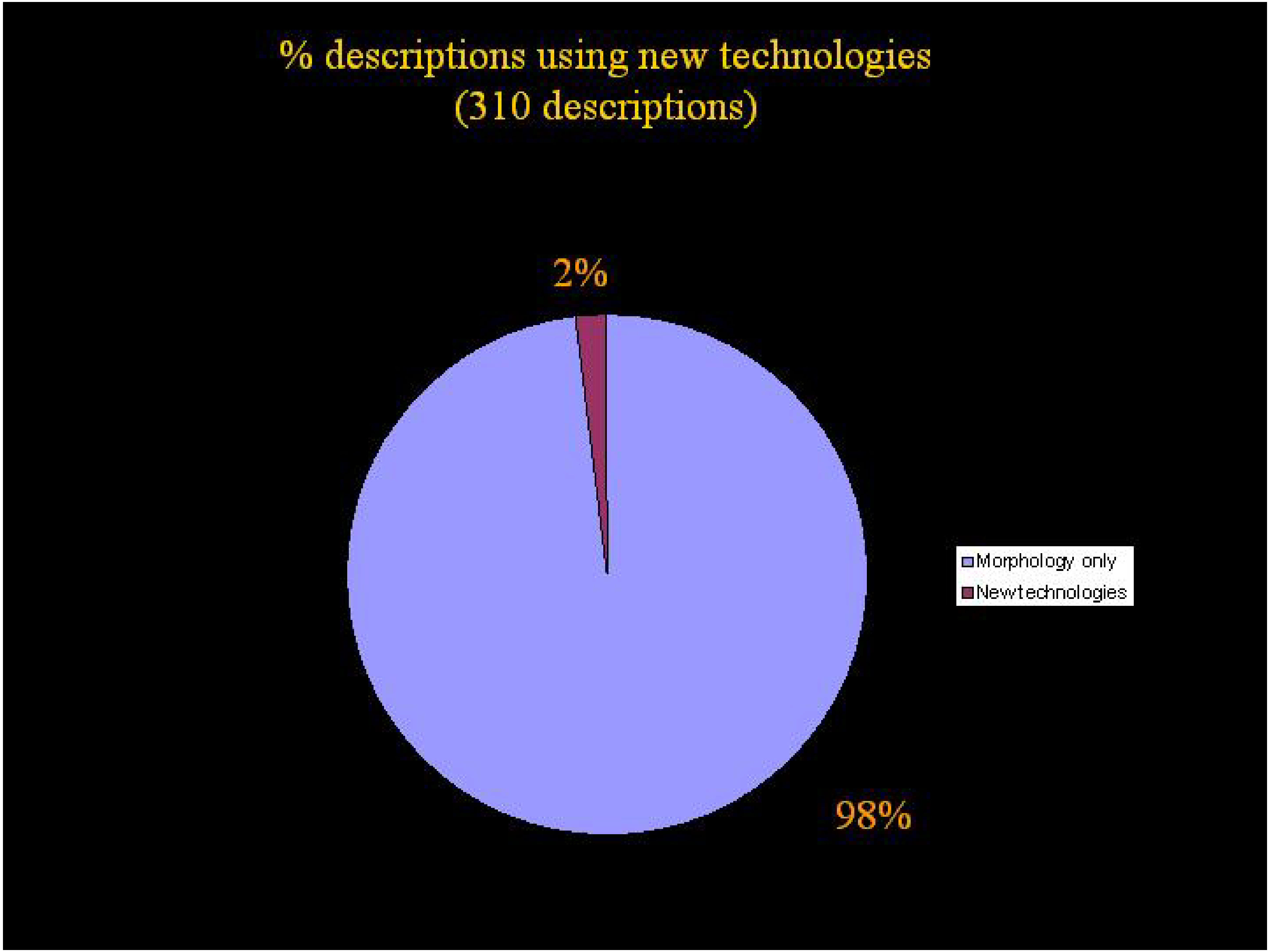
% descriptions using new technologies
(310 descriptions)
2%
98%
Morphology only
Newtechnologies

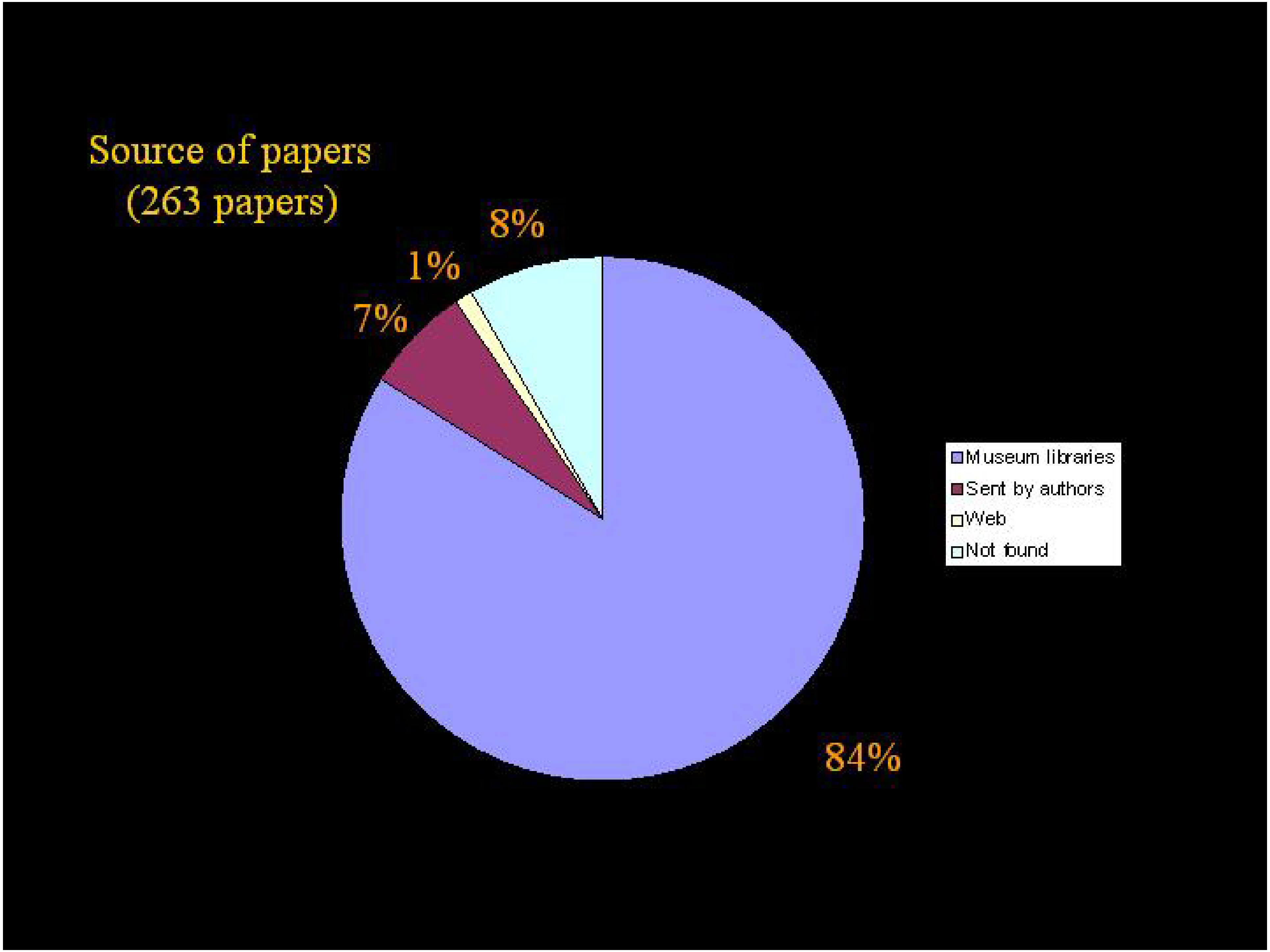
Source of papers
(263 papers)
8%
1%
7%
84%
Museum libraries
Sent by authors
Web
Not found
