# Supplementary material for: Fauna Europaea – all European animal species on the web
Source: Biodivers Data J. 2014 Sep 17;(2):e4034. doi: 10.3897/BDJ.2.e4034 (PMC4206781; doi:10.3897/BDJ.2.e4034)
Supplement: Supplementary material 9 — Fauna Europaea web statistics 2013 [file biodiversity_data_journal-2-e4034-s009.pdf]

Fauna Europaea web statistics

Total pageviews – 2013	5,525,641
Total pageviews – 2012	4,317,894
Total pageviews – 2011	3,584,484
Total pageviews – 2010	3,306,436
Total visitors – 2013	834,347
Total visitors – 2012	892,966
Total visitors – 2011	515,490
Total visitors – 2010	383,001
Yearly unique visitors – 2013	637,535
Yearly unique visitors – 2012	703,420
Yearly unique visitors – 2011	333,402
Yearly unique visitors – 2010	209,655

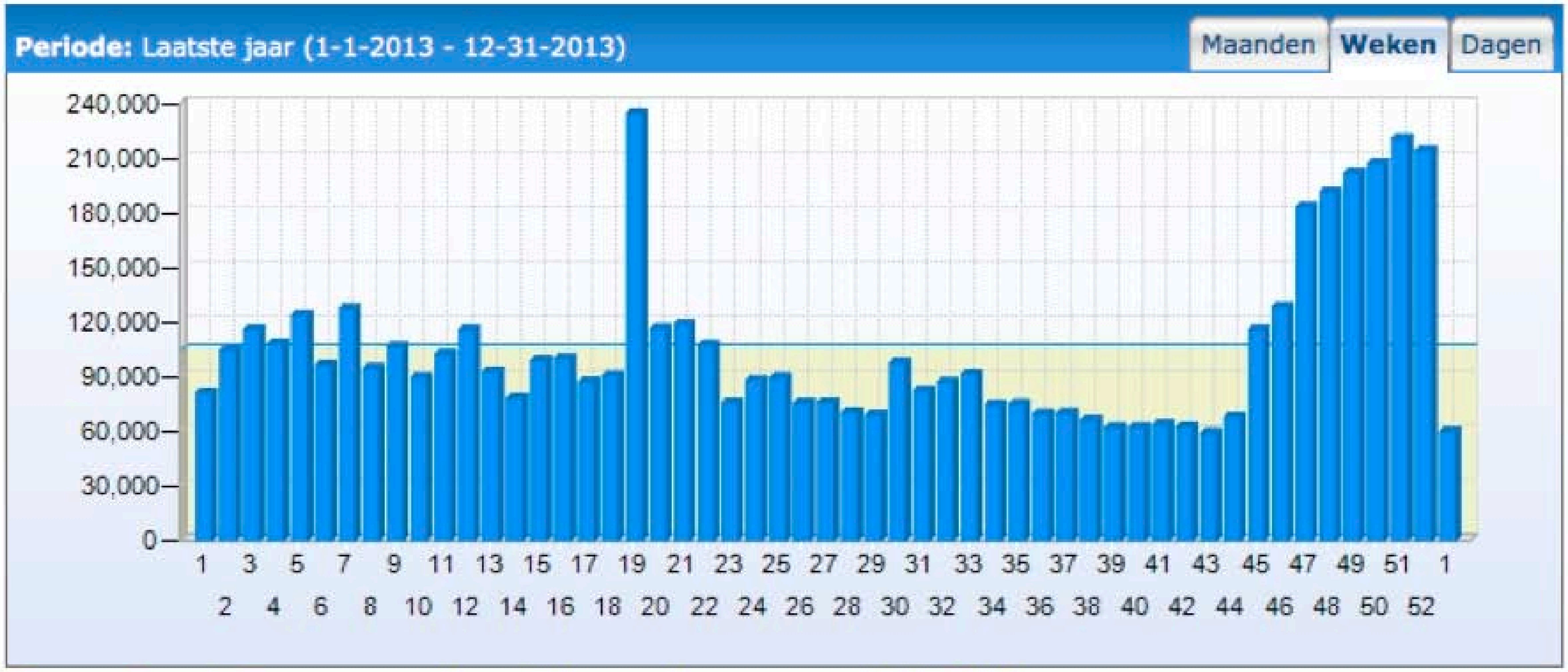

Figure: Fauna Europaea web-portal pageviews per week in 2013.
