# Supplementary material for: Fauna Europaea – all European animal species on the web
Source: Biodivers Data J. 2014 Sep 17;(2):e4034. doi: 10.3897/BDJ.2.e4034 (PMC4206781; doi:10.3897/BDJ.2.e4034)
Supplement: Supplementary material 10 — Fauna Europaea guidelines for cross-linking [file biodiversity_data_journal-2-e4034-s010.pdf]

Fauna Europaea

Cross-linking

Guidelines for Cross-linking

Yde de Jong & Günther Korb

Version — 23 July 2014

Cross-linking:

The European Commission encourage the use of Fauna Europaea as a standard list for Europe. This was also one of the rationales behind the project. Therefore we support the cross-linking of relevant taxonomic or related information systems to Fauna Europaea.

Two ways of cross-linking to Fauna Europaea have been established: (1) a method of direct linking via the respective FaEu taxon ID's and (2) an indirect method using parameterised URLs including the relevant text elements to be searched.

(1) Direct linking
A direct link to a taxon name in Fauna Europaea can be established by adding the appropriate taxon ID behind the following URL:
http://www.faunaeur.org/full_results.php?id={LSID}

For instance *Parus caeruleus* Linnaeus 1758 has the FaEu taxon ID '97274'. The relevant URL should be: http://www.faunaeur.org/full_results.php?id=**97274**

Recently for direct linking more condensed URIs has been created for species IDs following the below scheme:
http://www.faunaeur.org/t/{LSID}

For instance: http://www.faunaeur.org/t/**337992**

The proper Taxon IDs can be found at the website or within the data file.

---

(2) Indirect linking
A parameterised URL allows linking to the search function of Fauna Europaea. You can link to a genus name, a species name, a specific epithet or a higher taxon:

- To a genus name (for instance 'Parus'):
http://www.faunaeur.org/index.php?show_what=search%20results&genus=**Parus**

- To a species name (for instance 'Parus cristatus'):
http://www.faunaeur.org/index.php?show_what=search%20results&genus=**Parus**&species=**cristatus**

- To a specific epithet (for instance 'cristatus'):
http://www.faunaeur.org/index.php?show_what=search%20results&species=**cristatus**

- To a higher taxon (for instance 'animalia'):
http://www.faunaeur.org/index.php?show_what=search%20results&higher_taxon=**Animalia**

- It is also possible to include the 'search mode' (is, contains, starts with, ends with), for instance:

http://www.faunaeur.org/index.php?show_what=search%20results&genus=**Par**&species=**crista**&genus_search_mode=**starts%20with**&species_search_mode=**contains**

http://www.faunaeur.org/index.php?show_what=search%20results&higher_taxon=**malia**&higher_taxon_search_mode=**ends%20with**

Default search mode is always 'is'.

A similar method can be used for linking to advanced search input (for instance 'Parus' and '1783'):
http://www.faunaeur.org/advanced_search.php?show_what=search%20results&genus=**Parus**&year=**1783**

Evidently the second method contains a certain degree of fuzziness (meaning: is less exact) compared to the first method, however, the second method is far more easy to implement.

A shortened name URI is in preparation.

---

(3) Linking to PESI

To enable cross-referencing with the PESI portal[1], the following string can be used:

URL: http://www.eu-nomen.eu/portal/taxon.php?guid=urn:lsid:faunaeur.org:taxname:**441679**

Fauna Europaea LSID = 441679

---

(4) Linking to GBIF

To optimise cross-linking to the GBIF portal, Fauna Europaea is included into the GBIF Checklist Bank[2].

URL: http://data.gbif.org/species/**305289**/resource/**13560**

Fauna Europaea LSID = 305289
GBIF source = 13560

---

1 http://www.eu-nomen.eu/portal
2 Accessed via http://www.gbif.org/dataset/90d9e8a6-0ce1-472d-b682-3451095dbc5a
