# Supplementary material for: Fauna Europaea – all European animal species on the web
Source: Biodivers Data J. 2014 Sep 17;(2):e4034. doi: 10.3897/BDJ.2.e4034 (PMC4206781; doi:10.3897/BDJ.2.e4034)
Supplement: Supplementary material 12 — PESI Gap Analysis [file biodiversity_data_journal-2-e4034-s012.pdf]

A Pan-European Species-directories Infrastructure

PESI WP2 — Deliverable D 2.4

Detailed plan of how to complete taxonomic gaps in the pan-European species registers, including experts and informatics resources.

Roisin Nash (ed.)

Contents

Contents ... 2

Introduction ... 3

- The Taxon Registers in Europe ... 3
- Past procedures within the European registers ... 4
  - A) Species ... 4
  - B) Experts ... 7
  - C) Informatics resources ... 8

Identification of current taxonomic gaps ... 9

- Species ... 9
- Experts ... 11
- Informatics resources ... 13

Detailed Plan on how to complete taxonomic gaps ... 14

  - Species ... 14
  - Experts ... 16
  - Informatics resources ... 19

Discussion ... 22

References ... 24

Glossary of abbreviations ... 25

APPENDIX I ... 26

APPENDIX II ... 27

Introduction

The correct use of names is essential for biodiversity management and the availability of taxonomically validated standardised nomenclatures is fundamental for data infrastructures. A range of initiatives has been taken within the European Research Area to develop information systems assembling the integrated biological species information for various purposes. PESI is one of these initiatives and it provides standardised and authoritative taxonomic information.

However there are gaps within the infrastructure. In order to compile a detailed plan of how to complete the taxonomic gaps in the pan-European species registers it is necessary to revisit how theses registers initially collated, verified and designed the taxonomic framework and hierarchy that we see today.

The innovative approach to organising such registers is perhaps best perceived when realising that probably one fifth of all recently described species names are synonyms (Bouchet, 2006). The International Commission on Zoological Nomenclature (ICZN, based in London, UK) issues a Code of Nomenclature that promotes stability and universality by regulating the way scientific names are formed and used. However, it does not provide an umbrella for "authority lists" at a national, regional or global level, as the ICZN does not see its role as infringing on taxonomic opinions on the validity of species. New names proposed under the Zoological Code are compiled annually by the Zoological Record (published by Biosis UK), which provides the source for newly established names, but does not give opinion on the validity of the species denoted by these names. This is the register's role - to provide access to expert opinion on the species of animals living in Europe.

It is necessary here to first define an 'expert' within taxonomy before we can address the gaps. It is not as simple in practice however, as there is no established certification where competencies can be assessed. The working plan for the European Taxonomic workforce (D2.1) clearly illustrates this point stating that a very large part of the taxonomic work in Europe is carried out by non-career taxonomists i.e. amateurs retired professionals etc. For example more than half of the new European non-marine animal species described during the period 1998-2007 were described by non-career taxonomists (Benoît Fontaine et al., 2010). For the purpose of this report the term 'expert' is used as an all-inclusive term which supports everyone whom is involved and competent within their area of taxonomy.

The Taxon Registers in Europe

With the assistance of the Linnean Society of London a network of species-information projects were established in Europe, collectively titled Species 2000 Europa. The **European Register of Marine Species (ERMS)** was the first of the three component projects to be conducted, and it covered both fauna and flora in the marine environment. The **Fauna Europaea** project listed all land and freshwater animals (excluding protists), and recorded their occurrence in each country in Europe. The third component, **Euro+Med Plantbase**, covered the higher plants on land and in freshwater of Europe and neighbouring Mediterranean countries, updating the previously published Flora Europaea, a detailed synthesis of knowledge of Europe's flora, in electronic form.

The production and collation of the datasets and the review of lists and documents produced, was based on the work of scientists with expertise in taxonomy and long involvement in research throughout Europe. It was anticipated that each Register would become a standard reference (and technological tool) for biodiversity training, research and management in Europe.

Past procedures within the European registers

A) Species

Taxonomic framework, hierarchy and data verification.

ERMS: The hierarchy for ERMS was added in 2004 when the ERMS excel sheets were uploaded into the Aphia database. The hierarchy was mainly based on Margulis (and partly Parker - synopses), but this then got updated by the ERMS editors.

The first step taken towards upgrading ERMS was to re-organize the polychaete names registry along a hierarchal classification, based on recent phylogenetic studies (e.g. Fauchald & Rouse 1997, Rouse & Fauchald, 1997). This new classification scheme is very important for assigning equal status to clades at similar levels: a family clade is comparable to any other family clade, and so on. The latter has a particular meaning for studies targeted at marine biodiversity assessments that take into account not only species occurrences, but also their higher classification (e.g. the taxonomic distinctness measures, Warwick & Clark 2001). This extra information can provide valuable information on the species relatedness, which, in turn, is essential in defining both the inventory standards and also the criteria needed for departures from expectations in taxon diversity. This classification was created and submitted to the data management team, however simultaneously the system was upgraded to contain world-wide species (through the World Register of Marine Species - WoRMS). Conflicting classifications now existed and for data management reasons priority was given to the WoRMS classification scheme where for Polychetes it was by Dr. Kristian Fauchald who, in fact, largely adopted the one by Pettibone (1982).

Currently ERMS is examining the proposal that Dennis Gordon has drafted and that has also been adopted by Census of Life (CoL): Towards a management hierarchy: http://www.marinespecies.org/workshop/docs.php

Updated information and quality control

The process of updating followed a two-step approach, which guaranteed the quality control of the new information inserted into the registry:

(a) The taxon editor identified and contacted an associated editor to allocate the responsibility of updating the current information.

(b) In addition the taxon editor would identify another, world-class, taxon specialist to review the progress made; this reviewer/specialist would examine the updated information on the registry and provide a short review on the validity of the taxon names provided.

This two-tiered approach ensured high quality taxonomic information and made it possible to not only advertise PESI on the world-wide stage, but also to involve many taxonomists in the information updating process, either as associate editors or as reviewers.

Euro + Med PlantBase: The European Science Foundation European Documentation System (ESFEDS) database was used to provide the initial starting point for the taxonomic core of Euro+Med PlantBase. This database, developed at The University of Reading, comprised names and associated data from Flora Europaea. This was expanded with additional names and information from the MedChecklist (Greuter & al., 1984; 1986; 1989), the Flora of Macaronesia dataset (Hansen & Sunding, 1993), published regional and national Standard Floras and Checklists from the Euro-Mediterranean region, as well as with taxonomic monographs and relevant publications in scientific journals.

A key component of the Euro+Med PlantBase initiative was to critically evaluate the expanded database. A mechanism for the regional co-operative revision of the taxonomic status of all families, genera, species, subspecies and, where appropriate, cultivars described from the Euro-Mediterranean region was developed. The organization of this work involves specialists from over 50 countries and territories within the region. This revisionary process resulted in an agreed taxonomic core, which is one of the main outputs of the project.

Updated information and quality control

This Euro+Med PlantBase network was originally established between 2000 and2003 and continues to be active today. Seven Editorial Centres, each of them responsible for a certain share of the vascular plant families, and the Euro+Med Plantbase Secretariat coordinate the activities of the taxonomic experts and the network of the regional advisers all over Europe and the Mediterranean. The Editorial Centres are mainly responsible for updating taxonomic and nomenclatural information in close collaboration with the taxonomic experts for each group. The Euro+Med Plantbase Secretariat assembles the updates and is responsible for data standardization and corrections, thus strengthening the taxonomic backbone of European vascular plant taxa. The Secretariat distributes the updated plant families as they become available from the Editorial Centres into the network of regional advisers. The regional advisers, with their local expertise, mainly have the task of adding floristic information and to correct any erroneous entries. They receive taxonomically and regionally filtered ‘slices’ of the database, and they check this against their local information. The feedback from the regional advisers serves to update the information in Euro+Med Plantbase.

Fauna Europaea: The main building-blocks of the Fauna Europaea database were the family taxa that were established prior to Fauna Europaea. A default taxonomic higher hierarchy was thus needed to provide a framework for the lower taxa. This standard hierarchy was established with the help of leading European taxonomists and reviewed by the Fauna Europaea Taxonomic Advisory Team. Although limited by its original, practical scope (providing a management tool), its scientific scope (representing the temporal view of a restricted number of European experts), and its geographic scope (taxa not present within Europe are excluded, as are marine taxa), the taxonomic hierarchy thus established served as an authoritative standard for taxonomy in Europe.

Updated information and quality control

Fauna Europaea has a network of Group Coordinators (GCs), which, with associated contributing experts, make up the core of active members within the Fauna Europaea society.

Original Gap analysis within the registers

While each of the registers operated independently ERMS (carried out in 2006) and Euro+Med Plantbase (carried out in 2008) had a similar approach to gap analysis, where each taxon list was manually searched to identify whether there were gaps and priority or problematic families identified. The Fauna Europaea gap analysis (carried out in 2005) however, focused on the rate of discovery, recent descriptions and the number of descriptors in order to assess both geographical gaps in the data and whether they could predict further discoveries within Europe. It was expected within each of the registers that all lists could not be produced to the same standard because of the varying availability of recent published reviews, and expertise. For example, following gap analysis within ERMS, the species that became a priority for review were:

1.) lists not compiled for the entire European seas geographic area, namely Rotifera and Brachiopoda;

2.) lists that no expert on the European fauna checked, namely the non-epicaridean Isopoda, Cephalochordata, Appendicularia, Hemichordata, Hirudinea, Gnathostomulida, Ctenophora and Placozoa;

3.) lists known to be preliminary, including some of the above and several of the protist lists.

A further group of species lists that merit further attention within ERMS are the lists with many species. Because they have many species, it is most likely that these groups will contain species newly described to science, and/or changes in nomenclature, within a short time. The lists of macroalgae, Porifera, and Mollusca have been derived from well-established databases. The lists of fishes were cross-checked using a software programme against other world lists. However, other large lists were prepared for the first time for this project. Because of their size, no one person can be an expert on all of these species, and the editorial task per person is greater. Thus the lists of Polychaeta, Amphipoda, Harpacticoida and Turbellaria may benefit from further review.

Meanwhile in Euro+Med Plantbase problematic families and families that required part-funding in order to complete were identified (see Table 1).

Table 1. Problematic families and families that required part-funding in order to complete their checklists within Euro+Med Plantbase

Apiaceae	Elaeagnaceae	Loranthaceae	Polygalaceae	Saxifragaceae	Urticaceae
Apocynaceae	Escalloniaceae	Martyniaceae	Polygonaceae	Scrophulariaceae	Valerianaceae
Berberidaceae	Grossulariaceae	Menyanthaceae	Pontederiaceae	Sinopteridaceae	Violaceae
Caprifoliaceae	Hippocastanaceae	Pinaceae	Portulacaceae	Solanaceae	
Cistaceae	Hydrangeaceae	Pittosporaceae	Primulaceae	Tamaricaceae	
Clusiaceae	Hydrophyllaceae	Plantaginaceae	Rafflesiaceae	Theligonaceae	
Convolvulaceae	Lauraceae	Platanaceae	Salicaceae	Trapaceae	
Cupressaceae	Lentibulariaceae	Polemoniaceae	Santalaceae	Tropaeolaceae	

B) Experts

Initial contact: In ERMS an initial list of 1,200 people from 38 countries (29 European) with expertise in European marine species was compiled by the project. Each expert was contacted via e-mail (348) or letter (882) asking for their details to be checked and for permission to hold details in the database and display them on the website. The letter also had a summary of the ERMS project included.

A request was made to forward the letter, questionnaire and website address of the project to other relevant persons in the same institute. They passed it onto 160 colleagues that replied. Of the total of 614 respondents, 590 gave permission for their name to be held in the database (i.e. they were still active and available for this work).

A web-based submission form was also put on the web and a general call for submissions made to various Listserver discussion groups asking for experts' details.

Register of identification expertise: The taxa for which persons are experts were identified, where possible, to order level. In many cases additional information was made available, in some cases to family level. This information was been entered onto the database using the IOC register taxonomic structure for consistency.

Register of taxonomists: Additionally, the year of birth was collected to identify the age structure of experts and the country of origin. The extent of expertise was split into GLOBAL, REGIONAL and LOCAL level. Although some regional areas listed also fell into the local category. Cross-checking of this data is on-going.

The level of identification expertise was also noted, by asking the experts whether they are ecologists or other specialization, they have identification skills or they have written identification keys. Also, the status of experts was recorded and noted that the vast majority (80%) of those where professionals in the public service or academic sector.

The youngest person was 23 and the eldest, 89 years of age. The average age was 47, and on average taxonomists tended to be older than ecologists, perhaps because of modern emphases in

research funding. The age distribution did not indicate any imminent extinction of taxonomists. However, while there were generally more people identifying taxa with more species, there was no correlation between the number of taxonomists and species in their taxa. It is evident that some taxa with thousands of species have relatively few taxonomists.

In Fauna Europaea, it was discovered that only 55% of the descriptors of recently discovered species are professional and that amateurs describe proportionally more than professionals with more than half of the species described by people who are not paid for it.

Gap analysis: On examining the data received from ERMS, of particular concern was the fact that there was no correlation between the number of taxonomists and species in their taxa, and as marine species discovery rate analysis showed, there are more species yet to be described in these taxa. This suggests new efforts and funding needs to be found to address the mismatch between available taxonomic expertise and undiscovered species.

Recruitment: Group leaders and experts in the field should be asked to identify other known experts, who could potentially be contacted to fill the gaps. It was noted in ERMS that 26% of respondents were not contacted directly by the project. This suggests that despite efforts of the project team to compile individual contact details a number of experts may still be missing from the database.

C) Informatics resources

ERMS: The review of the bibliography of 842 marine identification guides found that there were fewer identification guides for southern European seas, although there were more species there compared to northern Europe. There were only adequate identification guides available for fish for all of Europe's seas. New guides were especially needed for the species rich, but smaller sized, taxa, such as polychaete, oligochaete, and turbellarian worms.

Fauna Europaea: A gap analysis carried out revealed that there was difficulty accessing resources such as primary literature with only 84% of the descriptions of recently described European species found in the various libraries of the Muséum National d'Histoire Naturelle in France in 2005, which is one of the largest in Europe and only 1% of these publications was found on the web. However, it was noted that the Natural History Museum in London had most of the journals that were missing in Paris.

Euro+Med PlantBase: Informatics resources were not covered in the gap analysis carried out by Euro+Med PlantBase.

Identification of current taxonomic gaps

In order to identify the current gaps present in the pan-European species registers, including experts and informatics resources, it was necessary to take a systematic, step-by-step approach, which looked at each of these elements separately and then in combination before trying to address how the gaps could be filled.

Species

A necessary first step was to harmonize the information infrastructures, which was a task assigned to WP5. Here, they initially implemented the consensus distribution model for species occurrences developed by WP4 and supported data enrichment that would allow the addition and extension of available data types. Then work began on the harmonization and merging of the data structures of the registers (i.e. the three taxonomic checklists). This allows an analysis of the information within the databases, particularly where originally there would have been a partial overlap between Fauna Europaea and ERMS.

The next logical step was to apply a consensus classification scheme, i.e. a working hierarchy for management purposes, to the merged registers in order to integrate taxonomic standards and to provide consistent responses from searches of different biodiversity resources. This was a task carried out by WP4. While the consensus classification for the time being will remain that of ERMS and Euro+Med Plantbase for the duration of the project, there is the option to move to the Catalogue of Life (CoL) classification in the future. Therefore, the logical option to identify gaps in both species and experts is to use the Catalogue of Life Taxonomic Hierarchy/Classification (Species 2000, 2011). It was decided to wait for the latest release in Jan 2011 to allow for a more informed analysis. (Please note that this classification scheme is still under review and as such the analysis will not be as accurate as was originally hoped).

Each classification scheme comes with a different taxonomic tree to organise the taxonomic data. Therefore, a number of discrepancies will ensue when trying to match both hierarchical data and corresponding experts. As a result it was originally thought to restrict the analysis to comparing Phylum, Class and Orders to identify any gaps present, as family level was thought to be too specific. While analysing to family level would provide more detailed and/or specific information it will not deter from the overall result of identifying the major gaps from the higher organisational level. For example, in most cases it would be easier to fill complete a gap for a family of species be it a checklist or an expert in that area than to fill complete a gap for a Phylum, Class or even Order of taxon. However, on receipt of databases to match from both the registers and PESI it became clear that it was necessary to match, and therefore analyse, to the lower level of family. The synchronising of data involved:

(i) Each Phylum, Class, Order and Family from the PESI database was manually checked and matched against to the CoL classification scheme.

  a. Where PESI has a level within the classification which was not found in CoL (e.g. subclass, infraclass, suborder etc.) the names were searched in order to ensure that CoL had not included them at a different level.

(ii) The non-matches in PESI vs CoL and those in CoL vs PESI were identified.

(iii) Where gaps in PESI were identified when compared to CoL, the taxa were checked for their distribution against the GBIF portal[1] to identify those gaps where the order or family in question was located outside of Europe (taking into consideration that the coverage of PESI is now expanded beyond the limits of Europe)

a. From the information provided by the portal however, it is not always evident whether the taxon is part of a collection held in that country or whether it is naturally occurring there. Where it is not specified clearly it is taken that the taxon is naturally occurring.

b. Where no information on the taxon is available on the portal then a search using the Google Scholar tool is carried out to try and identify a research paper on its distribution or with localities specified.

Table 1. The number of taxa from each Kingdom 'currently' not matching the corresponding classification schemes

Kingdom	In CoL not in PESI	In PESI not in CoL
**Animalia**	308	2,616
**Plantae**	487	31
**Chromista**	95	121
**Fungi**	436	84
**Bacteria**	385	4

[1] The GBIF portal is based on the 2007 CoL classification

Experts

List of the experts along with their associated Family or or higher taxon were obtained from the three main taxon registers (ERMS, Euro+Med PlantBase and Fauna Europaea) and the PESI database. The syncing of data involved:

(i) Each expert associated with a Phylum, Class, Order and/or Family from the databases was manually checked and matched against to the CoL classification scheme.

   a. Where PESI has a level within the classification which was not found in CoL (e.g. subclass, infraclass, suborder etc.) the names were searched in order to ensure that CoL had not included them at a different level if so then the expert could be assigned to the relevant taxon in CoL.

(ii) The non-matches in PESI vs CoL and those in CoL vs PESI were identified.

The results showed that Kingdom Animalia had only 4% of taxa not matched to editors based on the CoL classification scheme. However, as seen in Table 1 there are a number of taxa in PESI not accounted for as yet in the CoL classification, so this percentage may differ slightly once the classification is finalised. Also, we have included in the pie Chart below (Figure 1) the taxa considered living outside of Europe to illustrate the scale of the European flora and fauna.

Kingdoms such as Bacteria (415 taxa) which have only begun to be covered with Cyanobacteria (48 taxa) in PESI display much lower percentages of completeness (11.6%). The pie charts illustrating the degree of coverage of the remaining kingdoms can be seen in Appendix II.

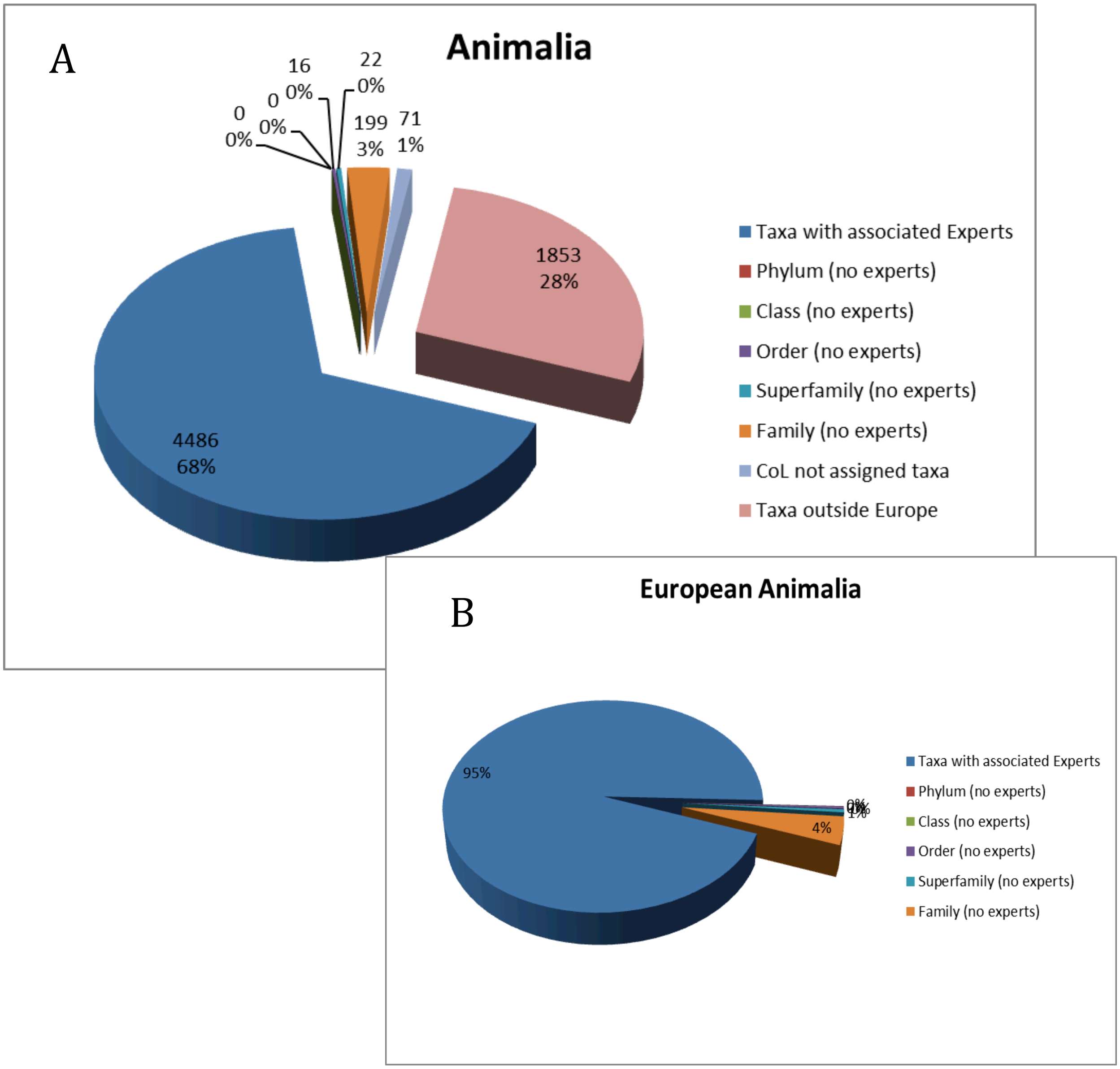

Figure 1. **A:** Kingdom Animalia based on the CoL classification scheme illustrating the relatively small proportion of gaps to fill in relation to editors. . B: The European Animalia shows the figures minus the worldwide taxa included in CoL.

Informatics resources

It is unlikely that there have been any significant changes in the allocation of taxonomic resources in the form of primary literature to museums around Europe since the last Fauna Europaea review and similarly any increase in identification guides would be minuscule. A gap identified by a PESI workshop was the availability of informatics resources to improve quality control and interoperability between online science resources, including taxonomic databases and journals, so as to make authoritative data and information more easily accessible to the scientific community.

However, where there has been a surge forward in increasing informatics resources and making them more freely available is in the latest online innovations, such as scratch pads and the Platform for Cybertaxonomy.

SCRATCH PADS

EDIT have developed what are known as Scratchpads i.e. a data-publishing framework which was built that allows distributed groups of scientists to create their own virtual research communities supporting biodiversity science. “EDIT’s system is a research platform that caters for the particular needs of individual communities through a common database and system architecture. This is flexible and scalable enough to support multiple networks, each with its own choice of features, visual design, and constituent data. In just two years the Scratchpads have been adopted by 100 multidisciplinary networks in more than 55 countries worldwide. Three quarters of our 1,200 users are based in EU member states and the framework has been used by the Encyclopedia of Life’s LifeDesk project” (extract from EDIT website).

PLATFORM FOR CYBERTAXONOMY

The EDIT Platform for Cybertaxonomy is a highly specialised software environment for taxonomists, a collection of tools and services which together cover all aspects of the taxonomic workflow. “The workflow is grouped into the following areas: taxonomic editing; publishing of edited data; data storage and exchange; collections and specimens; descriptions; fieldwork; literature; and geography. At the heart of the Cybertaxonomy platform is the Common Data Model (CDM), a repository for every conceivable type of data produced by taxonomists in the course of their work, and the backend for most EDIT components” (extract from EDIT website).

These initial developments have identified further gaps in informatics resources for example within the Platform for Cybertaxonomy:

a) full integration of structured descriptive information (following the SDD standard): The CDM Programming Code Library contains functions to import and export SDD, and it can display "natural language" text generated from SDD data, but it will not (by Feb 2011 and the completion of the EDIT project) contain features to edit structured descriptive data, nor to produce identification keys (traditional or interactive) from such data, nor to analyse that data. This can be done by existing tools such as Lucid, Exper, or DiversityDescription, but full integration into the Platform for Cybertaxonomy would be desirable, and a detailed plan exists on how to do so using Exper1 software developed in Paris (*pers comm* Walter Berendsohn, EDIT).

b) A toolkit to interactively develop simple web forms for data entry could help developers to build specialised applications using the CDM Library (*pers comm* Walter Berendsohn, EDIT).

On completion of the analysis for gaps in species and experts it is likely that identified gaps will correlate to gaps in informatics resources for those species.

Detailed Plan on how to complete taxonomic gaps

There is a clear link between taxonomic gaps whether they involve species experts or informatics resources. For example the absence of a taxonomic expert on a taxon may explain why there are no informatics resources available in the form of keys, guides, etc, for this group and essentially no checklist of species. Therefore in creating a 'plan' each of the three elements involved, while dealt with separately, will ultimately be intertwined. Some parts of this plan are already operational and moving forward while others are merely ideas and suggestions, which have to be implemented or discussed further. The success of this plan will depend on the due diligence of the taxonomic community around Europe.

Stage 1: The identification of gaps

While the current gaps, which need to be addressed, have been identified here based on the CoL 2011 there will be more or less once the CoL is completed and a European checklist is adapted. The subsequent stages of the plan are based on the current gaps identified here.

Stage 2: Identification of the end goal

The ultimate goal for each register has always been to have a full complement of experts corresponding to a complete checklist, as is physically possible. The corresponding informatics resources to augment the work were always a bonus. However, while the original 'core' taxa for PESI were Animalia, Chromista, Plantae, and recently extended to include the Fungi and Bacteria, the ultimate goal would be to include the absent kingdoms namely Archaea, Protozoa and Viruses.

Stage 3: Completion of Gaps

Species

The Catalogue of Life is a work in progress and while the latest possible version of the classification was used during this analysis (Jan 2011) there will be a possible option in the future to identify regional checklists such as Europe which will increase the ease of transfer or PESI data to a CoL classification. A European regional checklist was identified manually here using the GBIF portal. However in CoL there were still some outstanding taxa which were not assigned a taxon name but contain named taxa at the next level e.g. there are 34 divisions in the Kingdom Chromista and 105 in the Kingdom Fungi which have been entitled 'not assigned'.

'Gaps' vary from simply a family missing in PESI or the CoL to sub-divisions such as sub-orders, infraorders, etc, being absent from the CoL. This will cause future problems, when the current taxonomic tree in PESI is matched to the CoL tree for example where there are divisions

in PESI which are not in CoL and vice versa, however, this is beyond the scope of this report. Within the Kingdom Chromista PESI has 121 more taxon divisions with matching experts than CoL. On a larger scale PESI does not host a complete set of kingdoms, with Archaea, Protozoa and viruses missing.

At a lower level, which was not addressed here, but will need to be in the future, the question of the quality of the Global Species Database(GSD comes into question. Currently the information for CoL is sourced from a number of GSDs and as a result may not be representative of the Accepted/Valid/Correct (AVC) species of a taxon. PESI is accepted as a high quality database in relation to AVC species and therefore it would seem justified that the genus/species level in PESI is of a higher quality than CoL and could be used as the checklist for species names for Europe within CoL.

The completion or filling of gaps can be addressed in a number of ways:

i. Incorporation of new known checklists

   The gap analysis carried out by Euro+Med Plantbase identified a strategy of attaining external data sources to fill the gaps and have identified a number of potential checklists e.g. checklists from institutes, such as the Kew Gardens have been identified as a potential source of data for up to 35 families. This would be successful for the less problematic groups however, for those groups that present a problem to all and there are no checklists available then this requires a different approach.

   Work within WP3 and the Focal Point network has produced new sources of information from partner countries, where each has submitted country checklists (in varying stages of completeness) to PESI.

ii. Creation of new checklists

   In areas identified where checklists are completely absent, known experts in this area need to be encouraged to create such a checklist. Where European experts are not known, world experts should be contacted or workshops arranged coinciding with conferences to see if there is potential to create such a checklist. Checklists have been created from scratch by compiling a list of species reported by a region from a search of published accounts. Tools such as the taxon recognition in text developed by the Biodiversity Heritage Library would help when carrying out such a search.

The constant need to update checklists is a reflection on the more than 700 new species being descried each year almost four times the rate of two centuries ago (Fontaine et al., 2010). Fontaine goes further to say that this extraordinary rate of description of new species makes European an unexpected frontier for biodiversity exploration. This in turn may create gaps which will need to be addressed.

Experts

The use of pre-existing registers/databases of experts could potentially help to fill the gaps within the portal, but would also help to alleviate some of the workload from existing experts. It was decided to carry out an analysis based only on the current information on EditExpertNet, PESI FP database and the ETI BioInformatics specialist database to see whether this would potentially be a good first step. The syncing of data involved:

(i) The EditExpertNet database was manually searched entering the criteria or gender i.e. male or female (1,135 male and 370 female taxonomists) and taxonomists listing an interest in a taxon were recorded[2].

(ii) Each taxonomist associated with a Phylum, Class, Order and/or Family from the three new databases was manually checked and matched against the CoL classification scheme, minus the non-European families.

(iii) The new database with 331 taxonomists was then matched against the PESI and each register's database of experts (editors) to identify where gaps could potentially be filled. Table 3 in Appendix II illustrates both potential new editors to fill gaps and additional help for existing editors within the Kingdom Animalia.

(iv) The Focal Point (FP) database created within the PESI project where Focal Points from each country submitted a list of experts within their perspective countries would provide an additional pool of 'experts' or expert associations which could potentially be contacted to become editors moving forward. This database was not analysed within the current report however once the data has been incorporated in the PESI database it will be analysed and the report updated.

(v) The ETI BioInformatics specialist database was also matched and a selection of the results can be seen in Table 2 and in Appendix II, Table 3.

There was always the possibility that there would be a complete overlap of experts and no new experts would be identified. However this was rarely the case, and for example examining the Kingdom Fungi we found 4 new potential experts to cover gaps (Table 1) and 16 potential experts to help existing experts (Table 2).

While the aim of this report is to examine ways to fill the gaps it cannot be ignored the work load on the current editors. In the Kingdom Animalia there are 6,502 taxa which are covered by 236 editors of which a number of editors cover a single taxon while others may cover up to 130 taxa. This is a similar situation with both the Plantae and Fungi Kingdoms. Locating new editors in conjunction with sub-editors will help to alleviate the pressure and to allow current editors to be rejuvenated by a reduced workload and encouraged to be more active in their specific expertise.

Table 1. Newly identified taxonomists from the EDITExpertNet with interests in an order which currently have no identified expert

[2] There are currently >4,500 taxonomists with information in the EDITExpertNet. A random subset based on taxonomists who entered their gender was used to illustrate the potential usefulness of the database

	FP Experts	EDIT Experts	ETI BioInformatics specialists
**Order Russulales**		Slavomir Adamĉík	
**Order Boletales**		Machiel Noordeloos	
**Order Peltigerales**		Anna Guttova	
**Order Ascosphaerales**		Jens-Christian Frisvad	

Table 2. Taxonomists with an interest in areas which currently have experts but whom could be contacted to ease the workload now or to fill a gap in the future.

	FP Experts	EDIT Experts	ETI BioInformatics specialists
**Family Hymenochaetaceae**			T Bonsdor
**Order Agaricales**	Inita Daniele	Machiel Noordeloos, Tea von Bonsdorff	
**Phylum Basidiomycota**	Dorota Hilszczanska, Wieslaw Mulenko, Urszula Swiderska-Burek, Agata Wolczanska,	Andre Fraiture, Ivan Zimitrovich*, Ekatarina Malysheva,	
**Class Pezizomycetes**		Eugene Popov	
**Order Helotiales**		Eugene Popov, Gerard Verkleij	
**Order Lecanorales**		Anna Guttova	
**Phylum Ascomycota**		BJ Coppins, Dorota Hilszczanska, Wieslaw Mulenko, Urszula Swiderska-Burek, Agata Wolczanska,	Andre Fraiture

An additional search was carried out where the taxa considered as gaps were entered into the GBIF search box. The search followed through to 'Datasets with occurrences/Spreadsheet of results' links to generate a spreadsheet with the details of all GBIF records for a given

taxon. The spreadsheet was filtered for the records that fall in Europe and that were generated within the last 30 years. Then you have the option to select names and associated institution codes of up to three recorders/identifiers in hierarchical order according to the following criteria of their recording activity:

-relatively long timescale

-many different taxa within the target parent taxon

Similarly the Google Scholar tool was applied to carry out a search to try and identify taxonomic experts in Europe working on taxa identified as a 'gaps'. It was found that the most productive method was the EditExpertNet. While only 20% of the database had recorded their taxonomic interests, each of these were new names which could potentially aid current editors and for some be encouraged to consider a role as an editor to fill the gaps. Therefore gaps identified here **do not** indicate the absence of experts in Europe. However, the issue remains as to the willingness of these newly identified experts to participate in managing a taxon within PESI. This will in part reflect the success of elements of the 'working plan'.

The completion of EditExpertNet and the encouragement of those already registered to identify their areas of expertise may be a key to PESI reaching its goal of a compliment of taxonomic experts with resource pool to contact replacements. To develop this site further to become a social networking site could help with the shortage of professional taxonomists needed to identify organisms particularly as the facility would be available to anyone and unlike other technologies that require specialized equipment (Silvertown, 2010). EditExpertNet has an offshoot where the Dipterists are testing the start of a social networking site namely TaxNet for Diperists (http://192.38.114.240/elgg/).

On a larger scale PESI does not host a complete set of kingdoms, with Archaea, Protozoa and viruses missing. The authors suggest that an approach similar to the original methods employed by the current three main registers in Europe (outlined in 'the past procedures of European registers) is undertaken along with using the new databases available and the listservers to initiate new checklists.

The next steps towards completing the gaps are to establish

i. a protocol for contacting experts

ii. a sub-editor to help with the management and as a replacement if required

iii. a backup list of experts to contact in the future

iv. contact with the Focal Point network to encourage interested participants to become listed on the EditExpertNet

v. training from experts outside of Europe in areas where no experts identified in Europe

Informatics resources

"You can't just ask customers what they want and then try and give it to them. By the time you get it built, they'll want something new."

[Steve Jobs, quoted in The Guardian, Technology Section, 25 June 09]

1.) Long term support:

(i) Technical support for the taxonomists wanting to use Platform software developed such as the EDIT platform for cybertaxonomy.

Since the software has only recently reached a development stage that makes it actually usable, there are not many users up to this point. It's a cyclic process - in the long term, ideally institutional IT departments would take on the support for the researchers. However, these will do this only once the taxonomists actually use software tools. So the current situation is that support for the taxonomists will be through externally funded projects. Some follow-up projects have already been granted, mostly to support aspects of the technical infrastructure, but one also supporting users (GBIF Germany) (*pers comm* Walter Berendsohn).

(ii) Support for programmers developing new Platform-based software.

An open-source community working jointly on further developments needs to be created. Most of that will be project-driven and thus short-term result-oriented. Experience of successful open-source developments shows that some coordination is necessary for a successful community to remain productive. It is unlikely that a company will take on this role, institutions will have to join forces to establish a core staff to handle this coordination (*pers comm* Walter Berendsohn).

2.) Informatics resources through Funding

Addressing gaps identified through funding sources:

EU's e-Infra 2010 call: Virtual Biodiversity Research and Access Network for Taxonomy (ViBRANT) http://vbrant.org/

ViBRANT will help focus the collective output of biodiversity science, making it more transparent, accountable, and accessible. The system streamlines the production, management and publication of biodiversity data within multi-disciplinary virtual research communities engaged in biodiversity research. This will help structure the biodiversity science landscape through a highly flexible suite of web based tools and services. The project builds on a system developed within the FP6 funded European Distributed Institute of Taxonomy (EDIT), specifically Scratchpads, and is a collaboration with the LifeWatch initiative (part of the ESFRI roadmap). VIBRANT is integrated with a range of global biodiversity initiatives including the

Encyclopedia of Life, GBIF, the Consortium for the Barcode of Life, NCBI Genbank and the Biodiversity Heritage Library (extract from website).

3.) Quality control through existing sources:

Quality Control (Addressing misspellings, naming and classifications and erroneous or duplicate entries)

A recommendation from the PESI workshop on linking taxonomic databases with online science journals was to adapt existing tools/informatics resources, rather than develop new tools or resources de novo; and to work in a step-wise fashion to judge the success of initial measures, and facilitate adoption of new opportunities as they arose. For example, GBIF have developed an RSS feed which searches for pre-defined journal titles, abstracts and keywords for their relevance to particular species, and those inform database editors of new publications relevant to their taxa of interest i.e. a live link to recent journal publications.

Aside from the benefits of enriching species pages, increasing the visibility of published papers and more direct access to published papers for database taxonomic editors, this process would also improve the quality control. This would enable authors to check the nomenclature of species they will cite in their papers reducing the perpetuation of misspellings, naming and classification, of species prior to publication. It would also provide standard data schema (layout and vocabulary) for data appendices so as to facilitate their publication in integrating databases (e.g. GBIF) and use by other scientists.

The PESI workshop recommended that PESI could address this by writing to journal editors to inform them of these authoritative data publishing options and standards, perhaps in collaboration with GBIF and related initiatives (e.g. taxonomic societies like SMEBD and others).

Complimentary to this the post-publication services would at first be based on RSS feeds on species pages on online taxonomic databases where the RSS feed is modified to filter out papers relevant or published on a particular species. Potentially the RSS feed could link to a genus, family or higher taxon level where the species identity is not clear, or where it is a newly described species without a web page in the taxonomic database (e.g. "*sp. nov.*"). These processes would be done offline by comparing RSS feeds to a names index at the taxonomic database, and thus filtering out erroneous or duplicate entries. Candidate journals included PLoS, ZooBank and PhytoKeys. A list of taxonomic journals has also been complied (Appendix 3) for consideration.

4.) Investment into the universal application of new technologies

The development and application of automated species identification using technologies developed by researchers in pattern recognition, artificial intelligence and machine learning is another way forward to relieving time for experts to concentrate on other duties and potentially using this time to become gatekeepers helping to fill in the gaps. Computer systems now exist for classifying objects into between 2 and 30 categories e.g. The NHM in London has used DAISY (Digital Automated Identification System) to identify with 100% accuracy 15 species of wasps

and the University of Plymouth, UK, has used DiCANN (Dinoflagellate Categorisation by Artificial Neural Network) to identify phytoplankton species with about 72% accuracy (Valdecasas & Wheeler, 2010). Current grant applications for such interdisciplinary projects are falling between the boundaries defined by funding bodies in engineering and life sciences (Valdecasas & Wheeler, 2010).

Discussion

One of the main areas of discussion for the current document is the fact that the deviation between classifications schemes being matched will in essence cause gaps which are not ‘gaps’ but may simply result from remote classifications and/or different classification of taxon. However, the aim of this document is to look towards how to complete or fill the current gaps, so while the analysis gives an impression of the degree of completeness of the various Kingdoms the figures will vary somewhat. Particularly, so in relation to whether a taxon resided in Europe as it was not clear whether some taxa were actually located in Europe or whether they were held in museums or botanical gardens. From the data presented here we can see that Kingdom Animalia is the closest to nearing completion followed by the Kingdoms Chromista, Plantae, Fungi and Bacteria. The completion of each of the Kingdoms will not be without problems.

The EDITExpertNet looks very promising as a source of potential editors and subeditors particularly as it is currently testing a subsection of the site ‘TaxNet for Dipterists’ as a social networking site with a positive response to date. Through social networking the identification process would be made more efficient while simultaneously spreading real taxonomic knowledge (Silvertown, 2010). Here only a subset of taxonomists was examined and of these only 1/5th had registered their interests, and yet there were a significant number of new names appearing. Investing time to encourage the taxonomists already registered within the database to fill in their interests and to open a dialogue on their potential involvement in a European checklist is a must if this database is to be seen as a taxonomic pool of future editors.

The working plan for the European Taxonomic workforce (D2.1) clearly illustrates the need for an accreditation system where status and merit is attributed to gate-keepers and editors on the databases. This accreditation must come in parallel to either funding towards their time or scope within their current job description to allocate time to updating the online database. As important as the European checklist is and the many benefits towards its completion it must be taken into account the time and effort required not only to update the checklist but also the time and effort required to create the new checklists that are necessary to feed into the European Checklist for its completion. Earlier in this report it was mentioned how the unprecedented rate of species description has depended heavily on the scientific contribution of unpaid scientists. This argument is also presented in D2.1 (EWT Working Plan D2.1) and highlighted in a correspondence from Nature (Fontaine et al., 2010).

While this report has concentrated on filling gaps with ‘individuals’ as editors there is no reason why an organisation could not fulfil the same role and in fact some are already currently doing such roles. In fact a number of organisations are currently creating checklists particularly national recording schemes which are usually run by societies.

Essentially the ‘Hidden Kingdoms’ of Archaea, Protozoa and Viruses will be starting from scratch however, based on work done by the current European registers the initial stages should be easier as their will be a prototype of what they are aiming to achieve along with an example of the structural organisation of editors. Also, as they will be starting afresh their classification can be based on the CoL classification from the beginning. However even within the Kingdoms that have made great strides such as the Plantae there is still a section on the non-vasular plants such as mosses and liverworts which need to be tackled. Some of these taxa are important in relation

to ecological monitoring as they are indicator species and therefore it would be a priority moving forward to include these.

The recent decision of the EDIT Board of Directors to integrate with CETAF to continue supporting the activity of the established working group for IT departments (the ISTC, Information Science and Technology Committee) and to make that body fully operational by means of a substantial increase of membership fees will have a positive benefit for informatics resources in the future. Also a decision to continue supporting the activity of the established working group for IT departments (the ISTC, Information Science and Technology Committee) has been taken, and a subgroup is in the process of being constituted, which specifically addresses the Platform open-source development.

MacLeod *et al.* (2010) haven identified and tested the potential use of latest technology in pattern recognition in developing automating species identification is exciting. This would transform alpha taxonomy and would automate identification systems freeing them from the "drudgery of routine identifications" and allow them to concentrate on more difficult issues such as discovering, describing and revising species concepts etc. (MacLeod *et al.,* 2010). While agreeing with this concept a word of caution was issued as this technology would not be universally applicable and would not work with the 5,000 species of Drosophila (Valdecasas & Wheeler, 2010).

Species names are critical data for biodiversity management and for most branches of biodiversity related applied and fundamental research. Correct species names are likewise important for pest and disease control, agriculture, aquaculture, forestry, fisheries, habitat protection, environmental control, and nature conservation. Therefore the availability of high quality taxonomic name services, including valid species names and names relationships, functioning as authoritative taxonomic reference system, is essential for biological data management.

In conclusion the last paragraph should leave no doubts as to the benefit of filling the gaps and tackling the 'hidden' Kingdoms to provide a comprehensive checklist for Europe. However, this it is not without a price and inevitably further funding will be required to move forward.

References

Bouchet, P. (2006). The magnitude of marine biodiversity, in: Duarte, C.M. (Ed.) (2006). *The exploration of marine biodiversity: scientific and technological challenges*. Fundación BBVA: Bilbao, Spain. 158 pp.

Fauchald, K. and Rouse, G. W. (1997). Polychaete systematics: Past and present. *Zoologica Scripta* **26(2)**: 71-138.

Fontaine, B. et al., (2010). European bounty for taxonomists. *Nature* **467:** 377

Greuter, W., Burdet, H. M. and Long, g. (1984, 1986, 1989). *Med-Checklist.* Vols 1, 2 & 4. Geneva/Berlin Conservatoire et Jardin botanique de la Ville Genève/ Botanischer Garten & Botanisches Museum Berlin-Dahlem.

Hansen, A. and Sunding, P. (1993). Flora of Macaronesia. Checklist of vascular plants 4th Ed. *Sommerfeldia* **17**: 1-296.

MacLeod, N., Benfield, M. and Culverhouse, P. (2010) Time to automate identification. *Nature* **467:**154-155

Pettibone, M. H. (1982). Annelida. In S. P Parker (ed.), *Synopsis and classification of living organisms*, *Vol. 2*., pp. 1-43, McGraw-Hill Book Co, New York.

Rouse, G.W. and Fauchald, K. (1997). Cladistics and polychaetes. *Zoologica Scripta* **26(2)**: 139-204.

Silvertown, J. (2010) Taxonomy: include social networking. *Nature*: **467:** 788

Species 2000 (2011). Catalogue of Life accessed at www.sp2000.org Feb 2011.

Warwick, R.M. and Clarke, K.R. (2001) Practical measures of marine biodiversity based on relatedness of species. *Oceanography and Marine Biology* **39:** 207-231.

Valdecasas, A.G. and Wheeler, Q.D. (2010) Taxonomy: add a human touch too. *Nature*: **467:** 788

Glossary of abbreviations

AVC – Accepted/Valid/Correct.

CDM – Common Data Model

CETAF - Consortium of European Taxonomic Facilities

CoL – Catalogue of Life

EDIT – European Distributed Institute of Taxonomy

ERMS – European Register of Marine Species

ESFEDS - European Science Foundation European Documentation System

ESFRI - European Strategy Forum on Research Infrastructures

ETI - Expert Center for Taxonomic Identification

FP – Focal Points (national representatives for biodiversity and taxonomic research)

GBIF – Global Biodiversity Information Facility

GC – Group Coordinator (Fauna Europaea)

ICZN – International Commission of Zoological Nomenclature

IOC – Intergovernmental Oceanographic Commission

ISTC - Information Science and Technology Committee

NCBI - National Center for Biotechnology Information

PESI – Pan-European Species-directory Infrastructure

PLoS - Public Library of Science

RSS – Really Simple Syndication

SDD – Structured Descriptive Data

SMEBD - Society for the Management of Electronic Biodiversity Data

VIBRANT - Virtual Biodiversity Research and Access Network for Taxonomy

WoRMS – World Register of Marine Species

WP4 – PESI Work Package 4 – Taxonomic Standards and Authority Files

WP5 – PESI Work Package 5 – Taxonomic information e-infrastructure

APPENDIX I

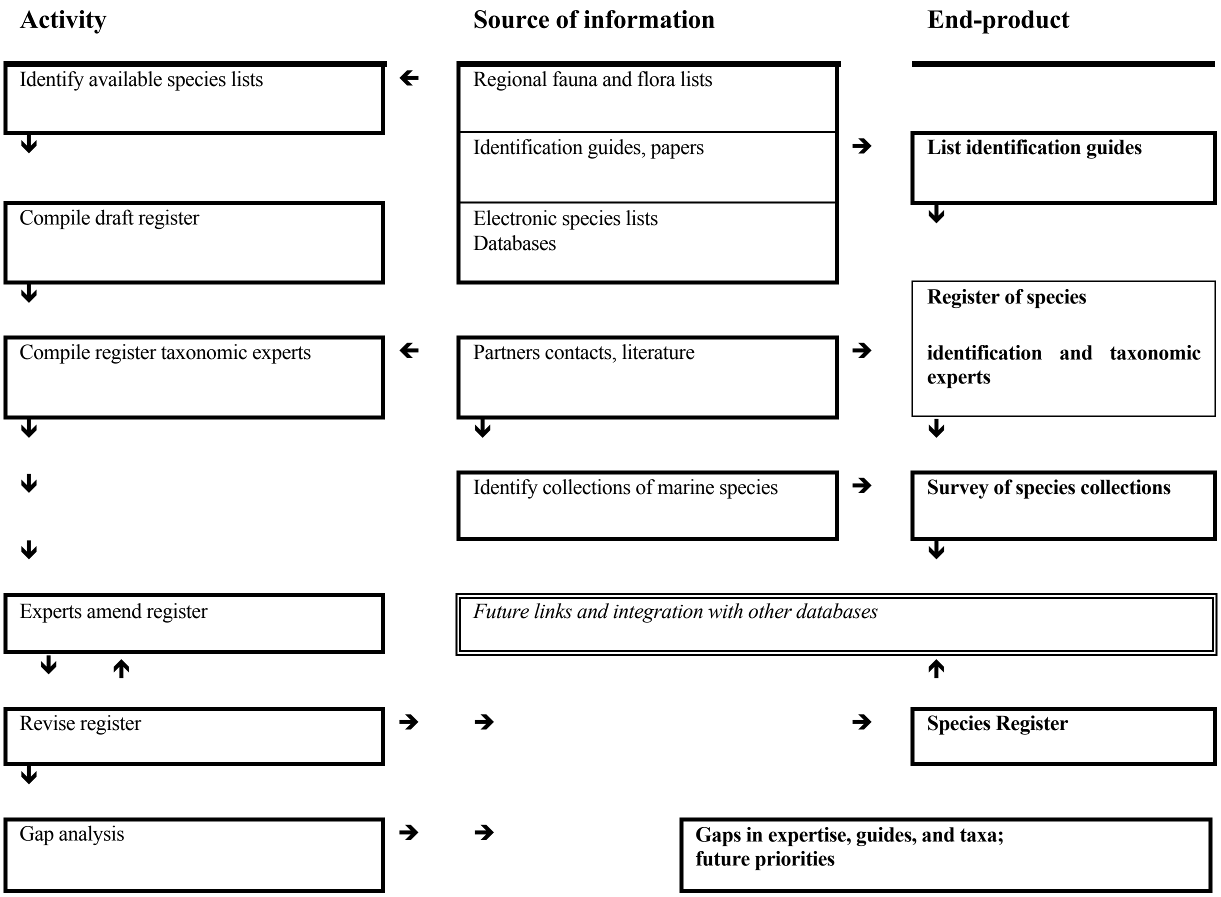

Figure 1. A diagram illustrating the elements of the European Register of Marine Species project, including activities, information sources, and end-products.

APPENDIX II

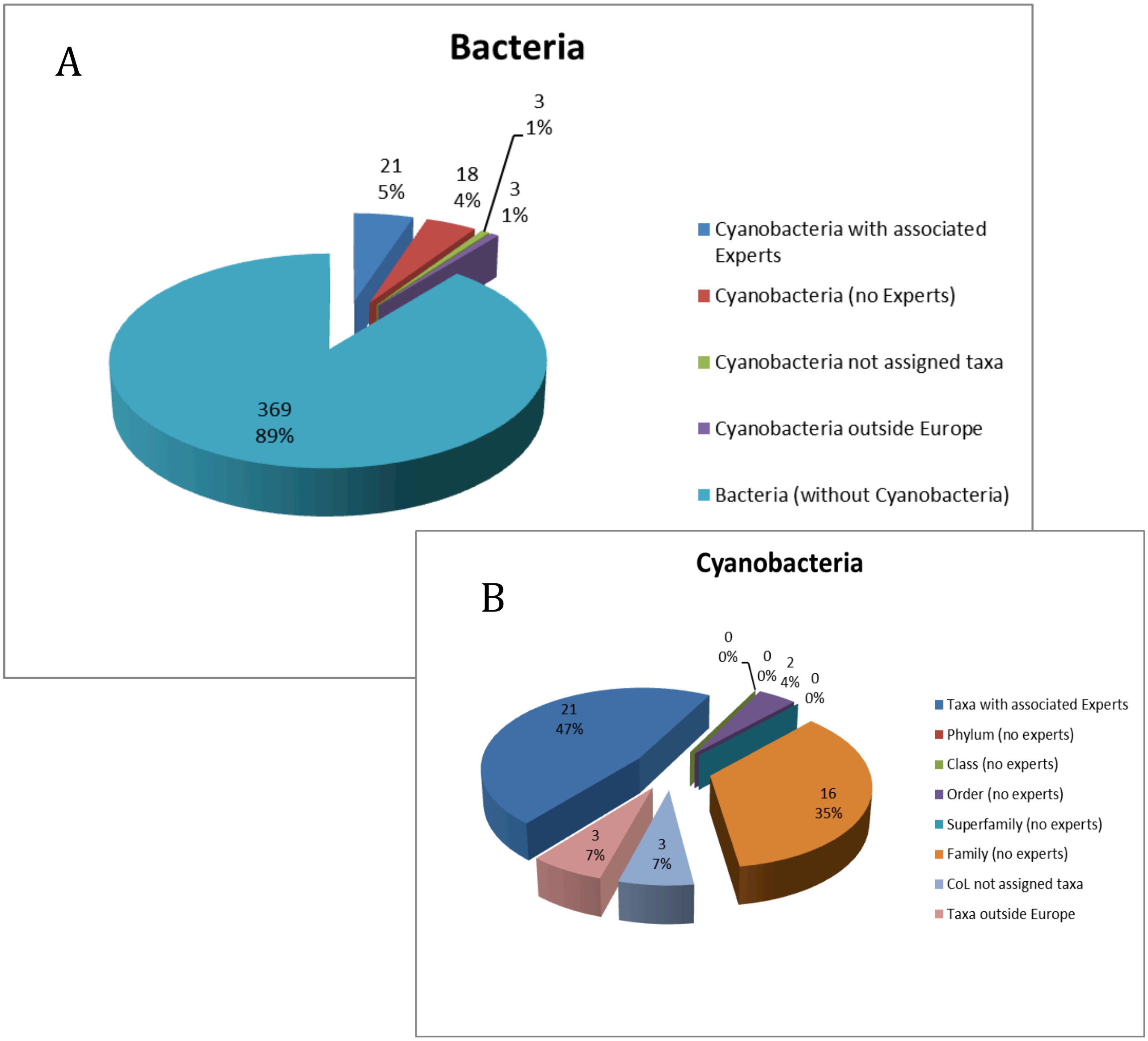

Figure 2. A: Kingdom Bacteria showing the relatively small portion covered by Cyanobacteria. B: Phylum Cyanobacteria based on the CoL classification scheme illustrating the relatively proportion of gaps to fill in relation to editors.

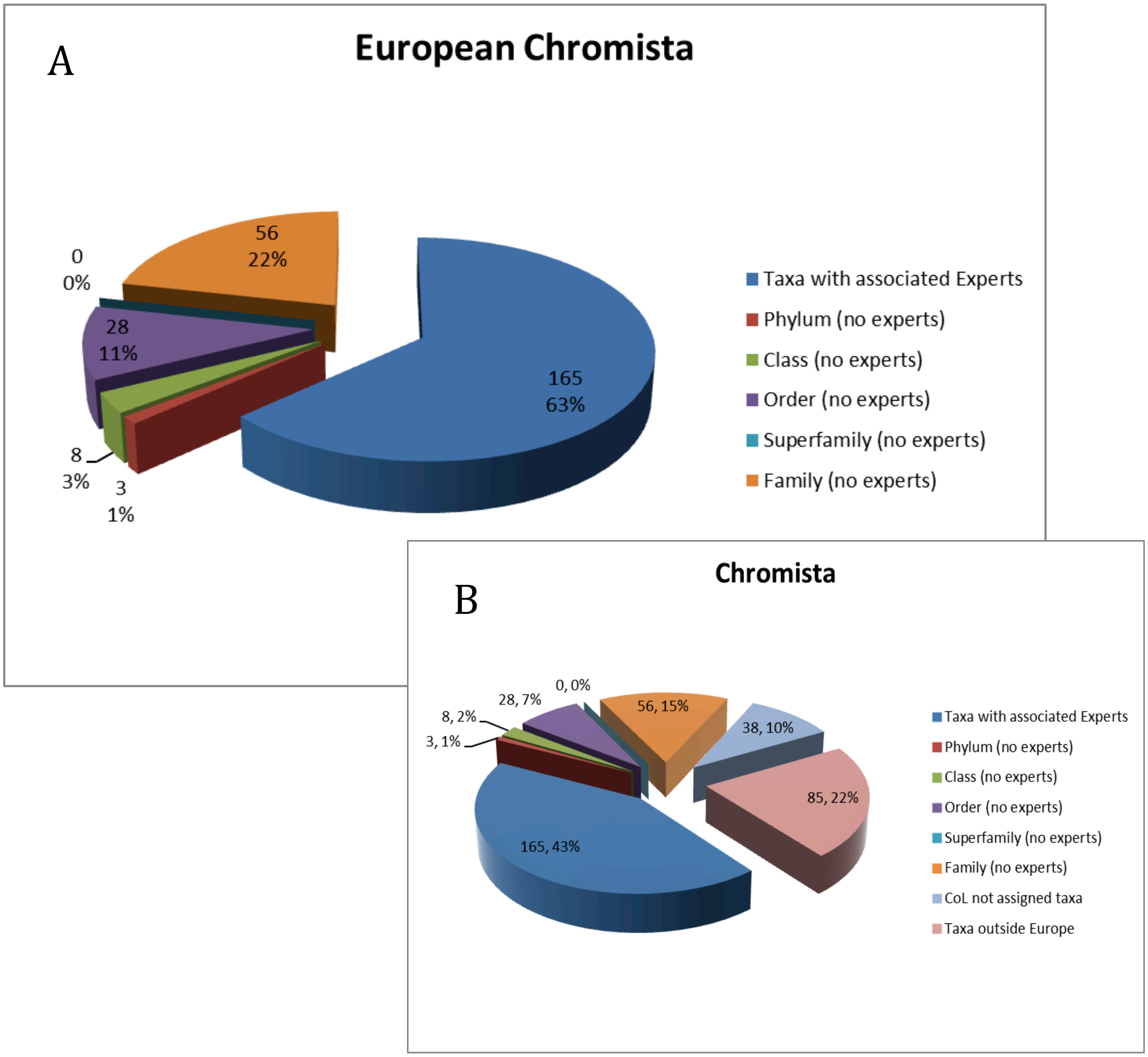

Figure 3. A: Kingdom Chromista based on the CoL classification scheme illustrating the relatively proportion of gaps to fill in relation to editors. B: The European Chromista shows the figures minus the worldwide taxa included in CoL.

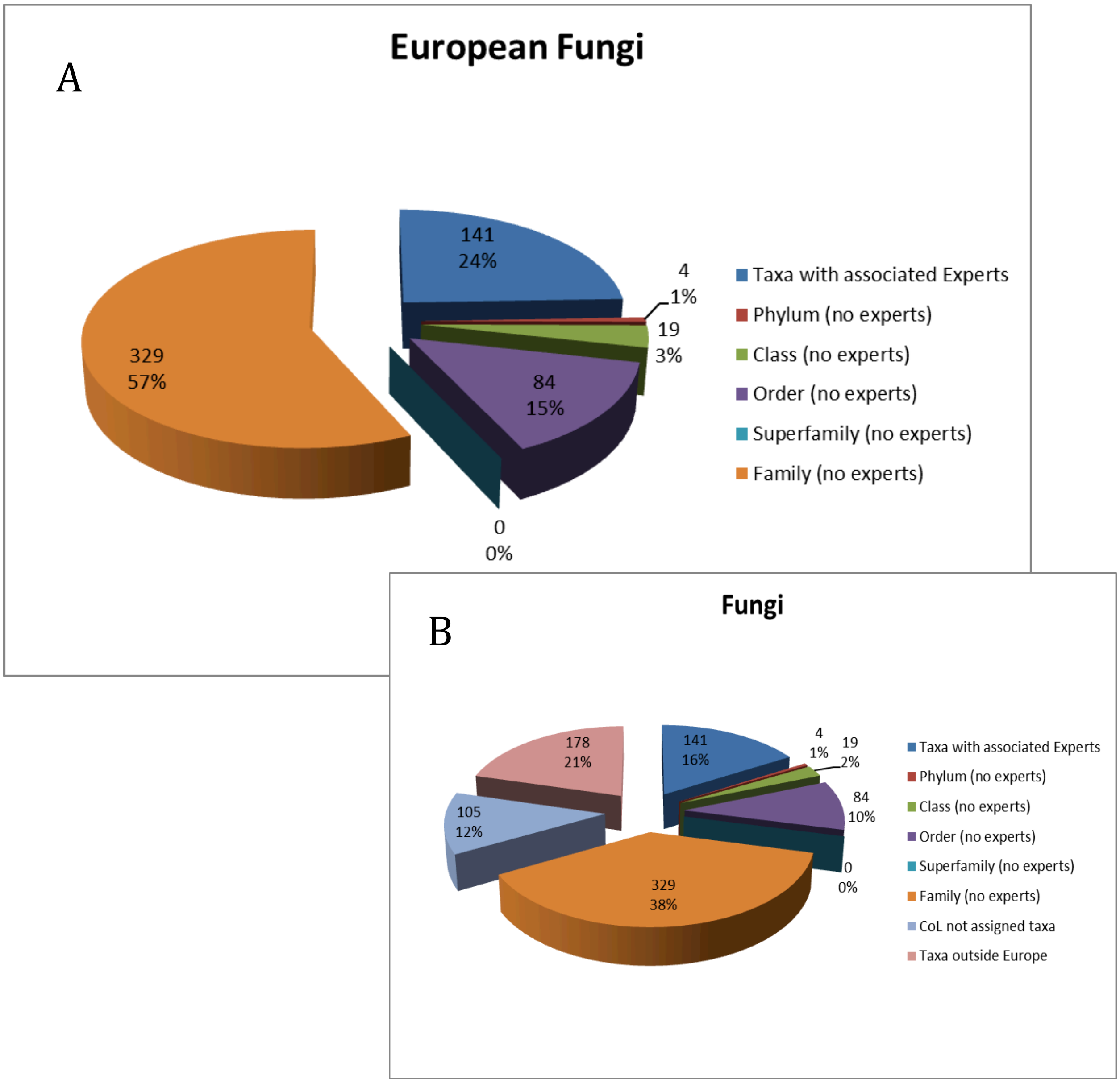

Figure 4. A: Kingdom Fungi based on the CoL classification scheme illustrating the relatively proportion of gaps to fill in relation to editors. B: The European Fungi shows the figures minus the worldwide taxa included in CoL.

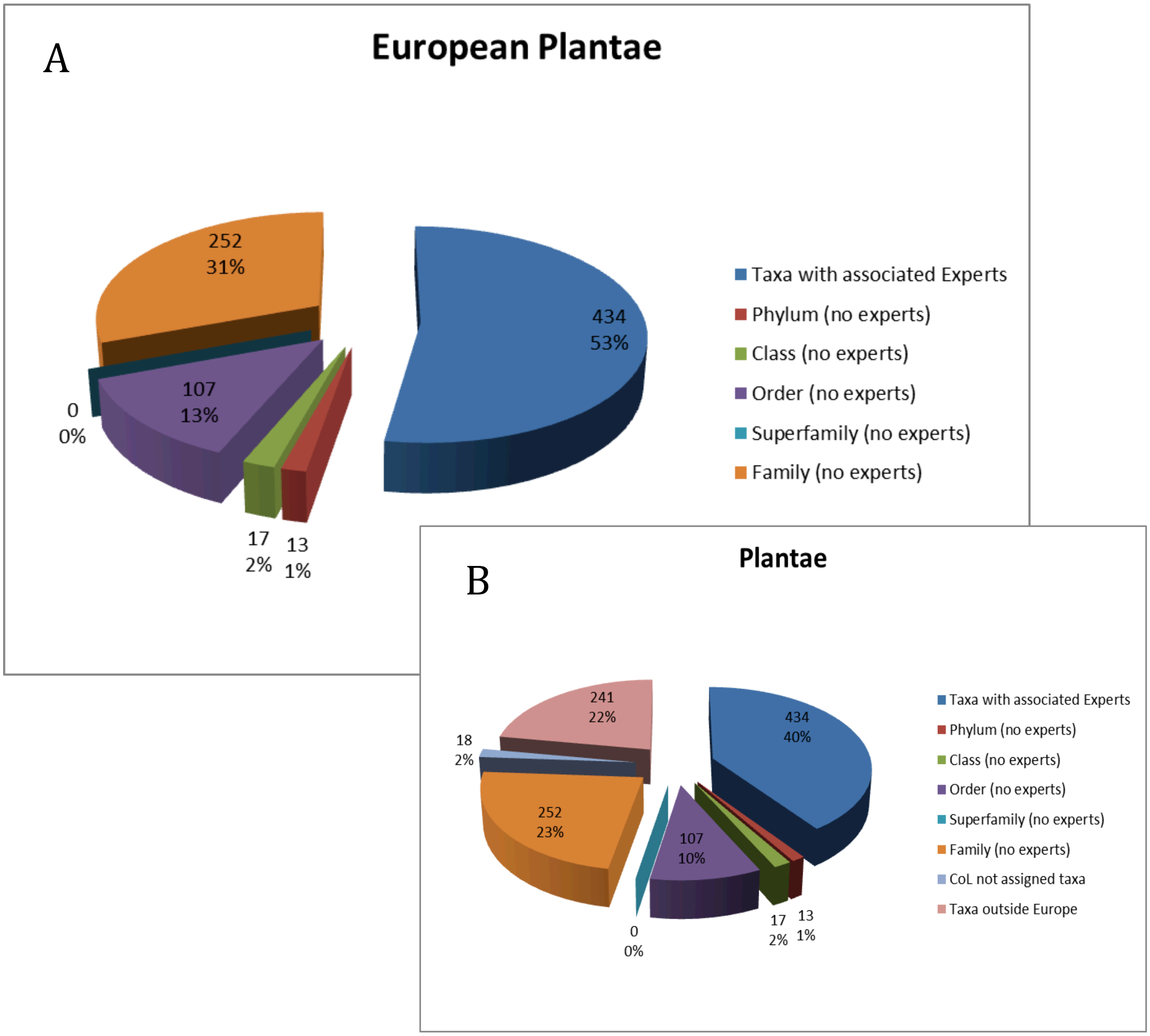

Figure 5. A: Kingdom Plantae based on the CoL classification scheme illustrating the relatively proportion of gaps to fill in relation to editors. B: The European Plantae shows the figures minus the worldwide taxa included in CoL.

Table 3. This table illustrates the number of taxonomists with interests in families which may either fill a gap or become a subeditor or provide a backup list of potential future editors.

Catalogue of life - taxa	Edit Experts	Catalogue of life - taxa	Edit Experts
**Taxa with no editor within the European registers**			
**Family Hominidae**	**Niels Bonde**	**Family Platydesmidae**	**William Shear**
**Family Characidae**	**Ignacio Doadrio**	**Superfamily Phytoseioidea**	**Ineta Salmane**
**Taxa with existing but different editors within the European registers**			
**Class Demospongiae**	Manuel Maldonado-Barahona, MariaJesus Uriz	**Family Helicidae**<br>**Family Neritidae**<br>**Family Melanopsidae**<br>**Family Hydrobiidae**	Zoltan Feher
**Phylum Porifera**	Javier Sanchez-Mfontenla, John Harper, Claire Valentine	**Order Pulmonata**	Bas Kokshoorn, Jackie van Goetham, Teresa Aparicio, Zoltan Feher
**Class Calcarea**	MariaJesus Uriz	**Family Vermetidae**<br>**Family Architectonicidae**	Rudiger Bieler
**Family Schistosomatidae**	Claire Standley	**Family Naticidae**	Thomas Hudsken
**Family Monocotylidae**	Joost van der Burg	**Order Basommatophora**	HansHenrik Bruun, Claire Standley,
**Class Trematoda**	Thomas Cribb	**Superfamily Trochacea**	Suzanne Williams
**Phylum Platyhelminthes**	Eileen Harris, Tom Artois	**Class Gastropoda**	Patrice Bail, Rafael Zardoya, Thomas Huelsken
**Family Trichoplacidae**	Maria Balsamo	**Family Tonnidae**<br>**Family Ficidae**<br>**Family Turritellidae**<br>**Family Pinnidae**<br>**Family Siliquariidae**	Chris Vos
**Phylum Nemata**	Oleksandr Holovachov, Andrea McEnvoy, Johan Coenjaerts, Oleksandr Holovachov	**Family Octopodidae**	Roger Villanueva
**Phylum Myxozoa**	B. Okamura	**Class Cephalopoda**	Dieter Korn, Michael Vecchione
**Phylum Mollusca**	Bram Breure, A MacLellan, Johan Coenjaerts, S. Long,	**Order Veneroida**	Jan Johan ter Poorten
**Phylum Hemichordata**	Billie J Swalla, Claire Mellish	**Order Unionoida**	Henning Scholz
**Phylum Gnathostomulida**	Martin Vinter Soerensen	**Family Propeamussiidae**<br>**Family Pectinidae**	Henk Dijkstra
**Phylum Gastrotricha**<br>**Family Turbanellidae**<br>**Family Proichthydiidae**<br>**Family Neogosseidae**<br>**Family Dichaeturidae**<br>**Family Dasydytidae**<br>**Family Chaetonotidae**	Maria Balsamo	**Phylum Cnidaria**	Rachel Hammer
**Class Holothuroidea**	Dider Vandenspiegal	**Family Testudinidae**	Rodham Tulloss
**Class Echinoidea**	Bruno David	**Order Testudines**	Sandra Chapman
**Class Cubozoa**<br>**Order Trachymedusae**<br>**Order Limnomedusa**	Allen G Collins	**Family Scincidae**	Peter Vetz
**Class Hydrozoa**	Peter Schuchert	**Family Colubridae**	Zoltan Nagy

Catalogue of life - taxa	Edit Experts	Catalogue of life - taxa	Edit Experts
**Order Alcyonacea**<br>**Family Nephtheidae**	Leen Ofwegenvan	**Order Cumacea**	Gary Anderson
**Class Anthozoa**	Daphne Fautin	**Order Bathynellacea**	AnaIsabel Camacho
**Order Trichoptera**	Michael Stoltze	**Order Amphipoda**	Dirk Platvoet,<br>Claude DeBroyer,<br>Johan Coenjaerts*,<br>Mark J Costello,<br>Oliver Coleman
**Order Raphidioptera**<br>**Family Diadocidiidae**<br>**Order Neuroptera**<br>**Order Mecoptera**<br>**Family Ditomyiidae**<br>**Family Keroplatidae**<br>**Family Mycetophilidae**<br>**Family Bolitophilidae**	Jan Ševčik	**Family Pentatomidae**<br>**Family Hebridae**<br>**Family Veliidae**<br>**Family Gerridae**<br>**Family Coreidae**	Romain Garrouste
**Order Plecoptera**	Louis Bouman ,<br>Maria-Angeles Puig,<br>Romolo Fochetti	**Order Orthoptera**<br>**Family Mantispidae**	Kirill Mark Orci,<br>Maximilian Weigend
**Order Phthiraptera**	Vincent Smith	**Family Tortricidae**	Sandrine Vlenberg
**Order Mecoptera**	W. Hogenes	**Family Papilionidae**	Michael Stoltze
**Family Chrysopidae**	Ladislav Jedlicka	**Family Micropterigidae**<br>**Family Gelechiidae**	Ole Karsholt
**Order Neuroptera**	Michael Ohl	**Family Gracillariidae**	Willy DePrins
**Order Lepidoptera**	Ian Kitching,<br>Camiel Doorenweerd,<br>Geoff Martin,<br>Hans Christof Zeller-Lukashort,<br>John Chainey,<br>Kevin tuck,<br>Koen Maes,<br>Francesca Barbero,<br>Martin Honey,<br>Michael Stoltze,<br>PerStadel Nielsen,<br>Petr Herman,<br>Willy DePrins	**Order Hymenoptera**	D Notton,<br>Alain Pauly,<br>Joseluis Nieves-Aldrey,<br>Claire Villemant,<br>Juan Carlos Monje,<br>Michael Ohl,<br>Suzanne Ryder
**Order Isoptera**	Yves Roisin	**Order Hemiptera**	Gernot Kunz,
**Family Platygastrida**	PeterNerup Buhl	**Family Siphlonuridae**	Eva Engblom
**Family Sphecidae**	Michael Ohl	**Family Tingidae**	Eric Guilbert
**Family Apidae**	Isabel Calabuig		
**Superfamily Aphidoidea**<br>**Family Aphididae**	Juanm Nieto	**Order Harpacticoida**	Andrea McEnvoy,<br>Kai Horst George,
**Superfamily Pentatomoidea**	Dominique Pluot-Sigwalt	**Family Ulidiidae**	John T Smit
**Order Ephemeroptera**	Maria-Angeles Puig	**Order Cyclopoida**<br>**Order Calanoida**	Andrea McEnvoy
**Family Cyclopidae**<br>**Family Diaptomidae**	Maria Hołyńska	**Order Tanaidacea**<br>**Order Mysida**<br>**Order Lophogastrida**<br>**Order Cumacea**	Gary Anderson,
**Order Tanaidacea**	Magda Blazewicz	**Family Cirolanidae**	Julia HM Kouwenberg
**Order Stomatopoda**	Pere Abello	**Family Processidae**	Pierre Noel
**Order Isopoda**	Angelika Brandt,<br>Gary Poore,<br>George DF Wilson,	**Family Carabidae**	Peer Schnitter, RFFL Felix,<br>Terry Erwin
**Family Galatheidae**<br>**Family Chirostylidae**	Patricia Cabezas	**Family Cryptochiridae**	Sancia van der Meij

Catalogue of life - taxa	Edit Experts	Catalogue of life - taxa	Edit Experts
**Order Decapoda**	Keith Crandall,<br>Enrique MacPherson,<br>Ferran Palero,<br>Gary Poore,<br>Keith Crandall,<br>Pere Abello,<br>Sarah Gerken	**Order Diptera**	Jan Sevcik,<br>Axel Ssymank,<br>Christophe Daugeron,<br>Hans-Peter Tschorsnig,<br>Jolana Tatocova,<br>Ladislav Roller,<br>Eva Bulankova,<br>Litta Greve,<br>Janet Beccaloni,<br>Libor Mazánek
**Family Lampropidae**	Zoltan Nagy	**Family Gracillariidae**	Willy DePrins
**Family Micropterigidae**<br>**Family Gelechiidae**	Ole Karsholt	**Family Platygastridae**	PeterNerup Buhl
**Family Anthomyzidae**<br>**Family Aulacigastridae**<br>**Family Asteiidae**	Jindrich Rohácek	**Family Tenebrionidae**	Wolfgang Schawaller
**Family Asteiidae**	László Papp,		
**Family Calliphoridae**	AnaIsabel Martinez-Sanchez,<br>Knut Rognes,<br>Zoe JO Adams	**Family Agromyzidae**<br>**Family Asilidae**	Michael Martinez
**Family Camillidae**<br>**Family Carnidae**	László Papp,	**Family Anthomyiidae**	Michael Aukland,<br>Verner Michelsen
**Family Solenofilomorphidae**	Ronni Lindsgaard	**Phylum Arthropoda**	Claire Mellish
**Order Opisthopora**	Emma Sherlock,<br>Phillip Boegh	**Class Arachnida**	Gunnar R. Hansen,<br>Jason Dunlop
**Order Astigmata**	Anne Baker	**Order Protura**	Javier Arbea,<br>Julia Shrubovych,
**Family Ologamasidae**<br>**Family Rhodacaridae**<br>**Family Ascidae**<br>**Family Digamasellidae**<br>**Family Parantennulidae**<br>**Family Rhinonyssidae**<br>**Family Varroidae**	Ineta Salmane	**Class Insecta**	Malcolm Scoble
**Family Opilioacaridae**	Jochen Martens,<br>Ruud vander Weele	**Order Blattodea**	Marco Bardiani
**Family Canthyloscelidae**<br>**Family Carnidae**	Jindrich Rohácek	**Order Coleoptera**	Christine Taylor,<br>Jorge Miguel Lobo,<br>Christine Taylor
**Class Branchiopoda**<br>**Family Sididae**	Laszlo Forro	**Family Buprestidae**	Charles Bellamy
**Family Hutchinsoniellidae**	Lasxlo Danyi	**Family Byrrhidae**<br>**Family Dryopidae**	Fedor Ciampor
**Family Spinothecidae**	H Enghoff	**Family Brentidae**	Luca Bartolozzi
**Family Cerylonidae**<br>**Family Cucujidae**<br>**Family Nitidulidae**	Piotr Tykarski	**Family Attelabidae**<br>**Family Anthribidae**<br>**Family Brentidae**<br>**Family Curculionidae**<br>**Family Nemonychidae**	Chris Lyal
**Family Coccinellidae**<br>**Family Endomychidae**	Wioletta Tomaszewska	**Family Staphylinidae**	Gyorgy Makranczy,<br>Volker Putnz
**Family Curculionidae**	Nikolai Yunakov	**Family Hydraenidae**	Fedor Ciampor
**Family Scarabaeidae**	Jason Maté,<br>Sergi Tarasov	**Family Dolichopodidae**	Marc Pollet,<br>Patrick Grootaert

Catalogue of life - taxa	Edit Experts	Catalogue of life - taxa	Edit Experts
**Family Meloidae**	Mario Garcia-Paris	**Family Oestridae**	Jan Minar
**Family Ceratopogonidae**	Ivan Országh,<br>A Jenny Mordue,<br>Radoslav Mraz	**Family Culicidae**	Keith R Snow,<br>Magdalena Zarowiecki,<br>Shelly Cook,<br>Verner Michelsen
**Family Ditomyiidae**<br>**Family Hesperinidae**<br>**Family Keroplatidae**<br>**Family Mycetophilidae**<br>**Family Ptychopteridae**<br>**Family Bolitophilidae**	Dimitar N Bechev	**Family Simuliidae**	Ladislav Jedlicka,<br>Jozef Halgos,<br>Daniela Illesova,<br>Viera Stloukalova,<br>Roger W. Crosskey
**Family Empididae**<br>**Family Hybotidae**	Milan Chvála,<br>Patrick Grootaert,	**Family Tephritidae**<br>**Family Syrphidae**	John T Smit,
**Family Ephydridae**	Tadeusz Zatwarnicki	**Family Tethinidae**	Lorenzo Munari,
**Family Lauxaniidae**	Milan kozanek,	**Family Mycetophilidae**	E. McAlister,<br>Vladimír Košel
**Family Lonchopteridae**	Jean-Paul Haenni	**Family Pipunculidae**	Milan kozanek,
**Family Megamerinidae**<br>**Family Micropezidae**<br>**Family Milichiidae**<br>**Family Tanypezidae**	Jindrich Rohácek	**Family Sciomyzidae**<br>**Family Sepsidae**<br>**Family Sphaeroceridae**	Lorenzo Munari,
**Family Milichiidae**<br>**Family Odiniidae**<br>**Family Asteiidae**<br>**Family Camillidae**<br>**Family Sphaeroceridae**<br>**Family Carnidae**	László Papp	**Family Heleomyzidae**<br>**Family Empididae**<br>**Family Hybotidae**<br>**Family Platypezidae**<br>**Family Pipunculidae**<br>**Family Stratiomyidae**<br>**Family Lauxaniidae**	Ruud vander Weele
**Family Muscidae**	Verner Michelsen,<br>Paul Beuk	**Family Phoridae**<br>**Family Platypezidae**	Sabine Prescher
**Family Psychodidae**	Louis Bouman	**Family Sciaridae**	Joachim Holstein
**Family Scathophagidae**	Verner Michelsen,	**Family Tachinidae**	Joachim Ziegler
**Family Scatopsidae**	Jean-Paul Haenni,<br>Milan kozanek	**Family Sepsidae**	Jean-Paul Haenni,<br>Rudolf Meier,
**Family Sphaeroceridae**	Jindrich Rohácek,	**Family Stratiomyidae**	Jean-Paul Haenni,<br>Rudolf Rozkošný,
**Family Syrphidae**	Gerard Pennards,<br>Snezana Radenkovic,<br>Pierre Mille	**Family Tephritidae**	Massimiliano Virgilio,<br>Milan kozanek,<br>Petr Herman

Catalogue of life - taxa	Bioinformatics specialists	Catalogue of life - taxa	Bioinformatics specialists
**Family Apidae**	Willy DePrins	**Phylum Arthropoda<br>Family Opilioacaridae**	Adriano B. Kury,
**Family Anthomyzidae<br>Family Aulacigastridae<br>Family Asteiidae**	E. Budrys	**Phylum Arthropoda<br>Family Opilioacaridae**	Reinhold Loch,
**Phylum Arthropoda<br>Class Arachnida<br>Family Spintheridae<br>Family Olpiidae<br>Family Opilioacaridae**	Abel Pérez González,	**Phylum Arthropoda<br>Family Spinothecidae<br>Family Spintheridae<br>Family Opilioacaridae**	William Shear
**Phylum Arthropoda<br>Family Opilioacaridae**	Luis Acosta,	**Phylum Arthropoda<br>Class Insecta<br>Order Coleoptera<br>Family Opilioacaridae**	Matthew Gimmel
**Phylum Arthropoda<br>Family Opilioacaridae**	Theo Blick,	**Phylum Arthropoda<br>Family Spintheridae<br>Family Opilioacaridae**	Peter J. Schwendinger,

Configuration History			
**Version No.**	**Date**	**Changes made**	**Author**
0.1	17 March 2010	First draft and discussions on method	RN et al.
0.4	01 February 2011	Completion of delivery of final versions of expert files by checklist custodians and concluding mapping to CoL most recent higher classification	RN<br>(supported by PESI WP5 & WP6)
0.5	9 March 2011	Improved draft circulated for internal review	RN
0.6	13 March 2011	Revision by PESI partners	CH, MC, WA, JK
0.7	16 March 2011	Minor corrections	RN
0.8	17 March 2011	Minor corrections	JK
0.9	18 March 2011	Final draft of first version	RN
1.0	18 March 2011	Final preparation for submission	YdJ
