# Supplementary material for: Fauna Europaea – all European animal species on the web
Source: Biodivers Data J. 2014 Sep 17;(2):e4034. doi: 10.3897/BDJ.2.e4034 (PMC4206781; doi:10.3897/BDJ.2.e4034)
Supplement: Supplementary material 13 — Fauna Europaea Guidelines for NAS validation [file biodiversity_data_journal-2-e4034-s013.pdf]

Guidelines for NAS Validation

Yde de Jong & Melina Verbeek

Version 6

Guidelines for the Fauna Europaea validation process

Data flow

Below an overview of the considered data flow during the FaEu validation process:

1. Export of the data sets from the Fauna Europaea database taxon-wise clustered per Group Coordinator (GC) coverage.
2. Distribution of around sixty data files to the FaEu Focal Points on CD-ROM.
3. Cross-checking (validation) of the data files by local experts focussed on the related geographic context. Preparing improved local versions of the data files
4. Transfer of data files of all FaEu - NAS Focal Points to the FaEu - NAS coordinator at MIZPAN (Warsaw) and of non - NAS members to the FaEu project bureau.
5. Data set collation and administration. Integration of all resulting comments into one data file for each GC.
6. Delivery of validated data files to the FaEu project bureau Amsterdam (for FaEu - NAS coordinator).
7. Distribution of the data files to the relevant GCs.
8. Verification and accord by GCs of the resulted comments. Improving and updating of data files also including the GCs own additions.
9. Re-sending of data files back to the FaEu project bureau Amsterdam. Data set collation and administration by FaEu project bureau Amsterdam.
10. Import of data sets into the FaEu database. Release of next version of Fauna Europaea.

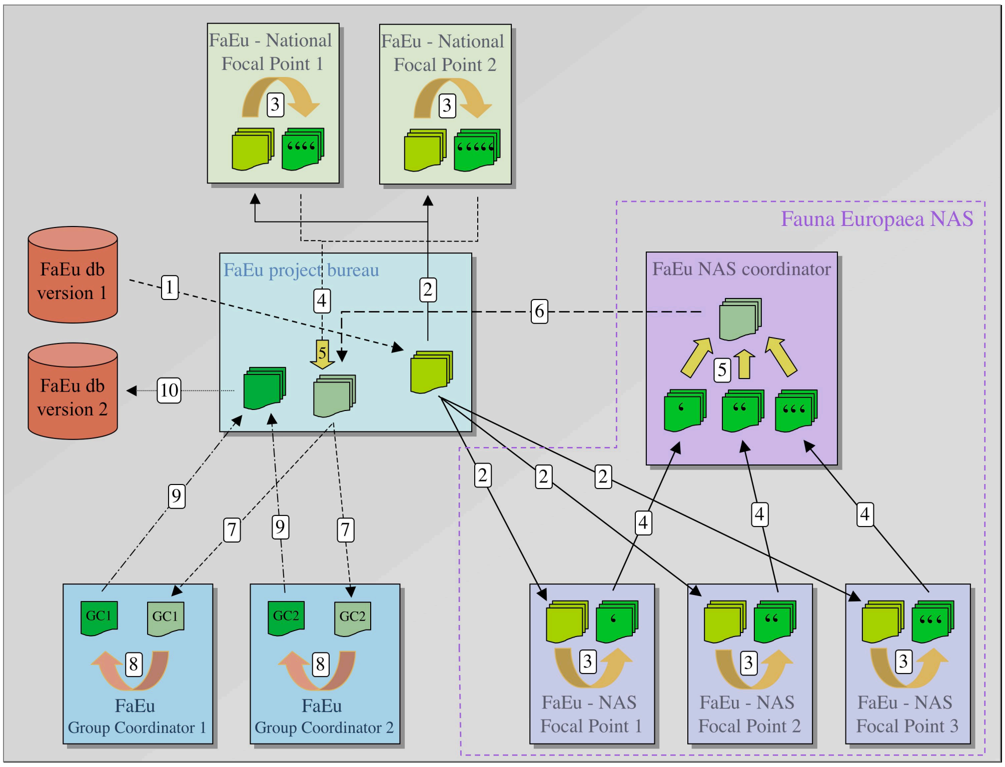

Geographic context

In general all Fauna Europaea NAS partners have to cover their own country. Some Fauna Europaea NAS partners offered also to take care of the validation of other East European countries not included within the Fauna Europaea NAS project at the moment (to be discussed with the project office).

Commenting the data files

To optimise the validation process it is important to follow strict rules when entering suggestions for modification into the data files. This contains:

1. No modifications can be made within the *Hierarchy*, *Taxonomy* and *References* sheets.
2. All suggestions for modification of the *Hierarchy*, *Taxonomy* and *References* sheets have to be listed within the *Reviewing* sheet, referring to the relevant sheet, row and column followed by an outline of the suggested modification:

	A	B	C	D	E	F	G
1	Reviewing						
2	Validator / Reviewer			Record			Comment / Suggested modification
3	Focal Point	Reviewer	Contact info	Sheet	Row	Column(s)	
4	Poland	Wieslaw Bogdanowicz	wieslawb@miiz.waw.pl	Taxonomy	356	B & D	Blabla1
5	Poland	Wieslaw Bogdanowicz	wieslawb@miiz.waw.pl	Faunistic	10	BI	Blabla2
6	......	......	......	......	......	......	......

3. Within the *Faunistic* sheet modifications can only be made in the columns dealing with the distribution within Europe and only for those areas covered by your own country (see above). Typing the proposed correction 'P' into the relevant cell after the original value 'A' with an arrow '–>' in between (so A–>P), using, a **red-bold** font and a light-yellow background, will show suggestions for modification:

	BB	BC	BD	BE	BF	BG	BH	BI	BJ	BK	BL	BM	BN	BO	BP	BQ	BR	BS	BT	BU
1																				
2																				
3	PL	PT-AZO	PT-MDR	PT-POR	PT-SEL	RO	RU-FJL	RU-KGD	RU-NOZ	RU-RUC	RU-RUE	RU-RUN	RU-RUS	RU-RUW	SE	SI	SK	SM	TR-TUE	UA
4																				
5																				
6	P	A	P	P	A	A	P	P	P	P	P	P	P	P	A	A	A	A	A	P?
7	P	A	P	P	P	A	P?	P	P	P	P	P	P	P	A	A	A	A	A	P?
8	P	A	A	P	P	P	P?	P	P	P?	P	P	P	P	A	A	A	A	A	P?
9	P	A	A	P	A	A	P	P	A	P	P	P	P	P	A	A	A	A	A	P?
10	P	A	P	P	P	A	P	A	P	P	P	P	P	P	A	A	A	A	A	P
11	A	A	P	P	P	P	A	P	P	A	P	P	P	P	A	A	A	A	A	P
12	P	A	A	P	A	A	P	P	A	A	P	P	P	P	A	A	A	A	A	P?
13	P?	A	P	P	P	A	P	A	P	A	P	P	P	P	A	A	A	A	A	P?

Original sheet

	BB	BC	BD	BE	BF	BG	BH	BI	BJ	BK	BL	BM	BN	BO	BP	BQ	BR	BS	BT	BU
1																				
2																				
3	PL	PT-AZO	PT-MDR	PT-POR	PT-SEL	RO	RU-FJL	RU-KGD	RU-NOZ	RU-RUC	RU-RUE	RU-RUN	RU-RUS	RU-RUW	SE	SI	SK	SM	TR-TUE	UA
4																				
5																				
6	P	A	P	P	A	A	P	P	P	P	P	P	P	P	A	A	A	A	A	P?
7	P	A	P	P	P	A	P?	P	P	P	P	P	P	P	A	A	A	A	A	P?
8	P	A	A	P	P	P	P?	P	P	P?	P	P	P	P	A	A	A	A	A	P?
9	P	A	A	P	A	**A -> P**	P	P	A	P	P	P	P	P	A	A	A	A	A	P?
10	P	A	P	P	P	A	P	A	P	P	P	P	P	P	A	A	A	A	A	P
11	A	A	P	P	P	P	A	P	P	A	P	P	P	P	A	A	A	A	A	P
12	P	A	A	P	A	**A -> P**	P	P	A	A	P	P	P	P	A	A	A	A	A	P?
13	P?	A	P	P	P	A	P	A	P	A	P	P	P	P	A	A	A	A	A	P?

Improved for Romania (RO) at rows 9 and 12.

The Fauna Europaea Guidelines for Experts are included at the CD-ROM, but can also been downloaded at:
http://www.faunaeur.org/PARTNERS/DOCUMENTS/DOCS/GUIDELINES/FaEu_Guidelines_v4.0.1.pdf

4. If relevant additional comments on the proposed faunistic modifications (e.g. literature references) can be made at the *Reviewing* sheet. Also suggestions for corrections on areas outside a Focal Point actual scope can either be made at the Reviewing sheet or be communicated to the responsible reviewer (=Focal Point).

   Please make comments on (missing) information resources as concise as possible. If - for instance - an expert apparently completely missed a local checklist, please make the relevant modification within the *Faunistic* sheet, but include only one comment within the *Reviewing* sheet referring to all the concerned (faunistic) rows.

Higher hierarchy

The data sets will be exported from the Fauna Europaea database and distributed as units of one file per Group Coordinator (GC). This means that the taxonomic coverage per file includes the taxonomic treatment of a GC:

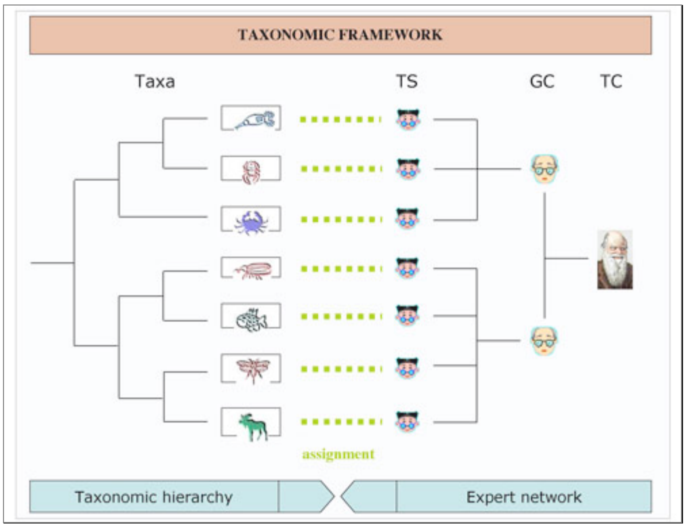

The Fauna Europaea higher hierarchy is included at the CD-ROM, but can also been downloaded at: http://www.zmuc.dk/entoweb/faeu/hierarchy/FaEu_Animalia3.6_publ040517.xls

Files related to Group Coordinators

An overview of the containing data files and their associated Group Coordinators can be found at the attached Fauna Europaea higher hierarchy file (first sheet):

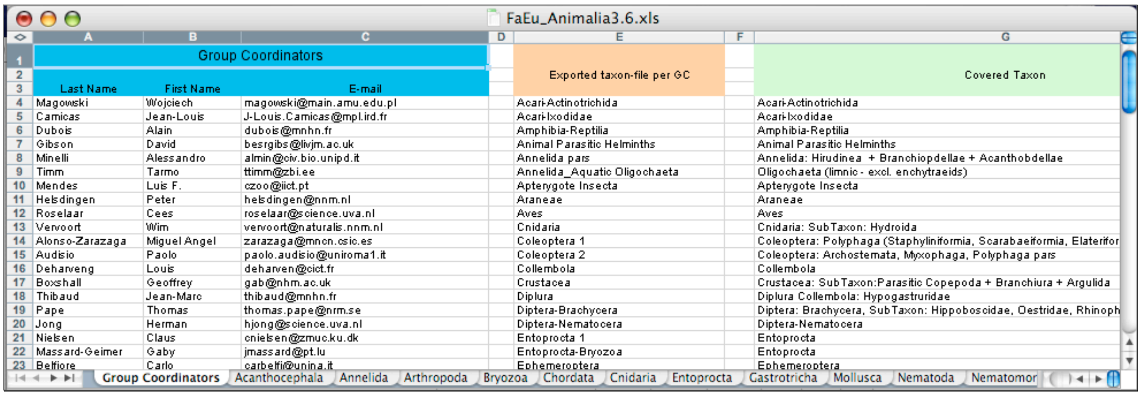

FaEu_Animalia3.6.xls

	Group Coordinators				
	Last Name	First Name	E-mail	Exported taxon-file per GC	Covered Taxon
4	Magowski	Wojciech	magowski@main.amu.edu.pl	Acari-Actinotrichida	Acari-Actinotrichida
5	Camicas	Jean-Louis	J-Louis.Camicas@mpl.ird.fr	Acari-Ixodidae	Acari-Ixodidae
6	Dubois	Alain	dubois@mnhn.fr	Amphibia-Reptilia	Amphibia-Reptilia
7	Gibson	David	besrgibs@livjm.ac.uk	Animal Parasitic Helminths	Animal Parasitic Helminths
8	Minelli	Alessandro	almin@civ.bio.unipd.it	Annelida pars	Annelida: Hirudinea + Branchiopdellae + Acanthobdellae
9	Timm	Tarmo	ttimm@zbi.ee	Annelida_Aquatic Oligochaeta	Oligochaeta (limnic - excl. enchytraeids)
10	Mendes	Luis F.	czoo@iict.pt	Apterygote Insecta	Apterygote Insecta
11	Helsdingen	Peter	helsdingen@nnm.nl	Araneae	Araneae
12	Roselaar	Cees	roselaar@science.uva.nl	Aves	Aves
13	Vervoort	Wim	vervoort@naturalis.nnm.nl	Cnidaria	Cnidaria: SubTaxon: Hydroida
14	Alonso-Zarazaga	Miguel Angel	zarazaga@mncn.csic.es	Coleoptera 1	Coleoptera: Polyphaga (Staphyliniformia, Scarabaeiformia, Elaterifor
15	Audisio	Paolo	paolo.audisio@uniroma1.it	Coleoptera 2	Coleoptera: Archostemata, Myxophaga, Polyphaga pars
16	Deharveng	Louis	deharven@cict.fr	Collembola	Collembola
17	Boxshall	Geoffrey	gab@nhm.ac.uk	Crustacea	Crustacea: SubTaxon:Parasitic Copepoda + Branchiura + Argulida
18	Thibaud	Jean-Marc	thibaud@mnhn.fr	Diplura	Diplura Collembola: Hypogastruridae
19	Pape	Thomas	thomas.pape@nrm.se	Diptera-Brachycera	Diptera: Brachycera, SubTaxon: Hippoboscidae, Oestridae, Rhinoph
20	Jong	Herman	hjong@science.uva.nl	Diptera-Nematocera	Diptera-Nematocera
21	Nielsen	Claus	cnielsen@zmuc.ku.dk	Entoprocta 1	Entoprocta
22	Massard-Geimer	Gaby	jmassard@pt.lu	Entoprocta-Bryozoa	Entoprocta
23	Belfiore	Carlo	carbelfi@unina.it	Ephemeroptera	Ephemeroptera

Group Coordinators | Acanthocephala | Annelida | Arthropoda | Bryozoa | Chordata | Cnidaria | Entoprocta | Gastrotricha | Mollusca | Nematoda | Nematomor

Higher Hierarchy and Taxonomic Framework

Details about the Fauna Europaea higher hierarchy and taxonomic framework can be found at the Fauna Europaea expert database:

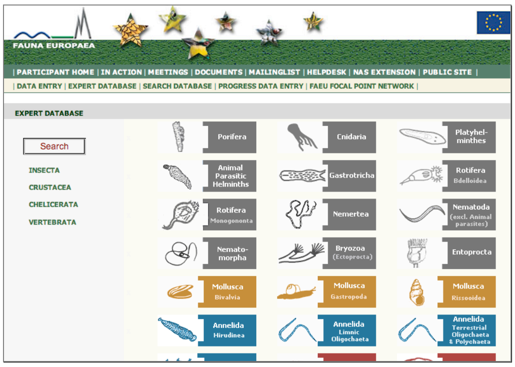

http://www.faunaeur.org/PARTNERS/EXPERT_DATABASE/experts.htm

Integration of data files

For the NAS coordinator (NAS countries) and Amsterdam project bureau (non NAS countries) there will be a major effort around the first two weeks of September to integrate all data files, which includes (1) copying the relevant faunistic columns of all partners into one single *Faunistic* sheet and (2) copying all review rows into one single *Review* sheet as well (see both figure below).

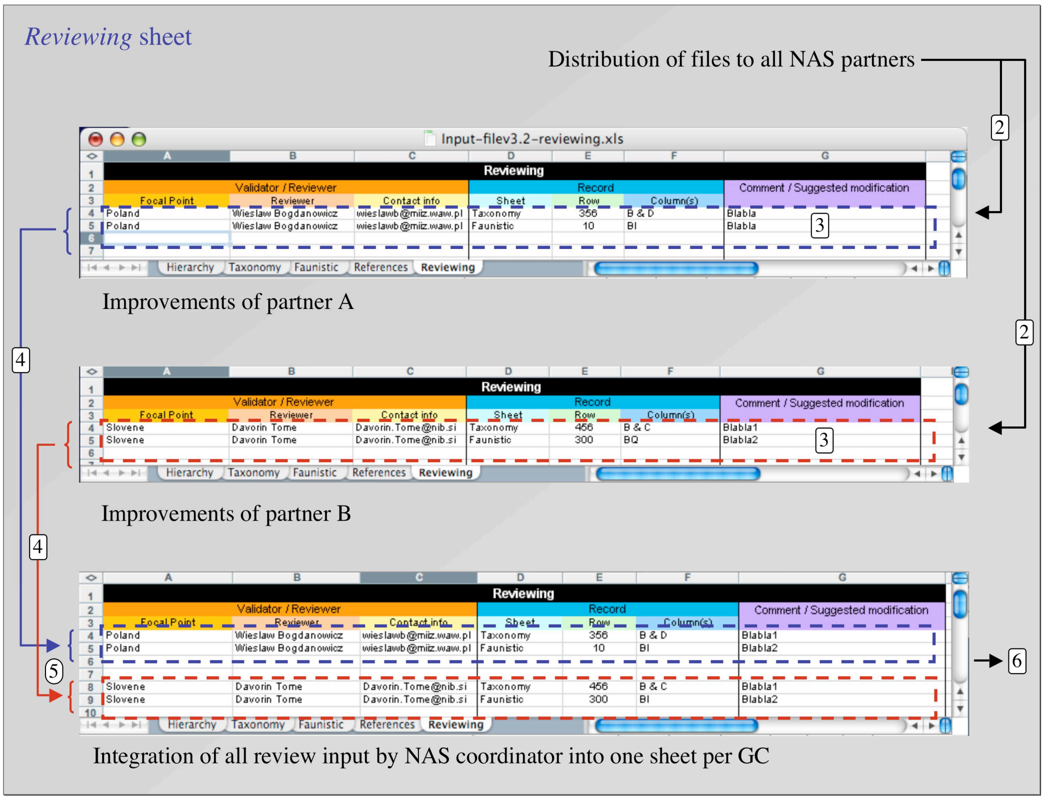

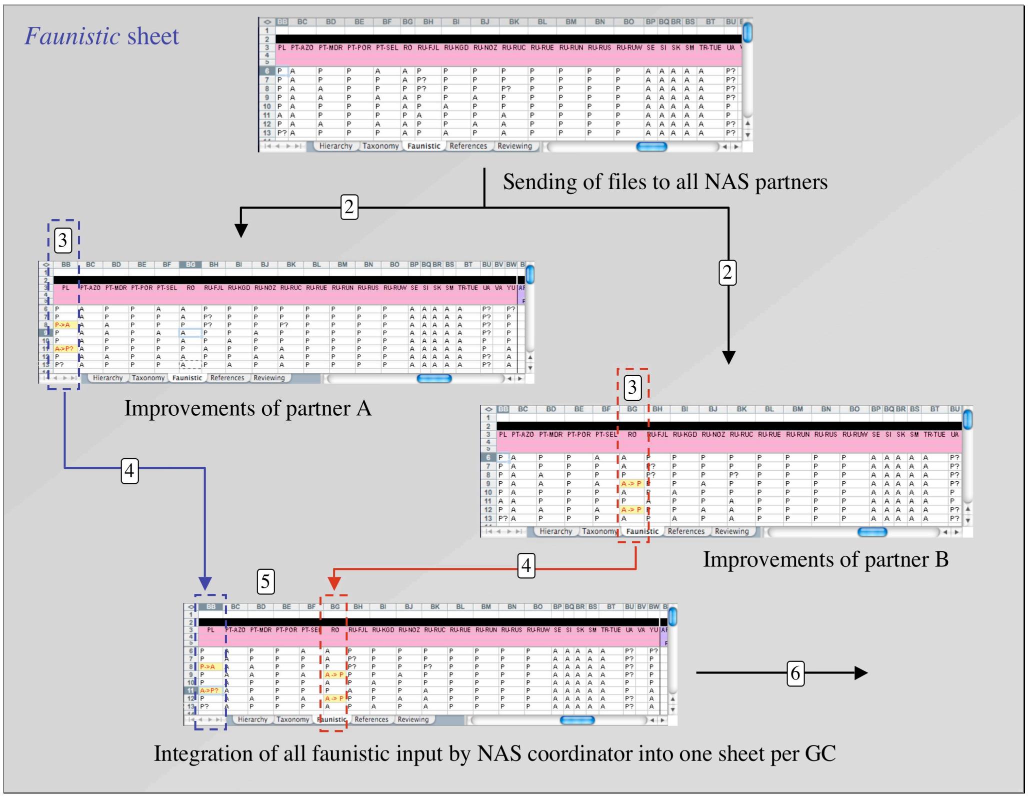

Time schedule

The considered data flow time schedule:

Date / period	Event number	Circumscription
End of May	1	Export of data files from FaEu database
Beginning of June	2	Distribution of data files to Fauna Europaea National Focal Points
June to September	3	Local validation at National Focal Points
Beginning of September	4	Transfer of data files to MIZPAN (NAS) or Amsterdam (non-NAS)
Beginning of September to Mid-September	5	Integration of data files by MIZPAN (NAS) or Amsterdam (non-NAS)
Mid-September	6 & 7	Delivery of data files to GCs via project bureau
Mid-September to Mid-October	8	Verification of the suggested comments
Mid-October to November	9 & 10	Return of data files to project bureau and import into the database

Additional comments

- The *Hierarchy* sheet, *Taxonomy* sheet and *Reference* sheet are read-only (no data handling will be allowed). The *Faunistic* sheet is read-only with respect to the taxonomic columns. For local use (excluding non relevant country columns and preparing selected lists for local experts) the data can be copy-paste to a new spreadsheet and organised in a preferred way (first un-merge all cells). However, the final (collated) results have to be sent to the NAS coordinator (for NAS countries) and Amsterdam project bureau (for non-NAS countries) within the original format!
- Within the *Faunistic* sheet modification are only allowed within the authorised column(s) of each partner, in most cases including only one particular country. Entering data within other columns makes no sense because they are split out during the integration and transformations of the files (step 5). Relevant suggestions for other countries can be included within the *Review* sheet or e-mailed to the involved National Focal Point partner.
- For some data files the faunistic data are incomplete or missing (e.g. Rotifera-Monogononta). Completing this data is optional, but will be very welcomed.
- If a national checklist not already exists, partners are strongly advised to set up a network of local experts and to share reviewing tasks.
- Not all Group Coordinators already delivered their files (see the last GCs listed at the FaEu higher hierarchy file). These files will be sent later on by e-mail.
- Please notice the attached Fauna Europaea Guidelines for Experts or outdated with respect to the higher hierarchy.

Disclaimer

The Fauna Europaea National Focal Point partners and their involved experts have to respect the Fauna Europaea copyright agreements, which prohibits the spread of Fauna Europaea data apart from the official (public) release(s).
