# Supplementary material for: Fauna Europaea – all European animal species on the web
Source: Biodivers Data J. 2014 Sep 17;(2):e4034. doi: 10.3897/BDJ.2.e4034 (PMC4206781; doi:10.3897/BDJ.2.e4034)
Supplement: Supplementary material 14 — Fauna Europaea Description of Work [file biodiversity_data_journal-2-e4034-s014.pdf]

Fauna Europaea

DoW

Description of Work

Annex I

Version: 22 November 1999

ANNEX I

DESCRIPTION OF WORK

FAUNA EUROPAEA

PROPOSAL No: EVR1-1999-00021

CONTRACT No: No: EVR1-CT1999-20001

**PROJECT COORDINATOR:**	
**Universiteit van Amsterdam**	**NL**
**CONTRACTORS:**	
**Koebenhavns Universitet**	**DK**
**Museum National d’Histoire Naturelle**	**F**

DURATION: 48 MONTHS

Date: 22 November 1999

TABLE OF CONTENTS

1 SUMMARY

2 SCIENTIFIC/TECHNICAL OBJECTIVES AND INNOVATION

3 PROJECT WORKPLAN
(1) Technical implementation plan
(2) Project planning and time table
(3) Graphical presentation of the projects components
(4) Work packages

4 CONTRIBUTION TO OBJECTIVES OF PROGRAMME/CALL

5 COMMUNITY ADDED VALUE AND CONTRIBUTIONS TO EU POLICIES

6 CONTRIBUTION TO COMMUNITY SOCIAL OBJECTIVES
(1) Quality of Life, Health and Safety of the Citizens
(2) Environment and Natural Resources
(3) Employment, Education, Training and Working Conditions

7 ECONOMIC DEVELOPMENT AND S&T PROSPECTS
(1) Exploitation and Dissemination of Results, IPR
(2) Economic Growth
(3) Scientific and technological Prospects

8 THE CONSORTIUM
(1) Overview of the Consortium
(2) Description of the Participants

9 PROJECT ADMINISTRATION
(1) Project Management
(2) Manpower Matrix

10 LIST OF REFERENCES AND RELATED PROJECTS
(1) References
(2) Related projects
(3) Project Cluster Participation
(4) Acronyms

1 SUMMARY

Problems to be solved
The assessment of biodiversity, monitoring changes, sustainable exploitation of biodiversity, and much legislative work depends upon validated knowledge of its components: the taxonomic diversity. The same applies for areas such as human health, agriculture, or freshwater quality. However, the required data are not directly available or in the requested format. Information and expertise on the European animal species is scattered around numerous public and private organisations, they are in different formats and standards, and are of unequal quality.
The Community Biodiversity Strategy of the European Commission recognises the current incomplete state of knowledge at all levels concerning biodiversity. It asks for establishing networks between European centres of excellence in biodiversity research. Fauna Europaea contributes to the European Community Biodiversity Strategy by supporting one of the main themes in the Strategy: the request to identify and catalogue the components of biodiversity, of which a database on the European terrestrial animal taxonomic diversity is a basic tool for science and policies.

Scientific objectives and approach
The Fauna Europaea project will build a public WWW service on the scientific names (with selected additional data) of the present living multicellular European terrestrial and fresh water animal species. As the project is complicated because of the large number of data of different quality and the widely distributed data sources in Europe, the development of the project depends on the contributions of numerous experts. Central in the workplan is the division of the work in three mutually linked main tasks: (1) data collation, (2) data validation, and (3) data management, including supporting information technology. The data originate from different sources, which implies the establishment of routines for easy and manageable ways to update the data, and the development of a dynamic system that allows for changes through time. Priority setting, product presentation and dissemination plans will be discussed with the end-user forum.

Expected impacts
Policies with respect to biodiversity and fauna-related products will be supported by standardized information and thus facilitate communication about the names of animals, thereby ensuring that a name (and its information attributes) is effectively referring to the same species Europe wide. The use of this thesaurus function is increasingly important for numerous reasons, the political, administrative and economic integration being a foremost factor. Users may also select various subsets of information or combine these with other data for many purposes, as for example for the management of animal species or for the use of correct reference data in biotechnology. The Fauna Europaea project will result in an unique overview of the state of art with respect to our knowledge and help to identify gaps in knowledge and expertise. The results of this analysis will contribute to more focussed European research activities and avoid duplication of efforts that otherwise should not be noticed. By networking of researchers, database custodians and users, an unique effort will result in building and maintaining an informatics infrastructure that will support the collation of harmonized and validated taxonomical data, as well as offer access to these data to a wider user community.

2 SCIENTIFIC/TECHNICAL OBJECTIVES AND INNOVATION

The Fauna Europaea project will result in a public WWW service on the scientific names and distribution of the present living multicellular European terrestrial and fresh water animal species, with search/retrieval modes available in each of the formal European languages. It also serves as a directory, while CD-ROM and printed versions of the whole data set of of parts will exploited to finance continuous updating of the service.
With the non-taxonomic users and its broad international scope in mind, the format and content of the FE list will be straightforward and easily understandable to all these (European) users: hierarchy, arrangement of taxa, design and typography, indexes (or user interface and search engine, for a computerized list) will be user friendly.
Taxonomic, thematic and regional experts warrant quality as a prerequisite for adequate service and support.

The European Community Biodiversity Strategy
Fauna Europaea contributes to the European Community Biodiversity Strategy by supporting one of the main themes in the Strategy: the request to identify and catalogue the components of biodiversity, of which a database on the European taxonomic diversity is a basic tool for science and conservation policies. Fauna Europaea addresses this priority by compiling and validating a catalogue. Such an overview does not exist on an European scale, except for a limited group of species within the European Nature Information System (EUNIS) of the EEA – Topic Centre on Nature Conservation. Partial overviews are scattered around Europe in different scientific institutes, while only some countries are working on national information systems. As such, it provides support for further development of the Clearing House Mechanism, the prime vehicle for international information exchange on biodiversity, managed by the European Environment Agency.
Science and policies in regard to biodiversity in Europe depend on a good knowledge of its components. The assessment of biodiversity, monitoring changes, sustainable exploitation of biodiversity, and much legislative work depends upon a validated overview of taxonomic biodiversity, for which Fauna Europaea will provide a major part. End-users of the projects results are involved in the Fauna Europaea network.

As the basic data are not stable – depending on scientific developments, and on changing faunal distributions - Fauna Europaea will be developed as a dynamic system that allows for changes through time. The data originate and will come from different sources, which implies the establishment of routines for easy and manageable ways to update the info.
The Fauna Europaea project builds on the scientific work of a lot of researchers, which partly already have produced partial information systems on specific taxonomic groups or on regional/national faunas. Some ‘Atlas’ projects also developed detailed distributional and other information sources. All these data sets constitute the basis of Fauna Europaea.

The project members are experts and/or custodians of these data. The transfer of data from these different data sources of not related formats requires a dedicated operation management with respect to the organisation and a software entry module. With respect to the latter, data gathering and standardisation will be facilitated by user-friendly software packages as a tool to simplify data entry and the transfer of existing databases. This procedure will support the process to merge and validate the data. The project makes full use of modern electronic tools, also to promote maximal compatibility with other database systems such as the European Register of Marine Species and Species 2000.

Identification of gaps in knowledge and expertise

The Fauna Europaea project will result in an unique overview of the state of art with respect to our knowledge of the European animal species. Within the time frame and budget for this project, it will not be possible to cover all present living European species. Partly, because not only are all species not known, but also because the data are not always based on accepted taxonomies, or not recently scrutinized, or not in the requested digital format. However, as such it will help to identify gaps in knowledge with respect to:

- insufficient information about taxonomic groups (not well studied taxa or disputed taxonomies),
- geographic distributions (lacking or inadequate sampling),
- no databased information or in incorrect formats,
- no available expertise and/or experts.

The objective is finally to report on this analysis and deliver the EU input for regional, national and community research policies.

Innovation

The Fauna Europaea project builds on the scientific work of many scientists, organisations and societies. Their work involved sampling of organisms, desciption of species, elucidating taxonomies, analysing geographic distributions, listing and databasing the results for various groups and/or regions. The European Topic Centre on Nature Conservation of the European Environment Agency, which already initiated the collection on legally protected species at European level (EC Directives and Bern Convention) in the framework of the EUNIS system, has been supportive by drawing conclusions with respect to standards for collecting and harmonising information at the European level. The project will take these into account. Fauna Europaea will only cover those data that can be obtained and processed within the time frame and the budget of the project. The plan is to process at least 70.000 names, with a target of 100.000 names.

It is the first attempt in Europe to collate comprehensive databases on terrestrial animal species (there has been a printed precedent for the fresh water: Limnofauna Europaea). The result will be complementory and goes beyond the EUNIS system, developed by the European Environment Agency, which focusss on a limited selection of species, those legally protected at European level.

The innovative approach to organise the system is perhaps best perceived when realizing that there are probably about 2 to 3 times as much proposed names than there are valid

animal species in Europe. The International Commission on Zoological Nomenclature (ICZN, based in London, UK) issues a Code of Nomenclature that promotes stability and universality by regulating the way scientific names are formed and used. However, it does not provide an umbrella for "authority lists" at a national, regional or global level, as the ICZN does not see its role as infringing on taxonomic opinions on the validity of species. New names proposed under the Zoological Code are compiled annually by the Zoological Record (published by Biosis UK), which provides the source for newly established names, but does not give opinion on the validity of the species denoted by these names. It is precisely the role of this project to provide access to expert opinion on the species of animals living in Europe.

European cooperative project

The project brings experts together from all over Europe. To process the sometimes very different databases in a variety of formats and of unequal quality, requires careful logistics, which will be supported by electronic transfer and a help desk. This approach will also help to develop the mechanisms to promote the future maintenance, continuity and updating of Fauna Europaea.

The European scientific community has discussed at various occasions the strategies of how to organise the research activities to produce a validated information system of the components of the taxonomic biodiversity. The challenge was to promote synchronised activities for the animal, plant, and microbial biodiversity (for both terrestrial and marine biotas), in connection to the various national initiatives, the global developments, and related projects such as the EU funded BioCise project. The Linnean Society of London was helpful in supporting the establishment of network activities, which resulted in the development of an Europe-wide structure for a couple of interrelated projects. The Consortium of European Taxonomic Facilities (CETAF), the consortium of the major European natural history institutions and collections, actively supports all these initiatives.

3 PROJECT WORKPLAN

3.1 Technical implementation plan

Scope of the project

The Fauna Europaea project is complicated because of the large number of data of different quality and the widely distributed data sources in Europe. The development of the project depends on the contributions of numerous experts. The project agreed with experts to contribute their data and expertise without calculating the real costs to assemble the data. Nevertheless, the project will support the delivery of data sets in the requested format by paying a modest sum per delivered species. This implies that the project is restricted to those data that can be collated within its time frame and budget. The project is further restricted with respect to the taxonomic detail and geographical coverage.

Taxonomic detail
The project is limited to present-living multicellular animals (metazoans). Only officially described and valid species will be listed.
The dataset comprises of:

(1) Valid name (incl. status and reference)
(2) List of unambiguous synonyms
    (Synonyms in the information system will be restricted to the original names, the common synonyms, the ones in use and the most common combinations. Inclusion of vernacular names will also follow a pragmatic approach, with at least the names in use in official texts).
(3) List of common name records (optional)
    (Restricted list; mostly if relevant with respect to legislation or comparable formal texts).
(4) Latest taxonomic specialist scrutiny
(5) Source database
(6) Optional / Comment field
    (In this field the species records will be flagged according to possible conservation status, legislative position in the EU (Habitats Directives, Bern Convention), and others).
(7) Family name
(8) References

The classification of the taxa (taxonomic level above the genus level) will only serve as a practical reference, and be kept as simple as possible. In consultation with end-users, the approach of Species 2000 (Biosis) will be studied. Both alphabetical as well phylogenetical organized search modes are to be developed.

Geographical coverage and detail

The project will cover all European terrestrial (and fresh water) animals. This implies that the sea shore is one of the geographical limits, but FaunaEur includes all European islands (in the Atlantic Ocean also Canary islands and Madeira; in the Mediteranean up to Cyprus). To the East, the limit is the Ural, thus restricted to biogeographic 'minimal Europe'. Turkey, except European Turkey, is not a part of the project.

The information about the geographical distribution of species (optional) will at least be on country level (political units), but only if available and in the correct format. The same holds for data on temporal change, as these data only exist for a limited number of species, and are often restricted to a region or not validated. However, if possible, these data will be added.

Structure and methodology of the workplan

Central in the workplan is the division of the work in three main mutually linked activities by the three contractors:

- 1 University of Amsterdam: data management, including supporting information technology,
- 2 University of Copenhagen: data collation
- 3 Muséum National d'Histoire Naturelle: data validation.

Priority setting and product presentation will be discussed with the end-user forum.
The planning and management of the project is organized in the following clusters of workpackages.

Co-ordination

1 Project management and co-ordination
2 Standards, protocols, scope, limits
3 End-user forum

Data structure

4 Design of taxonomic framework and hierarchy
5 Defining and coding of geographical regions
6 Taxonomic coordination

Data collation

7 Tools for data transfer
8 Collation of taxonomic groups
9 Help desk

Data processing

10 Verification and quality control
11 IT database and service management

Production

12 Identification of gaps in data and knowledge, priorities for research
13 Linkages to other registers
14 Publishing the Fauna Europaea register on the WWW
15 Data presentation
16 Dissemination, copyrights and exploitation plans (Technology Implementation Plan)

The success of the project is very much dependent of the cooperation of the European experts involved. The relevant experts have been carefully identified. Nevertheless, as scientific views may differ between the experts, the organisation of the project seperates the collation of data from the validation. Finally, Fauna Europaea will reflect the actual level of taxonomic knowledge. The exploiatation plan will describe the process of future updating.

3-2 Project planning and time table

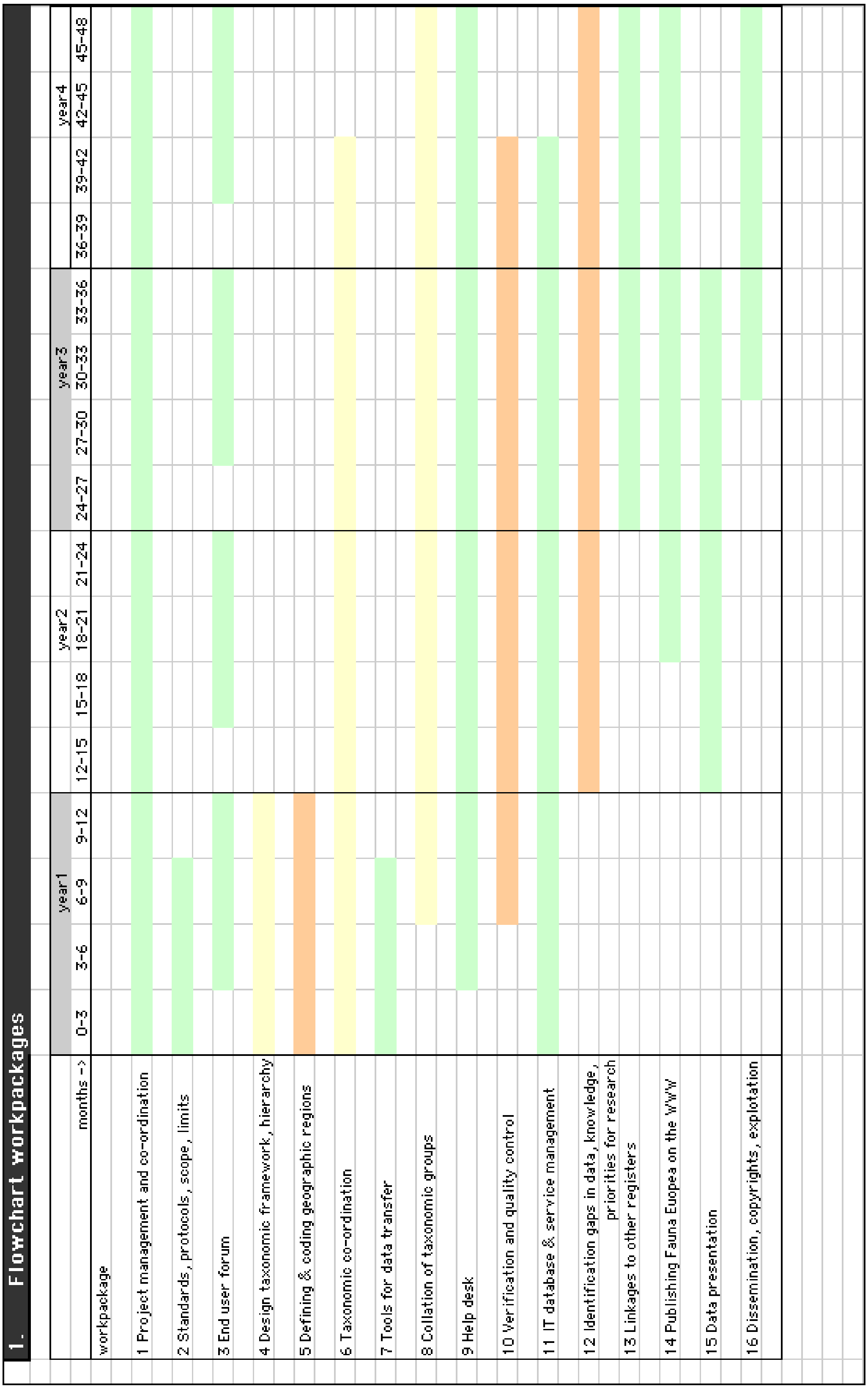

3-3 Graphical presentation of the projects components

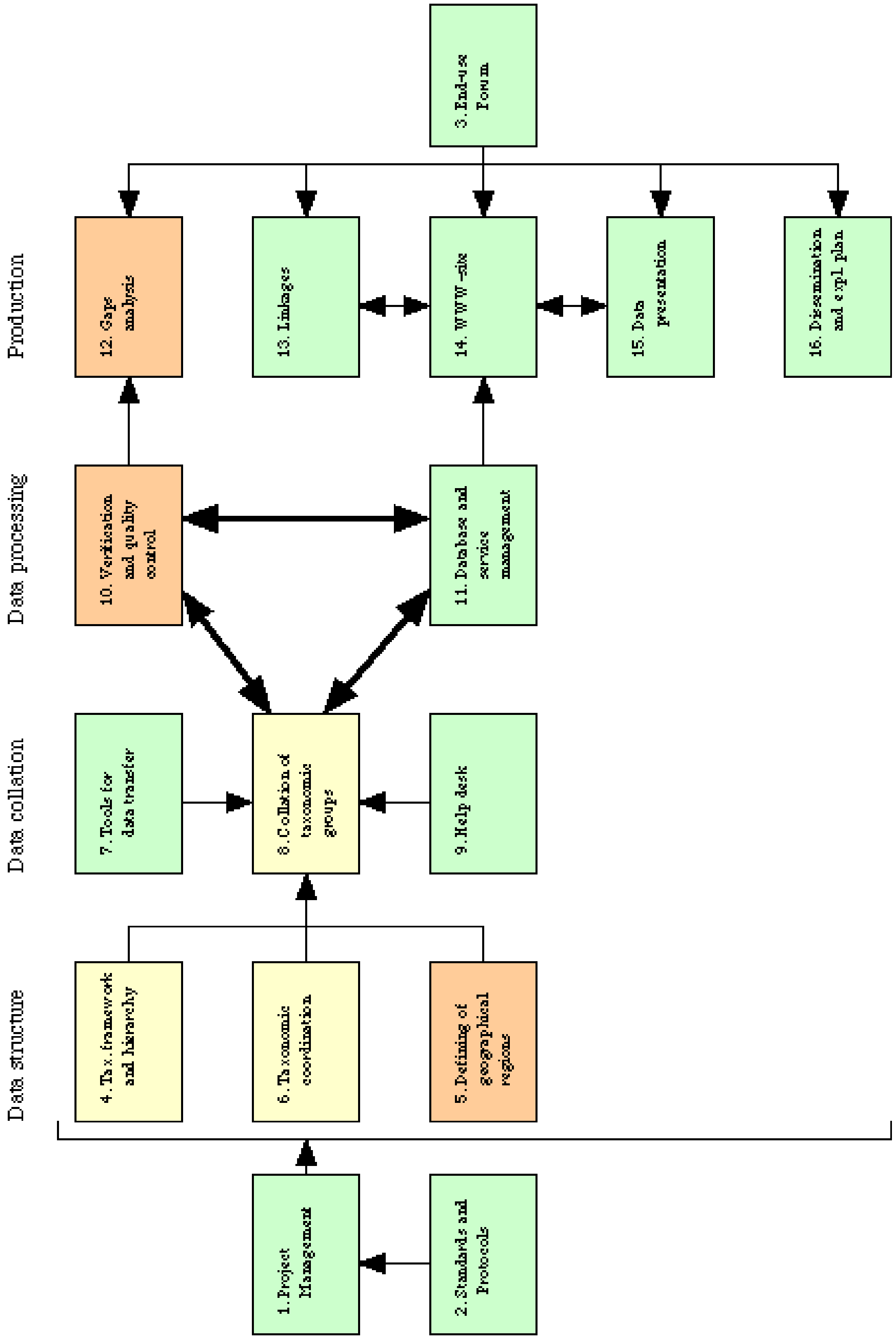

3-4 Workpackages

Each work package leader is responsible for the corresponding deliverables.

- 1 UVA - University of Amsterdam / Zoological Museum Amsterdam
- 2 ZMUC – Zoological Museum, University of Copenhagen
- 3 MNHN - Muséum National d'Histoire Naturelle

Work-package No	Workpackage title	Lead contractor No	Person-months	Start month	End month	Deliverable No
WP1	Project management and coordination	1	19	0	48	D1, D2
WP2	Standards and protocols	1	4	0	9	D3
WP3	End-user forum	1	14	3	48	D4
WP4	Taxonomic framework and hierarchy	2	5	0	12	D5
WP5	Geographic coverage	3	1	0	12	D6
WP6	Taxonomic coordination	2	22	0	42	D7
WP7	Tools for data transfer	1	20	0	9	D8
WP8	Collation of taxonomic groups	2	86	6	48	D9
WP9	Help desk	1	10	3	48	D10
WP10	Verification and quality control	3	33	6	42	D11, D12
WP11	IT databases	1	58	0	42	D13
WP12	Analysis of gaps in data and knowledge	3	14	12	48	D14, D15
WP13	Linkages to related databases	1	5	24	48	D16
WP14	Publishing Fauna Europaea on WWW	1	6	18	48	D17
WP15	Data presentation	1	3	12	36	D18
WP16	Dissemination and exploitation plans	1	5	30	48	D19, D20
	TOTAL		305			

Workpackage: Project management and coordination .

Workpackage number:	WP 1	
Start date / event:	Month 1	
Lead contractor:	UVA	
Person-months per partner:	UVA:	15 person-months
	ZMUC:	2 person months
	MNHN:	2 person months

Objectives and input to workpackage

Keeping focus on the projects objectives, in liaison with the Steering Committee (see chapter 9.1 Project Management). Monitoring the progress and logistics of the workpackages. Keeping track of all financial payments. Promoting synergy between the activities of the three principal contractors and their respective workpackages.

Description of work

- Employment and task assignment of project staff.
- Maintenance of a strict decision making structure.
- Monitoring of progress in the workpackages y a frequent internal reporting system.
- Establishment of advisory teams for each cluster, drawn from the members, depending on requested expertise.
- Regularly meetings for the integral project (Steering Committee), and for the cluster advisory teams.
- Written reports, and a newsletter on the WWW
- Supervision of correct operations of the help desk for the participating members

Deliverables

D1 Contracts with members.
D2 Reports to the European Commission.

Milestones and expected results

- Establishment of Steering Committee and Advisory teams in month 3.
- Two meetings of Steering Committee per year to monitor the projects progress.
- Timely results for each workpackage.
- Midterm report in month 24; Final report to European Commision in month 48.

Workpackage: Standards, protocols, scope and limits .

Workpackage number:	WP 2
Start date / event:	Month 1
Lead contractor :	UVA
Person-months per partner:	UVA: 2 person-months
	ZMUC: 1 person months
	MNHN: 1 person months

Objectives and input to workpackage

The execution of the project requires a strict focus on achieving results within the projects lifetime and budget. As a consequence, the principle contractors will define a set of rules – such as standards, protocols, scope, limits - to keep the project manageable.

Description of work

Within this workpackage, the principles for standards, protocols, scope, and limits of the project will be established . The standards etc themselves are part of other workpackages. Before detailing these, it is esential to have a common approach that will support a comparable and controlled input of data. This all is the basis for the specification and organisation of data flows, with the identification of responsible persons for these protocols.
The work starts with reparatory meetings of the advisory teams, leading to proposals from each principle contractor, responsible for its work field. Meetings of the Steering Committee, to assess the proposals, leading to decisions of the coordinator.

Deliverables

D3 Protocol and criteria to support the management decisions (workpackage 1) and background information for WWW site (workpackage 14).

Milestones and expected results

Management protocol repost in month 6.
Report on standards, scope and limits in month 8 (re-assesment in month 20)

Workpackage: End-user forum .

Workpackage number:	WP 3
Start date / event:	Month 3
Lead contractor :	UVA
Person-months per partner:	UVA: 8 person-months<br>ZMUC: 4 person months<br>MNHN: 2 person months

Objectives and input to workpackage

The results of the Fauna Europapaea project will serve the European Community at a wide level. A number of project members represent the end-users of the project. The end-user forum defines its recommendations and assist in the implementation of the project.

Description of work

The end-user forum will organise a number of meetings to discuss priority setting and the final data presentation, including linkages to other databases.
Inventory of the desirable attributes, as additional information on conservation or legal status, to the species checklist. Subsequent selection of those attributes to be included in the data fields (input for workpackage 13, 14 and 15). The members in the end-user forum will organize opportunities for exchange of views with a variety of (potential) users of fauna europaea. These users originate from the sectors of science, environment, agriculture, health, biotechnology, nature conservation, publishing.
Implementation by some members of parts of the WWW-site and external linkages.
Analysing possible applications of the project results, as an input for workpackage 12 and 16.

Deliverables

D4 Yearly reports of end-user recommendations, as input for workpacakes 12, 13, 14, 15, and 16.

Milestones and expected results

Yearly reports on the end-user recommendations (months 12, 24, 36, 48)

Workpackage: Design of taxonomic framework and hierarchy .

Workpackage number:	WP 4
Start date / event:	Month 1
Lead contractor number:	ZMUC
Person-months per partner:	ZMUC: 4 person-months<br>MNHN: 1 person month

Objectives and input to workpackage

1) Description of the taxonomic scope (which taxa), detail (primary data fields, synonyms, etc.), and attributes (end notes); input from workpackage
2) Tuning to other European master species databases.
3) Definition/construction of that classification/system above the genus level, that will support search and retrieval.

Description of work

The taxonomic framework and hierarchy will define the database structure and search & retrieval procedures. In animal systematics this is a very complex matter, as hierarchies are not always solved and generally agreed upon. As the taxonomic framework and hierarchies are of a conceptual nature, the best approach is to choose for stable systems that are practible for common use.
Meetings of the Steering Committee, partly jointly with the members Species 2000 and European Register of Marine Species project, will evaluate preparatory reports by the project staff, leading to decisions about the format for the data input from member experts (group coordinators),
Classifications / systems adopted above family level rank are decided upon in these meetings of by member experts (group coordinators, sometimes in advisory groups). Arrangements within any given family are decided on in consultation between group coordinators and individual contributing experts.

Deliverables

D5 Background information on taxonomic framework and hierarchy, to be published with the WWW site (workpackage 14), and input for workpackages 6, 7 and 8.

Milestones and expected results

Completed report to members describing the taxonomic framework and hierarchy (month 12).

Workpackage: Defining and coding of geographical regions .

Workpackage number:	WP 5
Start date / event:	Month 1
Lead contractor number:	MNHN
Person-months per partner:	MNHN: 1 person-months

Objectives and input to workpackage

Decisions on the geographic coverage (outer limits), the geographic detail (political territories, grids), and of its presentation (coding).

Description of work

Assessment of existing systems; interaction with Plant Euro+Med project and with EEA-topic centre biodiversity. Choice of system, in consultation with workpackage 6. Setting criteria for species selection at the European geographic borders. The objective is to follow more or less identifyable biogeographic zones.
The initial approach is that the sea shore is one of the geographical limits, including all European islands (in the Atlantic Ocean also Canary islands and Madeira; in the Mediteranean up to Cyprus). To the East, the intended limit is the Ural, thus restricted to biogeographic ‘minimal Europe’. Turkey, except European Turkey, is not a part of the project.
The information about the geographical distribution of species (optional) will at least be on country level (political units), but only if available and in the correct format.

Deliverables

D6 Definition of geographic limits and regions as input for workpackage 7 and 8.

Milestones and expected results

Report on geographic coverage for members to be applied in data collation (month 12).

Workpackage: Taxonomic coordination .

Workpackage number:	WP 6
Start date / event:	Month 1
Lead contractor number:	ZMUC
Person-months per partner:	ZMUC: 16 person-months
	UVA: 2 person months
	MNHN: 4 person months

Objectives and input to workpackage

Organising the network of group coordinators / taxonomic experts (as represented by various members), its work logistics, and the data flows.

Description of work

Establishment of a hierarchy of experts, from scientists in a relatively small group, through coordinators for a specific taxonomic group (such as a family or order), up to coordinators for an assembly of taxonomic groups. This approach will on one hand promote the best choice of experts, and at the other hand a decision-making structure with respect to the selection of data and their formats. The work will be organized and managed by establishing a hierarchy of experts and group coordinators (for taxonomic groups on various levels). This implies a study of the best way to combine at one the theoretical optimal approach, and at the other hand the involvement of available expertise. Establishing the whole framework and the logistics of taxonomic coordination requires a caraful approach to ensure the support of the wide European taxonomic community that will contribute with numerous data. As expertise and data from various data will overlap, and sometimes be in mutual conflict, the taxonomic coordination will also develop mechanisms to identify these and to establish mechanisms to harmonize the data input.

Deliverables

D7. List of contributing experts in various taxonomic groups, to be published in the WWW site (workpackage 16), and identification of missing expertise; and input to workpackage 12.

Milestones and expected results

List of contributing European experts in various taxonomic groups, to be published in the WWW site (initial: month 12 – final: month 48)

Workpackage: Tools for data transfer .

Workpackage number:	WP 7	
Start date / event:	Month 1	
Lead contractor number:	UVA	
Person-months per partner:	UVA:	18 person-months
	ZMUC:	2 person-months

Objectives and input to workpackage

Support for data transfer from data sources of unrelated formats. Application of standard export / import routines to transfer the distributed datasets into a common format.
In case of not yet databased information, the application of a user friendly software data entry module that will act as standard transfer unit and also can assist the project's members to import their data from existing databases, independent from its original format (printed, digital in various systems).

Description of work

- Making an inventory of existing databases and their structures (data models) of project members.
- Designing a common data exchange format to facilitate transfer of data to a central database.
- Application of a user friendly software data entry module that will act as standard transfer unit and assist the project's members to import their data from existing databases, independent from its original format (printed, digital in various systems). This module will also be used for generating new databases (in standard format) by members that do not own databases.

Deliverables

D8 Operating tool to assist workpackage 9.

Milestones and expected results

Data entry and transfer software module for exchange of data files, in operation from month 9.

Workpackage: Collation of taxonomic groups .

Workpackage number:	WP 8	
Start date / event:	Month 6	
Lead contractor number:	ZMUC	
Person-months per partner:	ZMUC:	66 person-months
	UVA:	10 person-months
	MNHN:	10 person-months

Objectives and input to workpackage

The data flows from member-experts will results in datasets of different size, detail and quality. The sets will be integrated (assisted by workpackages 7 and 9) in larger databases, ready for validation (workpackage 10), and subsequent storage (workpackage 11)

Description of work

Group coordinators (members) contact the taxonomic experts and national coordinators, who can provide partial datasets. Instructions from workpackages 4, 5 and 7 assisted by the help desk (workpackage 9), support the implementation of required data flows. Also identification of experts to do initial revisions in relation to the collation of datasets from different sources.
The lead contractor for this workpackage keeps contact with all experts according to the established logistic mechanisms. Precise instructions should promote data capture according to defined formats. In reality, it is expected that this will not be that simple, as the delivered datasets will be in many different formats, both with respect to data structure (and inclusiveness), and to data presentation (different database systems, various printed, sometimes even hand written, lists). In interaction with the experts, via the group coordinators the data flows will be optimized. In many times the datasets have to be digitized or transferred in other database systems, afterwards to be checked again by experts. Combined datasets to be checked for internal consistency. Much of spped and quality of work will depend on well established standards and protocols (workpackage 2), taxonomic framework and hierarchy (workpackage 4), coordinating mechanisms (workpackage 6), and tools for data transfer.

Deliverables

D9 Integrated data files to be validated (input to workpackage 10), resulting in partial datasets for inclusion in FE databases (workpackage 11).

Milestones and expected results

Completed databases on taxonomic groups, throughout the project period.

Workpackage: Help desk .

Workpackage number:	WP 9
Start date / event:	Month 3
Lead contractor number:	UVA
Person-months per partner:	UVA: 6 person-months
	ZMUC: 4 person-months

Objectives and input to workpackage

- Providing on-line assistance to the members (experts) with regard to data collation
- Assist members with using the data entry module (instructions)
- Provinding technical assistance in keeping the main databases on-line

Description of work

The tools for data transfer (workpackage 7) are essential for a smooth progress of the project. It is expected that the large number of contributing experts will face frequent problems with respect to managing and processing their data according to the requested format. The work plan is as follows.
Setting up structure for telephone, and e-mail service to assist member-experts with any emerging problem in relation to their work to build their specialist dataset or to transfer it. Advice with respect to taxonomic choices or priority setting.
The help desk will be established to provide a service for all possible expected problems. It will be active during the whole period of data collation. Start up in month 3, but in operation from month 6. In principle all services will be on-line or via telephone or fax. The operations of the help desk will be assessed with respect to its capability to solve any disturbance in the process of data collation.

Deliverables

D10 Operational help desk to assist for input for workpackage 8.

Milestones and expected results

Helpdesk in operation from month 6, and functioning up to the project end (month 48).

Workpackage: Verification and quality control .

Workpackage number:	WP 10
Start date / event:	Month 6
Lead contractor number:	MNHN
Person-months per partner:	MNHN: 30 person-months

Objectives and input to workpackage

Reviews of delivered data sets from workpackage 8. Assessment according to defined standards, protocols, scope, and to taxonomic framework. Assessment of data presentation (work package 15). Identification of gaps in information and expertise as input for workpackage 12.

Description of work

Development of measures (indexes) of the data quality. Records to be assessed as a whole or through each field. Levels are: data entry (standards), data semantics, data accuracy, data reliability, and inclusiveness of data. Finally identification of gaps in data, knowledge and expertise. The lead contractor of this workpackage will ask various network members to assist in the work. After development of data quality indicators, the work involves the verification of all incoming data sets from contractor ZMUC. Any comments will result in a feed back to ZMUC, such as to assess whether changes / improvements are possible. Quality problems of a general nature will be put forward to the Steering Committee with the objective to find a solution within the scope of the project. The work results in the assignment of meta data to data sets that will express the state of validation.

Deliverables

D11. Report on the criteria for quality control applied, the checking procedure followed, the resulting decisions (at a general level) – month 12.

D12. Assessment of the collated data sets, and input for the work package 18 – From month 12 up to month 42

Milestones and expected results

Report on criteria in month 12.
Periodic assessed datasets as input for workpackage 11.

Workpackage: IT database and service management

Workpackage number:	WP 11
Start date / event:	Month 1
Lead contractor number:	UVA
Person-months per partner:	UVA: 48 person-months
	ZMUC: 6 person-months
	MNHN: 4 person-months

Objectives and input to workpackage

- Building a central database (contents) according to the taxonomic frame work and hierarchy (workpackage 4) to accommodate incoming data sets.
- Experience in this workpackage will give input to the workpackage 9 (help desk).
- Links from the central database to the the Fauna Europaea WWW site (workpackage 14) which will allow users to view and search in the database.

Description of work

- Design of data model for the Fauna Europea central database. The data model has to reflect at one hand the results of work packages 2, 4 and 5 (taking into consideration the recommendations of the international Taxonomic Data Working Group) and at the other hand the latest database software developments.
- Building the Fauna Europea central database with links for data transfer, as this database will be central base for feeding new data and for extracting selections of data sets. As such, the work involves the development of a flexible, easy to adapt (taxonomic) structure, storage of various types of data (also potential mutimedia data, and derived data as sequences), options for linkages to supporting databases.
- Create linkage between the Fauna Europea central database and the Fauna Europea WWW site (workpackage 14)
- Service management includes the development of a transparant structure, that will guarantee that in future the database can be updated easily and can be adopted to new hardware and software requirements. A minimal task is to document the datastructure of the central database. The datastructure will have to meet ISO standards, which at least implies careful documentation.

Deliverables

D13 Fauna Europaea Database accommodating all incoming data from members with links to WWW site

Milestones and expected results

Data model for Fauna Europaea database (month 12)
Continuous building of database (contents) up to month 42.

Workpackage: Identification of gaps in data and knowledge .

Workpackage number:	WP 12
Start date / event:	Month 1
Lead contractor number:	MNHN
Person-months per partner:	MNHN: 6 person-months
	ZMUC: 2 person months
	UVA: 2 person months

Objectives and input to workpackage

In the course of the project, it will become apparent that not all data can be captured for different reasons:

- -no experts are available,
- -capturing the data will require fundamental taxonomic revisions that are not included in the project objectives,
- -the quality of the data is low or insufficient, and presently cannot be enhanced,
- -the data cannot be obtained within the projects time frame, budget of required format

Description of work

While assessing the inclusiveness and quality of the delivered data (workpackage 10), the gaps in data and in knowledge will be identified. The gaps will be listed with respect to data availability, data reliability, lacking scientific knowledge, and missing scientific expertise in Europe. The network members will assist in identifying the gaps.
In conjunction with workpackage 10 (verification and quality control), all emerging problems with respect to inclusiveness and quality of the data will be listed. If, after feed-back to ZMUC and consideration in the Steering Committee, it has been concluded that no further improvement is possible because of the current state of scientific knowledge, the problems will be analysed. Analysis should result in the identification of importance of gaps and priority setting and recommendations for European research and staffing policies in animal systematics.

Deliverables

D14. A list of gaps in data and knowledge, ordered by taxonomic group and/or geographical region,

D15. Recommendations to fill in the gaps (training of required experts, priority research areas, developments in biodiversity informatics)

Milestones and expected results

Report on gaps in data and knowledge, and in addition recommendations (month 48)

Workpackage: Linkages to other registers .

Workpackage number:	WP 13
Start date / event:	Month 24
Lead contractor number:	UVA
Person-months per partner:	UVA: 3 person-months
	ZMUC: 1 person months
	MNHN: 1 person months

Objectives and input to workpackage

Providing interoperability with the other related European taxonomic services and registers.

Description of work

Creating dynamic links from the Fauna Europaea central database via its webbrowser interface to other related taxonomic dataservices. Various user groups will ask for advanced mechanisms to enter the database or to extract (partial) datasets. The input from the End-user forum (workpackage 3) will be jointly assessed by the principle contractors, such as to specify the development work by UVA.

Deliverables

D16 Operational link between Fauna Europea database and other related taxonomic dataservices.

Milestones and expected results

Operational dynamic links for Fauna Europea database to other taxonomic databases

Workpackage: Publishing the Fauna Europaea register on WWW

Workpackage number:	WP 14
Start date / event:	Month 18
Lead contractor number:	UVA
Person-months per partner:	UVA 6 person-months

Objectives and input to workpackage

Producing webpages to present Fauna Europea in the Internet. Linking the webpages to the underlying central Fauna Europea database (package 11). Providing browse and functional search functions via Internet in the Fauna Europea database

Description of work

- Linking webpages to Fauna Europea database. The central database is the container for all data, but not always completely accessible. The WWW register will not necessarily give access to all data, as they can be partially under (re)validation or (re)construction. An operator interface between the central Fauna Europaea database and the WWW-service will allow to provide a reliable information service from the container database. The work involves designing and developing of the linking interface, with its control mechanism of selective linkages.
- Creating browse and functional search functions via Internet in the Fauna Europea database. Focus will be on the development of a (performance powerful) taxonomy browser that allows for a fast and user-friendly access to the complex taxonomic data and hierarchies.

Deliverables

D17 WWW-site and register of Fauna Europaea

Milestones and expected results

WWW register completed in month 48

Workpackage: Data presentation .

Workpackage number:	WP 15
Start date / event:	Month 12
Lead contractor number:	UVA
Person-months per partner:	UVA: 2 person-months<br>MNHN: 1 person months

Objectives and input to workpackage

Design and development of the Web-interfaces that will display the data in the WWW-site (workpackage 14), taking into account the non-stable nature of the data and the requirements of end-users (workpackage 2).

Description of work

Study of the data variability and of potential use of the Fauna Europaea databases in consultation with the end-user members. Prioritising the results as the basis for requirements. Users will access the WWW service via the Fauna Europaea interface.
UVA will design solutions for alternative ways of data presentation and for user-friendly methods to generate these.
A special feature is the development of interfaces (templates) in different European languages.
Testing on various user groups.

Deliverables

D18 Lay-out of Web-interfaces, and linkages to underlying software programs and databases.

Milestones and expected results

Report on data presentation in month 36.

Workpackage: Dissemination, copyrights and exploitation plans

Workpackage number:	WP 16
Start date / event:	Month 1
Lead contractor number:	UVA
Person-months per partner:	UVA: 4 person-months<br>MNHN: 1 person months

Objectives and input to workpackage

Make the Fauna Europea register available to users.
Make arrangement for copyright agreements with owners of the data (members). Market the Fauna Europea register by offering the service to the user groups. Sell the cd-rom with the Fauna Europea database for stand-alone use

Description of work

- Study and implementation of mechanism to allow the contributing experts to keep control on the quality and updating of the data, for which a 'Society for the management of biodiversity data' is considered.
- Preparation of contracts to guarantee continuity of Fauna Europaea after this projects period.
- Liaise with users concerning terms for using the on-line Fauna Europea register. Liaise with scientific publishers for marketing the Fauna Europea cd-rom.

Deliverables

D19 Mechanism to keep control on continuity of Fauna Europaea, including contracts with scientific publishers for distribution and sales
D20 Technology Implementation Plan

Milestones and expected results

- Technology Implementation Plan in month 48
- Report in month 48
- CD-ROM preparation ready in month 46

4 CONTRIBUTION TO OBJECTIVES OF PROGRAMME/CALL

Fauna Europaea contributes to “Support for Research Infrastructures”. Presently the databases and the expertise on the European animal species are scattered around numerous public and private organisations, they are in different formats and standards, and are of unequal quality. Some databases are restricted to a single taxonomic group, others only to country or other geographic level. Older different taxonomies have resulted in a complex situation with respect to the overview of the European animal species. The project will address the problem by encouraging the transnational use of excisting public and private databases, improving their exploitation, and by covering priority needs.
This project will bring together the European experts with authority in each major taxonomic group. By networking of researchers, database custodians and users, an unique effort will result in building and maintaining an informatics infrastructure that will support the collation of harmonized and validated taxonomical data, as well as offer access to these data to a wider user community. This will also help in identifying gaps in expertise and knowledge in taxonomy and in database infrastructure. A report on the results of this analysis will contribute to more focussed research activities and avoid duplication of efforts that otherwise should not be noticed.
Community interest is supported as the Fauna Europaea project will contribute to the EU commitments with respect to the Convention on Biological Diversity, especially with regard to the obligation to document the European diversity. It thus also contributes to the Clearing House Mechanism, and provides a directory to the Global Biodiversity Information Facility (GBIF) by its cooperation with the Species 2000 initiative. As such, it will promote the competitive European position by developing innovative IT tools for data collation, search and retrieval.
The plannned activities, in parallel with the related projects on plant and marine species, will offer a stimulus for European research in taxonomy and biodiversity. The Fauna Europaea network brings together a wide research community, and its work will promote further scientific initiatives.

5 COMMUNITY ADDED VALUE AND CONTRIBUTIONS TO EU POLICIES

The Community Biodiversity Strategy of the European Commission provides the framework for developing Community policies and instruments in order to comply with the Convention on Biological Diversity. The strategy recognises the current incomplete state of knowledge at all levels concerning biodiversity, which is a contraint on the successful implementation of the Convention. It asks for establishing networks between European centres of excellence in biodiversity research. One of the main themes in the Strategy is the intended support for studies to identify and catalogue the components of biodiversity, of which a database on the European taxonomic diversity is a basic tool for science and conservation policies. Fauna Europaea addresses this priority by compiling (University of Copenhagen – Zoological Museum), validating a catalogue for the terrestrial and fresh water animal species (Muséum National d'Histoire Naturelle), and presenting the catalogue in an advanced WWW environment (University of Amsterdam – Zoological Museum). Such an overview does not exist at an European scale, except for a limited group of species within the EUNIS system of the EEA Topic Centre on Nature Conservation.

Partial overviews are scattered around Europe in different scientific institutes, while only some countries are working on national information systems. As such, it provides support for further development of the Clearing House Mechanism, the prime vehicle for international information exchange on biodiversity, managed by the European Environment Agency.

Science and policies regarding to biodiversity in Europe depend on a good knowledge of its components. The assessment of biodiversity, monitoring changes, sustainable exploitation of biodiversity, and much legislative work depends upon a validated overview of taxonomic biodiversity. However, not all data are in a simple way available and in the requested format. By bringing together the community of experts, the project will promote the collation of databases of different formats, but also help to identify gaps in knowledge with respect to data on taxonomic groups (not well studied taxa or discutable taxonomies), geographic distributions (lacking or inadequate sampling), and with respect to not available expertise / experts. A report on this analysis will deliver the EU input for regional, national and community research policies.

6 CONTRIBUTION TO COMMUNITY SOCIAL OBJECTIVES

6.1 Quality of Life, Health and Safety of the Citizens

In various aspects of the quality of life of Europeans, it is essential to have access to a catalogue of the correct names of animal species. Health and safety depend on this correct information. The users of Fauna Europaea will apply the information source to their fauna-related products to standardize and thus facilitate communication about the names of animals, thereby ensuring that, based on available expertise, a name is effectively referring to the same species Europe wide. The use of thesauri in European cross border communication, and the Fauna Europaea service will indeed function as a thesaurus, is becoming increasingly important for numerous reasons, the political, administrative and economic integration being a foremost factor. Apart from the thesaurus function, the users will also, through the addition of straightforward country level information about the distribution of species, know which countries will be concerned with the management of these species in the widest possible sense, for whatever purpose.

6.2 Environment and Natural Resources

Community interest is supported as the Fauna Europaea project will contribute to the EU commitments with respect to the Convention on Biological Diversity, especially with regard to the obligation to document the European diversity. It thus also contributes to the Clearing House Mechanism, and provides a directory to the Global Biodiversity Information Facility (GBIF) by its cooperation with the Species 2000 initiative. As such, it will promote the competitive European position by developing innovative IT tools for data collation, search and retrieval.

6.3 Employment, Education, Training and Working Conditions

The result of the Fauna Europaea project also will have a considerable effect on expert taxonomic users. The peers of the compilers/authors/contributors of Fauna Europaea (and their successors during the next century) will critically review the list and its sources, and attempt to improve its quality through additional taxonomic, nomenclatural and faunistic research. Fauna Europaea will thus over time continue to gain in quality and usefulness -- certainly as long as the end user groups mentioned are indeed kept in mind.

The project contributes to Community social objectives, as the following end user groups will benefit from Fauna Europaea:

- government law-making agencies on various administrative levels
- environmental authorities on various administrative levels
- public and private water, air and soil management agencies

- the conservation community
- customs and other law-enforcing agencies - governmental statistics agencies
- the agricultural, forestry, fisheries and ecotourism industries
- the plant protection, veterinary, and human health services
- the scientific non-expert (non-taxonomic) community (particularly the ecologists)
- biodiversity collection and information management agencies (eg museums, herbariums, information centres)
- popular scientific publishing community (encyclopaedias, faunal field guides, handbooks etc) - the teaching community (from primary school level to universities)

7 ECONOMIC DEVELOPMENT AND S&T PROSPECTS

7.1 Exploitation and Dissemination of Results, IPR

Fauna Europaea is the result of European wide cooperation of experts and will in future stay dependent on these experts. The continuity and updating of Fauna Europaea will be organized in a structure that will guarantee the direct involvement of the experts. In liaison with the related projects on the marine and the plant organisms it is considered to establish a society for the management of biodiversity data, of which the members will be the contributing experts. This society will go in contract with hosting organisations to agree on (financial) mechanisms to keep Fauna Europaea updated, but also to exploit the information resources. The experts will thus keep control on the quality and updating of the data. For parts of the data it will be necessary to make copyrights agreements with owners of the data. The project will promote the involve of these owners in the management of the Fauna Europaea database. This database is an asset that will be exploited via several ways of commercial dissemination. However, the basic data will be in the public domain via the Internet. Commercial dissemination will focus on value added products or release of subsets of the data on CD-ROM or in print. A dissemination and use plan for the consortium as a whole and for the three contractors will be detailed at the end of the project.
Community interest is supported as the Fauna Europaea project will contribute to the EU commitments with respect to the Convention on Biological Diversity, especially with regard to the obligation to document the European diversity. It thus also contributes to the Clearing House Mechanism, and provides a directory to the Global Biodiversity Information Facility (GBIF) by its cooperation with the Species 2000 initiative.
In relation to the views in this paragraph, the Consortium will prepare a Technology Implementation Plan. Before the end of the project such a plan will be finished, indicating all potential foreground rights and intentions for use and dissemination of the results, including a time table. A first version of this report will be presented in the mid term report of the project.

7.2 Economic Growth

As Fauna Europaea will be a new and innovative product, it will support and promote various developments in science and applications. The economic exploitation of the Fauna Europaea database will be directed at establishing contracts in relation to these developments, and as such contribute to the continuity of the database.

7.3 Scientific and Technological Prospects

This plan, including reports on the present state of knowledge and expertise and the identification of gaps, will address the scientific and technological prospects of the Fauna Europaea project. A draft version of this plan will be presented in due course of the project.

8 THE CONSORTIUM

8.1 Overview of the Consortium

The three principal contractors (partners 1-3) will take responsibility for the main complementory clusters of tasks. These have been developed in preparatory meetings of a committee, established by the Linnean Society of London, (network member).

The *University of Amsterdam (Zoological Museum Amsterdam)* is in charge of the overall coordination and management, which includes the application of software and database tools to support these tasks. As coordinator, it will keep control over the project progress, and will bring decisions about priorities and choices into discussion. The coordinator will also build the Fauna Europaea main database, and develop mechanisms for dissemination and exploitation.
More specific, the University of Amsterdam is in charge of the workpackages 1, 2, 3, 7, 9, 11, 13, 14, 15, and 16, and contributes to the workpackages 6, 8, and 12.
Associated members are: (see explanation of numbers in 8.2)

The *University of Copenhagen (Zoological Museum)* will take care of the complicated task to collate the data for Fauna Europaea and merge these in integrated datasets. Cross checking of taxon based data files with country based data, and the inclusion of derived data (synonyms, conservation status), requires intensive contacts with experts throughout Europe, supported by the involvement of most network members.
More specific, the University of Copenhagen is in charge of the workpackages 4, 6, and 8, and contributes to the workpackages 1, 2, 3, 7, 9, 11, 12, and13.
Associated members are: (see explanation of numbers in 8.2)

The *Musée National d'Histoire Naturelle* in Paris decides on the quality of the data sets for publication. It will assign attributes to these sets in the main database, which will be a basis for selection in different kinds of dissemination. This role contributes to the task to draw a report on identified gaps in knowledge and expertise, including recommendations to tackle these in Europe.
More specific, the Muséum National d'Histoire Naturelle is in charge of the workpackages 5, 10, and 12, and contributes to the workpackages 1, 2, 3, 4, 6, 8, 11, 13, 15, and 16.
Associated members are: (see explanation of numbers in 8.2)

The members in the network are of three kinds.

End-users

A number of members (partners 4 – 8) represent different end-users in science and society. These members will assist in defining the outline of the final product and the ways to disseminate the results. These members are linked to the contractor University of Amsterdam.

The European Environment Agency (EEA)will be invited by the Fauna Europaea project as consultant to advice about its work.

Taxonomic experts
Most other members are involved by providing taxonomic expertise and information. They will partly act as group coordinators that will keep contact with the very large number of experts throughout Europe to capture data files on parts of the various taxonomic groups. Experts may be employed by the member institutes, but may also come from other institutes. The member institutes will work close with the University of Copenhagen (data collation) and the Musée National d'Histoire Naturelle (validation of data).

Support in information technology
Some members (7 – 9) will also assist in advise and support in information technology and the linkages to related database projects. These members are linked to the contractor University of Amsterdam.

8.2 Description of the participants

PRINCIPAL CONTRACTORS

COORDINATOR<br>1. Universiteit van Amsterdam (Zoological Museum Amsterdam) – The Netherlands

The University of Amsterdam is the coordinator of the National Research School of Biodiversity in the Netherlands, and of the Dutch initiatives with respect to the Global Biodiversity Information Facility. Its institute Zoological Museum Amsterdam (ZMA) manages a zoological collection as a large scale research facility. The collection covers various taxonomic groups with strenghts in the European, South-east Asian and Latin American regions, and in the marine environment. The research programme is directed at studies on speciation, evolution, biogeography and biodiversity assessment. The application of information technology is central to the programme.
The University of Amsterdam also manages large high performance computing facilities, and one of the strongest Internet nodes of Europe. The informatics (research) infrastructure supports the tasks of the IT work packages of this project.

2. University of Copenhagen (Zoological Museum) - Denmark

The Zoological Museum, University of Copenhagen (ZMUC), houses very large, global collections of all kinds of preserved animals. Each of the 17 collection curators is at the same time a researcher specializing in the global fauna of a particular animal group. The research at ZMUC is strongly concentrated around animal systematics and zoogeography, with phylogeny, evolutionary biology and biodiversity as important additional keywords.

ZMUC is a member of CETAF and in general has a very well-developed international network of connections with other museums. Further information on ZMUC's homepage: http://www.aki.ku.dk/zmuc/zmuc.htm.

3. Musée National d’Histoire Naturelle – Paris - France

The Museum National d'Histoire Naturelle (M.N.H.M.)is the French organism of reference for systematics. It keeps and manages the national natural history collections, which contains for its biological components more than 80 million specimens covering all groups of plants and animals both recent and extinct. The Institut de Systematique, federates 11 thematic laboratories devoted to research programs in biological systematics and phylogeny. The other units, focusing on ecology and environmental studies, are part of the "Institut d'Ecologie et de Gestion de la Biodiversité", which includes the "Service du Patrimoine Naturel", the national biological survey.
At present the M.N.H.N. is conducting research on all major groups of organisms at various integration levels, a number of researchers being experts in their field (80). Data control and validation will be the M.N.H.N. first task within this project given its technical and scientific skills developed in establishing check-lists (CLEMAM), databases of taxonomic objects (GICIM, SONNERAT, etc.) and classical taxonomic works.
The MNHM is also the leader institution of the Consortium “European Centre on Nature Conservation” of the European Environment Agency.

MEMBERS

4. The Linnean Society of London – UK .

The Linnean Society of London is a global society for the professionals in biology, epecially in biodiversity studies. The efforts of the society to promote the scientific work for the implementation of registers of the European plant and animal species, have been essential for these developments. The Society established and supported a committee for the preparation of the Fauna Europaea project, that resulted in this EU application. Linked to contractor UVA.

5. The University of Reading
Species 2000

The Centre for Plant Diversity and Systematics at the University of Reading is one of the three UK centres established under the NERC Taxonomy Initiative, and with a permanent staff of 7 academics and 4 technical support. It has specialised advanced taxonomic research laboratories in a) Floristics (herbarium), b) Biodiversity Informatics and c) Molecular Systematics.
The Species 2000 Secretariat is at the Centre for Plant Diversity & Systematics in the University of Reading, UK, and operated by Prof. Frank Bisby, chair of the Species 2000

Team. It has a start-up staff, presently funded by the University and BBSRC, and is itself the subject of a Framework 5 Proposal. Species 2000 is creating a Catalogue of Life by federating many taxonomic databases, each providing the global synonymised catalogue for one major group of organisms: Legumes, Fish, Geometrid Moths etc. The Catalogue of Life will be available both at the Web site and as a CD-ROM. It will be used both as a resource in its own right, and as the index for an Internet linking system, for instance interlinking the European regional taxonomic systems of Fauna Europaea, the European Register of Marine Organisms, and the Euro+Med PlantBase.
Linked to contractor UvA.

6. Ecological Consultancy Services Ltd (EcoServe) – Dublin – Ireland .

EcoServe routinely conducts ecological surveys for industry, local authorities, and government agencies. It specialises in marine and freshwater biodiversity, but also works with associates on terrestrial ecology. The company analysed marine sublittoral communities around Ireland for the Celtic Seas Quality Status Report for OSPARCOM, is writing a framework for a Marine Biodiversity Action Plan for Ireland, and a 'Review of marine nature conservation initiatives' for the Bern Convention Council of Europe. The company is an end-user of the project results and has developed close links with other end-users. EcoServe manages web sites for 2 EU projects and several other projects. It co-ordinates 2 EU Concerted Action projects, including the European Register of Marine Species. It runs several e-mail list servers, including the European Marine Research Information Network in Biodiversity. EcoServe is an SME, has 5 scientific staff, all environmental sciences or marine ecology graduates.
Linked to contractor UVA.

7. GSF – Forschungsinstitut für Umwelt und Gesundheit – Neuberberg – Germany
Institute of Soil Ecology

The Institute assembles a wide variety of profound knowledge on the soil as habitat for living organisms. The scientists have high expertise in manyfold soil ecological fields and techniques such as soil ecology (microbiology including a large number of molecular techniques, zoology, food web dynamics with special respect to nutrient turnover), soil science, transport and transformation of contaminants in soils, modelling of matter transport and nutrient dynamics).
Linked to contractor UVA.

8. The Expert center for Taxonomic Identification (ETI) – Amsterdam – The Netherlands

ETI is a non-governmental organization (NGO) in operational relations with UNESCO. It has branches in Japan, Chile, Uruguay, Russia and England. This international center was set up in 1990 with the aim to develop innovative ICT tools specifically for biodiversity documentation and species identification (Linnaeus II Software and derivatives), to promote international collaboration and networking between specialists in the field of biodiversity studies (human resouce management, data mining), and to create a

mechanism for worldwide dissemination of electronic species identification guides and biodiversity information systems. ETI provides the science community with a production facility of CD-ROM based publications biodiversity expert systems and a gateway for on-line applications to be released on the Internet. The basic taxonomic data of ETI's central database are provided to the Species 2000 name registry.
Linked to contractor UVA.

9. University of Padova – Italy .

Research carried on at the Biology Department of the University of Padova covers a very broad range of pure and applied biological disciplines, from molecular biology, genetics and biotechnology to systematics, faunistics (and floristics) and ecology. Since more than ten years, the office of the *Fauna d'Italia* project (developed by the Italian Zoological Society (UZI) and the Italian Academy of Entomology) has been associated with this Department. Within this project, a series of monographs with identification keys and detailed distributional data are produced, each volume devoted to the species of a selected animal group as ascertained for the fauna of Italy and neighbouring countires. A comprehensive *Checklist of the species of Italian fauna* has also been produced: this cooperative effort, in which 272 specialists were involved, resulted in the first complete inventory of the animal species known form a whole country, with over 57 000 species listed.
Linked to contractor MNHN.

10. Museo Nacional de Ciencias Naturales – Madrid – Spain .

The Museo Nacional de Ciencias Naturales (MNCN) is the Spanish Institution of reference for animal biodiversity since it coordinates the FAUNA IBERICA project. It keeps and manages the national natural history collections covering all groups of animals both extant and fossils with a wide representation (historical collections and important recently collected materials) of the Iberian fauna, the richest of the European Community. The Department of Biodiversity and Evolutionary Biology conducts research on systematics, phylogeny, biogeography, speciation, reproductive biology, biodiversity patterns, conservation biology and biodiversity assessment. The other units are focused on evolutionary ecology, paleontology, geology and vulcanology. The MNCN maintains a highly developed web site including a Directory of Spanish Taxonomists (DIRTAX) and relevant information on Iberian biodiversity in its Fauna Ibérica section. Its expertise in the management of the Fauna Iberica project, data collation and networking of experts will be connected with the tasks of the Fauna Europaea project.
Linked to contractor MNHN.

11. National and Kapodistrian University of Athens – Greece
Zoological Museum

The Zoological Museum of the University of Athens is the oldest zoological institution in Greece dating from the middle of the 19th century. It has large collections of animals and animal parts from Greece and the Balkans. The staff of the Museum has experience in dealing with biogeographical problems (various studies on the biogeography of mainland Greece and the Aegean islands), with endemism (endemism of terrestrial gastropods, terrestrial isopods, ants, beetles of Greece), with conservation problems (conservation biology of monk seals, raptors, reptiles, amphibians, invertebrates), with data collation (Greek Fauna Documentation Centre, Register of Marine Species, data base on the fauna of Greece) and with computer applications in Biogeography.
Linked to contractor MNHN.

12. Norwegian University of Science and Technology – Trondheim – Norway
Institute of Natural History

The institute established an overview of the Norwegian taxonomic biodiversity. This regional expertise on the Nordic biodiversity is vital for the project.
Linked to contractor ZMUC.

13. Naturhistorisches Museum Wien – Austria .

The Naturhistorisches Museum in Vienna is the main expert institute and custodian of biological collections in Austria. Its scientists are specialists in various taxonomic groups.
Linked to contractor ZMUC.

14. Institut Royal des Sciences Naturelles de Belgique – Brussels – Belgium .

Département d'Entomologie
Institut Royal des Sciences Naturelles is the central Belgium institute for natural history collections. Its large collections provide the basis for expertise on variuous taxonomic groups.
Linked to contractor ZMUC.

15. Forschungsinstitut und Naturmuseum Senckenberg – Frankfurt a M. – Germany

One of the major Natural History Museums in Germany and Europe. Main research area is the biodiversity of continental, marine, and fossil systems. The organisation includes departments of zoology, marine taxonomy working group, botany incl. paleobotany, paleozoology, messel, paleoanthropology, marine sciences within branches at Frankfurt (main institute), Bieber, Messel, Wilhelmshaven, Hamburg, Bremerhaven, List/Sylt. Fieldwork is carried out in the tropics as well as in central Europe. The scientific staff includes 72 individuals. About 60 ph. D.-students.
Linked to contractor ZMUC.

16. Humboldt University at Berlin - Germany
Museum für Naturkunde

The Museum für Naturkunde is the largest museum of Natural History in Germany. It houses ca. 25 Mio objects (zoological, paleontological, mineralogical). In the zoological collections, the majority are the insects (ca. 15 Mio., 6 of which are beetles). The strength of our collections is its vast holding of types, especially in the Coleoptera, Diptera, and Lepidoptera (e.g., Staudinger collection). The museum belongs to Humboldt-University, the research departments are university institutes, i.e. we also provide teaching to university students.
Linked to contractor ZMUC.

17. Staatliches Museum für Naturkunde – Stuttgart – Germany .

This State Museum of Natural History is involved in various activities related to biosystematics and biological informatics at the national level. The museum will take responsibility for major parts of the taxonomic group of terrestrial Isopoda.
Linked to contractor ZMUC.

18. University of Erlangen – Nürnberg – Germany

The university will act as the group coordinator of the complex group of Orthopteroid orders.
Linked to contractor ZMUC.

19. Universidad de Salamanca - Spain
Unidad de Zoologia

The Unidad will contribute to collating the large taxonomic group of Hymenoptera.
Linked to contractor ZMUC.

20. Université Paul Sabatier – Toulouse - France

The Laboratoire de Zoologie will take leadership in collating the data in the taxonomic group of Entognathan Hexapoda. The Laboratoire d'hydrobiologie is in charge of the taxonomic group Ephemeroptera.
Linked to contractor ZMUC.

21. L'Université de Rennes 1 – France
Laboratoire de Parasitologie, Faculté de Medicine

The Laboratoire de Parasitologie will provide the group coordination to compile the data in the taxonomic group Siphonaptera.

Linked to contractor ZMUC.

22. University of Florence – Firenze – Italy .

The Department of Animal Biology and Genetics will contribute to the collation of data for Decapoda.
Linked to contractor ZMUC.

23. Universitá di Roma “La Sapienza” - Italy

The university contributes to capture the data for the very large group of Coleoptera.
Linked to contractor ZMUC.

24. Universitá della Tuscia – Italy

The university is charge of the taxonomic group of Plecoptera.
Linked to contractor ZMUC.

25. Stichting National Museum of Natural History - Leiden - The Netherlands

Having been assigned this task since its inception in 1820 the Leiden museum has always maintained complete species registers of the Dutch fauna, and continues to do so. Its publications include major series on the fauna of The Netherlands and adjacent regions, including the new book series Nederlandse Fauna and the journal Nederlandse Faunistische Mededelingen. There are close connections with the European Invertebrate Survey, including the Dutch branch of its expert network.
Linked to contractor ZMUC.

26. Wageningen Agricultural University - The Netherlands

The Wageningen Agricultural University mission is to support agriculture with knowledge. As a result its expertise in nematology is world famous.
Linked to contractor ZMUC.

27. Plantenziektenkundige Dienst – Wageningen – The Netherlands

The Plantenziektenkundige Dienst is in charge of the control of plant deseases in the Netherlands. Contributing specialism is in the large group of Hemiptera.
Linked to contractor ZMUC.

28. Instituto de Investigacào Cientifica Tropical – Lisboa - Portugal
Centro de Zooloia, I.I.C.T.

The institute will act as coordinator to collate the data for the Apterygote insects.
Linked to contractor ZMUC.

29. University of the Azores – Horta - Portugal
IMAR – Instituto do Mar

The institute has contacts will all regional experts and the data for the regional biodiversity of Madeira, the Azores and the Canaries.
Linked to contractor MNHN.

30. Naturhistoriska riksmuseet – Stockholm - Sweden

Basic biological research at the Swedish Museum of Natural History concentrates on the origins of animals and plants, their systematics, and their distribution in time and space. Studies of tropical biodiversity are emphasized, as well as the Swedish flora and fauna. Within the area of palaeontology, research is conducted in both palaeozoology and palaeobotany, including studies of the origin and development of land plants. Geological research at the Museum is concerned with systematizing the structural and chemical properties of minerals. At the Laboratory for Isotope Geology, the development of the earth's crust is studied by determining the age of rocks and minerals.
Linked to contractor ZMUC.

31. The Natural History Museum – London – United Kingdom

The Natural History Museum (NHM) is one of the few largsts natural history institutions in the world. Its very rich collections are curated by specilist in various groups, that support the research programme of the NHM.
The Department of Zoology is the largest and most diverse in the Museum and holds about 27 million specimens. The 121 scientists in the Department maintain and enhance the collections to study how animals have evolved and how different animal groups are related. The results have vital relevance to ecology, conservation and human health issues worldwide. Recently developed research techniques, such as DNA sequencing, are adding new dimensions to the traditional science of the Department.
Linked to contractor ZMUC.

32. University of Greenwich – United Kingdom

The university is involved in the NODE (Nonmarine Ostracod Distribution in Europe) GIS database, established as part of a previous EU-funded project and now develops its analytical applications. The Fauna Europaea project will clearly benefit from "clustering " with the DynamO project.
Linked to contractor ZMUC.

33. Societa' Romana di Scienze Natural

Societa' Romana di Scienze Natural contributes with ssential expertise on Scorpiones.
Linked to contractor ZMUC.

9 PROJECT ADMINISTRATION

9.1 Project Management

Scope and organisation

The management of the Fauna Europaea project within the the limits of budget and time, requires the establishment of an organisation with clear lines of authority and responsibility, and avoiding beaurocracy. For this reason the project is organized as a thematic network with three principle coordinators that each are responsible for a cluster of interrelated workpackages. The members contribute to one or more of these workpackages, coordinated and managed by the respective principal contractors. The coordinating institute, in charge of the whole project, will select a Steering Committee to support the management of the project. The composition of the Steering Committee will be a subject of discussion at the first general meeting of the Fauna Europaea project. The projects bureau, in charge of the day to day overall management of the project, will report to the Steering Committee as representative of the whole network.

The three principle contractors will discuss in regular meetings with the Steering Committee the overall organisation and progress of the project with respect to:

Work programme
- results of the coordinators
- expenditures
- methods of dissemination

Scope of project
- taxonomic framework
- taxonomic detail
- geographic coverage and presentation

Each of the principle contractors will keep contact with the members to keep control over:

Gathering of the data
- data gathering and timely delivery of a specific group
- contacts with the network coordinators
- deliverage according to the general lay out
- assigning specialists for specific groups

Transfer of data
- software entry modules
- help desk
- checking timely deliverage

Validation
- check according to protocol

- reviews of delivered data bases
  - updating of data bases

Technical database managent
  - management of the databases in various stages of validation
  - integration of subsets of database
  - maintenance of hardware and software
  - providing information via the Internet

Tuning to users needs and dissemination
- organisation of the user forum
- contracts for different formats of dissemination
- Contacts with related projects

Facility management
  - finance
  - administration

Decision making structures

The workpackages are allocated to each of the partners . Apart from the general management and coordination of the project, the work is organized into four fields, for which each principal contractor has its responsibility with respect to decision making.

-	collation of the data	University of Copenhagen – Zoological Museum
-	validation and gap analysis	Musée National d'Histoire Naturelle
-	database management	University of Amsterdam – Zoological Museum
-	user forum	University of Amsterdam – Zoological Museum

The decision making will be supported by ad hoc advisory teams, drawn from the members. The establishment and compisition of ad hoc advisory teams depends on identified specific problems that need consideration and advice from an ad hoc group. The steering committee, the principal contractors and the advory teams, will meet yearly once or twice to discuss the project progress and any emerging general problems. The coordinator will take the final decisions of general kind.
Two meetings of all members will be organised, one to overview the instructions for data capture, and a second one to discuss the policy with respect to apparent gaps in data and knowledge.

Communication flows

The project will give much attention to communication with its members. Apart from the scheduled meetings, the project makes full use of communication via the internet. An example is the help desk to assist members and their experts in data capture and transmission. Via Internet and e-mail messages, the principle contractors will keep everyone informed about the progress of the project, and about the interaction with the end-users forum. One of the coordinators project staff members is responsible for drawing up regular news letters. Apart from the general information flows, the communication on specific tasks with all members is being managed by each of the principle contractors to which the members are attached. Management of communication

is assisted by precise work instructions, specifications of reports (such as databases), and feed back mechanisms.
As the project will collate datasets from different sources and also validate these, one may expect that controversial opinions will emerge. These will be subject to discussion in the project Web pages, such as to assist the final decision making by the principal contractors.

Reporting to the Commission

Reports to supply will be six-monthly management reports, annual scientific and technical reports, and a final report. Cost statements will be submitted together with the annual reports.

Six- monthly management reports
Structured according to the tasks in the Description of Work, include:
- A summary of all the activities conducted in the preceding period
- A review of progress in respect to details in the Description of Work. For each task will be indicated the actual state of advancement with respect to the timing foreseen in the Description of Work
- Plans for the following 12 months detailed on the Work Package level including, if necessary, a proposal for adjustments
- An updated listing of publications submitted and published and presentations of project results made on scientific meetings. Publications must acknowledge the funding by the Commission.
- A list of personnel paid partially or fully from project funds will be included for each partner and updated regularly.
- Any other matter relevant to the assessment of the progress of the project.

Annual scientific and technical reports
The co-ordinator has the responsibility for submitting a consolidated report on the project. Scientific reports follow the same layout of work packages as the Description of Work.

A final report set including
- a publishable synthesis of the scientific results
- an abstract for electronic dissemination, which shows the objectives and technical results of the project (normally the same as the publishable synthesis of the scientific results)
- an extended abstract for the general public and decision makers (avoiding as far as possible very technical terminology)
- a final scientific and technical report.
- a CD-ROM of the whole data set.
- a short report on the dissemination and exploitation of results, including the Technology Implementation Plan.
- 1 reprint of all publications of the project

9.2 Manpower Matrix

Fauna Europaea - Allocation of person/months to workpackages

	1	2	3	4	5	6	7	8	9	10	11	12	13	14	15	16	TOTAL
UvA	15	2	8			2	18	10	6		48	2	3	6	2	4	126
ZMUC	2	1	4	4		16	2	66	4		6	2	1				108
MNHN	2	1	2	1	1	4		10		33	4	10	1		1	1	71
Total	19	4	14	5	1	22	20	86	10	33	58	14	5	6	3	5	305

(10) LIST OF REFERENCES AND RELATED PROJECTS

10.1 References

Not applicable

10.2 Related projects

This projects will attempt to establish strategic links to several other projects working on biodiversity research under the 5th Framework Programme, including ENHSIN, and several projects funded under the 4th Framework Programme, including ERMS.
The Consortium of European Taxonomic Facilities (CETAF), the consortium of the major European natural history institutions and collections, actively supports all these initiatives.

10.3 Project Cluster Participation

This project participates in a cluster of projects working on biodiversity research. The aim of the cluster is to determine and promote strategic approaches to biodiversity assessment and management in Europe. Other members of the cluster are: Biodiversity Assessment, BIOMAN, BIOSTRESS, CASCADE, EURO+MED, Fauna Europea, FOSSILVA, METABIRD, MIDI-CHIP, PLANT DISPERSAL, and TRANSPLANT. The cluster will help to co-ordinate and to provide common purpose to an important collective European effort to develop biodiversity research; provide an opportunity to enrich each project by an exchange of ideas; and, where appropriate, increase the effectiveness of each proposal through joint field work or other activities. The cluster may later be extended to include other projects, in particular projects arising from future calls for proposals related to biodiversity.

Cluster activities

a. Two cluster meetings will take place involving the co-ordinators, but including other partners where appropriate. The exact dates of the meetings will be fixed by mutual consent. The first will take place within a few months of the start of the projects and the second about 18 months or 2 years after the start of the projects.

b. The cluster will prepare a leaflet to announce the creation of the cluster, to be produced shortly after the first cluster meeting.

c. The cluster will prepare a newsletter that will contain information on the activities of the projects and other pertinent news.

d. The cluster will produce a brochure that summarises the main findings of the projects

and strategic recommendations for biodiversity research in the future. This brochure will be produced before half of the projects have submitted their final reports.

e. Participants in the cluster will exchange views on quality assurance and data management practices, and if possible, generate guidelines or recommendations on these issues.

f. Where only one or two participants in the cluster attend international or national conferences of interest to other members of the cluster, those participants will brief the other members of the cluster on any significant papers delivered;

g. The cluster will establish a web page allowing the partners of the projects in the cluster to advertise their activities and announce results, meetings, and publications.

h. Occasionally the Commission may require an answer to a policy-relevant question related to biodiversity. The cluster will provide a reasoned, rapid response to such questions, provided that such questions fall within the competence of the cluster and do not require additional research.

10.4 Acronyms

BioCISE	Biological Collections Information Service Europe
BIOMAN	Biodiversity and Human impact in shallow lakes
BIOSTRESS	Biodiversity in Herbaceous Semi-Natural Ecosystems under Stress by Global Change Components
CASCADE	Securing gene conservation, adaptive breeding potential and utilisation of a model multipurpose tree species (Castanea sativa Mill.) in a dynamic environment
CETAF	Consortium of European Taxonomic Facilities
CLEMAM	Check List of European Marine Mollusca
DIRTAX	Directory of Taxonomists
EC	European Commission
ENHSIN	European Natural History Specimen Information Service
ERMS	European Register of Marine Species
EEA	European Environment Agengy
ETI	Expert center for Taxonomic Identification
EUNIS	European Nature Information System
EURO+MED	European and Mediterranean Plant Database
FOSSILVA	Dynamics of forest trees biodiversity: linking genetic, palaeogenetic and plant historical approaches
GBIF	Global Biodiversity Information Network
GIS	Geographic Information System
ICZN	International Commission on Zoological Nomenclature
ICT	Information and Communication Technology
IT	Information Technology
METABIRD	Viability of bird metapopulations

MIDI-CHIP	Design and testing of DNA microarrays to monitor microbial diversity with adequate biodiversity indexes, using cyanobacteria in freshwater as a model system
MNCH	Museo Nacional de Ciencias Naturales (Madrid)
MNHN	Muséum National d'Histoire Naturelle (Paris)
NERC	National Environment Research Council (UK)
NGO	Non Governmental Organisation
NHM	The Natural History Museum (London)
NODE	Nonmarine Ostracod Distribution in Europe
SME	Small Medium sized Enterprise
TRANSPLANT	Extinction risks and the re-introduction of plant species in a fragmented Europe
UNESCO	United Nations Educational and Scientific and Cultural Organisation
UZI	Italian Zoological Society
WP	Work Package
WWW	World Wide Web
ZMA	Zoological Museum Amsterdam
ZMUC	Zoological Museum, University of Amsterdam

Addition to Description of Work of FAUNA EUROPAEA

Mention the following related projects

1) Biodiversity Assessment (EVK2-1999-280) - Institute of Terrestrial Ecology, ITE - Natural Environment Research Council (UK) - (36 months) - "Biodiversity Assessment Tools" will develop biodiversity indicators to assess the impact of policies on changes in biodiversity in Europe. It will take into account the major biological, cultural and geographical influences on biodiversity. This project will help Member States to monitor biodiversity in response to political, ecological, scientific and cultural needs.

2) BIOMAN (EVK2-1999-309) - Laboratory of Aquatic Ecology , Katholieke Universiteit Leuven (BE) - "Biodiversity and Human impact in shallow lakes" will develop reliable and cost-effective indices of biodiversity in shallow water bodies in Europe. It will develop models to predict the effect of human activities, and provide a reliable measure of the success of restoration measures. The main beneficiaries of this work will be those responsible for the management and restoration of shallow water bodies.

3) BIOMARE (EVK2-1999-00250) - Centre for Estuarine and Coastal Ecology, Netherlands Institute of Ecology (NL) - "Implementation and networking of large-scale long-term MARine BIOdiversity research in Europe" is a concerted action to establish the infrastructure and conditions required for marine biodiversity research

4) BIOSTRESS (EVK2-1999-270) - Institut für Pflanzenoekologie, Justus-Liebig-Universität Giessen (DE) - (36 months) - "Biodiversity in Herbaceous Semi-Natural Ecosystems under Stress by Global Change Components" will set up experiments to quantify the changes in semi-natural herbaceous communities when ozone concentrations increase. Project results will be useful to evaluate critical levels of ozone for semi-natural vegetation in the context of the UN/ECE Convention on Long-Range Transboundary Air Pollution.

5) CASCADE (EVK2-1999-065) - Istituto per l'Agroselvicoltura, Consiglio Nazionale delle Ricerche (IT) - (42 months) - "Securing gene conservation, adaptive breeding potential and utilisation of a model multipurpose tree species (Castanea sativa Mill.) in a dynamic environment" will examine the genetic characteristics of the chestnut and identify markers linked with disease- and drought-resistant strains. The project will evaluate the probable socio-economic costs and benefits of the management strategies that it will recommend to conservation bodies, ministries, foresters and farmers.

6) EURO+MED (EVR1-1999-00024) - Department of Botany (Centre for plant diversity and systematics), University of Reading (UK) - "European initiative for the Euro+Med

plantbase" will develop a catalogue of European plants, with synonyms, based on a consensus taxonomy.

7) METABIRD (EVK2-1999-164) - Department of Zoology, Norwegian University of Science and Technology (NO) - (36 months) - "Viability of bird metapopulations" will develop procedures to estimate the minimum size of viable bird meta-populations. It will develop recommendations for the management of spatially structured populations of endangered or threatened bird species. The results of this project will influence the design of bird monitoring programmes and in the planning of reserve networks such as Natura 2000.

8) MIDI-CHIP (EVK2-1999-213) - Department of Plant Biology, Université de Liège (BE) - (40 months) - "Design and testing of DNA microarrays to monitor microbial diversity with adequate biodiversity indexes, using cyanobacteria in freshwater as a model system" will develop and test DNA microarrays, or DNA chips, to provide rapid and low-cost genetic measurements of within- and between-species biodiversity. It focuses on the problem of understanding blooms of cyanobacteria in European lakes, but will provide a practical, economical and versatile instrument with far wider applications.

9) FOSSILVA (EVK2-1999-244) - Institut Méditerranéen d'Ecologie et de Paléoécologie, Faculté St Jérôme, Université de Droit, d'Economie et des Sciences d'Aix-Marseille (FR) - (36 months) - "Dynamics of forest trees biodiversity: linking genetic, palaeogenetic and plant historical approaches" will work out ways of using molecular markers to identify fossil trees, to understand the origin of modern forests, and how the gene pool has changed. This will help to develop strategies for the protection and enhancement of our natural heritage. The techniques of recovering and analysing fragments of fossil DNA will have implications for the study of other organisms.

10) PLANT DISPERSAL (EVK2-1999-246) - Department of Identity and Genetic Diversity, DLO - Centre for Plant Breeding and Reproduction Research (NL) - (36 months) - "Dynamics of plant dispersal-related traits in fragmented European habitats: consequences for species survival and landscape management" will examine the genetic variability in seed dispersal in several important tree species, to understand to what extent these species can evolve to persist in a fragmented landscape. The models developed will make it easier for farmers and conservation officials to manage woodlands and nature reserves.

11) TRANSPLANT (EVK2-1999-042) - Department of Ecology and Environment, Katholieke Universiteit Nijmegen (NL) - (48 months) - "Extinction risks and the re-introduction of plant species in a fragmented Europe" aims to understand how habitat fragmentation influences the extinction risk for key plant species. It will work out how to restore habitats and re-introduce species. The results will be useful to restoration ecologists and those responsible for environmental impact assessments.

Add the following tasks, to be performed jointly by the related projects

a) a cluster meeting at the start of the projects and a second round mid term, involving the co-ordinators, but including other partners where appropriate;

b) one leaflet to announce the creation of the cluster, to be produced as the projects start;

c) one final brochure summarising the main findings of the projects and strategic recommendations for biodiversity research in the future;

d) exchange and discussion on quality assurance and data management practices;

e) briefing other members of the cluster on significant papers delivered at colloquia or conferences which one or two partners attended;

f) a web page allowing the partners of the projects in the cluster to advertise their activities and announce results, meetings, and publications.
a new paragraph

g) Occasionally the Commission may require an answer to a policy-relevant question related to biodiversity. The cluster will provide a reasoned, rapid response to such questions, provided that such questions fall within the competence of the cluster and do not require additional research."
